# Forest Therapy Trails: Development and Application of an Assessment Protocol

**DOI:** 10.3390/ijerph22091440

**Published:** 2025-09-16

**Authors:** Paul H. Gobster

**Affiliations:** Northern Research Station, USDA Forest Service, 1033 University Pl. Ste. 360, Evanston, IL 60201, USA; paul.gobster@usda.gov

**Keywords:** forest therapy, forest bathing, forest trails, landscape assessment, protocol, key characteristics, guidelines, landscape design and planning

## Abstract

What key characteristics of forest sites and trails contribute to enjoyable and healthy immersive nature experiences for visitors? Previous research has helped identify the conceptual structure and criteria important in facilitating successful experiences, but there remains a knowledge gap in translating this information into operational guidelines. To address this gap, the present work used a descriptive inventory approach combining field research with a variety of secondary data to develop a protocol for assessing four site level criteria (beauty, integrity, tranquility, accessibility) and six trail level criteria (ease of travel, attractiveness of layout, natural features, built features, explorable nature, interpretation and stewardship). Through an iterative process, the protocol was refined and applied to selected sites (*n* = 56) and trails (*n* = 157) in two study areas in metropolitan Chicago, Illinois, and the rural Northwoods of Wisconsin. Qualitative and quantitative information describes preferred conditions across criteria, while quantitative ratings highlight commonalities and differences between urban and rural sites and trails. Although the principal focus was on foot trails, sub-analyses show how the criteria can apply to bike and paddle trails. With regional adaptations, the protocol has utility for the planning and design of new and management of existing trails for the growing practice of forest therapy and related forms of nature-based recreation.

## 1. Introduction

The belief that contact with nature can positively impact human health and wellbeing is longstanding and helped drive development of public parks beginning in the mid-nineteenth century [1,2]. Empirical support for this belief is much more recent and was spurred by researchers’ response to the environmental movement of the 1960s [3]. Since then, collaborations between the health, design, and natural resource related disciplines and professions have fueled considerable growth in the evidence base. This work has revealed the importance of nearby nature [4,5], nature connectedness [6,7], and nature experience [8,9] on physical and mental health and wellbeing. The research has led to a range of applied programs and interventions, from the installation of healing gardens in institutional healthcare settings [10] to the advocacy of park prescriptions given by doctors to recommend a dose of nature to their patients [11]. Among these tools, forest therapy is becoming a particularly promising health promotion vehicle for connecting people with nature.

As used in this paper, forest therapy refers to a range of activities and practices, including forest bathing (shinrin-yoku) and forest or nature walks, where individuals seek immersive, multisensory experiences in forests or other natural environments to obtain health and wellbeing outcomes [12,13,14]. While these experiences can take place in various ways, most forest therapy occasions rely upon slow, guided or self-guided walks on trails through sensory-rich environments so participants can more fully take in the sights, sounds, smells, and other features that unfold along the route [15,16].

The practice of forest therapy has risen in popularity since it was first formally recognized as health promotion activity in Japan and the Republic of Korea in the 1980s [17]. Parallel movements and traditions in other places worldwide have coalesced around a shared goal to advance forest therapy as a legitimate stand alone or complementary therapy to help address a range of physical and mental health issues [18]. The focus on health outcomes has led to a plethora of research studies in recent decades, with consistent results across diverse population segments, types of illnesses, and interventions showing positive benefits from participation in a forest therapy treatment [19,20,21,22].

The emphasis of research on health outcomes, however, has come at the expense of attention given to understanding the conditions that give rise to health-providing nature experiences. In particular, there is a relative dearth of research as to what key characteristics of forest therapy sites and trails optimize multi-sensory immersive experiences. This was a principal finding from an investigation of the Forest Therapy Trails research project that I initiated in 2019. In a comprehensive review of the forest therapy literature, our team examined 266 research articles published 1998–2021, coding them in part for how the forest landscape was characterized and the role, if any, it or its component features played in affecting health and wellbeing outcomes [23].

Our analysis found that only about half of the articles provided detailed descriptions of their study sites while less than 20 percent actually examined how site features or qualities influenced health and wellbeing outcomes. Our findings parallel those of several other research reviews [24,25,26,27]. For example, Paletto et al. [26] identified the relationship between forest stand characteristics and suitability of forest therapy sites as a principal knowledge gap in the research literature. Further, Clark et al. [27] recommended that future forest therapy research provide detailed reporting of forest types to understand the features that most effectively affect positive health and wellbeing outcomes.

To help fill this gap, our team conducted a follow-up study using in-depth interviews with forest therapy professionals to identify important concepts for refinement and explication through a second review of literature. This time we drew upon a much broader corpus of research articles, planning studies, management guidelines, and other literature across a wide range of disciplines and professions dealing with human-nature interactions [28]. Through our analysis we identified three site level criteria (landscape character and quality, tranquility, accessibility) and two trail level criteria (design and construction parameters, key trailside features and qualities), each with a number of sub-criteria, that together provided a comprehensive accounting of the important characteristics for use in evaluating the suitability of trails for forest therapy.

While our second study helps advance the evidence base for trail planning and design, the criteria we identified remain largely at the conceptual level. To make the concepts operational so that they can be applied to assess actual sites and trails, more work is needed. The objectives of the present investigation are to:

1. Develop a protocol to operationalize key concepts derived from previous investigations of the literature and interviews with forest therapy guides.
2. Apply the protocol criteria to a variety of sites and trails in two substantially different study areas and compare the results.
3. Provide details of protocol procedures with support documentation, worksheet templates, and examples to aid others in applying and adapting the protocol for use on other sites and trails.

As an effort in applied research, the goal of this next phase in our Forest Therapy Trails project was to close the gap between concept and application, extending the reach and value of evidence-based research and planning efforts into the development of guidelines and procedures for assessing the suitability of sites and trails for forest therapy. In so doing, the intended outcomes of this work are to support efforts of professional organizations that certify forest therapy trails, provide public land planners and managers with insights on how to develop new and manage existing trails to optimize forest therapy experiences, and add to the nascent forest therapy site and trail assessment literature.

## 2. Materials and Methods

### 2.1. Study Approach and Conceptual Framework

To address the study objectives, I employed an expert-based descriptive inventory approach to protocol development and application. Descriptive inventories are commonly used in landscape assessments where large amounts of information from diverse sources must be synthesized to make suitability evaluations [29,30]. While these evaluations rely on professional judgment, in this study the conceptual criteria derive directly from our forest therapy guide interviews and review of the research literature [28].

**Figure 1:**
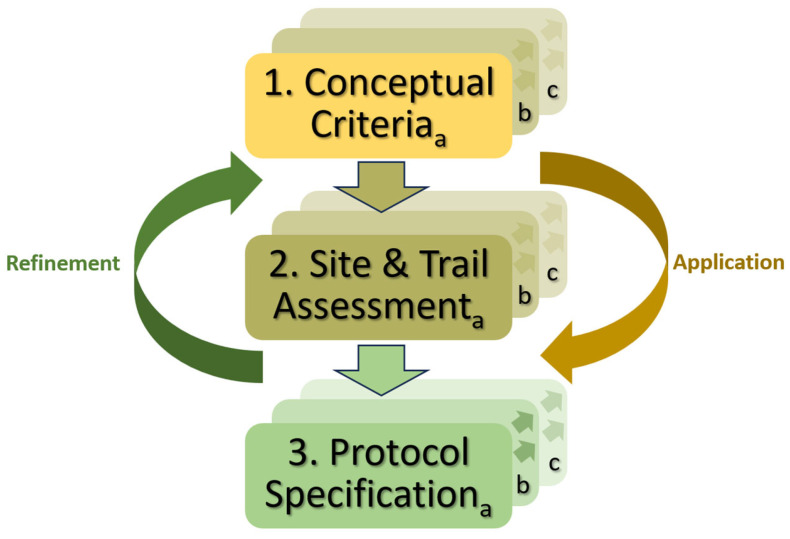
Illustration of steps (1–3) and rounds (a–c) used in the process for refinement and application of conceptual criteria, site and trail assessment procedures, and protocol specification.

This approach used a three-step, iterative process for specifying the protocol and applying it to assess the sample of sites and trails (Figure 1). With the initial conceptual criteria from our earlier study in mind (Step 1a), I began visiting a few test sites and trails within the two study areas to gain an understanding of how the concepts could best be assessed (Step 2a). In these initial visits I made field notes, took photographs, and experimented with different worksheets for specifying protocol criteria (Step 3a).

While field observation and measurement would comprise the core data for assessment of trail level criteria (e.g., key natural features), these visits suggested that evaluation of other criteria, especially at the site level, would be aided by external secondary data from various sources including site maps, brochures, and reports; historical and contemporary aerial imagery; and measurements from online mapping platforms.

**Figure 2:**
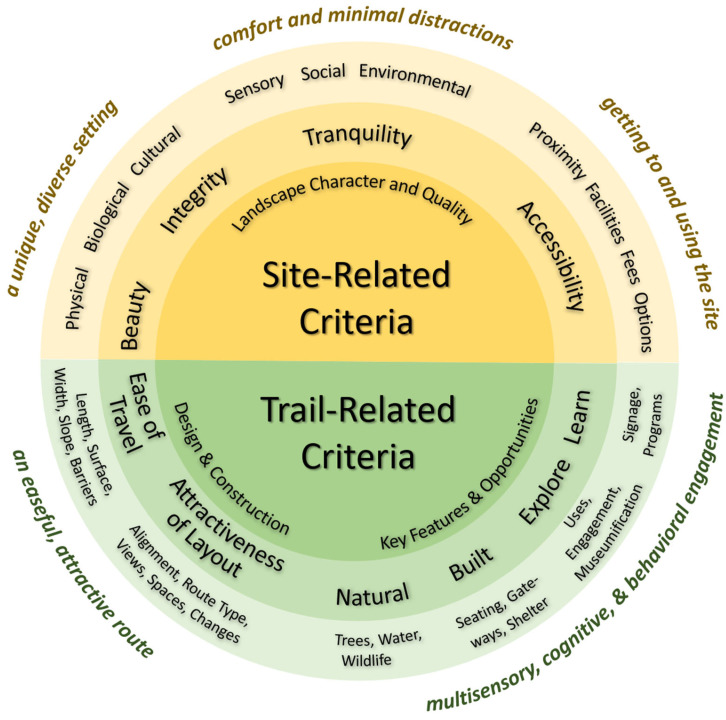
Revised conceptual structure of key characteristics of forest therapy sites and trails for use in the protocol and assessment.

The field visits helped to refine not only how the concepts generated from our earlier study could be measured in the protocol, but also how they could be better expressed and structured based on that original framework. This revised framework is shown in Figure 2 and identifies four site level criteria (beauty, integrity, tranquility, accessibility) and six trail level criteria (ease of travel, attractiveness of layout, natural features, built and borrowed features, explorable nature, interpretation and stewardship), each with several sub-criteria as shown in the figure.

In successive rounds of this iterative process (b–c in Figure 1), the protocol refinement suggested how to structure subsequent fieldwork, including the selection and organization of sites and trails for assessment. This expanded and diversified the numbers and types of trails and sites present within the study areas, which in turn helped to refine operational measures for the protocol to address study objective one.

### 2.2. Study Area Descriptions

**Figure 3:**
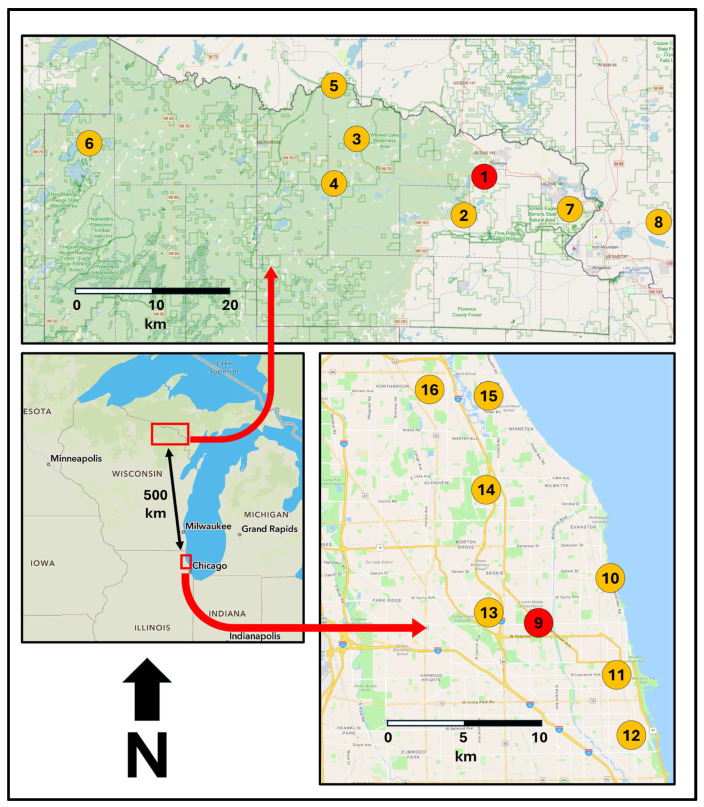
Regional locations (**bottom left**) of Northwoods (**top**) and Chicago (**bottom right**) study areas with numbers showing location of macrosite “places.” Numbers in red show location of origin points for proximity measures used for the site accessibility assessment.

Two study areas were chosen to assess how the protocol criteria functioned across diverse social-ecological conditions [31,32,33] (Figure 3). The Northwoods study area centers around Florence County in northeastern Wisconsin (45.922686 N, 88.260185 W, 2020 population 4300 (3.6 people/km^2^)), a rural, largely forested region with a high percentage of federal, state, and county public lands [34]. Glacial retreat 10,000 years ago left a varied topography of rolling moraines and outwash plains, with elevations ranging from 330 to 530 m [35]. There is an abundance of lakes, streams, and wetland resources, and the spine connecting many of the sites is the Pine River, a state designated Wild River [36]. Original forest cover was primarily hemlock-hardwood except for a portion along the eastern edge of the study area, which contains pine barrens and associated open and forested natural communities [37]. The area remains sparsely settled, with major economic activity oriented around forest industries and outdoor tourism. The numerous waterfalls along the Pine River have become a promotional focus for Florence County in recent decades [38].

The Chicago study area centers around northern Cook County in northeastern Illinois (41.987129 N, 87.724166 W, 2020 population 5.27 million (1200 people/km^2^)). It includes the north side of the City of Chicago and suburbs along the north shore of Lake Michigan and the North Branch of the Chicago River, the study area’s defining water features [39]. Glacial retreat 13,000 years ago left a nearly flat landscape, with elevations ranging from 176 to 207 m. Original vegetation included beach, dune, scrub oak, wetland, wet prairie, and dune communities on sandy soils close to the lake, while clay soils further west hosted wet and mesic prairies, wetlands, oak savannas, and oak woodlands [40]. While much of the area has long been transformed by urban development, efforts at greenspace protection beginning in the 1860s helped create systems of city parks and county forest preserves [41,42]. More recent activities have sought to enhance the natural values of these spaces through ecological restoration and management including creation of small natural areas within urban parks [43,44].

Although the Northwoods and Chicago study areas are separated by a distance of 500 km, the two have important functional connections. Historically, the growth of Chicago depended in large part on the Northwoods pineries for building material, and railroad development emanating from Chicago beginning in the 1850s fostered growth of small settlements across the Northwoods [45]. Today, Chicago remains a major market for Northwoods lake resorts and second home development [46].

### 2.3. Site and Trail Selection

Besides providing a diversity of social-ecological conditions to develop and apply the assessment protocol, the two study areas were also chosen for logistical reasons in conducting the extensive fieldwork that would be required. Initial site and trail visits began in 2020 at the outset of the COVID pandemic, limiting options for travel and involvement of others due to social distancing requirements. I used my home as the base for the Chicago study area and a family second home in Florence County for the Northwoods study area bases. While my site visits emanated from these two locations, in reporting data I use nearby nature centers that were part of the sample as they provide logical departure points for individuals and groups in accessing the sites and trails (see red numbers 1 and 9 in Figure 3).

Along with findings on site and trail level criteria from our earlier research [28], the initial visits within two study areas helped to refine and structure concepts for the protocol, and also clarified how sites and trails should be selected to serve as the sample for evaluation to address study objective two:

- Sites should be in primarily natural settings with trees, forests, or other natural communities [12,15]. This criterion was satisfied for most candidate sites in the Northwoods study area but played a greater role for Chicago sites, where designated natural areas within city parks and forest preserves were chosen over groomed green spaces, active recreation areas, or hardscape dominated settings;
- A site should have at least one and preferably more than one existing trail that could be used in its entire length or, in the case of long trails, as a logical segment for a forest therapy outing [47,48]. Sites were usually defined as having a single or connected network of trails accessible from one or more trailheads, while larger park and forest areas with multiple trail networks were divided into separate sites;
- Sites should be accessible within a reasonable proximity to represent logical choices one would make for a forest therapy outing [4,49]. In practice, I chose a ½ hour driving distance (under light traffic for the Chicago study area), and in the few cases where this was exceeded it was accounted for in evaluation of the site accessibility criterion (proximity measure).

Using these strategic criteria, I began expanding my initial sample, adding sites and trails that I was familiar with and exploring new ones within the proximity boundary. As the sample expanded, a structuring of sites emerged. Multiple sites naturally clustered around macrosites or “places” that suggested a common theme. For example, Pine-Popple Wild Rivers in the Northwoods study area included eight sites falling within a state-designated Wild Rivers project area. In the Chicago study area, Northside Neighborhood Natural Areas encompassed three City of Chicago sites featuring natural areas geared primarily toward neighborhood residents. In the first example, the name Pine-Popple Wild Rivers is an official designation while in the second the label was supplied by me, but both examples reflect ways in which forest therapy opportunities can be communicated to potential users as forms of placemaking and placekeeping, strengthening the ties between people and place [50,51].

Once identified, the places also suggested the inclusion of additional trails that connected individual sites or would otherwise add to visitors’ experience, understanding, and place appreciation. Specifically, these opportunities included bike trails—longer trails and roads within or between sites (“multi-sites”)—and paddle trails—lake and river routes that made use of the rich array of waterways in the two study areas. While forest therapy is usually thought of in terms of slow walks on forest trails, Bartlett Hackenmiller [52] describes how the concepts of forest therapy can be applied through a variety of activities.

This process of site and trail selection and place structuring continued iteratively along with protocol development and application until the sample adequately represented the range of conditions present in the study areas. This included eight places in each study area (Figure 3), with 29 sites and 91 trails in the Northwoods study area and 27 sites with 66 trails in the Chicago study area, for a total of 16 places, 56 sites, and 157 trails (see Table A1 for complete listing).

### 2.4. Criteria Inventory and Evaluation Procedures

While initial fieldwork began in 2020, the protocol was not finalized until 2022, with most visits occurring in 2023–2024 coincident with coding and evaluation. This extended sampling period allowed multiple visits to most trails to gain familiarity, and across the year to better understand how seasonality affected criteria such as leaf-on versus leaf-off visibility conditions (viewing distance, visual impacts), seasonal changes in vegetation such as spring flora and fall colors (natural features), summer suitability for physical access and use of water bodies (natural features), and winter road conditions (site accessibility) [53,54]. Overall, each trail was visited an average of 2.4 times in 1.9 seasons.

Given the potential differences between expert- and visitor-based landscape evaluations [55], I adapted commonly prescribed forest therapy techniques to better align fieldwork procedures to capture the essential elements of a forest bathing experience [12,16]. This included an initial stationary period of centering through breathing and multisensory awareness of the trail followed by slow, purposeful travel with active noticing and engagement with trail features and qualities. I approached fieldwork as an interactive, experiential activity, photographing site and trail features, measuring tree diameters and trail widths, and geolocating key features such as big trees and sitspots on a smartphone trail app as part of the trail experience rather than simply data collection. These and other participatory activities not only helped identify important characteristics of sites and trails but also clarified additional types of external information needed for evaluation [56].

In office work following a field visit, I mapped the trail route on Google Earth and if sites had no designated boundaries I drew a polygon around the immediate area of the site following natural or human-defined boundaries or transitions. I then compiled a fieldnote summary using the photographs, measures, and mapped information to recall important characteristics relating to the site and trail. Fieldnotes, photos, and related information from subsequent visits were added to the original set to build a portfolio of sites and trails. Once an initial structure of sites and trails became clear, I began coding field and secondary inventory data for site and trail criteria evaluation onto a spreadsheet. Using the iterative process described above (Section 2.1), the spreadsheet allowed me to add data for site criteria and sub-criteria, add trails and trail segments, and structure sites and places until I had a final set of inventory data and sample of sites and trails for evaluation.

Evaluation included qualitative and quantitative description of each of the criteria along with a simple three-point rating (low–moderate–high) [57]. Definitions, evaluation procedures, and data sources for the criteria are summarized in Table A2, Table A3 and Table A4 and detailed in the final protocol, with supporting literature cross-referenced here. Site level criteria (Table A2) relate to the character and quality of the landscape surrounding the trails and included beauty, integrity, tranquility, and accessibility. In addition, the protocol included a description of the site’s landscape character and history, summarizing its main geographical, jurisdictional, ecological, land use designation or other characteristics along with historical information influencing current patterns and features. This overview helped set the context for evaluating site level criteria but was not itself rated [58,59,60].

Beauty and integrity both pertain to the natural physical, biological and/or cultural-historic patterns and features of the landscape, with evaluation of beauty focusing on the variety, vividness, and uniqueness of these patterns and features [59,61,62,63,64,65] and integrity on their condition or intactness [59,66,67,68,69,70]. Their evaluation combines field data with several secondary data sources including maps, aerial imagery, and site-specific reports and websites. This data was synthesized in qualitative summary narratives highlighting the principal patterns and features contributing to the evaluation and rating of a site on each of the criteria. In contrast, the evaluation of tranquility [71,72,73,74] and accessibility [75,76] first entailed description of more discrete quantitative and qualitative sub-criteria. For tranquility this included setting [71,77,78], visual [79,80,81,82,83], sound [84,85,86,87,88,89,90], other sensory [91,92,93,94,95], social [96,97,98,99,100,101,102], and environmental [103,104,105,106,107] intrusions on the site’s peacefulness and isolation. Accessibility sub-criteria included proximity [4,108,109,110], trailhead facilities [111,112,113], user fees [114,115,116,117,118], and trail and accessibility options [47,48,77,119,120]. These sub-criteria were summarized using field and secondary data, and from this an overall narrative and rating was provided for each criterion.

Trail level criteria relating to trail design and construction include ease of travel and attractiveness of layout (Table A3). Trail design parameters of distance to trailhead [121,122,123], length [124,125], surface [126,127,128,129], width [120,121,127], slope [64,120,127,130], and accessibility barriers [120,130,131,132] together contribute to the ease of trail use. Trail design configuration parameters of alignment [71,133,134,135,136,137]; route type and directionality [126,135,136]; and views [29,59,138,139,140], spaces [141,142,143], and changes [144,145,146,147,148] contribute to an attractive, engaging layout. Most of these sub-criteria were assessed through field observation or measurement in combination with secondary data including topographic maps, aerial imagery, Google Earth, and site-specific information from websites and other sources. For trail length I also made use of the Avenza smartphone trail mapping application, which has been shown to have good horizontal positional accuracy [149]. However, trail slope information from both Avenza and Google Earth sometimes delivered exaggerated values and were cross-checked with topographic maps and adjusted where necessary. Information on these sub-criteria was listed or summarized then rated for each criterion.

Lastly, trail level criteria relating to key trailside features and opportunities include natural features, built and borrowed features, explorable nature, and interpretation and stewardship (Table A4). Dominant vegetation cover type [150,151,152,153] was described to set the context for natural feature sub-criteria relating to trees [154,155,156,157,158,159,160], water [161,162,163,164,165,166,167,168], wildlife [169,170,171,172,173], and other distinctive features and related sensory effects [174,175,176,177,178]. Built and borrowed features related to human-built, naturally occurring, or human-adapted features that serve utilitarian or other functions [179]. Sub-criteria included seating, gateways, shelter, and other features [180,181,182,183,184,185,186]. Explorable nature related to policies and design features that facilitate or restrict interaction with natural trailside features. Sub-criteria included permitted or restricted use policies [187,188,189,190,191,192], signage or barriers that physically restrict or symbolically discourage use (museumification) [193,194], and other design and management aspects of the trail and corridor such as trail width and right-of-way vegetation management that facilitate or hinder engagement with natural features (on-trail engagement) [71,193,194]. Interpretation and stewardship included two sub-criteria related to onsite signage and on- and off-site interpretive programs [195,196,197] and volunteer stewardship opportunities aimed at environmental learning and involvement in restorative site and trail management activities [198,199,200]. All of these sub-criteria were mainly assessed through field on-trail observation, with information on policies, programs, and volunteer opportunities available online or at trailhead kiosks. Data related to these sub-criteria were listed or summarized then rated for each criterion.

### 2.5. Analysis and Documentation of Procedures

To address objective two regarding the comparison of criteria for sites and trails, rating and descriptive data were analyzed in the following way. For the rating data, the three-point scale was considered ordinal and data were not assumed to be normally distributed, steering analyses toward non-parametric statistics. The reliability of the rating procedure was assessed by re-rating site and trail criteria three months after the original ratings were made and comparing the two sets of ratings using Cohen’s linear weighted kappa [201]. Kappa values over all criteria averaged 0.783 and ranged from a low of 0.647 for natural features to a high of 0.886 for interpretation and stewardship. In all cases these values indicated good to excellent reliability and the original ratings were used for all subsequent analyses. For comparative purposes among individual sites and trails, ratings were summed across criteria to produce a total score for each site (value range 4–12 for four criteria) and a separate total score for each trail (value range 6–18 for six criteria). To understand relationships between criteria, the ratings for the four site criteria were correlated with each other using the Kendall’s tau statistic (*n* = 56 sites), as were the ratings for the six trail criteria (*n* = 157 trails) [202]. To understand differences between sites and between trails, the ratings for site and trail level criteria were compared between study areas (Northwoods versus Chicago) and between trail types (foot, paddle, bike) using the Kruskal–Wallis H statistic [203]. All statistical procedures were executed in SPSS Version 30.

For the descriptive data, sites and trails were sorted by their rating on a given criterion and descriptions were qualitatively compared to understand how they differed across high and low ratings. This procedure was used to provide summary narratives with selected examples to illustrate how a given criterion contributes toward making a good site or trail for forest therapy.

To address objective three regarding the documentation of protocol procedures and their application, a number of Supplementary Documents were prepared as part of project reporting. A detailed protocol document was prepared that included definitions, evaluation procedures, explanatory notes, and data sources for each criterion and sub-criterion, along with Supporting Information that included representative quotes from interviews with forest therapy guides and summary/highlights of results and key citations from the research, planning, and design literature. This Supporting Information expands upon work from our earlier study [28] and integrates it with the protocol. To foster application of the protocol beyond this study, a worksheet template was prepared with abbreviated instructions for describing and rating site and trail criteria along with two completed worksheet examples. A rapid assessment screening worksheet was also prepared for those wishing to conduct initial field ratings prior to detailed analysis. An additional Supplementary Document provides illustrated examples of trail level features and opportunities for selected criteria and sub-criteria.

## 3. Results

### 3.1. Criteria Ratings

Criteria ratings for individual sites and trails are shown in Supplementary Tables S1–S4. Total site scores ranged from a low of 5 for one site to a perfect score of 12 for three sites, with a mean total score of 9.9 (*SD* 1.37). Mean total scores were not significantly different between the Northwoods and Chicago study areas. Total trail scores ranged from a low of 8 for four trails to a perfect score of 18 for three trails, with a mean score of 13.1 (*SD* 2.05). Chicago area trails scored slightly higher than Northwoods trails (*M* 13.5 vs. 12.7, *F* (1, 155) = 6.237, *p* = 0.014), while there were no significant differences in total scores between foot, paddle, and bike trails.

Intercorrelations between site and between trail criteria are shown in Table 1. For site criteria, integrity had a significant but weak positive correlation with tranquility, perhaps reflecting a lack of environmental site disturbance, and a moderate positive correlation with beauty, as both criteria focus in part on diversity of vegetation. The only other significant correlation was between tranquility and accessibility, which were weakly and negatively associated. Here, closer proximity, higher trailhead facilities, and more trail options may relate to higher noise levels from access roads and site users disturbing the peacefulness of the site.

Several trail level criteria were significantly correlated, though all were under a 0.500 value considered as a lower end of a moderate correlation. The highest correlations showed layout attractiveness associated with natural features and with explorable nature, perhaps reflecting trail layouts that capitalized on natural, engaging views and changes along the route, and between built features and interpretation and stewardship, as both criteria deal in part with visitor support facilities such as signage and nature centers.

The mean ranks of site and trail criteria ratings between study areas and trail types are shown in Table 2; complete frequencies, percentages, mean scores and standard deviations are available in Supplementary Tables S5–S9. As might be expected in comparing rural versus urban conditions, Northwoods sites were significantly higher in tranquility while Chicago sites were significantly higher in accessibility. There were no significant differences between study areas in beauty or integrity. Chicago trails tended to be better developed for a wide range of visitors, with significantly higher ratings for ease of travel, built and borrowed features, and interpretation and stewardship. Northwoods trails tended to have fewer use restrictions, rating significantly higher for explorable nature. There was no significant difference between study areas on natural feature ratings and attractiveness of layout was only slightly higher for Northwoods trails. With respect to trail types, paddle trails had significantly higher ratings on explorable nature and foot trails were rated higher on interpretation and stewardship, while other criteria ratings showed no or only slightly significant differences between trail types.

### 3.2. What Makes a Good Site for Forest Therapy?

#### 3.2.1. Beauty

Sites rated high in beauty tended to have a combination of features high in variety, vividness, or uniqueness, including varied topography, unique landform or vegetation features, prominent or vivid water features, a number of different land cover types, and/or prominent cultural-historic features. Many sites (70%) rated high in beauty in both the Northwoods and Chicago study areas, with no significant difference between mean ranks. One highly rated Chicago study area example was the Alfred Caldwell Lily Pool (Site 12.1). A small, historic designed landscape, it features a variety of native trees, ground flora, and wetland vegetation surrounding a central pond bordered by unique rockwork and a naturalistic fountain emulating a Midwestern prairie river. Sites with moderate beauty ratings tended to be more homogeneous, with level topography, uniform vegetation cover, and were lacking in water or distinctive landform, cultural, or vegetation features. A moderately rated Northwoods study area example was Halls Creek Trails (Site 2.3). Its varied topography is notable but its relatively uniform forest cover, large areas of dense young aspen, and lack of visible water along the trails reduced its rating. Some relatively homogeneous sites, however, received high beauty ratings due to exceptional single features such as unique rock outcrops or landscape types, prominent lake and river views, extensive old growth forests, or diverse understory vegetation.

#### 3.2.2. Integrity

Sites high in integrity showed a high degree of condition or intactness in landscape patterns and features contributing to its ecological, recreational, and/or cultural-historic quality. Integrity ratings were nearly split between high (46%) and moderate (48%) ratings, again with no significance between Northwoods and Chicago sites in mean ranks. Somme Preserves (Sites 16.1–16.3) are good examples of high ecological integrity Chicago sites. They benefited from early preservation efforts which spared old growth oak woodlands and prairies from being cut or plowed for agriculture, and from more recent ecological restoration efforts to remove invasive species and restore native understory plant diversity. While ecological elements played the principal role in determining integrity for most sites, for some sites the condition and quality of cultural-historic and recreational elements factored into ratings. The Hidden Lakes Trail (Site 6.1) in the Northwoods Study Area is a good example. This popular National Forest Recreation Area is protected under several designations and management objectives to ensure high integrity land, water, and cultural resources are maintained. The site is managed under a high Scenic Integrity Objective that limits timber harvest to maintain and enhance mature and old growth character, State Natural and Pine Marten Protection Areas to protect rare ecological communities and species, a local no-motor boating ordinance on the area’s many small lakes to protect shore and water quality, and historic preservation designations to preserve nationally significant Native American archeological and Civilian Conservation Corps historic sites.

#### 3.2.3. Tranquility

High tranquility sites had characteristics that shielded them from social and sensory intrusions, and here Northwoods sites were much more likely than Chicago sites to be rated high in tranquility (66% vs. 15%). Northwoods sites tended to be larger in size, lower in other recreational and adjacent uses, and more distant from major highways and development. One exception was the Wild Rivers Interpretive Center (Site 1.1), the central departure point for Northwoods study area sites. While being high in accessibility (see next section), it rated low in tranquility due to its small size, visible residential and commercial development, and close proximity to two highways (Average Annual Daily Traffic (AADT) 3800 and 1800). Road noise tended to be the major intrusion into the tranquility of sites in both study areas, and even relatively low use highways contributed to moderate tranquility ratings for Northwoods sites. For example, at Fox Maple Woods (Site 3.1), individual vehicles passing along an adjacent State Highway (AADT 850) interrupted the silence every few minutes. While traffic noise was nearly ubiquitous for Chicago sites, high traffic levels on roads further away from sites tended to create a constant background din that could more easily be ignored. This was the case at Montrose Point located in Lincoln Park along the city’s north lakefront (Site 11.1), the only city site rated high in tranquility. While this portion of the park was built on landfill in part to accommodate what has become one of the city’s busiest thoroughfares (AADT 82,000), the site’s 1.0–1.4 km distance from DuSable Lake Shore Drive effectively reduces road noise, although it is still vulnerable to occasional disturbances from an adjacent beach use, summer motorboats, and overhead planes on the runaway approach to busy O’Hare airport 20 km to the west.

#### 3.2.4. Accessibility

Chicago sites were much more likely to be rated high in accessibility compared to Northwoods sites (85% vs. 35%). Chicago sites had better all-season roads leading to the trailhead, a higher level of trailhead facility development, and a wider range of trail options that included several trails that were wheelchair accessible. On other sub-criteria there were few differences between study areas. Chicago sites were closer in distance compared to Northwoods sites but the average drive time was about the same, and several Chicago sites had hourly parking fees while several Northwoods sites had day use fees. Facility development was a major sub-criterion in evaluating accessibility, and sites without at least a portable toilet were rated low while more highly accessible sites included toilets and other facilities. The two most developed sites were the central departure points for Northwoods and Chicago study areas (Sites 1.1, 9.1), both of which had nature centers with indoor restrooms, meeting spaces, and interpretive displays along with outdoor shelters and picnic areas. Sites with paddle trails tended to have low or modest facility development, though one site in each study area offered watercraft rentals, greatly increasing accessibility for this type of forest therapy experience (Sites 1.7, 15.1).

### 3.3. What Makes a Good Trail for Forest Therapy?

#### 3.3.1. Ease of Travel

Ease of travel was one of two trail design and construction criteria, and sub-criteria associated with high ratings included close trailhead access, a non-fatiguing trail length of 1–2 km, a comfortable trail width of 0.5–3.0 m that varied by use level and type, a gentle average slope, a stable surface tread of natural or naturalistic materials, and few physical accessibility barriers. Foot trails made up the bulk of the trail sample (*n* = 116 (74%) of 157 trails) and for these, Chicago trails were more than twice as likely as Northwoods trails to be rated high in ease of travel (77% vs. 26%). Overall, Chicago foot trails had shorter lengths (*M* = 1.3 vs. 2.4 km), gentler average (*M =* 2% vs. 5%) and maximum (*M =* 19% vs. 23%) slopes, and were much more likely than Northwoods trails to have constructed surface treads of concrete, asphalt, stone, crushed gravel, or woodchips (50% vs. 2%). Within the Chicago study area there were also clear differences between city park and suburban forest preserve trails, with city trails tending to be shorter and wider, and many had hard surfaces and barrier-free access for visitors with mobility constraints (e.g., Trails 9.21, 11.11). Length was often the major sub-criterion in evaluating trails on ease of travel. While the Northwoods study area included some shorter, more developed, and accessible nature-interpretive trails (e.g., Trails 1.11, 4.22, 6.11, 6.31), many Northwoods trails were designed for longer hiking or cross-country skiing. In some cases this necessitated splitting trails into shorter segments to include them as viable candidates in the study sample (e.g., Trails 3.41, 8.14). Additionally, nearly all Northwoods trails had lightly managed dirt or grassy surface treads compared to Chicago trails (95% vs. 37%), and many had minor accessibility barriers such as exposed rocks and roots in the trail tread and brushy rights-of-way.

While generally following the same principles, paddle and bike trails had somewhat different standards for what qualified as highly rated for ease of travel. Highly rated paddle trails included flatwater lakes and streams with shorelines 1–2 km (loop or there and back) that facilitated travel ease with minimal fatigue (e.g., Trails 6.21, 15.16), while rivers with current on gentle gradients could be 2–5 km (e.g., Trail 1.71). Smaller lakes and streams with sufficient depth and sandy or other firm substrates (e.g., Trail 7.41) were easier for travel than large water bodies and muck bottoms (e.g., Trails 1.82, 11.41), which could make navigation in windy/wavy conditions and ingress/egress difficult. Easy trailhead access was important for paddle trails, and long or difficult portages hampered otherwise easy travel (e.g., Trail 3.23). Highly rated bike trails had lengths between 5 and 11 km, with shorter routes better for steeper terrain and rougher trail surfaces. For the Chicago study area, low traffic paved and gravel park trails 2.5–4 m in width offered room for shared use with minimal conflict (e.g., Trail 11.42), while narrower shared trails and sidewalks or wide streets with numerous vehicles were problematic (e.g., Trail 9.42). For the Northwoods study area, dirt forest roads offered the best ease of travel as long as they were not too wide to encourage higher speed use by motor vehicles (e.g., Trail 1.85), while narrow mountain bike trails were difficult to enjoy in a relaxing way due to steep grades and trailbed obstacles (Trail 4.13).

#### 3.3.2. Attractiveness of Layout

Attractiveness of layout was the second criterion relating to trail design and construction and sub-criteria included trail alignment, route type and directionality, and views, spaces, and changes. Foot trail layouts rating high in attractiveness had curvy to winding looped routes with a variety of view types, private and group spaces, and changes. Northwoods foot trails rated higher in layout attractiveness than Chicago trails, in part because their larger average site size, more varied topography, and longer average trail lengths offered more opportunities for different views and changes along the route. One highly rated example was the 3.5 km North Loop of the Rainbow Hunter Walking Trail network in the Chequamegon-Nicolet National Forest (Trail 3.51). While located in a predominantly interior maple-aspen forest, its moderately hilly terrain (30 m elevation change) and winding alignment traversed several changes in forest stand types and age classes, and occasional openings offered private sitspots and group spaces with interior mature growth forest, distant hilltop, and eye-level river and waterfall views. Some longer Chicago area trails such as the Prairie-Grove Loop at Somme Prairie Grove (Trail 16.22) also had highly attractive layouts due to naturally occurring variations in the landscape. For some smaller sites and trails, such as the Japanese Garden at the Chicago Botanic Garden (Trail 15.24), high attractiveness was accomplished through carefully constructed changes in landcover types, added water features, and purposeful view sequences.

Highly rated paddle trails followed natural shorelines with high complexity and different upland and wetland cover types. Examples included the Pine River Oxbow and Sea Lion Lake Islands and Bays in the Northwoods study area (Trails 1.71, 2.14) and the Skokie Lagoons trails in Chicago (Trails 15.15–15.17). These routes had close-up views affording changes in shoreline vegetation, distinctive trees, and abundant wildlife that unfolded in a near-continuous sequence of delight. Less attractive routes followed simpler shorelines, such as paddle trips along the shores of Little Fumee Lake in the Northwoods (Trail 8.11) and Chicago’s Lake Michigan (Trails 10.32, 11.141) While these trails offered distant, panoramic lake views, the shores themselves held little interest. This was magnified for the Lake Michigan paddle trails, which restrict boats from going within a 100 m zone during beach season to avoid conflicts with swimmers. For bike trails, highly rated routes were mostly on designed bike trails within Chicago parks and forest preserves (e.g., Trails 11.42, 12.51). These routes followed curved trail alignments and provided changing scenery while also linking together and providing access to foot trails. Less successful routes followed straight roads and converted rail corridors, which while serving as linkages between sites and foot trails, sometimes offered little in the way of distinct views or changes beyond the immediate corridor (e.g., Trails 1.84, 9.41). This unfortunately was the case with most of the bike routes in the Northwoods Study area, most of which were rated moderate or low in layout attractiveness.

#### 3.3.3. Natural Features

Key natural features included trees, water, wildlife, and other vegetation, landform, and natural objects and related multisensory effects. Trails rated high on this criterion had one or more outstanding natural features such as many large diameter trees or old growth forest stands; water bodies that were prominent in size, visually accessible at close distance and for long duration, or physically accessible for partial or full body immersion; prominent wildlife habitat, nesting, and observation opportunities; and other key natural features such as distinctive groundcover flora, rock, moss, or fungi. There was no significant difference in natural feature mean ranks for foot trails between study areas, though from a qualitative perspective there were distinct differences between Northwoods and Chicago trails in the types of features contributing to high natural feature ratings. With respect to trees, while both study areas had trails where big trees and old growth forest conditions were highlights (e.g., Northwoods Trails 1.11, 3.13, 4.25; Chicago Trails 9.24, 16.21, 16.31), the closed hemlock-hardwood Northwoods forests and open oak Chicago savannas provided distinctly different landscape experiences and seasonal effects. Both study areas had several trails with prominent water features, but except for the routes along the shore of Lake Michigan (Trails 10.21–10.31), waters in the Chicago study area were not desirable for physical contact while many lakes and rivers in the Northwoods were. Northwoods trails had distinctive rocks, rock outcrops, and glacial landform features such as eskers (e.g., Trails 1.31, 2.13, 4.23, 6.13), while Chicago trails traversed much more subtle terrain. Finally, the open Northwoods barrens (e.g., Trails 7.11, 7.21, 7.31) and Chicago prairies (e.g., Trails 11.13, 13.21, 16.11) both had highly diverse groundcovers of grasses and forbs, though their species compositions, experiential, and ephemeral qualities were each unique.

Due to their prominent water features, most paddle trails rated highly on the natural features criterion, but the water routes also provided outstanding opportunities to experience the dynamic, multisensory qualities of the water body; observe deer, birds, and aquatic wildlife; and view large, distinctive trees and other vegetation along the shore. Fewer bike trails rated highly in natural features, in part because wider trail widths and routing increased viewing distance from water bodies and trees, while the greater speed of travel reduced wildlife viewing opportunities. On the positive side, greater speed increased awareness and appreciation of landform changes along the route, with dips and curves adding to the experience.

#### 3.3.4. Built and Borrowed Features

Built and borrowed features included seating, gateways, shelters, and other human-built, naturally occurring, or human-adapted natural features serving utilitarian, aesthetic, or symbolic functions. While Chicago foot trails ranked significantly higher on this criterion than Northwoods trails, the percent of trails that were rated high (19% overall, 8% Northwoods vs. 35% Chicago) was lower than most other trail level criteria. Highly rated trails tended to be located at nature centers (e.g., Trails 1.11, 6.11, 9.11, 12.21), in city parks (e.g., Trails 9.31, 11.42), or in institutional settings (e.g., Trails 10.11, 15.25) where visitor comfort and amenities are more expected. Built features at Northwoods and Chicago forest preserve trails were noticeably more low-key and rustic in character than they were at Chicago city nature centers and park natural areas. The former more often adapted natural materials for features such as rough-hewn logs for benches (e.g., Trails 4.22, 14.11), stream crossings, or boardwalks (e.g., Trails 6.32, 14.14) or simply borrowed unmodified logs and rocks to serve mainly utilitarian functions (e.g., Trails 3.41, 16.33). City trails more often included durable and functional finished wood or metal benches (e.g., Trails 9.31, 10.21), plastic boardwalks and observation decks (e.g., Trails 11.31, 12.41), gazebos or picnic shelters (e.g., Trails 11.31, 12.41), and sometimes included sculptures (e.g., Trails 9.21, 12.21), stone council rings (e.g., Trails 12.11, 15.21), and other features with overt symbolic and aesthetic attributes.

By their nature, paddle trails in both the Northwoods and Chicago study areas were less developed with few built features beyond the trailhead boat landings, and even those tended to be primitive in nature unless they also catered to fishing or motor boats. Chicago bike trails rated higher than Northwoods trails, mainly because they traversed parks and forest preserves that served users and activities beyond the trail, while most Northwoods bike trails followed forest road corridors.

#### 3.3.5. Explorable Nature

Explorable nature included use policies and design features that facilitated or restricted exploratory on- and off-trail activities. For this criterion, Northwoods foot trails had a significantly higher mean rank than Chicago trails, with most trails, even within designated State Natural Areas, permitting off-trail use for activities such as foraging, hunting, and nature study. No Northwoods trails had physical or symbolic “museumification” barriers such as fencing or ropes along the trail that discouraged use, except for one site where trails went near an old mine shaft (Trails 8.21–8.24). In contrast, most Chicago trails had policies either posted at trailhead kiosks or stated in online site information that visitors should stay on-trail and refrain from harvesting any plant materials. However, fishing was permitted from paddle trails, and one foot trail allowed fishing (Trail 9.22) while another had a casting pier (Trail 12.31). Rope barriers lined several trails in city park natural areas (e.g., Trails 10.23, 11.12), and more formal fencing and other symbolic museumification visual cues such as curbing or low walls were employed on trails at some of the nature center and institutional sites (e.g., Trails 10.11, 12.41, 15.24).

For the Chicago study area, moderate exploratory activity was possible along the narrower trails, especially in prairie, savanna, and open woodland areas (e.g., Trails 15.25, 16.23). Narrow trails in these environments allowed groundcover vegetation to reach out from the right of way into the trailbed and enabled visitors to feel, smell, and otherwise interact with the vegetation, especially during peak summer growth. Wider trails lessened such interaction, including most bike trails in both Chicago and Northwoods study areas. In contrast, paddle trails rated high in explorable nature in both study areas, where navigating the rivers and lakes was a highly interactive experience.

#### 3.3.6. Interpretation and Stewardship

Interpretation and stewardship sub-criteria included onsite signage, on- and off-site programs, and other opportunities aimed at enhancing nature experience, learning, and involvement in environmental improvement efforts. Interpretation opportunities pertained almost exclusively to foot trails, with highly rated trails in both study areas having interpretive signage and other opportunities including programs and activities (e.g., Trails 6.31, 9.21), demonstration gardens (e.g., Trails 9.13, 11.11), children’s nature play areas (e.g., Trails 9.13, 9.21), and nature centers offering printed information and exhibits (e.g., Trails 1.11, 9.13). Moderately rated trails had fewer such opportunities. Chicago trails had a significantly higher mean rank than Northwoods trails, due largely to active volunteer ecological restoration stewardship groups that offered regular workdays. These were usually mentioned on signs posted onsite with links to online signups (e.g., Trails 12.31, 13.11), with some websites providing additional learning resources (e.g., Trails 16.21–16.24). Paddle and bike trails were not oriented toward interpretation and stewardship and thus rated low on this criterion.

### 3.4. Protocol, Worksheets, and Examples

To address study objective three, a set of documents was produced for inclusion as Supplementary Materials to this paper. These materials include a detailed protocol of procedures and supporting documentation for operationalizing site and trail level criteria (Document S1), a worksheet template with brief instructions for applying the protocol to new sites and trails (Document S2), a rapid assessment worksheet for screening trails prior to detailed assessment (Document S3), completed worksheet examples for sites and trail networks in the Northwoods (Site 4.2, Document S4) and Chicago (Site 11.1, Document S5) study areas, and illustrated examples of trail features and opportunities relating to selected criteria and sub-criteria (Document S6).

## 4. Discussion

### 4.1. Criteria Assessment and Management Implications

Building on conceptual criteria from previous research that included interviews with forest therapy guides and a comprehensive literature review [28], this investigation operationalized and applied a protocol for evaluating the suitability of sites and trails for forest therapy engagements. Through an expert-based, iterative approach, I refined measures for evaluating four site (beauty, integrity, tranquility, and accessibility) and six trail (ease of travel, attractiveness of layout, natural features, built and borrowed features, explorable nature, and interpretation and stewardship) level criteria. I then applied them to 56 sites and 157 associated trails in two social-ecologically diverse study areas in the rural Northwoods of Wisconsin and urban-suburban Chicago. The criteria ratings showed good to excellent test–retest reliability [201] and helped distinguish differences within and between study areas and trail types [204]. Qualitative descriptions provided depth of understanding and insight into how the criteria helped determine what makes a good site and trail for forest therapy [30,205].

Many of the sites rated high in beauty and site descriptions detailed how beauty can be expressed at the site level in a variety of ways though different combinations of diverse, prominent, or unique landform, vegetation, water, and cultural landscape features and patterns. From closed old growth forests on hilly terrain to flat open prairies, one lesson from the field studies is that evaluation of this criterion is not simply a matter of finding the most beautiful sites but rather looking at what makes each site beautiful in its own right [206,207]. While evaluations of integrity examine similar landscape features and patterns, the focus on condition and intactness demands a strategy that is more multidimensional in nature [208], including investigations of management histories using secondary data of a site’s ecological, recreational, and/or cultural integrity to supplement field observation. This is because some aspects of integrity, especially ecological integrity, may not be easily perceived on the ground, which can lead to a disjunct between beauty and integrity [209]. In this study moderate positive correlations indicated frequent correspondence between beauty and integrity ratings for sites, and well-researched and written site descriptions can help site managers in communicating an ecological or cultural landscape aesthetic to visitors. This is especially important when sites undergo restoration and management such as prescribed burning or tree removals to improve integrity, which could be seen by visitors as temporarily detracting from visual beauty [207].

Tranquility as operationalized in this study looked at social and sensory intrusions disrupting a site’s peacefulness. Of these intrusions, noise was the major factor [89], especially in the Chicago study area, which tended to have smaller sites and busier roads in closer proximity. Even on sites that were large and somewhat remote from roads, overhead planes could pose major disturbances during peak landing hours at busy O’Hare airport, which was on the direct flight line from several of the Chicago sites. A number of the criteria in this study had temporal and/or spatial dynamics that affected their evaluation, and in the case of tranquility, sound intrusions are somewhat predictable and repeated site visits and secondary data can help identify optimal times for scheduling forest therapy visits [210]. Accessibility and tranquility were somewhat negatively correlated, and qualitative comparisons revealed that sites tended to group in terms of those that were highly accessible but rated lower on the other criteria, and those that rated high for beauty, integrity and tranquility but lower on accessibility. This split was mainly a function of study area, with Chicago sites rated significantly higher in accessibility due to better road access and trailhead facilities, but one insight from the analysis is that successful forest therapy sites can be found in a variety of settings and it is important to identify and work with each site’s individual strengths and limitations [211].

For the trail level criteria, ease of travel and attractiveness of layout both dealt with design and construction parameters. While this label implies a type of control over length, width, alignment, and other trail sub-criteria, most of these are already decided when dealing with existing trails. Many of the trails in this study, especially in the Northwoods study area, evolved from deer trails, Native American travel routes, 19th century logging roads and railways, or in the case of paddle trails, from nature itself. Other trails were designed for different recreational activities that may not provide optimal conditions for forest therapy. For locations where existing foot trail options are limited, interpretive trails and shorter trails or trail segments can better accommodate forest bathers with average skills and comfort levels than longer hiking or shared use trails, while off-street routes and low-use forest roads can make suitable bike trails [124,125]. While length is a primary consideration for all trail types, paddle trails on flatwater lakes and waterways or streams and rivers with gentle gradients are better suited to facilitate slow, exploratory travel experiences. Trail managers can enhance the attractiveness of existing trail layouts by managing vegetation to create or maintain views and provide openings for private sitspots and group activity spaces. Short spur trails can also be added to take advantage of different vegetation types and forest age structures that provide a sequence of changes along the route [12,15]. These relatively simple management changes can benefit not only forest therapy visitors but all trail users [120].

Trees, water, wildlife, and other natural features encountered along the trail are in many ways the highlights that enrich forest therapy experiences, especially for self-guided visits where trail users do not have the benefit of guides who can curate experiences through directed activities or invitations [212]. While results highlighted how the two study areas differed in the types of natural features that contributed to engaging experiences, these differences were also seen within study areas at the macrosite level. Understanding the natural features that make places special can help managers better communicate the natural values of opportunities to visitors and aid forest therapy guides to tailor their programs to incorporate the natural assets present [213]. Seating, gateways, shelter, and other built and borrowed features can also enrich forest therapy experiences, but their presence and level of development depend highly on the nature of the trail setting [214]. Results showed lower ratings of this criterion in Northwoods and Chicago forest preserve settings, and while fewer built features are generally more appropriate in these settings, many trails could benefit from at least some built features to facilitate use. The key here is to introduce built features that fit visually and experientially with the natural context in terms of material selection, rusticity, number, and placement. Principles of contextual compatibility and biophilic design can aid in decision-making [182,183,184,185,186,214], and examples of built features in the Supplemental Materials (Document S6) illustrate the range of options found in the study areas.

Written policies and trail design elements can facilitate or restrict the degree to which visitors can engage with natural features and off the trail, and study results for explorable nature found major differences between the two study areas. While more restrictive use policies and higher levels of museumification are often warranted in sensitive areas that receive high use, blanket deterrence can unnecessarily restrict important forest therapy opportunities that are benign when conducted with respect for nature [193]. In the same way that some parks in this study provided nature play areas for children, managers could consider allowing low-impact off-trail exploration and interaction with natural features on some trails in less environmentally sensitive areas, either as part of a self-guided forest therapy walk or as supervised by a forest therapy guide [193]. Enhancing opportunities for explorable nature can go hand in hand with interpretation and stewardship activities, where signage can inform and direct nature engagement and stewardship programs can facilitate environmental learning and experiential appreciation while also building reciprocal relationships that strengthen the health and wellbeing of people and nature [215].

### 4.2. Protocol Application Limitations and Adaptations

Being grounded in evidence-based research and planning efforts, the conceptual criteria used in the protocol should have good generalizability for further application [204], though procedures and values assigned for evaluating them may require adaptation depending on regional and cultural variability, purpose of the assessment, and skills of the evaluators [30,204]. For example, what constitutes a big tree, steep gradient, low and high visitor use or noise levels, or other sub-criteria can vary widely by region and culture and thus measures should be adapted accordingly. This was evident in evaluating the tranquility criterion in this study, where ambient noise from road traffic at Chicago sites formed a consistent din that did not exist at Northwoods ones, requiring a somewhat different frame for evaluation for the two study areas. While the two study areas were chosen precisely to identify how criteria and their measures functioned across diverse settings, in actual planning and management applications it would be better to focus on sites and trails within a relatively homogeneous region if comparisons between sites and trails are an important purpose of the assessment.

This study purposefully included a large number of sites and trails to test how criteria measures and procedures functioned across a diverse sample of conditions. In early team discussions about protocol application there was some hesitancy about “overquantifying” the assessment, as conceptually the criteria are qualitative in nature and operationally synthesize the qualitative and quantitative description of sites and trails. In facing the large amount of accumulated data, however, I decided to employ a rating system to facilitate comparisons between sites and trails, and more generally between the two study areas and foot, paddle, and bike trail types. The high–moderate–low rating was chosen to keep quantification simple yet still allow for some basic statistical comparisons [56].

As the sole evaluator applying this expert-based approach, my rating reliability was quite high and attributable in part to four decades of experience in research and application of landscape assessment methods. Intra-rater reliability, however, is a limited assessment of measurement reliability and a logical next step would be to conduct a test of inter-rater reliability using a small panel of experts on a selected subset of sites and trails [216].

Beyond reliability testing, I encourage others to apply and adapt the protocol to strengthen confidence in the assessment procedures. In further applications and extensions of this protocol, while a rigorous approach should be followed that draws on available data as suggested in the protocol procedures, ratings could be considered optional and reserved for situations where comparisons between sites and/or trails are deemed useful. For example, planners and researchers undertaking comprehensive assessments could build on the protocol by adopting more sophisticated measurement and rating procedures, such as those employed in recent similar investigations [217,218,219,220,221,222]. In other cases and for more limited applications, for example, for forest therapy guides who wish to evaluate potential trails for use in walks, a simpler rating and descriptive analysis might suffice, using the screening worksheet for rapid field assessment (Supplementary Document S3).

While the conceptual criteria underlying protocol measures were derived from our earlier work with forest therapy guides and a comprehensive review of the literature [28] and thus provide a solid evidence base for their use, further work is needed to help support the validity of the protocol. Future research field testing the approach across a variety of sites and trails with forest therapy visitors, alone or with forest therapy guides, would help determine how site and trail assessment criteria measures relate to preferences and other outcomes. Isolating individual site and trail characteristics and their contribution to the overall experience could be aided by post-visit surveys or guide/researcher participant observation [48,64]. Laboratory approaches such as discrete choice experiments, while abstracting the multisensory characteristics of in-field experiences, provide attractive opportunities for isolating protocol criteria and studying their importance and interaction effects as well as individual and interest group differences [223].

## 5. Conclusions

Forest therapy is a growing international practice with roots not only grounded in different cultures but also originating in widely different landscapes around the world. While much has been accomplished in recent years to expand this practice globally through professional organizations and to quantify its health benefits through research, the development of tools for the identification, design, and management of sites and trails for forest therapy is lacking. The Forest Therapy Trails project was undertaken to help fill this gap, and building on a conceptual foundation of evidence-based research, the work described here operationalizes and applies a set of criteria for evaluating the suitability of sites and trails for forest therapy. Using an iterative process, applying the protocol to sites and trails in two different urban and rural landscape regions helped to specify and refine criteria measures, which in turn helped to the expand the number and types of trails (including paddle and bike trails) and structure them within broader, thematically relevant places. While the depth and breadth of this process help ensure the generalizability of the protocol to further application, the need for its adaptation to different regional and cultural conditions and assessment purposes is both recognized and encouraged. The place concept was an unintended outcome of the protocol development process and one that deserves further attention in future work. In particular, site level landscape history descriptions and visual material [224,225], combined in StoryMaps or other interactive forms of communication [226,227], hold high potential for showing the value and appreciation of places for forest therapy and other nature-based activities as forms of regional placemaking and placekeeping that strengthen connections between people and places.

## Figures and Tables

**Table 1:** Kendall’s tau b correlation coefficients and significance levels ^1^ for site and trail criteria.

Site Criteria *n* = 56	Beauty	Integrity	Tranquility	Accessibility		
Beauty	--					
Integrity	0.578 ***	--				
Tranquility	0.233	0.280 *	--			
Accessibility	−0.013	−0.07	−0.378 **	--		
Trail Criteria ^2^ *n* = 157	Easeful	Attractive	Natural	Built	Explore	Learn
Easeful	--					
Attractive	0.020	--				
Natural	0.044	0.460 ***	--			
Built	0.341 ***	0.053	0.108	--		
Explore	−0.223 **	0.429 ***	0.359 ***	−0.145 *	--	
Learn	0.304 ***	−0.093	−0.084	0.425 ***	−0.273 ***	--

^1^ * *p* ≤ 0.05; ** *p* ≤ 0.01; *** *p* ≤ 0.001. ^2^ Full labels for trail criteria were ease of travel, layout attractiveness, natural features, built and borrowed features, explorable nature, and interpretation and stewardship.

**Table 2:** Mean ranks, Kruskal–Wallis H values, and significance levels ^1^ for site and trail criteria ratings between Northwoods and Chicago study areas and foot, paddle, and bike trail types.

Site Criteria *n* = 56	Northwoods *n* = 29	Chicago *n* = 27	K-W H df = 1	Foot	Paddle	Bike	K-W H
Beauty	30.36	26.50	1.23	--	--	--	--
Integrity	31.10	25.70	1.95	--	--	--	--
Tranquility	35.81	20.65	14.78 ***	--	--	--	--
Accessibility	21.45	36.07	14.99 ***	--	--	--	--
Trail Criteria ^2^ *n* = 157	*n* = 91	*n* = 66	df = 1	*n* = 116	*n* = 24	*n* = 17	df = 2
Easeful	65.80	97.20	22.84 ***	76.61	85.90	85.59	1.539
Attractive	85.46	70.09	5.72 *	77.20	92.04	72.88	3.223
Natural	83.35	73.00	3.65	77.11	94.29	70.32	6.506 *
Built	64.18	99.43	27.13 ***	80.94	69.75	78.85	1.42
Explore	98.01	52.79	46.57 ***	76.97	106.58	53.94	17.509 ***
Learn	62.80	101.33	36.03 ***	88.02	50.69	57.41	23.217 ***

^1^ * *p* ≤ 0.05; *** *p* ≤ 0.001. ^2^ Full labels for trail criteria were ease of travel, layout attractiveness, natural features, built and borrowed features, explorable nature, and interpretation and stewardship.

## Data Availability

Data available upon request.

## Supplementary Materials

The following supporting information can be downloaded at: https://www.mdpi.com/article/10.3390/ijerph22091440/s1, Table S1: Site level characteristics and ratings for the Northwoods study area; Table S2: Site level characteristics and ratings for the Chicago study area; Table S3: Trail level characteristics and ratings for the Northwoods study area; Table S4: Trail level characteristics and ratings for the Chicago study area; Table S5: Comparison of site level ratings for Northwoods versus Chicago study areas; Table S6: Comparison of trail level ratings for Northwoods versus Chicago study areas for all trails; Table S7: Comparison of trail level ratings for Northwoods versus Chicago study areas for foot trails; Table S8: Comparison of trail level ratings for Northwoods versus Chicago study areas for paddle trails; Table S9: Comparison of trail level ratings for Northwoods versus Chicago study areas for bike trails; Document S1: Final protocol; Document S2: Protocol worksheet template; Document S3: Rapid assessment screening worksheet; Document S4: Worksheet example for site and trail networks in the Northwoods study area; Document S5: Worksheet example for site and trail networks in the Chicago study area; Document S6: Illustrated examples of selected trail level criteria and sub-criteria from the two study areas.

## Appendix A

**Table A1:** Listing and characteristics of places (ruled rows), sites (italics), and trails in the Northwoods (numbers 1–8) and Chicago (numbers 9–16) study areas.

Nbr.	Place, Site, and Trail Names	Area (ha)	Tl Trails	Type	Length (km)
1	Pine-Popple Wild Rivers	4673	17		
*1.1*	*Wild Rivers Interpretive Center*	16	1		
1.11	The Return of Trees Interpretive Trail			Foot	1.1
*1.2*	*Pine River Flats*	227	1		
1.21	Pine River Flats Paddle			Paddle	5.3
*1.3*	*Pine River Outcrops*	129	2		
1.31	Outcrops-River Loop			Foot	1.3
1.32	Outcrops-Western Trail			Foot	1.6
*1.4*	*Pine River-Meyers Falls/Bull Falls*	146	3		
1.41	Meyers Falls Trail			Foot	0.3
1.42	Bull Falls Trail			Foot	0.8
1.43	Goodman Grade-Falls Bike Tour			Bike	12.9
*1.5*	*Pine River- LaSalle Falls*	809	1		
1.51	LaSalle Falls Trail			Foot	3.2
*1.6*	*Pine River-Breakwater Falls/Pine River Flowage*	202	3		
1.61	Breakwater Falls North Bank			Foot	0.8
1.62	Breakwater Falls South Bank			Foot	1.5
1.63	Pine River Flowage Paddle			Paddle	1.6
*1.7*	*Pine River Oxbow*	486	1		
1.71	Oxbow Paddle			Paddle	5.6
*1.8*	*Savage-Robago Wild Lakes Complex*	762	5		
1.81	Savage Lake Shoreline Walk			Foot	1.0
1.82	Savage Lake Paddle			Paddle	0.8
1.83	Wild Lakes Land and Water Loop			Foot	2.4
1.84	Wild Lakes Bike Loop			Bike	20.9
1.85	Savage Lake Road			Bike	12.1
2	Florence County Forest and Parks	14,756	11		
*2.1*	*Sea Lion Lake*	30	4		
2.11	Point Trail			Foot	0.3
2.12	Loop Trail			Foot	1.3
2.13	Ridge Trail			Foot	2.6
2.14	Sea Lion Lake Islands and Bays Paddle			Paddle	0.5
*2.2*	*Lake Emily Recreation Trail*	263	4		
2.21	North Loop			Foot	3.1
2.22	South Loop			Foot	1.8
2.23	South Loop Little Lake Emily Spur			Foot	1.2
2.24	Little Lake Emily Paddle			Paddle	0.8
*2.3*	*Halls Creek Trails*	672	3		
2.31	Red Loop			Foot	1.6
2.32	Green Loop			Foot	2.4
2.33	Blue Loop			Bike	4.7
3	Rainbow Trail	5666	18		
*3.1*	*Fox Maple Woods*	17	4		
3.11	Main Trail			Foot	0.8
3.12	Marsh Loop			Foot	0.8
3.13	Upland Loop			Foot	1.0
3.14	Big Loop			Foot	1.3
*3.2*	*Perch Lake*	121	3		
3.21	Loop Trail North Portion (to site 2)			Foot	1.9
3.22	Full Loop Trail (winter only)			Foot	3.7
3.23	Perch Lake Paddle			Paddle	0.8
*3.3*	*CCC Camp Rainbow*	567	2		
3.31	Camp Perimeter Loop			Foot	0.8
3.32	Rainbow Fire Tower			Foot	1.6
*3.4*	*Whisker Lake Wilderness*	3006	4		
3.41	Whisker Lake Trail Portion			Foot	4.4
3.42	Riley Lake Trail Portion			Foot	4.2
3.43	Edith Lake Paddle			Paddle	0.8
3.44	Edith Lake—Montagne Creek Paddle			Paddle	1.6
*3.5*	*Rainbow Hunter Walking Trails*	567	4		
3.51	North Loop			Foot	3.4
3.52	Middle Loop			Foot	2.2
3.53	South Loop			Foot	3.2
3.54	West Trail/Rainbow Creek			Foot	2.4
*3.6*	*Multi-Sites*	2428	1		
3.61	Rainbow Trail CCC Sites Bike Tour			Bike	4.8
4	Lauterman/Lost Lake Recreation Areas	3035	9		
*4.1*	*Lauterman National Recreation Trail*	911	3		
4.11	Beginner’s Trail Loop			Foot	1.5
4.12	Lauterman Lake Trail Loop			Foot	3.5
4.13	Chipmunk-Little Porky Loop			Bike	11.9
*4.2*	*Lost Lake Recreation Area*	405	6		
4.21	Lakeshore Trail Loop			Foot	2.6
4.22	Assessor’s Interpretive Trail Loop			Foot	1.5
4.23	Lakeshore-Ridge Trail Loop			Foot	4.2
4.24	CCC Cabins to Ridge Trail Loop			Foot	1.5
4.25	West Lakeshore Trail			Foot	1.6
4.26	Lost Lake Paddle			Paddle	1.1
5	Brule River Cliffs	2023	4		
*5.1*	*Brule River Cliffs*	2023	4		
5.11	Old Field Loop			Foot	2.0
5.12	Cliffs-River Loop			Foot	2.8
5.13	Brule River Cliffs Paddle			Paddle	8.1
5.14	Brule River Cliffs Bike Loop			Bike	17.7
6	Hidden Lakes	4856	12		
*6.1*	*Hidden Lakes Trail*	4856	6		
6.11	Franklin Nature Trail			Foot	1.6
6.12	Franklin Nature Trail—Two Dutchmen Lake Loop			Foot	4.8
6.13	McKinley Lake—Three Johns Lake segment			Foot	3.2
6.14	McKinley Lake—Luna Lake segment			Foot	3.2
6.15	Luna Lake Trail			Foot	3.2
6.16	White Deer Trail			Foot	2.3
*6.2*	*Hidden Lakes Dispersed Sites*	4856	4		
6.21	Three Johns Lake Paddle			Paddle	1.2
6.22	McKinley Lake Paddle			Paddle	1.2
6.23	Two Sisters Lake Bog Walk			Foot	0.3
6.24	Quartz Lake Paddle			Paddle	1.1
*6.3*	*Other Area Trails of Interest*	100	2		
6.31	Healing Nature Trail			Foot	0.6
6.32	Sam Campbell Memorial Trail			Foot	3.4
7	Spread Eagle Barrens	3440	12		
*7.1*	*Fire Lane Rd.*	607	3		
7.11	Fire Lane Rd. Forest Edge-Barrens Short Loop			Foot	2.2
7.12	Fire Lane Rd. Loop			Foot	5.6
7.13	Fire Lane Rd. to Menominee River Bike Loop			Bike	5.6
*7.2*	*Barrens Lake*	182	3		
7.21	Short Loop			Foot	2.2
7.22	Full Loop			Foot	3.7
7.23	Barrens Lake Paddle			Paddle	0.8
*7.3*	*Lake Anna*	202	5		
7.31	West Loop			Foot	3.0
7.32	East Loop			Foot	2.7
7.33	Full Loop			Foot	3.6
7.34	Bog Loop			Foot	0.6
7.35	Lepage Creek Overlook			Foot	0.0
*7.4*	*Sand Lake*	61	1		
7.41	Sand Lake Paddle			Paddle	0.8
8	Fumee Lake Natural Area	728	7		
*8.1*	*Little Fumee Lake—South Ridge*	40	3		
8.11	Little Fumee Lake Loop			Foot	2.5
8.12	Little Fumee Shoreline Paddle			Paddle	0.8
8.13	South Ridge Loop			Foot	5.2
8.14	South Ridge-South Loop Portion			Foot	3.7
*8.2*	*Fumee Lake*	324	4		
8.21	Fumee Lake-North Ridge Trail Loop			Foot	5.3
8.22	Fumee Lake-Mountain Trail Loop			Foot	5.3
8.23	Mountain-North Ridge Short Loop			Foot	1.6
8.24	Big Fumee Lake Bike Loop			Bike	8.1
9	West Ridge/North Park Neighborhood Natural Areas	526	12		
*9.1*	*North Park Village*	24	5		
9.11	Nature Center Wetland Loop			Foot	0.5
9.12	Nature Center Woodland Loop			Foot	0.8
9.13	Nature Center Wetland-Woodland-Savanna Loop			Foot	1.2
9.14	Rock Garden Loop			Foot	0.2
9.15	Walking Stick Woods Trail/Nature Place Space			Foot	0.8
*9.2*	*West Ridge Nature Park/Rosehill Cemetery*	142	4		
9.21	Nature Park Woodland Loop			Foot	0.7
9.22	Nature Park Lake Loop			Foot	1.1
9.23	Nature Park Woodland-Lake Loop			Foot	1.8
9.24	Rosehill Cemetery Tree Meander			Foot	1.6
*9.3*	*Indian Boundary Park*	5	2		
9.31	Natural Area Loop			Foot	0.3
9.32	Park Loop			Foot	0.7
*9.4*	*Multi-Sites*	526	1		
9.41	Neighborhood Natural Areas Bike Loop			Bike	6.8
10	Loyola Lakeshore Natural Areas	34	7		
*10.1*	*Loyola University Lakeshore Campus*	14	1		
10.11	Lakeshore Campus Loop Walk			Foot	0.5
*10.2*	*Loyola-Leone Parks*	16	3		
10.21	Loyola Natural Area-Park Loop			Foot	0.8
10.22	Loyola Natural Area-Pier Loop			Foot	0.8
10.23	Leone Natural Area-Park Loop			Foot	0.4
*10.3*	*Multi-Sites*	20	3		
10.31	Hartigan-Leone Beach Walk			Foot	2.2
10.32	Loyola Waterfront Paddle			Paddle	1.4
10.33	Loyola Waterfront Bike Route			Bike	3.2
11	Lincoln Park North Natural Areas	81	8		
*11.1*	*Montrose Point*	11	4		
11.11	Bird Sanctuary Main Loop			Foot	0.6
11.12	Bird Sanctuary and Montrose Beach Dunes Loop			Foot	1.2
11.13	Point-Dunes-Lake-Prairie Loop			Foot	1.4
11.14	Breakwater-Pier			Foot	2.4
*11.2*	*Marovitz Savanna*	4	1		
11.21	Savanna Loop			Foot	0.9
*11.3*	*Bill Jarvis Migratory Bird Sanctuary*	4	1		
11.31	Bird Sanctuary Loop			Foot	0.6
*11.4*	*Multi-Sites*	81	2		
11.41	Montrose Waterfront Paddle			Paddle	1.6
11.42	Montrose Area Bike Loop			Bike	7.6
12	Lincoln Park South Natural Areas	53	5		
*12.1*	*Alfred Caldwell Lily Pool*	1	1		
12.11	Lily Pool Loop			Foot	0.4
*12.2*	*Nature Museum*	0.4	1		
12.21	Deb Lahey Nature Trails			Foot	0.5
*12.3*	*North Pond*	5	1		
12.31	Natural Area Loop			Foot	1.1
*12.4*	*Lincoln Park Zoo*	5	1		
12.41	Nature Boardwalk			Foot	0.8
*12.5*	*Multi-Sites*	53	1		
12.51	Fullerton Area Bike Loop			Bike	4.0
13	Caldwell Preserves	150	6		
*13.1*	*Sidney Yates Flatwoods*	40	2		
13.11	Savanna-Flatwoods-Loop			Foot	0.8
13.12	Savanna-Flatwoods-River Loop			Foot	1.2
*13.2*	*Oxbow Prairie*	3	1		
13.21	Prairie Loop			Foot	0.5
*13.3*	*Bunker North Flatwoods*	31	1		
13.31	Flatwoods meander			Foot	1.1
*13.4*	*Multi-Sites*	101	2		
13.41	Caldwell Preserves Chicago River Paddle			Paddle	3.2
13.42	Caldwell Preserves Bike Tour			Bike	7.1
14	Harms Woods Preserves	134	6		
*14.1*	*Harms Woods Nature Preserve*	68	4		
14.11	Woodland-River West Loop			Foot	1.6
14.12	Woodland-River East Loop			Foot	1.6
14.13	Woodland-Meadow Loop			Foot	1.2
14.14	Full Loop			Foot	3.9
*14.2*	*Multi-Sites*	93	2		
14.21	Harms Woods Chicago River Paddle			Paddle	3.2
14.22	Harms Woods Bike Loop			Bike	4.4
15	Skokie Marsh	504	14		
*15.1*	*Skokie Lagoons Forest Preserve*	348	8		
15.11	Inner Trail-Main Trail			Foot	1.6
15.12	Inner Trail-North Loop			Foot	2.6
15.13	Inner Trail-South Loop			Foot	4.8
15.14	Inner Trail-Full Loop			Foot	6.6
15.15	Skokie Lagoons Paddle—Lagoons 4–5 Loop			Paddle	4.8
15.16	Skokie Lagoons Paddle—Lagoon 3 Loop			Paddle	1.6
15.17	Skokie Lagoons Paddle—Lagoons 1–3 Loop			Paddle	7.2
15.18	Skokie Lagoons North Branch Trail Bike Loop			Bike	7.1
*15.2*	*Chicago Botanic Garden*	156	6		
15.21	McDonald Woods South Loop			Foot	0.4
15.22	McDonald Woods North Loop			Foot	0.6
15.23	McDonald Woods Big Loop			Foot	1.1
15.24	Japanese Garden			Foot	0.6
15.25	Dixon Prairie			Foot	1.5
15.26	Chicago Botanic Garden Bike Trail			Bike	3.2
16	Somme Preserves	153	8		
*16.1*	*Somme Prairie*	28	1		
16.11	Prairie Loop			Foot	1.2
*16.2*	*Somme Prairie Grove*	34	4		
16.21	Vestal Grove Savanna Loop			Foot	1.1
16.22	Prairie-Grove Loop			Foot	1.9
16.23	Prairie Inner Loop			Foot	2.0
16.24	Prairie Outer Loop			Foot	2.4
*16.3*	*Somme Woods*	91	3		
16.31	West Inner Loop			Foot	0.5
16.32	West Outer Loop			Foot	1.0
16.33	East Loop			Foot	2.1

## Appendix B

**Table A2:** Conceptual definition and evaluation procedures for site level criteria and sub-criteria dealing with landscape character and quality.

Criteria and Sub-Criteria	Definition	Evaluation Procedures	Data Sources Used
Landscape character and history			
	The essential geographical and social characteristics of the landscape setting and its physical, biological, and/or cultural patterns and features that define the site and surrounding landscape. While the focus is on existing character, information on landscape history can provide important clues on a site’s essential patterns and features that contribute to its desired character.	List the key characteristics of the site’s geographical and social setting, jurisdiction, land use type, land cover or ecological landscape type, geographic or cultural region, and special designation. Provide a summary narrative of the site’s landscape history that has influenced current patterns and features. Landscape character is not rated but helps set the context for evaluating other site level criteria.	Regional and site-specific information is drawn from a wide variety of historical and current sources including federal, state, county, and local public land management agency reports, maps, aerial imagery, websites, online land resource and ownership information systems, and scholarly and popular books and articles relating to geological, cultural, and ecological landscape history.
Beauty			
	The variety, vividness, and/or uniqueness of a site’s existing landscape character patterns and features that together contribute to its aesthetic quality.	Rate and provide a summary narrative describing the physical (landform, water, rock/soil), biological (vegetation, wildlife), and/or cultural (heritage, land use) patterns and features in terms of their variety-diversity and vividness-prominence on the site, highlighting any unique or special features. Generally, sites high in elements of variety, vividness, and/or uniqueness are high in beauty.	Field study notes and photography, USGS topographic and/or county Lidar mapping, Google Earth satellite imagery (color, seasonal), site-specific reports and website information.
Integrity			
	The condition or intactness of a site’s desired landscape character patterns and features that together contribute to its ecological, recreational, and/or cultural-historic quality.	Rate and provide a summary narrative describing the ecological (physical, biological, environmental), recreational (facilities and scenery) and/or cultural-historic (built environment, landscape) patterns and features in terms of their condition (level of maintenance, degree of degradation), or intactness (wholeness-fragmentation, pristineness, biodiversity). Sites in high condition and/or intactness are generally of high integrity.	Field study notes and photography, information on historical vegetation types and patterns (historical GLO survey data and contemporary syntheses at site and regional scales), current site planning and management information and data from reports and websites, cultural and archeological studies and assessments, historical and contemporary aerial and satellite imagery.
Tranquility			
	The sensory, social, and environmental qualities of or intrusions into a site’s peacefulness and isolation that together promote psychological and physiological comfort and minimize distractions.	Synthesizing the information from the sub-criteria below, rate and provide a summary narrative describing the setting, sensory, social, and environmental conditions that intrude upon the site’s tranquility. Sites isolated from competing uses with low levels of intrusions are generally considered high in tranquility.	See sub-criteria below.
Setting	The size of the site on which the trail is located and its degree of isolation from potentially competing adjacent uses.	Measure the size of the site using Google Earth (or similar) and characterize the adjacent land uses surrounding the site in terms of their compatibility. If the site has no formal boundaries within its larger context, draw a polygon defining the immediate area around the trail or trail network, following natural or human-defined boundaries or transitions.	Site-specific information, Google Earth (or similar applications) used to plot trails and site boundaries (image overlay).
Visual	Visual impacts of development seen from trails on the site.	Describe type, distance zones (immediate foreground, foreground, middleground, background), magnitude/scale, and compatibility of intrusive visible development including roads, buildings, and land uses. Note key locations along trails where visual impacts are experienced.	Field study and photography, Google Earth to assist in estimating distance zones.
Sound	Noise or other negative sound impacts heard from trails on the site.	Describe the types and magnitude of sound disturbances including roads, buildings, and land uses and activities. These sources are usually external to the site but may also come from other site uses and activities. For most sites, road noise is the major intrusion and one should measure (Google Earth) and report the shortest and furthest distance of trails on the site from roadways and report roadway type and estimated traffic volume. For sites near busy airports note whether trails are in the flightline of takeoffs and landings.	For distance to roads, Google Earth. For traffic volume, state Department of Transportation websites. For transportation related noise, see the National Transportation Noise Map. See also other transportation-specific (e.g., aircraft overflights) and composite anthropogenic sound impact websites and apps.
Other	Offensive odors or other sensory intrusions experienced from trails on the site.	Describe the type and magnitude of any other potential intrusions such as smells from surface waters, farm fields, or other ambient or point sources of offensive odors, dust or smoke, light, etc. Note any temporal variations.	Field study and site-specific information.
Social	Use level and compatibility of other users on trails or adjacent use areas.	Estimate the average range in trail use levels in terms of numbers of parties encountered. Note user type and compatibility, including those in adjacent use areas that might affect privacy/tranquility. Note temporal/spatial patterns of use for potential for off-peak visits.	Field study.
Environmental	Physical and biotic factors that can impact comfort and physical safety.	Record everyday or seasonal environmental conditions that could be challenging and induce physical or emotional stress to some users, e.g., wet/muddy trail areas, steep grades, narrow trails, rocks/roots, downed trees, insects, poisonous plants, etc.	Field study.
Accessibility			
	The ability and ease with which people can get to and use a site, including its proximity from home, available trailhead facilities, user fees, and range of trail options and degrees of difficulty.	Synthesizing the information below, rate and provide a summary narrative describing the proximity (distance, travel time), available facilities (parking, toilet, other), user fees (parking, trail use, guide), and trail options (number, type, and range of difficulty) that facilitate or impede access and use of the site. Generally, sites that are nearby, have basic trailhead facilities, are reasonably priced, and have a range of trail options are considered good candidates for forest therapy.	See sub-criteria below.
Proximity	Nearness to site trailhead from some logical or popular point of origin.	Using Google Maps or a similar procedure, calculate the distance and/or time to the main trailhead of the site from a chosen point of origin. Choose a maximum threshold time or distance that seems reasonable for use in establishing an evaluation rating for site accessibility. For this study, most trails were selected to be within a 30 min maximum driving distance from a centrally located nature center.	Google Maps.
Trailhead facilities	Key support amenities that are present at or near the site trailhead.	List and briefly describe parking, toilet, drinking water, and other support facilities available at or within close access to the trailhead. While the level of trailhead facility development is highly site dependent, most trailheads should at least have a parking area and, ideally, a portable toilet or outhouse.	Field assessment, site-specific information.
User fees	Costs or other requirements such as membership that may pose barriers to entry and use of a site and its trails.	Describe any parking, day use, or other entry fees needed to access the trailhead or individual trails on the site, in addition to guide or tour fees if applicable. Nominal costs are often acceptable but steep costs can impact accessibility.	Field assessment, site-specific information.
Trail and accessibility options	The number, type, and range of difficulty of trails available at a site.	Using design and construction information on individual trails described in the sections below, provide a brief narrative summarizing the number, type, and range of difficulty (length, steepness, or other potential challenges) of all trails available within the site. While a single trail geared to wheelchair users may be considered highly accessible, sites with multiple trails, different trail types, and a range of levels of challenge may be better suited to serving a diversity of users and interests.	Field assessment and trail level measures, site-specific information.

**Table A3:** Conceptual definition and evaluation procedures for trail level criteria and sub-criteria dealing with design and construction.

Criteria and Sub-Criteria	Definition	Evaluation Procedures	Data Sources Used
Ease of travel			
	Trail design parameters (trailhead distance, length, surface, width, slope) and obstacles (physical accessibility barriers) that contribute to the ease or difficulty of trail use.	Synthesizing the information below, make an evaluative rating incorporating distance to trailhead, length, surface, width, slope, and accessibility barriers to assess the ease of travel on the trail. Generally short trails readily accessible from trailheads that follow easy grades with comfortable widths and stable natural surfaces with few obstructions are preferred.	See sub-criteria below.
Distance to trailhead	The distance of travel between the starting point (e.g., parking lot) to where the actual forest therapy trail experience begins.	Measure in Google Earth from starting point (e.g., parking lot) to where the actual forest therapy trail experience begins. Some trails may require a walk along a main trail, road, or less desirable trail segment before in order to reach the starting point. Generally, trail layouts with short trailhead distances are desirable.	Field assessment, Google Earth, site-specific information.
Length	The distance along trail or trail segment from the trailhead and back.	Trail length can be measured using Google Earth or similar program, in the field with a phone app such as Avenza, or taken from site-specific information such as websites and brochures. Most trails in this assessment were not designed for forest therapy, and for sites with longer trails and trail networks it may be desirable to map a route that uses a portion of a trail or connected segments of two or more trails of appropriate length for a forest therapy trail visit and develop a separate evaluation for that particular configuration. Optimal length can vary, but foot trails in the range of 1–2 km are often considered as best suited for forest therapy.	Field assessment, Google Earth, site-specific information.
Surface	The composition or material of the trailbed tread surface.	Field observation: in the case of multiple trail surfaces record different surface types and dominant surface(s). Natural materials are usually preferred for forest therapy trails so long as they provide a firm and stable surface and resist erosion from weathering and use.	Field assessment.
Width	The width of the trailbed tread surface or the right-of-way if no clear trailbed is apparent (i.e., mown grass routes, snow-covered corridors).	In the field, visually estimate or set a tape measure perpendicular to trail tread at a number of locations along the length of the trail. Trail routes in this assessment often had variable widths so record minimum, maximum, and modal trail width. While trail width preferences vary by trail use, class, and other factors, generally for foot trails in low to moderate use sites, tread widths of 1–2 m with active ROW vegetative management allow users an immersive experience with some room for other users to pass.	Field assessment.
Slope	The running slope of the trail surface, specified in terms of maximum slope and average slope.	Plot trail in Google Earth based upon trail maps, Avenza smartphone app, or other data and use elevation profile data to record maximum slope and average slope. If necessary, adjust values based on field assessment and topographic maps. Foot trails that are nearly level with some gentle grades for variation are suitable for most users.	Google Earth, cross-check with field assessment, smartphone trail app, and/or topographic maps.
Accessibility barriers	The type and frequency of obstacles encountered in the trailbed tread surface and right-of-way corridor, including side and overhead height obstacles.	Field observation: list type and frequency of barriers or obstacles such as rocks and exposed roots in the trailbed, fallen logs, brush, side and overhead branches, primitive water body crossings, wet and muddy stretches, uneven pavement, stairs, slippery surfaces, etc. Most trail users prefer a trail corridor with few obstructions or barriers to accessibility, and trails with long stretches of wet areas or brush should be avoided.	Field assessment.
Attractiveness of layout			
	The design configuration (alignment, route type and directionality, views, spaces, and changes) of a trail that contributes to an engaging experience.	Synthesizing the information below, make an evaluative rating incorporating alignment, route type and directionality, key views, spaces, and related spatial-temporal changes encountered along the trail that enhance forest therapy experiences. Look for curving to winding loop trails with a variety of views, private and group spaces, and changes along the route.	See sub-criteria below.
Alignment	The horizontal and vertical layout or routing of a trail corridor through a site, the path it follows to connect features or destinations in the landscape with sensitivity to functional, environmental, and aesthetic considerations.	Along with providing an overview description of topographic and landform characteristics, summarize the dominant horizontal and vertical alignment character of the trail layout through field assessment, aided by quantitative measures of degree of route straightness or curvature and changes in elevation (Google Earth, Avenza phone app, topographic maps). Generally, gently curving to winding trails that traverse varied topography with some changes in grade contribute to an engaging experience.	Field assessment (observation and/or phone app, e.g., Avenza), Google Earth, topographic maps.
Route type and directionality	The path a trail follows from and back to the trailhead, usually described by a loop or linear configuration or some permutation of either or both.	Record route type and directionality using field assessment or other site information from websites, maps or brochures. Route type is usually described by a loop or linear (there-and-back) configuration or some permutation of either (e.g., stacked loop) or both (partial loop). Some loop trails (e.g., cross country skiing) are signed for one-way travel, and many paddle trails on rivers are considered one-way due to their currents making upstream travel difficult. Generally, loop routes are considered preferable, while one-way trails can lessen encounters and conflicts with other parties.	Field assessment, site-specific information.
Views	Types and observer position of views experienced along the trail, including the location of key observation points (KOPs).	Through field assessment, describe view types (panorama/vista/distant, feature/focal, enclosed/interior/canopied, detail/close-up) and observer position (level, above, below) of typical and important views. Topographic maps, aerial imagery, and/or the placemark feature on a smartphone mapping application can be useful to note the location of KOPs. Trails with a variety of views and view types are preferred.	Field assessment, topographic maps and aerial imagery, smartphone trail mapping app with placemark feature.
Spaces	Openings, clearings, key locations, or other spaces that occur naturally or are created through vegetation management along the trail that provide a pleasant, suitable stationary setting for private reflection (“sitspots”) or group forest therapy activities (“invitations”).	Through field assessment, record the presence of suitable openings, clearings, key locations, or other spaces that occur naturally or are created through vegetation management along the trail for private sitspots or group forest therapy invitations. Topographic maps, aerial imagery, and/or the placemark feature on a smartphone mapping application can be useful to note the location of these spaces. Trails should have spaces every so often for individuals to stop and rest and reflect, and/or for groups to assemble for guided activities.	Field assessment, topographic maps and aerial imagery, smartphone trail mapping app with placemark feature.
Changes	The number or variety of transitions along the length of a trail that create different “rooms” or “reaches” and provide noticeably different perceptual and sensory experiences.	Using field assessment with the aid of aerial imagery or other site specific information sources, describe the number or variety of transitions in elevation, vegetation types, spatial patterns, or other features along the length of a trail that create different “rooms” or “reaches” and provide noticeably different perceptual and sensory experiences. Preferred trails have a variety of changes along their length.	Field assessment.

**Table A4:** Conceptual definition and evaluation procedures for trail level criteria and sub-criteria dealing with key features and opportunities.

Criteria and Sub-Criteria	Definition	Evaluation Procedures	Data Sources Used
Natural Features			
	Objects and elements of nature occurring along the trail that enhance forest therapy experiences including dominant vegetation cover and distinctive trees, water bodies, wildlife and other elements along with related notable multisensory characteristics.	Using the information below, make an evaluative rating that expresses the amount or prominence of natural features visible and accessible along the trail. While each trail will have a unique combination of natural features, trails with big and/or distinctive trees, water that is prominent and physically accessible, observable wildlife, and/or other distinctive natural vegetation, landform, or rock features and related multisensory effects will enhance forest therapy experiences.	See sub-criteria below.
Vegetation cover	The dominant vegetation community types visible along the trail corridor.	Through field assessment along with the aid of aerial imagery and available site-specific information, list and briefly describe the dominant natural or cultural vegetation community types visible along the trail. For forest communities it may be helpful to include dominant species, density, and age class of overstory tree cover. Inclusion of this information helps set the context for key natural features described below.	Field assessment, aerial imagery, site-specific information.
Trees	Large and/or distinctive trees and related multisensory characteristics.	Through field assessment, identify any trees with noticeably large trunks, noting if many such trees occur, in groups, dispersed, or whether the trail corridor goes through a mature or old growth forest. In addition to big trees, note any distinctive tree types or species, those with distinctive branching or other growth habits, growths such as burls or trees with distinctive moss, lichen, algae, or fungus growing on them, distinctive standing dead or downed trees, etc.	Field assessment, smartphone trail mapping app with placemark feature.
Water	Type, visual prominence, access, and usability of natural or human modified/created water bodies and water-related sensory effects.	Using field assessments along with other available site information, list water bodies visible along the trail in terms of water body type, including natural water bodies (e.g., lake, river, wetland) as well as any human created water features (e.g., fountains, waterfalls). Describe their visual prominence in terms of their size, how close they are to the trail, whether they can be accessed visually or physically, and the duration of time or percentage of trail length that they are visible. For water bodies that are physically accessible give some indication of their usability for human contact, taking into account physical safety and water quality, use restrictions, and desirability for contact from touching to partial to full immersion.	Field assessment, topographic maps and aerial imagery, other available site information.
Wildlife	Commonly viewed and/or seasonally important mammals, birds, insects, etc., along with prominent habitat, nesting, and observation opportunities, and key wildlife related sensory effects and seasonal opportunities.	Using field assessments along with other available site information, list mammals, birds, insects, etc., along with prominent habitat (e.g., beaver dams and ponds, productive wetland areas), nesting (eagle, egret nests), and observation opportunities (e.g., protected breeding areas, wildlife blinds, seasonal migrations).	Field assessment, available site-specific information.
Other	Other distinctive vegetation, landform, and rock features and related sensory effects.	Using field assessments along with other available site information, list or briefly describe other distinctive vegetation (ferns, moss, fungi), landform (canyons, ridges, hilltops, flats or plains), and rock (large rocks, outcrops, rocky shorelines and lake or stream bottoms) features.	Field assessment, available site-specific information.
Built and borrowed features			
	Human-built, naturally occurring, or human-adapted natural features that serve utilitarian, aesthetic, or symbolic functions along the trail for seating, gateways, shelter, and other purposes.	Using the information below, make a summary evaluative rating that expresses the amount or prominence of human-built, naturally occurring, or human-adapted objects or spaces present along the trail that serve utilitarian, aesthetic, or symbolic functions for seating, gateways, shelter, and other purposes. Built and borrowed features that work in harmony with the trail’s setting and natural features can help enhance forest therapy experiences.	See sub-criteria below.
Seating	Human-built, naturally occurring, or human-adapted natural objects or spaces that afford places to sit along the trail.	In a field assessment, identify and note the location of seating and types of seating from informal natural materials and features such as fallen logs and stones to borrowed or modified natural materials such as cut tree stumps and cut stone slabs, to manufactured seating such as benches, chairs, and picnic tables.	Field assessment, smartphone trail mapping app with placemark feature.
Gateways	Human-built, naturally occurring, or human-adapted natural objects and spaces that provide a physical or symbolic entry or exit point to a trail.	In field assessment, note any signs, markers, archways, gates, rocks, trees, modifications in vegetation, ground textures and materials, or other human-built (e.g., metal gates, trailhead information kiosk), naturally occurring (big standing or fallen trees, boulder), or human-adapted natural objects and spaces (trail marker trees, rocks arranged in place), singly or in combination, that provide a physical or symbolic entry or exit point to a trail.	Field assessment.
Shelter	Human-built, naturally occurring, or human-adapted natural objects and arrangements that provide a partial buffer or more complete protection from weather elements including sun, wind, and temperature, or precipitation.	Using a field assessment and any available site-specific information, identify and locate any human-built (gazebos, picnic shelters), naturally occurring (big canopy trees), or human-adapted natural objects and arrangements (lean-tos made of branches) that provide a partial buffer or more complete protection from weather elements including sun, wind, and temperature, or precipitation.	Field assessment, available site-specific information.
Other	Additional built and borrowed features that facilitate use and protect people and the environment, special features to enhance the nature experience, and other features or evidence of past features and activities that reflect and maintain the cultural-historical landscape.	Using a field assessment and any available site-specific information, list any additional human-built, naturally occurring, or human-adapted natural features including boardwalks (mention material), bridges (from logs placed across a stream to major bridge constructions), fencing (split rail, metal, etc.) or other features that facilitate use and protect people and the environment; special features to enhance (e.g., wildlife blinds, observation platforms, sculptures) the nature experience; and other features or evidence of past features and activities (buildings, stone walls, building foundations) that reflect and maintain the cultural-historical landscape.	Field assessment.
Explorable Nature			
	Policies and design features that provide or restrict types of activities that can enhance forest therapy experiences.	Using the information below, make a summary evaluative rating that expresses the degree of allowable uses or restrictions, museumification, and engagement on and off the trail. Generally policies and design features that permit responsible interactions with natural features within and outside the immediate trail corridor are desirable.	See sub-criteria below.
Uses and restrictions	Written or otherwise expressed policies that allow, provide for, or restrict off-trail exploration, play, sampling, foraging, collecting, and related activities as part of a forest therapy experience.	Using available information at trailhead kiosks or online for site or agency, list and describe allowable activities or activity restrictions as they pertain to going off-trail, sampling, foraging, collecting (take home or onsite use), and related activities, including provisions for activities such as a firepit for making a fire for use in a forest therapy tea ceremony.	Information at trailhead kiosks or online for site or agency.
Museumification	Signage, physical or symbolic barriers, or visual cues installed along the trail that have the effect of limiting forest therapy interactions to mostly visual on-trail observation, as if visitors were in a museum.	Field observation—list and describe signs, fencing, rope barriers, or other physical or symbolic barriers or visual cues installed continuously or at multiple points along the trail that remind visitors to “look but don’t touch” that have the effect of limiting user experience and multisensory engagement to mainly visual observation.	Field assessment.
On-trail engagement	Design and management characteristics of the trail and its right of way that facilitate or hinder engagement and interaction with plants and other natural features of the immediate trail setting.	Briefly describe whether and how trail design (e.g., width, alignment) and right of way (ROW) management (e.g., mowing and trimming of vegetation) facilitate or hinder engagement and interaction with plants and other natural features within the immediate trail setting.	Field assessment.
Interpretation and Stewardship			
	Onsite signage and on- and offsite programs and other opportunities that help enhance nature experiences, knowledge, appreciation, and stewardship behavior.	Using the information below, make a summary evaluative rating of the level of interpretation and learning/stewardship opportunities available on or in association with the trail. Attractive, contextually compatible interpretive signage and auxiliary information, programs, and activities that facilitate environmental learning and involvement can enhance nature experiences over the short and long term.	See sub-criteria below.
Signage	Signs or markers along the trail that help to interpret or enhance nature experiences and appreciation.	In field assessment, identify and describe any informational or interpretive signage or markers along the trail.	Field assessment.
Environmental learning and stewardship opportunities	Programs, volunteer workdays, on- and off-site activities, and other opportunities aimed at educating visitors about the site and trail and/or involving them in efforts to protect, maintain or restore the site and trail’s natural or cultural qualities.	Mostly provided through off-site information on agency or organization websites, identify any programs or events (guided nature walks), volunteer workdays (litter pickups, trail maintenance, ecological restoration workdays), or other on- and off-site activities (e.g., nature center exhibits) aimed at educating visitors about the site and trail and/or involving them in efforts to protect, maintain or restore the site and trail’s natural or cultural qualities.	Field assessment, available site-specific information.
