# Supplementary material for: Forest Therapy Trails: Development and Application of an Assessment Protocol
Source: Int J Environ Res Public Health. 2025 Sep 16;22(9):1440. doi: 10.3390/ijerph22091440 (PMC12470198; doi:10.3390/ijerph22091440)
Supplement: Supplementary file 1 [file ijerph-22-01440-s001.zip › Supp Doc S1 Detailed Protocol.pdf]

Forest Therapy Site and Trail Evaluation Protocol

Part I. Site Level Criteria

1. Landscape Character and History

Definition
The essential geographical and social characteristics of the landscape setting and its physical, biological, and/or cultural patterns and features that define the site and surrounding landscape. At a broad level, landscape character descriptions provide the frame or context within which sites are evaluated and compared. At a more detailed level, they describe the particular characteristics that give each site its unique sense of place. While the focus is on existing character, information on landscape history and planning initiatives can provide important clues on a site's essential patterns and features that contribute to its desired character.

Evaluation Procedures
List the key characteristics of the site's geographical and social setting (e.g., urban, suburban, suburban-rural fringe, rural, wildland-urban interface, wildland), jurisdiction (private, city, county, state, federal), land use type (e.g., park, forest or nature preserve), land cover or ecological landscape type (e.g., forest, Northern Hardwood Forest, pine barrens), geographic or cultural region (e.g., Chicago Wilderness, Northwoods), and special designations (e.g., State Natural Area, Wilderness, National Recreation Trail, etc.). Provide a summary narrative of the site's landscape history (e.g., post-glacial geology, land use practices and ownership) that has influenced current patterns and features.

Evaluation Rating
not rated

Additional Explanatory Notes
Depending on the goal of the effort, the number, type, and specificity of descriptors included can be useful in describing a single site and classifying and comparing between a portfolio of sites. While these descriptors help establish the basic landscape context, the summary narrative provides further detail about the landscape characteristics that give each site its unique sense of place or "genius loci." Although landscape character is not rated, existing landscape character helps set the frame or context for evaluating the beauty of a site and information about landscape history and desired landscape character helps to establish and evaluate site integrity. On a practical level, a landscape character

description can also help visitors in selecting a trail and can help guides in reading the landscape and communicating to their clients via information and "invitations."

Data Sources Used

Regional and site-specific information is drawn from a wide variety of historical and current sources including federal, state, county, and local public land management agency reports, maps, websites, and online land resource and ownership information systems; scholarly and popular books and articles relating to geological, cultural, and ecological landscape history; map collections including land surveys and plat maps (1850s – current), USGS topographic maps (1880s – current), special purpose maps (e.g., railroad and highway maps, forestry and other resources, Wisconsin Economic Land Inventory (Bordner) survey); aerial and satellite imagery (1938 – current); and county property deed historical records, historical newspaper articles, and historical and contemporary tourism brochures and maps.

Data Sources Key URL links used

For the Northwoods study area many historical reports, maps, and aerial photos are available online through the University of Wisconsin-Madison and State Historical Society libraries and special collections; https://www.library.wisc.edu/; https://search.library.wisc.edu/digital/AAerialWI; https://digicoll.library.wisc.edu/SurveyNotes/; https://search.library.wisc.edu/digital/AWILandInv. For the Chicago study area many historical reports, maps and photographs relating to the Forest Preserve District of Cook County are online: https://researchguides.uic.edu/fpdcc/photographs; Chicago park historical photos and summary descriptions: https://www.chicagoparkdistrict.com/about-us/history-chicagos-parks; Chicago area historical areal imagery and GLO survey maps: https://www.ilglos.com/; https://clearinghouse.isgs.illinois.edu/data/imagery/1937-1947-illinois-historical-aerial-photography; https://hub-cookcountyil.opendata.arcgis.com/pages/imagery; https://cmap.illinois.gov/data/land-use/imagery-explorer/. For both study areas also see HathiTrust Digital Library https://www.hathitrust.org/; https://earthexplorer.usgs.gov/; https://ngmdb.usgs.gov/topoview/viewer/#4/40.01/-100.06

Representative Quotes from Forest Therapy Guide Interviews

Each place is unique. It is up to us as a guide to work with the land as opposed to making the land work with us.

The designer should listen to the people who live in that place and know the place and know what is important about the place.

Plants or animals live in a particular place because they all have different energy, they embody different archetypes of the land and the way the land speaks to us is so unique depending on the place where we are connecting to it.

Summary/Highlights from Research, Planning, and Design Literature
At broad regional scales, landscape character assessments identify places that share similar physical, biological, and cultural patterns and features [57,58]. Applied at the site scale, these assessments help to identify geographical areas with characteristics that express a unique sense of place that is often more readily perceived and valued by local residents and visitors [60]. Biographical approaches to landscape character assessments help to identify the complexities of how a site's accumulated history shapes current patterns, features, and uses [221,222]. Storymaps and related deep mapping approaches can help to visualize and interpret these historical layers [223,224], adding meaning and value to people's landscape experiences and providing useful tools to planners and others concerned with placemaking and placekeeping [50,51].

2. Beauty

Definition
The variety, vividness, and/or uniqueness of a site's existing landscape character patterns and features that together contribute to its aesthetic quality.

Evaluation Procedures
Provide a summary narrative describing the physical (landform, water, rock/soil), biological (vegetation, wildlife), and/or cultural (heritage, land use) patterns and features in terms of their variety-diversity and vividness-prominence on the site, highlighting any unique or special features. Generally, sites high in elements of variety, vividness, and/or uniqueness are high in beauty.

Evaluation Rating
low–moderate–high

Additional Explanatory Notes
The focus here tends to be on the beauty of the overall landscape patterns at the site level, though in some cases (e.g., lake and river sites) it can be related to the vividness or

prominence of specific features (e.g., waterfalls, rock outcrops). Visual (scenic) beauty is usually the dominant sensory modality at the site level, but for some sites, especially those with prominent water bodies and features, sounds, motions, and other sensory modalities may be significant considerations in the evaluation.

It is important to note that the characteristics of some sites such as prairies or cultural landscapes may lack traditional scenic qualities in terms of broad scale variety in vegetation, water features, and landform but may have outstanding qualities for their character type (e.g., a high diversity of native plant and animal species, well-maintained historic gardens or buildings). Here one should highlight how a site's high integrity contributes to and can help communicate an "ecological aesthetic" or "cultural aesthetic" to the trail visitor. This makes the evaluation less of an exercise in seeking which sites are the most beautiful than what is the special or unique beauty of each site.

Data Sources Used

Field study notes and photography (trail level), USGS topographic maps (10 ft contour elevation) and county Lidar (1 – 2 ft contour), Google Earth satellite imagery (color, seasonal), site-specific reports and website information.

Data Sources Key URL links used

GIS Land Information Links: for Chicago sites, Cook Co has 1 ft contour interval maps at https://maps.cookcountyil.gov/cookviewer/; for the Northwoods study sites each county has ~ 2020 high resolution imagery and other land information records including (for most counties) 2 ft contour intervals: Florence Co WI: https://www.florencecountywi.com/departments/?department=b2d111e3ecff&subdepartment=f8196dfe2326; Forest Co WI: https://beacon.schneidercorp.com/Application.aspx?App=ForestCountyWI&PageType=Map; Oneida Co WI: https://www.oneidacountywi.gov/departments/li/land-records-system/; https://gis.co.oneida.wi.us/gismapping/; Dickinson Co MI: https://www.dickinsoncountymi.gov/government/county_departments/equalization.php; Iron County MI: https://ironmi.com/

Representative Quotes from Forest Therapy Guide Interviews

I think it is nice to have a diversity of elements.

Diversity contributes to a richer multisensory experience (e.g., morning symphony of birdsong, many flowers of diverse colors and fragrances bring a diversity of pollinators;

different trees with different leaves bring different visuals, sounds, feel of wind on your skin, etc.).

Topography is important—hilly to provide interest but not difficult hilly to negotiate. Flat and boring is not great.

For some people water is very important, the blue spaces.

Nature is our nature, if we can connect people with the heritage and tradition, and with some introduction to the Indigenous people from here and these plants and animals were their sustenance and in some cases still are.

Summary/Highlights from Research, Planning, and Design Literature
Diversity (variety, complexity) is a central landscape dimension across many expert and preference-based visual quality assessments, especially at larger scales of concern [59,61]. This especially relates to topographic and vegetation variety across a site, though in forest landscapes changes in vegetation cover relate highly to natural appearance [62,63]. Key landscape features such as water bodies, rock landforms, and historic buildings are particularly important when they are defining elements of a site in terms of their prominence or vividness [64,65].

3. Integrity

Definition
The condition or intactness of a site's desired landscape character patterns and features that together contribute to its ecological, recreational, and/or cultural-historic quality.

Evaluation Procedures
Provide a summary narrative describing the ecological (physical-biological-environmental), recreational (facility, scenic), and/or cultural (built environment, landscape) patterns and features in terms of their condition (level of maintenance, degree of degradation) or intactness (wholeness-fragmentation, pristineness, biodiversity). Sites in high condition and/or intactness are generally of high integrity.

Evaluation Rating
low-moderate-high

Additional Explanatory Notes

For wildland sites and designated natural areas the focus tends to be on the ecological quality of a site's vegetation and animal life (e.g., native biodiversity, historical vegetation species, natural community types, ecological structure and function), while other physical and environmental aspects of site condition or intactness (e.g., water quality, streambank erosion) are important across all types of settings and opportunities. For park and forest sites managed for multiple uses, the condition of recreational facilities and "scenic integrity" is especially important, as degraded infrastructure and visually impacted viewsheds can disrupt the continuity of recreational experiences. And for some sites, particularly in rural and urban settings, built features and artefacts of the cultural landscape can play important parts in a site's unique image and sense of place, enhancing connections between people and the natural environment. The geographic focus of concern should be limited to the site proper and not the broader landscape beyond (but perhaps still within the viewshed of) the site; these aspects are dealt with as part of the tranquility assessment (e.g., visual intrusions).

Additional Explanatory Notes

It should be noted that ongoing restoration activity aimed at maintaining and enhancing ecological integrity can at times appear disruptive and degrade from the intactness of a site and one's experience of it, as if one were in a construction zone. Examples include signs of tree removals (stumps, brushpiles), blackened areas from recent burns, fencing to protect vegetation from deer browsing, and flags noting plantings or herbicide spraying. How does one reconcile this apparent incongruity? Active management is necessary to maintain and enhance integrity, and hiding activity or conducting an immaculate cleanup could lessen the authenticity of experiencing integrity as a dynamic process. In such cases one should report and date what activities are present and to what degree so that those selecting sites for forest bathing know what they might experience or how guides might interpret them for their participants. This information could also be included in the section on environmental learning and stewardship.

Data Sources Used

Field study notes and photography (trail level), information on historical vegetation types and patterns (historical survey data and contemporary syntheses at site and regional scales), current site planning and management information and data from reports and websites, cultural and archaeological studies and assessments, historical and contemporary aerial and satellite imagery.

Data Sources Key URL links used

Historical vegetation: IL: https://chicago-region-trees-initiative-mortonarb.hub.arcgis.com/apps/a250fd78e414497baa6c72e4746c7b48/explore (see also paper and map by Hanson 1981); WI: https://data-wi-dnr.opendata.arcgis.com/datasets/wi-dnr::original-vegetation-polygons/explore?location=46.004788%2C-88.552309%2C14.95 (Codes from Finley Veg of WI https://www.arcgis.com/home/item.html?id=9f473ce5c46e447e889c7458d6f54913); MI: https://mnfi.anr.msu.edu/resources/vegetation-circa-1800

Current natural areas information: IL Nature Preserves Chicago area: https://dnr.illinois.gov/inpc/directory/naturepreservearea2.html; Cook County Forest Preserves: https://fpdcc.com/; https://fpdcc.com/nature/illinois-nature-preserves/; North Branch Restoration Project: https://northbranchrestoration.org/; Chicago Park District Natural Areas: https://www.chicagoparkdistrict.com/; https://www.chicagoparkdistrict.com/natural-areas; WI State Natural Areas: https://dnr.wisconsin.gov/topic/StateNaturalAreas

Representative Quotes from Forest Therapy Guide Interviews

I guess the more diverse ecologically a space is, the more opportunity people have to experience different reflections of themselves…The greater amount of ecological diversity the greater the palate of psychological reflection can become.

Biodiversity is important, where you have a sense of community, a community of differences.

Most suitable conditions for optimal experiences would be an abundance (and diversity) of trees, other plants, and wildlife. More pristine conditions would be better.

Summary/Highlights from Research, Planning, and Design Literature

A site has high ecological integrity when its native species composition and community structure occur, function, and are sustained within their natural range of variability [66,67]. High recreational and scenic integrity occur when sites maintain their desired landscape character in terms of natural appearance and function without noticeable degradation due to recreation use levels and types of use or competing land uses and practices [59,68]. High cultural integrity is reflected by the visible presence and maintenance of valued and/or historically significant landscape characteristics such as buildings, small scale features, and vegetation patterns [69,70].

4. Tranquility

Definition
The setting, sensory, social, and environmental qualities of or intrusions into a site's peacefulness and isolation that together promote psychological and physiological comfort and minimize distractions.

Evaluation Procedures
Synthesizing the information from the sub-criteria below, provide a summary narrative describing the setting (size, isolation), sensory (visual-sound-other), social (number and type of trail and adjacent users/uses), and environmental (physical, biotic) conditions present that intrude upon the site's tranquility. For factors such as sound and social use levels that may have considerable temporal variability, estimate average conditions for use in overall tranquility evaluation ratings. Sites isolated from competing uses with low levels or intrusions are generally considered high in tranquility.

Evaluation Rating
low-moderate-high

Additional Explanatory Notes
While tranquility as discussed in the research literature is a function of both positive and negative sensory, social, and environmental factors, it is applied here largely in terms of intrusions or lack thereof. Positive factors that can contribute to tranquility such as bird sounds or the presence of big trees are inventoried in the context of other dimensions. As applied to sites in this assessment, sound and social factors described below often carried the weight of the tranquility evaluation, although when applied elsewhere the other factors may have overriding importance. Also, because of the substantial differences in sound and social use levels encountered in the Northwoods sites compared to the Chicago sites, somewhat different standards of tranquility were adopted in establishing low–moderate–high ratings. In most applications, study sites considered in a comparative assessment would not span such a large range of sound and social use levels.

Summary/Highlights from Research, Planning, and Design Literature
Separation from distraction is a key element in restorative environments [71]. Participatory mapping and other techniques can be helpful in identifying tranquil areas and positive and negative attributes of tranquility [72–74].

4.1. Setting

Definition
The size of the site on which the trail is located and its degree of isolation from potentially competing adjacent uses.

Evaluation Procedures
If site size information is not available, measure the size of the site using the polygon feature in the ruler tool of Google Earth (or similar) and characterize the adjacent land uses surrounding the site in terms of their compatibility. If the site has no formal boundaries within its larger context, draw a polygon defining the immediate area around the trail or trail network, following natural (e.g., vegetation water) or human-defined (e.g., roads) boundaries or transitions.

Additional Explanatory Notes
Small sites or narrow linear corridors that juxtapose significantly different land covers and uses may impact people's ability to achieve an immersive experience even though there are no major visual or sound intrusions. Examples include a canopied rail-trail corridor crossing open agricultural lands and a small urban park natural area surrounded by open park lawns or ballfields.

Data Sources Used
Site-specific information, Google Earth (or similar applications) used to plot trails and site boundaries (image overlay).

Representative Quotes from Forest Therapy Guide Interviews
It is a nice size and it has multiple ecosystems in one location.

It has to be somewhat secluded.

The sense of privacy is important.

Summary/Highlights from Research, Planning, and Design Literature
Generally speaking, larger sites provide better isolation from intrusive elements and also different options for "getting away" and into nature for having a restorative experience [71, 77,78].

4.2. Visual

Definition
Visual impacts of development seen from trails on the site.

Evaluation Procedures
Describe type, distance (suggested description of distance zones: Immediate Foreground or IFG 0–100 ft (0 – 30 m), Foreground or FG 100 ft – 1/4 mi (30 m – 0.4 km), Middleground or MG 1/4 – 1 mi (0.4–1.6 km), Background or BG > 1 mi (> 1.6 km)), magnitude/scale, and compatibility of intrusive visible development including roads, buildings, and land uses. Note locations along trails where visual impacts are experienced, making particular note of key points or stretches.

Additional Explanatory Notes
For some sites, there can be substantial differences in visibility due to leaf-on/leaf-off conditions, vegetation type, topography, etc. Distance zone intervals will vary depending on landscape type and region of study.

Data Sources Used
Field study and photography, Google Earth to assist in estimating distance zones.

Representative Quotes from Forest Therapy Guide Interviews
Avoiding powerlines would be nice, sometimes you can't avoid them.

Not too much evidence of human use and not too much distraction from other users.

… *Avoid manmade elements.*

Summary/Highlights from Research, Planning, and Design Literature
Visual impact assessment approaches consider the magnitude (scale and distance), noticeability (color contrast, reflections, lights, etc.), view duration, and view/viewer sensitivity of visible features or development considered to be intrusive [79,80]. In natural and naturally-appearing landscapes, built elements such as powerlines, buildings, and transportation corridors are often considered intrusive [81], as can forest harvest activities such as clearcutting and the appearance of slash [82]. Vegetative pattern and screening, contrast matching or camouflage, and other techniques can be used to reduce the visual impacts of development [83].

4.3. Sound

Definition
Noise or other negative sound impacts heard from trails on the site.

Evaluation Procedures
Describe the types and magnitude of sound disturbances including roads, buildings, and land uses and activities. These sources are usually external to the site but may also come from other site uses and activities. For most sites, road noise is the major intrusion and one should measure (Google Earth) and report the shortest and furthest distance of trails on the site from roadways and report roadway type and traffic volume (Department of Transportation average annual daily traffic volume or AADT (suggested description of AADT levels: Very Low (VL) = under 200 AADT; Low (L) = 200–2000; Moderate (M) = 2001–10,000; High (H) = 10,001–30,000; Very High (VH) > 30,000). For sites near busy airports note whether trails are in the flightline of takeoffs and landings (report average elevation and decibel levels if available). Summarize primary sound impacts and provide a composite anthropogenic sound impact rating (Soundscore (see data sources below) uses these interval levels and descriptions: 96–100 Very Quiet, 91–95 Quiet, 86–90 Very Calm, 81–85 Calm, 76–80 Active, 71–75 Very Active, 66–70 Busy, 61–65 Very Busy, 56–60 Loud, 50–55 Very Loud).

Additional Explanatory Notes
For urban sites it may be useful to distinguish four types of noise: overhead noise from planes, roadway noise, noise from adjacent or nearby land uses, and noise from other trail users or site visitors. Roadway noise is probably the most critical and one should consider proximity to roads, types of roads, and volume (flow) and timing of traffic. Low volume intermittent noise may not be too much of a problem, and constant traffic that is far off and creates a din may also not be a major distraction. But for some sites, the low tranquility of a close, high-volume road can overwhelm the restorative of experience of sites that are otherwise high in beauty, integrity, accessibility, and related trail attributes. For such sites, noise levels may vary considerably as a function of time of day, day of week, and other factors, and some note of optimal windows of quietest times (e.g. Sunday mornings) would be helpful.

Data Sources Used
For distance to roads, Google Earth. For traffic volume, state Department of Transportation websites. For transportation related noise, see the National Transportation Noise Map. See

also other transportation-specific (e.g., aircraft overflights) and composite anthropogenic sound impact websites and apps.

Data Sources Key URL links used

AADT volume: IL https://www.gettingaroundillinois.com/Traffic%20Counts/index.html; WI https://wisconsindot.gov/pages/projects/data-plan/traf-counts/default.aspx; MI https://mdot.maps.arcgis.com/apps/webappviewer/index.html?id=50dd462048fd4971998567699698f285. Airport flight altitude information for Chicago study region is available at: https://webtrak.emsbk.com/cda. Transportation noise information is available at: https://maps.dot.gov/BTS/NationalTransportationNoiseMap/. Composite anthropogenic sound impact ratings for selected urban areas are available at: https://howloud.com/soundscore/.

Representative Quotes from Forest Therapy Guide Interviews

For me most important is the quietude, that is my biggest one. That's most important.

I think one of the biggest things that can improve the experience is lack of noise pollution. I think that can be distracting and detrimental to the experience. So, environments that provide more of a pristine sound experience, and have mostly natural sounds can be beneficial.

…Airplane noise can be very distracting. Highway noise can also bother people.

Summary/Highlights from Research, Planning, and Design Literature

Anthropogenic sounds are usually perceived as intrusive and a major disruption to feelings of tranquility and restorativeness in urban and natural settings [84–86]. Noise mapping and soundwalk techniques can help identify areas of tranquility [87,88] and policies and best practices can help protect them [89,90].

4.4. Other Sensory Intrusions

Definition

Offensive odors or other sensory intrusions experienced from trails on the site.

Evaluation Procedures

Describe the type and magnitude of any other potential intrusions such as smells from surface waters, farm fields, or other ambient or point sources of offensive odors, dust or smoke, light, etc. Note any temporal variation.

Additional Explanatory Notes
Other sensory intrusions were rarely encountered for the sites studied in this assessment, but one place where they could have been were on bike trails that shared the road or trail with motor vehicles, where dry conditions on dirt/gravel surfaces can kick up considerable dust. Light intrusion could be a factor for dark sky experiences.

Data Sources Used
Field study and site-specific information.

Representative Quotes from Forest Therapy Guide Interviews
A visible barrier from distracting lights.

Summary/Highlights from Research, Planning, and Design Literature
Unpleasant odors have long been at the forefront of land use nuisance regulations, and research on measuring and mapping odors has grown in recent years [91–93]. Light and air pollution can also impact tranquility, and studies of these distractions aim at the protection of dark skies and visibility [94,95].

4.5. Social

Definition
Use level and compatibility of other users on trails or adjacent use areas.

Evaluation Procedures
Estimate the average range in trail use levels in terms of numbers of parties encountered (e.g., very low = 0–1, low = 2–4, moderate = 5–10, high = 11–50, very high = > 50 or 11–50 plus large groups of 10 or more). Note user type and compatibility, including those in adjacent use areas that might affect privacy/tranquility. Note temporal/spatial patterns of use for potential for off-peak visits.

Additional Explanatory Notes
The nature of the setting or context can be an important filter governing people's feelings of isolation and privacy, where higher use levels may be more tolerable in urban settings than in wildland ones. Environmental (e.g., vegetation screening, water sounds from waves or waterfalls) and individual and/or cultural factors can also modify expectations.

Data Sources Used
Field study.

Representative Quotes from Forest Therapy Guide Interviews
The first one I have to say is a trail that is not bombarded with a bazillion trail users. A quieter space, it doesn't have to be totally still, that helps, but just someplace where we are not bombarded by runners, hikers and mountain bikers and dog walkers.

…there won't be a lot of other people on the trail that make the person feel intimidated, so that this person can have a one-on-one experience with nature.

I would try to create the path in its own space away from bike trails, equestrians, and other uses where you have to negotiate around other outside stimulus.

Summary/Highlights from Research, Planning, and Design Literature
Privacy and solitude are key components of restorative nature experiences [96,97]. Restorative nature experiences are more likely to occur when one is alone or with a close friend [98,99]. The presence of other individuals and groups, especially those with recreational or non-recreational goals that differ markedly, can result in psychological conflict [100–102].

4.6. Environmental
Physical and biotic factors that can impact comfort and physical safety.

Evaluation Procedures
Record everyday or seasonal environmental conditions that could be challenging and induce physical or emotional stress to some users, e.g., wet/muddy trail areas, steep grades, narrow trails, rocks/roots, downed trees, lack of a formal trail, insects, poisonous plants, etc.

Additional Explanatory Notes
While environmental factors such as mosquitos or poison ivy can certainly impact psychological and physical comfort and are important to note for sites and trails where they occur, in practice they were not given much weight in the composite tranquility rating compared to sound and social factors. Trail-related environmental factors such as grade and surface were more explicitly considered under Design and Construction below.

Data Sources Used
Field study.

Representative Quotes from Forest Therapy Guide Interviews
We want to minimize hazards along the trail. I identify types of snakes, watching for rocks and logs, poison oak and poison ivy, potential for ticks. I'm hoping to minimize encounters with hazards as much as possible.

…everyone should feel comfortable…If there are bugs bothering them, they are going to clam up.

…adversity from the environmental natural elements and other species…Avoid hazards. Stay away from steep terrain, ledges, drop-offs.

Summary/Highlights from Research, Planning, and Design Literature
Physical obstacles, discomfort from the elements, and environmental phobias can impact individual's feelings of tranquility and connectedness to nature [103–105]. Managers and forest therapy guides can work to minimize risks and prepare visitors to cope with these issues to maximize their nature experiences [106,107].

5. Accessibility

Definition
The ability and ease with which people can get to and use a site, including its proximity from home, available trailhead facilities, user fees, and range of trail options and degrees of difficulty.

Evaluation Procedures
Synthesizing the information below, provide a summary narrative describing the proximity (distance, travel time), available facilities (parking, toilet, other), user fees (parking, trail use, guide), and trail options (number, type, and range of difficulty) that facilitate or impede access and use of the site. Generally, sites that are nearby, have basic trailhead facilities, are reasonably priced, and have a range of trail options are considered good candidates for forest therapy.

Evaluation Rating
low–moderate–high

Additional Explanatory Notes
As applied in this assessment, most of the sites were visited without guides or other fees and were selected for inclusion based on a 1/2 hr. drive time maximum proximity threshold, so these factors weighed somewhat less on the accessibility evaluation than facilities and trail options. It is also important to note that while site level accessibility considerations in part determine people's ability to use individual trails, trail level accessibility are further determined through specific trail design and construction factors such as trail length, slope, and accessibility barriers evaluated below.

Summary/Highlights from Research, Planning, and Design Literature
Highly accessible sites offer a range of opportunities within easy and affordable reach to a broad-based clientele [75,76].

5.1. Proximity

Definition
Nearness to site trailhead from some logical or popular point of origin.

Evaluation Procedures
Using Google Maps or a similar procedure, determine address or geocalculate the distance and/or time (driving, walking/biking, public transportation as is most relevant) to the main trailhead of the site from a chosen point of origin. Depending upon the location, choose a maximum threshold time or distance that seems reasonable (i.e., doesn't overly fatigue the visitor in getting to or returning from the site) for use in establishing an evaluation rating for site accessibility.

Additional Explanatory Notes
The chosen point of origin could be from one's home for an individual, a convenient or logical meeting place for departure for a group outing, or a city or town center for a regional assessment. As applied in this assessment, a popular, conveniently located public nature center was selected as point of origin for each of the two study areas, and a 1/2 hr. maximum drive time threshold was chosen for the selection of most trails from those origin points, recognizing that urban travel distances can be shorter than rural ones for the same amount of time. For the rural (Northwoods) study area, rough road conditions were noted in the summary narrative and incorporated as part of the site accessibility evaluation as steep, wet, or unmaintained dirt roads can lessen access opportunities for visitors with unsuitable vehicles.

Data Sources Used
Google Maps, field assessment, site-specific information.

Representative Quotes from Forest Therapy Guide Interviews
The trail needs to be reasonably accessible to where people live so they don't have to drive too far. …For a lot of people, it is an edge to come out on one of these walks and they don't want to be too far away. If they are with a group of people they don't know, proximity is important.

…not far from where people live. Develop trails close to where people live for easier access.

Summary/Highlights from Research, Planning, and Design Literature
Nearby nature opportunities figure highly in research on restorative urban environments [4,108], while proximity is a key factor in establishing accessibility standards in local and regional park and open space assessments [109–110].

5.2. Trailhead Facilities

Definition
Key support amenities that are present at or near the site trailhead.

Evaluation Procedures
List and briefly describe parking, toilet, drinking water, and other support facilities available at or within close access to the trailhead. While the level of trailhead facility development is highly site dependent, most trailheads should at least have a parking area and, ideally, a portable toilet or outhouse. In more developed sites and urban settings, restrooms with flush toilets, drinking water, and other facilities are expected by many visitors.

Additional Explanatory Notes
Include any daily time or seasonal closures or limitations, such as whether parking and access roads are plowed for winter snow conditions.

Data Sources Used
Field assessment, site-specific information.

Representative Quotes from Forest Therapy Guide Interviews
Parking and a bathroom are two major things. A visitor center with a bathroom is nice. Adequate parking and accessibility are important. …It has to have parking, bathrooms.

Accessibility and access to restrooms, and parking are critical for participants. A nature center or ranger station is secondary, predominantly the restroom is key.

It is nice to have facilities available at the start where they can use the bathroom and get water, it helps people ease into the experience.

Summary/Highlights from Research, Planning, and Design Literature
A well-maintained toilet facility is often a minimum site requirement for many visitors [111,112], and higher levels of support facilities are often expected at sites that are more urban in nature and among some user groups such as older adults [113].

5.3. User Fees

Definition
Costs or other requirements such as membership that may pose as barriers to entry and use of a site and its trails.

Evaluation Procedures
Describe any parking, day use, or other entry fees needed to access the trailhead or individual trails on the site, in addition to guide or tour fees if applicable. Nominal costs are often acceptable but steep costs can impact accessibility.

Additional Explanatory Notes
Make note of fee waivers or other exceptions such as free days and early/late entry hours, discounts for different groups or individuals, or walk-in or alternate means of access to the site that help reduce financial burdens and barriers. While most of the sites in this assessment were on local, county, state, and federal public lands, some private sites may have access restrictions such as membership that limit access and should be noted.

Data Sources Used
Field assessment, site-specific information.

Representative Quotes from Forest Therapy Guide Interviews
I do donate 30% back, like to the Audubon, when I lead groups on their lands. I charge $30/person. I also ask groups what their budgets are.

We need to have trails for all people, especially underserved groups and groups with differing abilities.

The executive director is excited that I am bringing people here. She isn't charging me anything, she doesn't want anything, she says "please talk about how awesome this place is and invite them to come back."

Summary/Highlights from Research, Planning, and Design Literature
While modest user fees are often accepted by visitors as a means of sustaining quality outdoor recreation opportunities, they can also reduce site visitation and can disproportionately affect individuals with low incomes [114,115]. The impact of fees may be more acute for urban sites and for some types of outdoor recreation activities [116,117]. As a nature-based outdoor activity, forest therapy is often promoted as a cost-effective approach to physical and mental health and wellbeing when compared to other types of therapy [118].

5.4. Trail and Accessibility Options

Definition
The number, type, and range of difficulty of trails available at a site.

Evaluation Procedures
Using design and construction information on individual trails described in the sections below, provide a brief narrative summarizing the number, type, and range of difficulty (length, steepness, or other potential challenges) of all trails available within the site. While a single trail geared to wheelchair users may be considered highly accessible, sites with multiple trails, different trail types, and a range of levels of challenge may be better suited to serving a diversity of users and interests.

Additional Explanatory Notes
Trails that are wheelchair accessible in part or whole are important to note, as well as barriers or challenges such as rocks and roots in the trailbed that might make otherwise "easy" trails more difficult for some visitors. For paddle trails, mention muddy landings, water current, waves, or large potentially windy expanses of water that could pose challenges. On the positive side, also highlight assets like stretches of moss or pine needle covered trail that facilitate uses such as barefoot walking. While this information is also covered in the trail-level factors below, having it as part of the site level summary will help

guides and individuals in assessing the overall accessibility of a site in terms of the range of options available.

Data Sources Used
Field assessment and trail level measures, site-specific information.

Representative Quotes from Forest Therapy Guide Interviews
Opportunities for people with different accessibility levels are really important. …I can imagine, the same way there are nature trails with different accessibility levels, there might be forest therapy trails, some that are more accessible and some that are more wild.

…I think we should think about the Recreation Opportunity Spectrum (ROS) and how forest bathing fits within ROS and how we can apply what we know from ROS.

The fun thing is broadening the scope of what a trail is to include kayaking and mountain biking, etc.

Summary/Highlights from Research, Planning, and Design Literature
The Recreation Opportunity Spectrum or ROS is a framework for outdoor recreation planning, design, and management that recognizes how different types of forest settings from primitive to developed can provide desired experiences to people in terms of challenge, risk, isolation, and other factors [79,119]. While the framework was developed in the context of U.S. national forests, it has been applied in the context of identifying trail opportunities and preferences in a range of wildland and urban sites [113] and incorporated into accessibility guidelines [120].

Part II. Trail Level Criteria

Part II A. Design and Construction

6. Ease of Travel

Definition
Trail design parameters (trailhead distance, length, surface, width, slope) and obstacles (physical accessibility barriers) that contribute to the ease or difficulty of trail use.

Evaluation Procedures
Synthesizing the information below, make an evaluative rating incorporating distance to trailhead, length, surface, width, slope, and accessibility barriers to assess the ease of travel on the trail. Generally short trails readily accessible from trailheads that follow easy grades with comfortable widths and stable natural surfaces with few obstructions are preferred.

Evaluation Rating
low–moderate–high

Additional Explanatory Notes
Most trail accessibility guides or trail class ratings do not include trail length in their criteria, but for this assessment length was considered an important element of what makes for an easeful forest therapy trail experience. "Trail Class" is an established scale for designating trails along a five-category continuum from minimally to fully developed and among other factors also include trail design parameters of surface, scale, slope, and obstructions that similarly indicate ease of travel. While Trail Class was also rated in this assessment, a number of trails included in the sample had parameters that did not fall neatly into the Trail Class matrix (i.e., narrow trails on flat slopes with minimal accessibility barriers that were rated high for ease of travel).

6.1. Distance to Trailhead

Definition
The distance of travel between the starting point (e.g., parking lot) to where the actual forest therapy trail experience begins.

Evaluation Procedures
Measure in Google Earth from starting point (e.g., parking lot) to where the actual forest therapy trail experience begins. Some trails may require a walk along a main trail, road, or less desirable trail segment before in order to reach the starting point. Generally, trail layouts with short trailhead distances are desirable.

Data Sources Used
Field assessment, Google Earth, site-specific information.

Representative Quotes from Forest Therapy Guide Interviews

Access to the trail is important. ...It is better in the beginning for less than 5 minutes to the start.

So, there is even some consideration in designing a trail so you can have a little bit of exercise that precedes it, people will then naturally slow down more if they are tired versus if they are filled with energy and you want them to do a self-guided trail, it can be hard to get people to slow down.

Summary/Highlights from Research, Planning, and Design Literature

Trailheads should be located at or near parking areas to allow for ease of access. Easy trail access equates with higher trail preference and use [121]. Access routes between starting points and trailheads should be clearly marked, safe, and accessible [122,123].

6.2. Length

Definition

The distance along the trail or trail segment from the trailhead and back.

Evaluation Procedures

Trail length can be measured using Google Earth or similar programs, in the field with a phone app such as Avenza, or taken from site-specific information such as websites and brochures. Most trails in this assessment were not designed for forest therapy, and for sites with longer trails and trail networks it may be desirable to map a route that uses a portion of a trail or connected segments of two or more trails of appropriate length for a forest therapy trail visit and develop a separate evaluation for that particular configuration. For linear trails or trails that can be accessed only after traveling along another trail first (e.g., stacked loop trails), measure and record the complete travel distance needed to return to the trailhead. Optimal length can vary (see additional explanatory notes), but foot trails in the range of 0.5–1.0 mi (1–2 km) are often considered as best suited for forest therapy.

Additional Explanatory Notes

The optimal length of a trail for forest therapy will vary depending on trail type, surface, grade, and other design and construction factors, but here are some suggested guidelines: For foot trails, optimal length is usually less than 1 mi (1.6 km) and often under .5 mi (< 1 km) for guided groups, while trails longer than 3 mi (5 km) are fatiguing for many visitors. Optimal length for paddle trails will vary by type of water; on calm flatwater lakes and pond 0.5–1.5 mi (0.8–2.4 km) is often optimal, but on rivers with current an optimal length might

be from 1–3 mi (1.6–4.8 km) or more. For bike trails, a suggested optimal length range is between 3 and 7 mi (4.8–11.3 km) but will vary considerably depending on surface, grade, and other trail characteristics.

Data Sources Used
Field assessment, Google Earth, site-specific information.

Representative Quotes from Forest Therapy Guide Interviews
Length isn't that big a deal.

We don't cover a lot of territory. I think in the beginning I felt I really had to do so many invitations, cover lots of space, but as time has passed, I don't feel that way. I don't need to cover a lot of ground. Spokes going out from a central point can work.

Maybe a quarter of a mile…around a quarter of a mile or not longer than a quarter of a mile would be good.

Summary/Highlights from Research, Planning, and Design Literature
Trails should be long enough to provide a satisfying experience without being so long that they induce fatigue. Length can vary, but trails in the range of 0.5–1.0 mi (1–2 km) are best suited for nature study and forest therapy [124,125]. Accessibility guidelines state that length should be at least 500 ft (150 m) to the first extreme environmental barrier and include interesting natural, cultural, or historic features that provide for a meaningful experience [120].

6.3. Surface

Definition
The composition or material of the trailbed tread surface.

Evaluation Procedures
Field observation; in the case of multiple trail surfaces, record the different surface types and dominant surface(s). Suggested terms include dirt (native soil, compacted by use or design and sometimes amended with soil binders such as clay to increase stability and reduce erosion), grass (native and introduced vegetation, mown or compacted by use), woodchips, sawdust, crushed gravel (limestone screenings), gravel, asphalt, concrete, stone, brick, wood or plastic wood (boardwalk material), or other introduced or naturally-occurring material (e.g., moss, pine needles) that form a significant part of the tread along

the route. Natural materials are usually preferred for forest therapy trails so long as they provide a firm and stable surface and resist erosion from weathering and use.

Additional Explanatory Notes
For paddle trails it can be useful to record river or lake bottom properties of shorelines areas (e.g., % sand, marl, muck, gravel, rubble, boulders) to help understand the potential for using the waters and shore areas for swimming, soaking or wading at sitspots, etc.

Data Sources Used
Field assessment.

Representative Quotes from Forest Therapy Guide Interviews
It's nice to have a variety of paved and stone dust or boardwalk surfaces that wheelchairs can go on.

I like to see accessibility for disabled people with walkers or wheelchairs, paved or packed earth... Having a variety along the trail, paved, to packed ground to mulch, grass and gravel are nice...the variation along the trail is nice.....area with grass for people to stand barefoot."

Summary/Highlights from Research, Planning, and Design Literature
Natural/local materials are preferred (i.e., forest soils) so long as they provide a firm and stable surface for walking that can be sustained for the expected environmental conditions and level of use [122,126,127]. A soft but firm surface (e.g., pine needles, moss, leaves in fall) along edges or portions of the trail tread may be desirable to enhance sensory experience, especially for barefoot walking [128]. In some locations or along some trail segments where wet or steep conditions or high use would lead to erosion or difficult walking, trail "hardening" with imported materials may be needed. This could range from gravel amendment of soil to wood chips to gravel (limestone screenings) paving, to more intensive treatments such as wooden or plastic wood boardwalks in very wet areas, to asphalt or concrete in high use, "urban" conditions [129].

6.4. Width

Definition
The width of the trailbed tread surface or the right-of-way if no clear trailbed is apparent (i.e., mown grass routes, snow-covered corridors).

Evaluation Procedures
In the field, visually estimate or set a tape measure perpendicular to trail tread at a number of locations along the length of the trail. Trail routes in this assessment often had variable widths so record minimum, maximum, and modal trail width if necessary. Some route segments included in this assessment did not have formal trails, such as those along riverbanks and lakeshores, and other places where established foot or deer trails disappeared or were obscured by vegetation required "bushwhacking" (definition: hiking off-trail through the bush, where a cleared path doesn't exist. Bushwhacking might require hikers to weave their way through overgrown areas and branches.) In these cases, trail width was recorded at 0. While trail width preferences vary by trail use, class, and other factors (see additional explanatory notes), generally for foot trails in low to moderate use sites, tread widths of 3–6 feet (1–2 m) with active ROW vegetative management allow users an immersive experience with some room for other users to pass.

Additional Explanatory Notes
The optimal width of a trail for forest therapy will vary depending on trail use (foot vs bike), group type and size (guided vs self-guided), trail use level and development class (low use primitive foot trails to moderate use urban foot and bike trails), and other factors, but here are some suggested guidelines: low use foot trails, 1.5–3 ft (0.5–1 m), moderate use foot trails 3–6 ft (1–2 m), moderate use trails for group use and shared foot and bike trails 8–12 ft (2.4–3.7 m), foot or bike trails that share the corridor with very low use motor vehicles (e.g., ATV trails and forest roads) 10–15 ft (3–4.5 m). Foot and bike trails that use corridors managed for horses, motor vehicles, or seasonal use such as cross-country and hunter walking trails are often wider (> 15 ft or 4.5 m) than what is optimal, while trails in some backwoods and ecological areas often follow animal trails and are narrower (< 1 ft or .3 m and sometimes disappear altogether) than optimal but can still provide an acceptable corridors to access otherwise good sites for individual or small group forest therapy, particularly for those with moderate or higher skill levels. For paddle trails, small streams as narrow as 10–15 ft (3 – 4.5 m) in width can be engaging if water levels are high enough, while rivers wider than 150 ft (45 m) can be, too, if currents are not too strong and they have interesting shorelines. But generally, streams and rivers between these widths are optimal. Small ponds and lakes 10–100 ac (4–40 ha) with complex shorelines are preferable to large lakes with simple shorelines.

Data Sources Used
Field assessment.

Representative Quotes from Forest Therapy Guide Interviews

The width of the trail is important too. You want people to be able to be in a state of mindfulness so anytime they have to concentrate on their footing takes away from that and is distracting.

Wide enough for people to accompany someone, say 3–5 feet wide, makes it easier for people passing by the group in either direction. It makes it more comfortable for everyone.

We keep the trails narrow so they resemble deer trails. We have a separate trail for a wheelchair accessible trail. It is much better to have two trails than just one so that people who can walk normally can have a narrow trail so that people who can walk have the experience of close-by vegetation that they can brush up against.

Summary/Highlights from Research, Planning, and Design Literature

While narrower trails allow visitors opportunities to experience nature more intimately [71], for other users trails wider than 10 ft (3 m) tend to be used more by visitors [121]. Standards for accessible trails specify a clear tread width of at least 3 feet (1 m) [120,127].

6.5. Slope

Definition

The running slope of the trail surface, specified in terms of maximum slope and average slope.

Evaluation Procedures

Plot trail in Google Earth based upon trail maps, Avenza smartphone app, or other data, and use the elevation profile data to record maximum slope and average slope. If necessary, adjust values based on field assessment and topographic maps. Suggested slope terms are level or flat, gentle, moderate, steep, and very steep. For river slopes, gentle < 1%, moderate 1–4%, steep > 4%. Foot and bike trails that are nearly level with some gentle grades for variation are suitable for most users and trail uses. For paddle trails, flatwater lakes and rivers with low gradients and gentle currents are best.

Additional Explanatory Notes

In a few instances in this assessment, Google Earth values did not seem accurate compared to actual field experience. This may be due to inaccurate trail plotting or inaccuracies in the elevation data reported by Google Earth. In such cases, some adjustment to reported values may be needed using field observation aided by smartphone

trail app, and/or topographic maps. As the main goal is to include a measure of relative trail steepness as a factor in the overall ease of travel, precise numbers are less important.

Data Sources Used
Google Earth, cross-check with field assessment, smartphone trail app, and/or topographic maps.

Data Sources Key URL links used
For rivers use https://www.omnicalculator.com/math/slope-percentage and report the per mile percentage; https://streamhandbook.org/evaluating-your-property/classification/stream-slope/

Representative Quotes from Forest Therapy Guide Interviews
Fairly flat without too much up and down...

It is better not to have trails that gain a lot of elevation. People become focused on how difficult it is to walk rather than on the experience. A little bit of slow grade can be good.

Maybe the trail would be better with a bit of up and down requiring use of proprioception and vestibular senses.

It is slightly hilly but not too difficult for people with accessibility issues. Hilly enough to provide interest but not difficult hilly... Topography is important. Flat and boring is not great.

Summary/Highlights from Research, Planning, and Design Literature
Preferences are usually for an easy, level or nearly level trail to accommodate a wide variety of trail users and abilities and so that users can focus on the immersive nature experience rather than negotiating demanding, difficult terrain [64,130]. If the site location permits it, there might be a selection of trails of different grade difficulties, and accessibility criteria specify that more steeply sloped trail segments are okay if they incorporate adequate intervals for resting and spaces for passing: Running slope = 1:20 (5%), segments 1:8 (12%), no more than 30% of trail should be > 1:12 (8 1/3%); Cross slope = 1:20 or 5% [120,127].

6.6. Accessibility Barriers

Definition
The type and frequency of obstacles encountered in the trailbed tread surface and right-of-way corridor, including side and overhead height obstacles.

Evaluation Procedures
Field observation; list type and frequency of barriers or obstacles such as rocks and exposed roots in the trailbed, fallen logs, brush, side and overhead branches, primitive water body crossings (log bridges, steppingstones), wet and muddy stretches, uneven pavement, stairs, slippery surfaces, etc. For paddle trails, list frequent in-stream rocks, strong current or rapids, overhead and side branches along shore, low water periods, etc. Most trail users prefer a trail corridor with few obstructions or barriers to accessibility, and trails with long stretches of wet areas or brush should be avoided.

Data Sources Used
Field assessment.

Representative Quotes from Forest Therapy Guide Interviews
The trail needs to be accessible, both ADA accessible and universally designed for people of all physical abilities in terms of topography, inclines, declines, surfaces, and invitingness of the space, and leading people down a path.

Condition of the trail is important, not overgrown… Roots can be problematic if people are unstable on their feet.

I use one trail that is uneven, so people are walking one foot up and one foot down and it is uncomfortable.

Stone steps can be challenging. … hiking poles… might be a consideration if there are steps.

Summary/Highlights from Research, Planning, and Design Literature
Exposed rocks, tree roots, and other obstacles in the trailbed can be challenging to some users [130]. Accessibility guidelines specify that trail obstacles should be < 2 in (5 cm) and openings (i.e., gaps between boards in a boardwalk) < 1/2 in (1.25 cm) [120,127]. Sustainable trail design principles can minimize accessibility barriers by preventing erosion [131,132].

7. Attractiveness of Layout

Definition
The design configuration (alignment, route type and directionality, views, spaces, and changes) of a trail that contributes to an engaging experience.

Evaluation Procedures
Synthesizing the information below, make an evaluative rating incorporating alignment, route type and directionality, key views, spaces, and related spatial-temporal changes encountered along the trail that enhance forest therapy experiences. Look for curving to winding loop trails with a variety of views, private and group spaces, and changes along the route.

Evaluation Rating
low–moderate–high

7.1. Alignment

Definition
The horizontal (degree of straightness or curvature) and vertical (relative flatness or changes in elevation) layout or routing of a trail corridor through a site; the path it follows to connect features or destinations in the landscape with sensitivity to functional, environmental, and aesthetic considerations.

Evaluation Procedures
Along with providing an overview description of topographic and landform characteristics, summarize the dominant horizontal and vertical alignment character of the trail layout through field assessment, aided by quantitative measures of degree of route straightness or curvature (river sinuosity and/or lake development indexes) and changes in elevation (Google Earth, Avenza phone app, topographic maps). Suggested topographic/landform terminology includes flat, gently rolling, rolling, hilly, mountainous; changes in elevation expressed in feet or meters of relative relief; land and water trail horizontal alignment straight, gently curving, winding, twisty, meandering. Generally, gently curving to winding trails that traverse varied topography with some changes in grade contribute to an engaging experience.

Additional Explanatory Notes

For paddle trails stream sinuosity and lake development measures can indicate degree of shoreline curvature or complexity. For rivers, suggested sinuosity values are: 1–1.05: Straight; 1.05–1.25: Winding; 1.25–1.50: Twisty; 1.5 or more: Meandering. For lakes in this study, bias-corrected lake development index (Dbc) values (see citations below) ranged from 1.36 for a relatively round lake with a simple shoreline to 7.74 for a lagoon with a highly complex shoreline. Overall, lakes with interesting shorelines (bays, wetland areas, points, etc.) usually had values > 2.0.

Data Sources Used

Field assessment (observation and/or phone app e.g., Avenza), Google Earth, topographic maps.

Representative Quotes from Forest Therapy Guide Interviews

If I were designing the ideal forest therapy path, I would want to incorporate mystery, bends where you can't see around the corner, or maybe you see light, but you can't see where it is coming from.

Shouldn't be so linear that people will trip over themselves or have to walk too far from the group to get some space. A linear trail can be limiting, you need some openings that are not so closed in… A trail with twists and turns.

The trail itself, eliminate straight lines. They don't exist in nature. Everything should have a bit of a dimension or curve; it should be somewhat sinuous and flow through the environment like an animal trail would.

Summary/Highlights from Research, Planning, and Design Literature

Trail alignments that have curves and changes in elevation can add mystery and invite exploration more than flat, straight routes like some road and rail-trail corridors [71]. Shoreline indexes provide quantitative estimates of curvature for paddle trails [133,134]. Trail layout should balance design principles of safety (environmental sensitivity and physical safety), efficiency (directness of destination, cost of construction), playfulness (mystery, surprise), and harmony (conformance to topography, water bodies and other features) [135–137].

7.2. Route Type and Directionality

Definition
The path a trail follows from and back to the trailhead, usually described by a loop or linear configuration or some permutation of either or both.

Evaluation Procedures
Record route type and directionality using field assessment or other site information from websites, maps, or brochures. Route type is usually described by a loop or linear (there-and-back) configuration or some permutation of either (e.g., stacked loop) or both (partial loop). Some loop trails (e.g., cross-country skiing) are signed for one-way travel, and many paddle trails on rivers are considered one-way due to their currents making upstream travel difficult. Generally, loop routes are considered preferable, while one-way trails can lessen encounters and conflicts with other parties.

Additional Explanatory Notes
In addition to directionality, trail type and user restrictions can have important effects on the attractiveness of a trail for forest therapy in minimizing user conflicts. These factors are considered under the "social" dimension of tranquility above.

Data Sources Used
Field assessment, site-specific information.

Representative Quotes from Forest Therapy Guide Interviews
Loops are nice. ...The loop works well.

...circular...Ideally loop trails are best.

A loop or one way out and back both work.

Summary/Highlights from Research, Planning, and Design Literature
Loop trails permit users to return to the starting point without retracing steps; they present the user with a unique and varied sequence of features and experiences. Linear routes are appropriate for destination trails, long distance trails, and for small or narrow sites where loop trails are not possible [126,135,136].

7.3. Views

Definition

Types and observer position of views experienced along the trail, including the location of key observation points (KOPs).

Evaluation Procedures

Through field assessment, describe view types (panorama/vista/distant, feature/focal, enclosed/interior/canopied, detail/close-up) and observer position (level/normal, above/superior, below/inferior) of typical and important views. Topographic maps, aerial imagery, and/or the placemark feature on a smartphone mapping application can be useful to note the location of KOPs. Trails with a variety of views and view types are preferred.

Additional Explanatory Notes

In this assessment, there were substantial seasonal differences (leaf-on and leaf off) in focal and distant views, particularly for the Northwoods study area which had higher topographic variation.

Data Sources Used

Field assessment, topographic maps and aerial imagery, smartphone trail mapping app with placemark feature.

Representative Quotes from Forest Therapy Guide Interviews

Vistas are nice…Access to open spaces is nice to notice different directions, feeling the sun from different directions… A place overlooking water is nice.

Extent also plays a role in how restorative we perceive an environment is; how far we can see, can give us a sense of safety that there isn't anything hiding. Based on our evolution we are alert for predators … Those kinds of elements give a sense of safety.

…vistas into a variety of landscapes are nice… I love the big, magnificent trees, but it is often the small things that people are drawn into.

Summary/Highlights from Research, Planning, and Design Literature

For most people, visual perception is the dominant sensory facility and the visual landscape the dominant form of nature appreciation [59, 138]. The character of views is central to scenic quality assessments and landscape architects have developed viewscape taxonomies to describe the various types of views that elicit aesthetic

responses [29]. Likewise, environmental psychologists and others have studied and theorized how view qualities such as expanse, extent, and prospect-refuge relate to people's preferences, perceptions, and behavior to affect human health and wellbeing [71,139,140].

7.4. Spaces

Definition

Openings, clearings, key locations, or other spaces that occur naturally or are created through vegetation management along the trail that provide a pleasant, suitable stationary setting for private reflection ("sitspots") or group forest therapy activities ("invitations").

Evaluation Procedures

Through field assessment, identify openings, clearings, key locations, or other spaces that occur naturally or are created through vegetation management along the trail that provide a pleasant, suitable stationary setting for private reflection ("sitspots") or group forest therapy activities ("invitations"). Topographic maps, aerial imagery, and/or the placemark feature on a smartphone mapping application can be useful to note the location of these spaces. Trails should have spaces every so often for individuals to stop and rest and reflect, and/or for groups to assemble for guided activities.

Additional Explanatory Notes

Private spaces tend to be small in scale and enclosed by landform or vegetation ("nooks"), located off-trail or along trail spurs to provide a sense of privacy (refuge), and make use of built/borrowed features and views to afford opportunities for reflection (prospect). Group spaces should ideally also be off main trails if the trail is a busy one but can be larger and more open to accommodate desired group size. As group activities are often driven by invitations provided by a group guide, views from group spaces may be less important than having a level, open space with low grass or durable groundcover for standing or sitting.

Data Sources Used

Field assessment, topographic maps and aerial imagery, smartphone trail mapping app with placemark feature.

Representative Quotes from Forest Therapy Guide Interviews

Then, also having places for refuge where you can be on your own in the midst of a vast environment. These elements allow people to have their own experiences.

Next would be nooks along the trail where someone could step aside for some reflective time.

A (narrow) linear trail can be limiting, you need some openings that are not so closed in… places to be able to sit in a circle.

Places to sit and gather are important…Open areas to have circle. Need a place for 10 people to gather…natural gathering places.

Summary/Highlights from Research, Planning, and Design Literature
Trails should have spaces every so often for individuals to stop and rest and reflect, and/or for groups to assemble and participate in guide-led invitations [12,71]. Trails should provide a diversity of spatial types for different preferences, group sizes, and purposes [141–143]. For individuals or small groups, small spaces can be defined by vegetation to provide a sense of privacy and focus attention on landscape details, while larger group spaces should ideally be shaped so that individuals can come together in a circle, or at least be wide enough so that other trail users can pass [15]. Accessibility standards call for trails to have resting intervals every 200 ft (60 m) for grades < 8 1/3%, 30 ft (9 m) for grades 8–10%, and 10 ft (3 m) for grades 10–12%, with resting spots 5 ft (1.5 m) long and as wide as the trail, and passing spaces every 1000 ft (300 m) if the trail tread width is < 5 ft (1.5 m) wide [120].

7.5. Changes

Definition
The number or variety of transitions in elevation, vegetation types, spatial patterns, or other features along the length of a trail that create different "rooms" or "reaches" and provide noticeably different perceptual and sensory experiences.

Evaluation Procedures
Using field assessment with the aid of aerial imagery or other site specific information sources, describe the number or variety of transitions in elevation (e.g., flat to hilly), vegetation types (e.g., forest to wetland, young dense forest to mature forest with open understory), spatial patterns (e.g., closed forest to open clearing), or other features (e.g., rocky area, water body, buildings or developed area) along the length of a trail that create different "rooms" or "reaches" and provide noticeably different perceptual and sensory experiences. Preferred trails have a variety of changes along their length.

Additional Explanatory Notes
A room or reach is an identifiable area or segment along a trail of variable size or length that has its own homogeneous character with respect to stream gradient, vegetation cover, design qualities, or other attributes that differ in a noticeably significant way spatially, physically, or biologically from other rooms or reaches.

Data Sources Used
Field assessment.

Representative Quotes from Forest Therapy Guide Interviews
Next would be habitat transitions, which are very helpful as well. This can be any type of habitat transition. It can be going from woods to meadow to wetland and also differences in elevation work, too. As many of those as possible without being redundant. You can overdo it very easily. On a half mile trail three or four habitat transitions are enough.

...an overstory and changes in types of plants is nice; variety of plants and colors, natural transitions, grassy to woodland creek etc., natural changes every 15-20 minutes.

I think a variety of experiences, a stream with a platform or a rock you can sit on in the middle of the stream, that takes you into a different landscape or archetypal features, big rocks, open spaces, noticing the different features in the land.

Summary/Highlights from Research, Planning, and Design Literature
Changes in vegetation cover types, elevation and landform, spatial enclosure and view types, and other small- and large-scale natural features increase interest and encourage a sense of exploration in forest therapy engagements [144–148].

Part II B. Key Trailside Features and Opportunities

8. Natural Features

Definition
Objects and elements of nature occurring along the trail that enhance forest therapy experiences including dominant vegetation cover and distinctive trees, water bodies, wildlife, and other elements along with any notable related multisensory characteristics.

Evaluation Procedures
Using the information below, make an evaluative rating that expresses the amount or prominence of natural features visible and accessible along the trail. While each trail will have a unique combination of natural features, trails with big and/or distinctive trees, water that is prominent and physically accessible, observable wildlife, and/or other distinctive natural vegetation, landform, or rock features and related multisensory effects will enhance forest therapy experiences.

Evaluation Rating
low–moderate–high

8.1. Vegetation Cover

Definition
The dominant vegetation community types visible along the trail corridor.

Evaluation Procedures
Through field assessment along with the aid of aerial imagery and available site-specific information, list and briefly describe the dominant natural or cultural vegetation community types (e.g., forest, savanna, wetland, prairie, barrens, beach, old field, garden) visible along the trail. For forest communities it may be helpful to include dominant species, density, and age class of overstory tree cover.

Additional Explanatory Notes
While dominant vegetation cover may not be a key natural feature, it helps to characterize the context or matrix in which key natural features occur.

Data Sources Used
Field assessment, aerial imagery, site-specific information.

Representative Quotes from Forest Therapy Guide Interviews
The main trail is under a shady canopy of trees with a medium size meadow or open field.

It has a massive wetland system with a board walk and other things and then it has forest ecosystem and prairie grassland ecosystem… and depending on the length of my walk and what I want to achieve I can utilize all of those. It is great to guide people through the various ecosystems.

The forest at the Nature Center is deciduous, some pines would be nice. It would be nice if the understory was clear…People don't want to go out there because of ticks and other things. If it was more of a beech or maple basswood forest with a clean forest floor that would be ideal.

Summary/Highlights from Research, Planning, and Design Literature

Vegetation cover or land cover is a principal component in landscape evaluations, and describing its characteristics and patterns is essential in determining landscape quality for cultural ecosystem services including scenic beauty, recreation, and human well-being [58,59]. In forested landscapes, the structural components of forest stands are often used in predictive models of landscape quality and user preference [150–153].

8.2. Trees

Definition

Trees with large diameters and/or old growth characteristics, single or multiple in number, including stands of mature and old growth forests, as well as distinctive tree species, shapes, growths, large standing dead and downed trees, and related multisensory characteristics including seasonal effects.

Evaluation Procedures

Through field assessment, identify any trees with noticeably large trunks, estimating their diameter at breast height visually or with the use of a tape measure. If using a phone-based mapping app such as Avenza, it is helpful to locate and label large trees with a placemark. Note if many such trees occur, in groups, dispersed, or whether the trail corridor goes through a mature or old growth forest. In addition to big trees, note any distinctive tree types or species, those with distinctive branching or other growth habits, growths such as burls or trees with distinctive moss, lichen, algae, or fungus growing on them, distinctive standing dead or downed trees, and notable multisensory characteristics such as bark texture, scented blossoms and leaves, rustling and creaking sounds.

Additional Explanatory Notes

Whether a tree is considered big is highly area dependent and thus it can be important to identify such trees in the context of dominant vegetation cover (above). For this assessment, the diameter of big trees varied by forest community type: For the Northwoods study, guidelines specify hardwoods and conifers with minimum diameter at breast height (dbh) of 16 in (40 cm) and for the Chicago study area northern mesic oaks of 20 in (50 cm) dbh or larger. Northwoods forests were considered mature and old growth if

10 trees/ac (25 trees/ha) were at least 12–16 in (30–40 cm) dbh (avg 141 yrs old), and for Chicago mesic forests 5 trees/ac (12 trees/ha) at least 20 in (50 cm) dbh (avg 161 yrs).

Data Sources Used
Field assessment, smartphone trail mapping app with placemark feature.

Representative Quotes from Forest Therapy Guide Interviews
I love the big, magnificent trees.

...Trees are so important, reciprocity or communication with a tree is dynamic and powerful. An experience with a tree can be really powerful. Tree interaction is really important. I love it when people can lean against a tree or sit on a tree. There are so many fabulous ways to interact with a tree.

(The guide) led me up to this enormous redwood tree and he got me about an inch away from it and he said, "OK open your eyes." It was kind of an awakening moment for me because I remember seeing the bark of the tree in such intense and complex detail and it was kind of a call back to ...really seeing the world.

Summary/Highlights from Research, Planning, and Design Literature
Big trees, alone and as part of mature or old growth forests [154], provide a host of ecological and cultural ecosystem benefits, and for the latter they are revered not only for their aesthetic quality but also have important spiritual values for many religious and cultural groups around the world [155–157]. Related big tree and old growth properties, from attractive bark texture to rare and common plant and animal species that live in symbiotic relationships with such trees and forests, broaden the range of benefits of big trees and old growth forests [158–160].

8.3. Water

Definition
Type (e.g. lake, river, wetland), visual prominence (size, distance zones, and duration of view), access (visual, physical), and usability (safety, restrictions on, and desirability for bodily contact from touching to full immersion) of natural or human modified/created water bodies along the trail, along with key water-related sensory effects.

Evaluation Procedures
Using field assessments along with other available site information, list water bodies visible along the trail in terms of water body type, including natural water bodies from springs, small creeks, wetlands, rivers, and lakes to large lake- and sea-shore areas, as well as any human created water features such as fountains or waterfalls. Describe their visual prominence in terms of their size, how close they are to the trail (Immediate Foreground IFG 0–100 ft (0–30 m), Foreground or FG 100 ft – 1/4 mi (30 m – 0.4 km), Middleground or MG 1/4 – 1 mi (0.4–1.6 km), Background or BG > 1 mi (> 1.6 km)), whether they can be accessed visually or physically, and give some indication of the duration of time or percentage of trail length that they are visible (e.g., single point, multiple points, length of trail segment(s)). For water bodies that are physically accessible, give some indication of their usability for human contact, taking into account physical safety and water quality, use restrictions, and desirability for contact from touching to partial to full immersion. Finally, mention whether the water bodies provide key multisensory effects such as ripples and wave motion, sounds, microclimate changes in temperature and humidity, smells.

Additional Explanatory Notes
For paddle trails, visual prominence, duration of view, and physical access are givens, but usability is especially important. In some urban areas, paddle trails can provide satisfying experiences even though the water may not be desirable for direct human contact due to pollution or eutrophic conditions.

Data Sources Used
Field assessment, topographic maps and aerial imagery, other available site information.

Representative Quotes from Forest Therapy Guide Interviews
Water features are important, including creeks, rivers, waterfalls, and lakes for a variety of invitations. ...Sounds of water are good for people, especially people with autism and other challenges. Many conditions benefit from sounds of water.

I would love to have access to water. While there is water in the wetlands at the Nature Center you can't get to it and put your hands in the water. To get to the water's edge is difficult.

Water features are important. ... if they can get close to water that is a huge thing. The tactile sense brings people in very quickly...

Summary/Highlights from Research, Planning, and Design Literature

The presence of water in a landscape has high aesthetic appeal and the ability to view and physically access water can have important restorative health impacts for those who come into contact with water [161–163]. These relationships hold for many types of water bodies large and small, natural and artificial, though the quality of water resources can affect perceptions and determine the suitability of use for people [164–168].

8.4. Wildlife

Definition

Commonly viewed and/or seasonally important mammals, birds, insects, etc., along with prominent habitat, nesting, and observation opportunities, and key wildlife-related sensory effects and seasonal opportunities.

Evaluation Procedures

Using field assessments along with other available site information, list mammals, birds, insects, etc., along with prominent habitat (e.g., beaver dams and ponds, productive wetland areas), nesting (eagle, egret nests), and observation opportunities (e.g., protected breeding areas, wildlife blinds, seasonal migrations), and key wildlife-related sensory effects (e.g., birdsong, migrating flocks moving across the sky, smells, etc.).

Additional Explanatory Notes

Many wildlife encounters and opportunities are highly ephemeral in time and space and thus it is not easy to count on experiencing them on a given trail trip. The key here is to describe the more stable types of habitats and conditions where different types of wildlife are more likely to occur, and trails that have such conditions can enrich forest therapy experiences.

Data Sources Used

Field assessment, available site-specific information.

Representative Quotes from Forest Therapy Guide Interviews

…it works better if you have an open space where there are birds and insects, bees and butterflies. Birds are important. …birds and insects in meadow for the "what's in motion" invitation; birds are important; sounds of birds, crickets, etc. are the most important thing to me.

A variety of wildlife species would be good, but no snakes or scary animals (or alert people of possible dangers).

Or if they see a feather, as long as it is not an endangered species or protected species feather, we encourage them to pick it up. What is this feather telling you? They may want to put it in their lapel or their hair, anything to help make the connection.

Summary/Highlights from Research, Planning, and Design Literature
Wildlife big and small ("from bears to bugs") is an important source of fascination to people in outdoor environments. Wildlife not only helps bring the landscape to life with motion and sound but can make humans more aware of the broader non-human world and their place in it [144,169–171]. Landscapes that are high in environmental quality provide greater opportunities to observe wildlife and appreciate the habitats that support it [172,173]. People's comfort with wildlife depends on their familiarity, experience, and knowledge, and programs and information can help wary individuals to better appreciate and understand wildlife in all forms [103–105].

8.5. Other Natural Features

Definition
Other distinctive vegetation, landform, rock features and related sensory effects.

Evaluation Procedures
Using field assessments along with other available site information, list or briefly describe other distinctive vegetation (e.g., ferns, moss, fungi), landform (e.g., canyons, ridges, hilltops, flats or plains), and rock (e.g., large rocks, outcrops, rocky shorelines and lake or stream bottoms) features and related sensory effects (e.g., tactile, smells, sounds).

Additional Explanatory Notes
While many natural features provide important tactile or haptic experiences, moss and large rocks or rock outcrops seem to especially invite bodily contact for sitting or lying, walking, and other forms of sensuous exploration.

Data Sources Used
Field assessment, available site-specific information.

Representative Quotes from Forest Therapy Guide Interviews
…archetypal features, big rocks, open spaces, noticing the different features in the land.

...Moss is magical...There is another section of that trail that is really covered with moss because they don't get a lot of people there. Every day I would go and just lie in the moss. It was just amazing. ...

I love doing barefoot walks when possible. People really love that. That is a huge sensory experience.

Summary/Highlights from Research, Planning, and Design Literature
Natural areas are often host to a variety of other natural features that can be focal points of attention in the course of forest therapy walks [64,148]. Many of these are small in scale and noticed at the ground level such as flowers, rocks, moss, and fungi, and are distinguished from the matrix of green vegetation by vibrant colors, unique textures, smells, shapes, and other multisensory characteristics [174–176]. The properties of these features often make them popular for inclusion in garden designs that can heighten people's awareness and appreciation of the beauty in nature [177,178].

9. Built and Borrowed Features

Definition
Human-built, naturally occurring, or human-adapted natural features that serve utilitarian, aesthetic, or symbolic functions along the trail for seating, gateways, shelter, and other purposes.

Evaluation Procedures
Using the information below, make a summary evaluative rating that expresses the amount or prominence of human-built, naturally occurring, or human-adapted objects or spaces present along the trail that serve utilitarian, aesthetic, or symbolic functions for seating, gateways, shelter, and other purposes. Built and borrowed features that work in harmony with the trail's setting and natural features can help enhance forest therapy experiences.

Evaluation Rating
low–moderate–high.

9.1. Seating

Definition
Human-built, naturally occurring, or human-adapted natural objects or spaces that afford places to sit along the trail.

Evaluation Procedures
In a field assessment, identify and note the location of seating and types of seating from informal natural materials and features such as fallen logs and stones to borrowed or modified natural materials such as cut tree stumps and cut stone slabs, to manufactured seating such as benches, chairs, and picnic tables.

Additional Explanatory Notes
It is helpful to note whether seating is designed for solitary or small group use as well as locations along the trail where seating can accommodate larger groups, such as campfire and council ring areas.

Data Sources Used
Field assessment, smartphone trail mapping app with placemark feature.

Representative Quotes from Forest Therapy Guide Interviews
It doesn't need to be park benches. It can easily be a log or a large rock with some flatness to it. Find things that work for sitspots.

How beneficial it would be to have natural looking seating. Manmade structures that look safe would help some people who might hesitate to sit on a log for example. Small benches or sit-upons would be nice. It's almost like there is an inviting quality.

Thinking about comfort in seating for different user groups is important. Sometimes it is hard to get up and down from the ground, and you can only lean against a tree for so long and you need to sit. That would be an important feature to consider. It would be important to have 2–3 places along the trail, not all together, where people could sit. These could be part of sitspots. Those would be important features… And have places to sit so people don't have to sit on the ground.

Summary/Highlights from Research, Planning, and Design Literature
Natural objects and natural materials adapted for use in seating may have high visual appeal but must also afford comfort and function [71,179–181]. Principles of biophilic

design and contextual compatibility can help identify a good balance between aesthetics and utility to ensure that seating and other built features are optimally designed [182–186].

9.2. Gateways

Definition
Human-built, naturally occurring, or human-adapted natural objects and spaces that provide a physical or symbolic entry or exit point to a trail.

Evaluation Procedures
In field assessment, note any signs, markers, archways, gates, rocks, trees, modifications in vegetation, ground textures and materials, or other human-built (e.g., metal gates, bridges, trailhead information kiosks), naturally occurring (e.g., big standing or fallen trees, boulder), or human-adapted natural objects and spaces (e.g., trail marker trees, rocks arranged in place), singly or in combination, that provide a physical or symbolic entry or exit point to a trail.

Additional Explanatory Notes
Even modest gateways can be symbolically useful in providing a perceptual threshold marking the beginning and end of a trail experience.

Data Sources Used
Field assessment.

Representative Quotes from Forest Therapy Guide Interviews
Having a physical threshold is nice, an entry from tame to wild. Then coming back to the same spot for completions and transitioning back. One thing that works very well is a bridge. I have one spot I go to, and the threshold is a bridge. We enter over the bridge, and we leave over the bridge. Rock outcroppings work, too. Something that marks "we are here."

…an (entry) threshold of some sort that would be symbolic of letting go of the past, at least temporarily, and immersing yourself in the bosom of the mother. The threshold could be a bridge, it could be walking into another habitat, into the woods from a meadow, something that could be a symbolic transition. Some kind of line, something that feels like a threshold.

Summary/Highlights from Research, Planning, and Design Literature
Good gateways create a sense of passage, they may constrain the view to focus or lead the eye. Entry gateways invite the user to explore while exit gateways can provide a sense of closure; each serves to distinguish spaces between inside and outside of the trail experience. Gateways can also be employed at transition points to mark changes such as between two spaces along a trail [71,122,176].

9.3. Shelter

Definition
Human-built, naturally occurring, or human-adapted natural objects and arrangements that provide a partial buffer or more complete protection from weather elements including sun, wind, temperature, or precipitation.

Evaluation Procedures
Using a field assessment and any available site-specific information, identify and locate any human-built (e.g., gazebos, picnic shelters), naturally occurring (e.g., big canopy trees), or human-adapted natural objects and arrangements (e.g., lean-tos made of branches) that provide a partial buffer or more complete protection from weather elements including sun, wind, and temperature, or precipitation.

Additional Explanatory Notes
Most formal, built shelters will be at or near the trailhead, though some trails will have shelters at locations along the trail, especially if the trails are part of a longer distance hiking or ski trail.

Data Sources Used
Field assessment, available site-specific information.

Representative Quotes from Forest Therapy Guide Interviews
...should have shelter of some sort, in case of thunderstorms or rain. A gazebo is wonderful.

Also, some kind of shelter from the sun, throughout. A simple awning or something that could provide shade, especially in an area that is really hot and where shade is illusive... Maybe there could be a tarp or a special half dome tarp so you could have a fire, and people could still get out of the rain and still be open with their senses. You just have to work with whatever is there but look for options for colder, wetter weather.

Like a grove of trees, to have a space like a sense (that there's a) room, where you feel comfortable and cared for.

Summary/Highlights from Research, Planning, and Design Literature

As with seating, principles of biophilic design and contextual compatibility are important in designing shelters that fit within the context of the trail setting. Use of natural materials, scale, and location can ensure human-environment and enhance psychological restoration [71, 179–186].

9.4. Other Built and Borrowed Features

Definition

Additional built and borrowed features to facilitate use and protect people and the environment, special features to enhance the nature experience, and other features or evidence of past features and activities that reflect and maintain the cultural-historical landscape.

Evaluation Procedures

Using a field assessment and any available site-specific information, list any additional human-built, naturally occurring, or human-adapted natural features including boardwalks (mention material), bridges (from logs placed across a stream to major bridge constructions), fencing (e.g., split rail, metal, etc.) or other features to facilitate use and protect people and the environment; special features to enhance the nature experience (e.g., firepits, wildlife blinds, observation platforms, sculptures); and other features or evidence of past features and activities that reflect and maintain the cultural-historical landscape (e.g., buildings, stone walls, building foundations).

Additional Explanatory Notes

The focus here is on other built and borrowed elements that facilitate trail use and enhance the nature experience; other built elements that may be discordant to this purpose are inventoried under tranquility (visual disruptions).

Data Sources Used

Field assessment.

Representative Quotes from Forest Therapy Guide Interviews

Then, at the end of the trail, a sit-down area, a place to reflect, sometimes a fire pit in the center is nice. …

And then an area for tea ceremony that might have a nice view or underneath a large tree, those special features.

We always look for dynamic places. I often go to a site 5–6 times before I take a group there. …I usually find a very dramatic tree. A place overlooking water is nice.

Summary/Highlights from Research, Planning, and Design Literature
Again, principles of biophilic design and contextual compatibility are important in designing other features that fit within the context of the trail setting. Use of natural materials, appropriate scale, and location can ensure human-environment fit and enhance psychological restoration [71, 179–186].

10. Explorable Nature

Definition
Policies and design features that provide or restrict types of exploratory on and off-trail activities that enhance forest therapy experiences.

Evaluation Procedures
Using the information below, make a summary evaluative rating that expresses the degree of allowable uses or restrictions, museumification, and engagement on and off the trail. Generally, policies and design features that permit responsible interactions with natural features within and outside the immediate trail corridor are desirable.

Evaluation Rating
Low–moderate–high.

10.1. Uses and Restrictions

Definition
Written or otherwise expressed policies that allow, provide for, or restrict off-trail exploration, play, sampling, foraging, collecting, and related activities as part of a forest therapy experience.

Evaluation Procedures
Using available information at trailhead kiosks or online for the site or agency, list and describe allowable activities or activity restrictions as they pertain to going off-trail,

sampling, foraging, collecting (take home or onsite use), and related activities, including provisions for activities such as a firepit for making a fire for use in a forest therapy tea ceremony.

Additional Explanatory Notes

In this assessment, most of the Chicago study area trails had policies restricting off-trail use and foraging/collecting, while most of the Northwoods trails had few restrictions beyond statewide regulations for hunting and fishing, collecting endangered plants, and digging up live plants, and even most State Natural Areas allowed for some types of foraging for nuts, berries, and mushrooms.

Data Sources Used

Information at trailhead kiosks or online for the site or agency.

Representative Quotes from Forest Therapy Guide Interviews

Places where people can wander in the woods are also nice. ...And having places where people can spread out is optimal and where people can go off trail. ...allowing guides to harvest plants. Hopefully, in the future there will be forest therapy parks where guides can harvest natural herbs.

We have been so indoctrinated with Leave No Trace some people are very uncomfortable about potential impact... (For this reason) the concepts of (nature being) welcoming and safety and not being a stranger are so important to convey along the trail...

Summary/Highlights from Research, Planning, and Design Literature

While the protection and restoration of quality environments from trampling or removal of sensitive plants and objects is an important goal of natural and cultural areas management, being able to physically interact with plants and other elements of nature is a key dimension of forest therapy experiences [187–189]. Programs that teach trail use ethics such as Leave No Trace help can help ensure resource protection through responsible use [190–192].

10.2. Museumification

Definition

Signage, physical or symbolic barriers, or visual cues installed along the trail that have the effect of limiting forest therapy interactions to mostly visual on-trail observation, as if visitors were in a museum.

Evaluation Procedures
Field observation-- list and describe signs, fencing, rope barriers, or other physical or symbolic barriers or visual cues installed continuously or at multiple points along the trail that remind visitors to "look but don't touch" that have the effect of limiting user experience and multisensory engagement to mainly visual observation.

Additional Explanatory Notes
Some level of museumification may be necessary at sites such as botanic gardens and sensitive natural areas, especially along busy urban trails.

Data Sources Used
Field assessment.

Representative Quotes from Forest Therapy Guide Interviews
There is this thing about separation that we internalize. Many trails have logs as barriers along the edges to separate people and it is nice when there are only logs on one side of the trail to kind of direct people to where they are going, and the other side is open ...where you invite people to step off the trail and interact with the environment where it is appropriate.

Summary/Highlights from Research, Planning, and Design Literature
As the qualities and features of natural and cultural sites also make them attractive candidates for forest therapy trails and related forms of nature-based recreation, care must be taken to balance goals for ecological and cultural authenticity with providing for the authenticity of nature experiences [193,194].

10.3 On-Trail Engagement

Definition
Design and management characteristics of the trail and its right of way that facilitate or hinder engagement and interaction with plants and other natural features of the immediate trail setting.

Evaluation Procedures
Briefly describe whether and how trail design (e.g., width, alignment) and right of way (ROW) management (e.g., mowing and trimming of vegetation) facilitate or hinder engagement and interaction with plants and other natural features within the immediate trail setting.

Additional Explanatory Notes
While trail width and alignment factor into the design and construction aspects for evaluating a trail's layout attractiveness and ease of travel, they also play mediating roles in making nature usable and explorable within the trail ROW. This is especially important for urban trails and trails in sensitive ecological areas where use restrictions such as foraging and off-trail travel and/or museumification elements are present. In these cases, note whether trail widths and trail right of way vegetation management still allow visitors opportunities to touch, brush against, smell, or otherwise come into close contact with natural elements to obtain a more engaging, multisensory experience beyond visual observation.

Data Sources Used
Field assessment

Representative Quotes from Forest Therapy Guide Interviews
In places with manicured gardens where you can't leave the trail it is important to have places where… people get the feeling that they are stepping off the trail even though it might be part of the trail.

…having trails where people who are able to can get close and be in direct contact with the plants along the sides of the trail.

Summary/Highlights from Research, Planning, and Design Literature
Plants and objects that are within arm's reach maximize opportunities to strengthen people's psychological connection with nature. A trail with a managed right of way that allows for voluntary instead of forced contact invites exploration while minimizing any psychological discomfort for people unfamiliar with or wary of potentially harmful plants and insects [71,193].

11. Interpretation and Stewardship

Definition
Onsite signage and on- and offsite programs and other opportunities that help enhance nature experiences, knowledge, appreciation, and stewardship behavior.

Evaluation Procedures
Using the information below, make a summary evaluative rating of the level of interpretation and learning/stewardship opportunities available on or in association with the trail. Attractive, contextually compatible interpretive signage and auxiliary information, programs, and activities that facilitate environmental learning and involvement can enhance nature experiences over the short and long term.

Evaluation Rating
Low–moderate–high.

11.1 Signage

Definition
Signs or markers along the trail that help to interpret or enhance nature experiences and appreciation.

Evaluation Procedures
In field assessment, identify and describe any informational or interpretive signage or markers along the trail.

Additional Explanatory Notes
Beyond their immediate trailheads, most trails in this assessment had few or no interpretive signs along the route beyond directional or wayfinding markers. Exceptions to this were interpretive nature trails on the grounds of nature centers or botanic gardens.

Data Sources Used
Field assessment.

Representative Quotes from Forest Therapy Guide Interviews
...you don't want a bazillion signs that overwhelm people, but good signage with simple invitations in the right spots. Signs that invite people to sit and pay attention. ...really work with the natural features that are there... I really would encourage trail builders to work with what is the natural invitation in that spot naturally.

Reading is such a cognitive process. It brings us away from the awareness of our embodiment when we read something. So, that poses an interesting challenge for use when we are thinking about how to create signage to help people get into a state of embodiment.

Many people don't want to hold a brochure, or kill a tree, so in time invitations may be digital… I think there are ways with OnCell (https://www.oncell.com/), where you can download a self-guided trail app and go to that trail and walk to spot one and it would pop up and guide with an invitation. It could even be voice activated.

Summary/Highlights from Research, Planning, and Design Literature
While the design and implementation of interpretive communication through onsite signage and offsite and online delivery systems is not yet well developed for use in forest therapy, the basic formats used in nature and museum visitor interpretation have potential for adaptation, particularly for self-guided experiences [195–197]. Attractiveness and simplicity are two principles that can increase the probability that signs will be used [122,136].

11.2. Environmental Learning and Stewardship Opportunities

Definition
Programs, volunteer workdays, on and offsite activities, and other opportunities aimed at educating visitors about the site and trail and/or involving them in efforts to protect, maintain or restore the site or trail's natural or cultural qualities.

Evaluation Procedures
Mostly provided through off-site information on agency or organization websites, identify any programs or events (e.g., guided nature walks), volunteer workdays (e.g., litter pickups, trail maintenance, ecological restoration workdays), or other on and offsite activities (e.g., nature center exhibits) aimed at educating visitors about the site or trail and/or involving them in efforts to protect, maintain, or restore the site or trail's natural or cultural qualities.

Additional Explanatory Notes
In this assessment, while many of the urban natural area sites had active volunteer organizations that supported agencies in ecological restoration efforts, most did not link these activities with experiential programs such as forest bathing or nature walks, missing an important opportunity to connect appreciation and action.

Data Sources Used
Field assessment, available site-specific information.

Representative Quotes from Forest Therapy Guide Interviews

I believe one of the benefits of forest therapy work includes higher levels of environmental stewardship after a walk. Empathy for nature can increase.

Avoid engaging conversation on "what kind of tree is that?" Where that does happen is in tea ceremony, concluding the sensual experience and inviting other inquiry. That might be the time to entertain these other ideas and questions… As the guide I would share my knowledge and suggest other sources and other places they might go.

The whole point of having a (forest therapy walk) near a visitor center or arboretum… is to engage people and raise awareness and have people explore and learn more about the area. Definitely a perk. People may come back. They may even volunteer or help out.

Summary/Highlights from Research, Planning, and Design Literature

Connecting aesthetic experience to environmental stewardship takes a delicate hand, and making environmental learning available for trail users to seek out on their own time and pace can help raise awareness and concern that in turn may lead to action in terms of engagement in stewardship activities and other forms of support for nature protection and restoration [198–200].
