# Supplementary material for: Forest Therapy Trails: Development and Application of an Assessment Protocol
Source: Int J Environ Res Public Health. 2025 Sep 16;22(9):1440. doi: 10.3390/ijerph22091440 (PMC12470198; doi:10.3390/ijerph22091440)
Supplement: Supplementary file 1 [file ijerph-22-01440-s001.zip › Supp Doc S2 Protocol Template.pdf]

Site and Trail Evaluation Worksheets

Part I. Site Evaluation Worksheet (See full protocol for details)

**Site Name:**						
**Location:**						
**Site Type:**						
**Online Info:**						
**Summary Scores**	**Beauty**	**Integrity**	**Tranquility**	**Accessibility**	**Total**	**Pct**
**Low = 1, Moderate = 2, High = 3**						**n/12**

1. **Landscape Character and History-** List the key characteristics of the site's geographical and social setting, jurisdiction, land use type, land cover or ecological landscape type, geographic or cultural region, and special designations. Summarize the site's landscape history that has influenced current patterns and features. (Not rated)

2. **Beauty-** Summarize and rate the physical, biological, and/or cultural patterns and features in terms of their variety-diversity and vividness-prominence on the site, highlighting any unique or special features.

3. **Integrity-** Summarize and rate the ecological, recreational, and/or cultural-historic patterns and features in terms of their condition or intactness.

4. **Tranquility-** Summarize and rate the setting, sensory, social, and environmental conditions present that intrude upon the site's tranquility.

**Setting**	State the size of the site and describe the character and compatibility of adjacent land uses.
**Visual**	State the type, distance (distance zones), magnitude/scale, and compatibility of intrusive visible development, noting key points or stretches.
**Sound**	Describe the types and magnitude of sound disturbances including roads, other transportation, and adjacent land uses and activities (on- or off-site).
**Other**	Describe the type and magnitude of any other sensory intrusions (smells, smoke/dust, light).
**Social**	Estimate the average range in trail use levels and note user type and compatibility on trails and in adjacent use areas.
**Environmental**	Record everyday or seasonal environmental conditions that could challenge or induce physical or emotional stress to some users.

5. **Accessibility-** Summarize and rate the proximity, available facilities, user fees, and trail options that facilitate or impede access and use of the site.

**Proximity**	Estimate the distance and/or time to the main trailhead of the site from a chosen point of origin.
**Facilities**	List and briefly describe parking, toilet, drinking water, and other support facilities available at or within close access to the trailhead.
**Fees**	Describe any fees needed to access the trailhead or individual trails on the site, in addition to guide or tour fees if applicable.
**Options**	Summarize the number, type, and range of difficulty of trails available within the site.

Part II. Trail Level Criteria

<table>
<tr><td colspan="5">Trail: Trail name</td><td colspan="5">Type: e.g., Foot, Bike, Paddle</td></tr>
<tr><td colspan="10">Trail info: List any trail-specific URLs</td></tr>
<tr><td colspan="5">N visits:</td><td>Seasons:</td><td>W</td><td>Sp</td><td>Su</td><td>F</td></tr>
<tr><td>Score</td><td>Ease of Travel</td><td>Attractive Layout</td><td>Natural Features</td><td>Built Features</td><td>Explorable Nature</td><td colspan="2">Interp.</td><td>Total</td><td>Pct</td></tr>
<tr><td>L/M/H
1/2/3</td><td></td><td></td><td></td><td></td><td></td><td colspan="2"></td><td></td><td>n/18</td></tr>
</table>

Part II A. Design and Construction

6. **Ease of Travel-** Synthesizing the information below, make an evaluative rating incorporating trailhead distance, length, surface, width, slope, and accessibility barriers to assess the ease of travel on the trail.

**Trailhead Distance**	Measure distance between the starting point (e.g., parking lot) to where the actual forest therapy trail experience begins.
**Length**	Record the distance along the trail or trail segment from the trailhead and back.
**Surface**	List different surface types and dominant surface(s).
**Width**	Record minimum, maximum, and modal trail width.
**Slope**	Record maximum and average slope.
**Accessibility Barriers**	List type and frequency of barriers or obstacles.

7. **Attractiveness of Layout-** Synthesizing the information below, make an evaluative rating incorporating alignment, route type and directionality, key views, spaces, and related spatial-temporal changes encountered along the trail that enhance forest therapy experiences.

**Alignment**	Record the horizontal and vertical layout or routing of a trail corridor through a site.
**Route Type**	Describe route type and directionality: loop/linear/other, 1-way/2-way.
**Views**	Describe view types and observer position of typical and important views, noting the location of key observation points (KOPs).
**Spaces**	Identify openings, clearings, or other spaces that occur naturally or are created through vegetation management that provide settings for private reflection ("sitspots") or group activities ("invitations").
**Changes**	Describe significant transitions in elevation, vegetation types, spatial patterns, or other features along the trail that create different "rooms" or "reaches" and provide different perceptual experiences.

Part II B. Key Trailside Features and Opportunities

8. **Natural Features-** Using the information below, make an evaluative rating that expresses the amount or prominence of natural features along the trail, including trees, water, wildlife, and other significant or distinctive natural features.

**Vegetation Cover**	Describe the dominant natural or cultural vegetation community types to help characterize the context in which key natural features occur.
**Trees**	Identify noticeably large trees along with other distinctive tree types or species, growth habits, growths, distinctive standing dead or downed trees, and notable multisensory characteristics.
**Water**	List water bodies in terms of types; their visual prominence in terms of size, distance from trail, and duration of view; whether visually or physically accessible and if the latter, their usability for human contact; and notable multisensory characteristics.
**Wildlife**	List mammals, birds, insects, etc., along with prominent habitat, nesting, observation opportunities, and key wildlife-related sensory effects.
**Other**	List other distinctive vegetation, landform, and rock features and related sensory effects.

9. **Built and Borrowed Features-** Using the information below, make a summary evaluative rating that expresses the amount or prominence of built, natural, or human-adapted natural features present along the trail that support visitor use and experience.

**Seating**	Identify seating and types of seating, if any.
**Gateways**	Note any features that provide a physical or symbolic entry or exit point to a trail.
**Shelter**	Identify any features that provide a partial or more complete protection from weather elements.
**Other Features**	List other built features that facilitate use and protect people and the environment, enhance the nature experience, or reflect and maintain the cultural-historical landscape.

10. **Explorable Nature-** Using the information below, make a summary evaluative rating that expresses the degree of allowable uses or restrictions, museumification, and engagement on and off the trail.

**Uses and Restrictions**	Describe allowable activities and accommodations for, or restrictions on, going off-trail, sampling, foraging, collecting, and other activities desirable as part of forest therapy experiences.
**Museumification**	List signs, fencing, or other physical or symbolic barriers that have the effect of limiting user experience and multisensory engagement to visual on-trail observation.
**On-Trail Engagement**	Describe how trail design and ROW management facilitate or hinder nature engagement within the immediate trail setting.

11. **Interpretation and Stewardship-** Using the information below, make a summary evaluative rating of the level of interpretation and learning/stewardship opportunities available on or in association with the trail.

**Signage**	Identify any informational or interpretive signage or markers along the trail.
**Learning and Stewardship Opportunities**	Identify any programs, volunteer workdays, or other on and offsite that educate visitors or involve them in site or trail protection, management, or restoration efforts.
