# Supplementary material for: Forest Therapy Trails: Development and Application of an Assessment Protocol
Source: Int J Environ Res Public Health. 2025 Sep 16;22(9):1440. doi: 10.3390/ijerph22091440 (PMC12470198; doi:10.3390/ijerph22091440)
Supplement: Supplementary file 1 [file ijerph-22-01440-s001.zip › Supp Doc S3 Screening Worksheet.pdf]

Site and Trail Rapid Assessment Screening Worksheet

Site Name:						
Location:						
Date of Evaluation:						
Summary Scores	Beauty	Integrity	Tranquility	Accessibility	Total	Pct
Low = 1, Moderate = 2, High = 3						

1. Beauty	Notes:
a. Physical	
b. Biological	
c. Cultural	
2. Integrity	Notes:
a. Ecological	
b. Recreational	
c. Cultural	
3. Tranquility	Notes:
a. Setting	
b. Visual	
c. Sound	
d. Other	
e. Social	
f. Environmental	
4. Accessibility	Notes:
a. Proximity	
b. Facilities	
c. Fees	
d. Trail options	

Part II. Trail Level Criteria

Trail:					Type:			
**Score**	**Ease of Travel**	**Attractive Layout**	**Natural Features**	**Built Features**	**Explorable Nature**	**Interp.**	**Total**	**Pct**
**L/M/H 1/2/3**								**n/18**

**5. Ease of Travel**	**Notes:**
**a. Trailhead Dist.**	
**b. Length**	
**c. Surface**	
**d. Width**	
**e. Slope**	
**6. Layout Attract.**	**Notes:**
**a. Alignment**	
**b. Route Type**	
**c. Views**	
**d. Spaces**	
**e. Changes**	
**7. Nat. features**	**Notes:**
**a. Veg. Cover**	
**b. Trees**	
**c. Water**	
**d. Wildlife**	
**e. Other**	
**8. Built features**	**Notes:**
**a. Seating**	
**b. Gateways**	
**c. Shelter**	
**d. Other**	
**9. Exploration**	**Notes:**
**a. Uses/Restrict.**	
**b. Museumification**	
**c. On-Trail Engage.**	
**10. Interpretation and Stewardship**	**Notes:**
**a. Signage**	
**b. Learn/Steward-ship Opps.**	

Criteria and sub-criteria description (refer to full protocol document for details)

1. **Beauty-** Variety-diversity, vividness-prominence, and/or uniqueness of physical, biological, and/or cultural patterns and features.
   a. Physical- Landform, water, rock/soil patterns and features
   b. Biological- Vegetation, wildlife patterns and features
   c. Cultural- Heritage, land use patterns and features

2. **Integrity-** Condition or intactness of ecological, recreational, and/or cultural-historic patterns and features.
   a. Ecological- Physical, environmental, biological patterns and features
   b. Recreational- Facility and scenic patterns and features
   c. Cultural- Built environment and land use patterns and features

3. **Tranquility-** Setting, sensory, social, and environmental intrusions.
   a. Setting- Size of the site and character and compatibility of adjacent land uses.
   b. Visual- Type, distance (distance zones), magnitude/scale, and compatibility of intrusive visible development.
   c. Sound- Types and magnitude of sound disturbances including roads, other transportation, and adjacent land uses and activities (on- or off-site).
   d. Other- Type and magnitude of any other sensory intrusions (smells, smoke/dust, light).
   e. Social- Trail use levels user type/compatibility on trails and adjacent use areas.
   f. Environmental- Challenging or stressful everyday/seasonal environmental conditions.

4. **Accessibility-** Proximity, available facilities, user fees, and trail options facilitating or impeding site access and use.
   a. Proximity- Distance and/or time to the main trailhead of the site from common point of origin.
   b. Facilities- Parking, toilet, drinking water, and other support facilities available at or within close access to the trailhead.
   c. Fees- Fees needed to access the trailhead or individual trails on the site, in addition to guide or tour fees if applicable.
   d. Options- Number, type, and range of difficulty of trails available within the site.

5. **Ease of Travel-** Trailhead distance, length, surface, width, slope, accessibility barriers.
   a. Trailhead Distance- Distance from parking to trail.
   b. Length- Distance along the trail or trail segment from the trailhead and back.
   c. Surface- Different surface types and dominant surface(s).
   d. Width- Minimum, maximum, and modal trail width.
   e. Slope- Maximum and average slope.
   f. Accessibility Barriers- Type and frequency of barriers or obstacles.

6. **Attractiveness of Layout-** Alignment, route type and directionality, key views, spaces, and related spatial-temporal changes encountered along the trail.
   a. Alignment- Horizontal and vertical layout or routing of a trail corridor through a site.
   b. Route Type- Route type and directionality: loop/linear/other, 1-way/2-way.
   c. Views- View types and observer position of typical and important views.
   d. Spaces- Openings, clearings, or other settings for private or group activities.
   e. Changes- Transitions in elevation, vegetation types, spatial patterns, etc.

7. **Natural Features-** Amount or prominence of trees, water, wildlife, and other significant or distinctive natural features.
   a. Vegetation Cover- Dominant natural or cultural vegetation community types.
   b. Trees- Large or distinctive living or standing dead or downed trees.
   c. Water- Water bod types, prominence, distance from trail, duration of view, visual or physical accessibility, usability for human contact.
   d. Wildlife- Mammals, birds, insects, etc., along with prominent habitat, nesting, observation opportunities.
   e. Other- Distinctive vegetation, landform, and rock features.

8. **Built and Borrowed Features-** Amount or prominence of built features.
   a. Seating- Seating and types of seating, if any.
   b. Gateways- Features that provide a physical or symbolic entry or exit point to a trail.
   c. Shelter- Features that provide a partial or more complete protection from weather.
   d. Other - Other built features that facilitate use, protect people, enhance experience, or reflect the cultural-historical landscape.

9. **Explorable Nature-** Allowable uses or restrictions, museumification, and engagement on and off the trail.
   a. Uses/Restrictions- Allowable activities or restrictions on going off-trail, foraging, etc.
   b. Museumification- Signs, fencing, or other barriers limiting user experience to visual on-trail observation.
   c. On-Trail Engagement- Trail design and ROW management influencing nature engagement.

10. **Interpretation and Stewardship-** Interpretation & learning/stewardship opportunities.
    a. Signage- Informational or interpretive signage or markers along the trail.
    b. Learning and Stewardship Opportunities- Programs, volunteer workdays, or other on and offsite that educate visitors or involve them in site or trail protection, management, or restoration efforts.
