# Supplementary material for: Forest Therapy Trails: Development and Application of an Assessment Protocol
Source: Int J Environ Res Public Health. 2025 Sep 16;22(9):1440. doi: 10.3390/ijerph22091440 (PMC12470198; doi:10.3390/ijerph22091440)
Supplement: Supplementary file 1 [file ijerph-22-01440-s001.zip › Supp Doc S4 Northwoods Example.pdf]

Evaluation Worksheet Example for Northwoods Study Area

Part I. Site Evaluation Worksheet

**Site Name:** Lost Lake Recreation Area						
**Location:** 4450 Chipmunk Rapids Rd, Long Lake, WI 54542						
**Site Type:** rural national forest recreation area						
**Site Info:** https://www.fs.usda.gov/recarea/cnnf/recarea/?recid=27877; https://www.fs.usda.gov/recarea/cnnf/recarea/?recid=27875 https://www.fs.usda.gov/Internet/FSE_DOCUMENTS/fseprd529735.pdf						
**Summary Scores**	**Beauty**	**Integrity**	**Tranquility**	**Accessibility**	**Total**	**Pct**
**Low = 1, Moderate = 2, High = 3**	**3**	**3**	**3**	**3**	**12**	**100**

1. **Landscape Character and History-** The 880 ac Lost Lake Recreation Area in the Chequamegon-Nicolet National Forest provides a variety of facilities for nature-based recreation in a relatively pristine setting centered around 84 ac Lost Lake. Landform patterns include collapsed moraines and outwash plains with bedrock knolls and ridges; soils are silt loams on gentle to moderate slopes surrounding Lost Lake and sandy loams on a 50' high ridge, a braided esker, that runs the length of the western edge of the site. Besides Lost Lake, there are a number of smaller lakes and wetlands on the site, and the Pine River, a state-designated Wild River, forms the northern border of the site. The site's original tree vegetation was hemlock-hardwood; an impressive old growth stand (150+ years) of nearly pure hemlocks still exists along much of the shore around Lost Lake, and large hemlocks and hardwoods (mainly maples and yellow birch) are present along the ridge. Native American presence in the area goes back at least 4000 years, and an archaeological village site at nearby Fay Lake dates to Oneota Culture of the Late Woodland Period of 1000-1499 AD. The site was part of Ojibwe tribal territory that was ceded to the US in 1842 and after the General Land Office survey in the 1850s the land around Lost Lake was sold to timber and railway companies. Much of the site was logged in the early 1900s and a major wildfire, the Tipler Fire, went through parts of the area in 1931. The Nicolet National Forest (now Chequamegon-Nicolet or CNNF) was first established in 1928 and lands in and around Lost Lake were purchased in 1936. In 1938, the Lost Lake Organization Camp was built along the south shore of the lake by the Civilian Conservation Corps for use as a group summer camp, with a central lodge/kitchen and group sleeping cabins. After periods of different uses and vacancy it was restored and reopened in 2011 for individual cabin rentals operated under a concession. Besides the CCC camp, the Lost Lake Recreation Area includes a boat landing for non-motorized boating, a 27-unit campground, and a network of trails of varying lengths and degrees of difficulty, including the Assessor's Interpretive Trail,

named for the local tax assessor. Legend has it that when this assessor came to assess the lands around Lost Lake in the 1920s, he persuaded the owner of the timberlands to save the virgin hemlocks around the lake in exchange for a promise not to increase the assessment fee.

2. **Beauty-** The scenic beauty of this site is high and early development as a recreation area and full complement of facilities makes it one of the more popular visitor destinations in the Florence portion of the CNNF's Eagle River-Florence Ranger District, where it is managed under a high Scenic Integrity Objective (SIO). Prominent scenic features include an impressive old growth stand (150+ years) of nearly pure hemlock along much of the shore surrounding Lost Lake; a nearly continuous 50' high ridge, a braided esker, that runs the length of the western edge of the site; a number of smaller lakes and wetlands visible along the trail network; the Pine River, a state-designated Wild River, which forms the northern border of the site; and the Lost Lake Organization Camp, a grouping of log buildings built by the CCC in 1938 that, along with the Assessor's Interpretive Nature Trail, are two of the Ranger District's major cultural sites.

3. **Integrity-** Ecological integrity of the site is high overall, with high water quality in Lost Lake and other water bodies on the site and high-quality upland and lowland forests along the Ridge Trail. Areas along the lakeshore have some groundcover and soil erosion due to trail use under the dense hemlock canopy and areas leading down to the lake from the campground and the CCC Organization Camp. The CCC Camp has undergone significant restoration and maintenance in 2011 and more recently to keep the buildings in good condition and recreational uses of the area are compatible with maintaining the site's cultural integrity. The site is managed by the CNNF under a high Scenic Integrity Objective (SIO).

4. **Tranquility-** Tranquility overall is high as the site is large, distant from major roads, and low in use, though there may be sounds of campers on peak summer weekends around campsite and camp as well as a few encounters with other trail users on the Lakeshore Trail Loop.

**Setting**	880 ac site surrounded by national forest and state forestry lands.
**Visual**	Visual intrusions are negligible and development (CCC cabins and campground, beaches, boats (few)) that is visible at a few points along the lakeshore trail is part of the site's recreational infrastructure and is visually compatible with the forest therapy experience.

**Sound**	The site is 2.24–3.42 mi from a low use state highway (850 AADT Hwy 70 and the site access road is very low use (60 AADT Chipmunk Rapids Rd.) Composite anthropogenic sound impact rating is estimated as Quiet to Very Quiet and mainly low use non-motorized recreation.
**Other**	None.
**Social**	Average use levels are very low and low on peak summer weekends. Use is mostly nature oriented, hiking. Loop Trail has a 27-unit campground, boat landing, and picnic grove along the south shore and primitive rental cabins (8) along the west shore. Lake has non-motorized boats only but in winter snowmobiles and power ice drills are permitted (very low use).
**Environmental**	Minor trail challenges include rocks and exposed roots, uneven stretches; wet areas may be buggy and grassy areas may have ticks. Ridge Trail has steep stretches.

5. **Accessibility-** Accessibility is high; drive time to the main trailhead is 27 min and located on a well-maintained paved/dirt forest road 4 mi off a state highway. Trailhead parking is usually plowed in winter and includes an all-season outhouse. There is a $5 daily use fee. Six land routes and one water route are identified, with land route options for length and degree of difficulty ranging from 0.9–4.2 mi, grade easy to very steep, avg width 2–4', surface all mostly dirt. The 1.3 mi water trail is an easy paddle on a small lake with a good boat landing.

**Proximity**	21.3 mi/27 min drive from the Wild Rivers Interpretive Center
**Facilities**	Trailhead parking lots (3); lots and access road are sometimes not plowed in winter. Non-motorized boat landing has public parking and an all-season outhouse. Lodging includes 8 seasonal historic CCC rental cabins with lodge, shower facilities, group meeting/rec hall, swimming beach, and boat rental (for cabin guests only), and a 27-unit seasonal campground with fire pits, picnic tables, and outhouse but no electricity or water. There is also a separate picnic area with fire pits.
**Fees**	$5 daily per car fee or annual pass good for all National Forest fee areas; cabin and campsite rentals available.
**Options**	Six land routes and one water route are identified, with land route options for length and degree of difficulty ranging from 0.9 – 2.6 mi, grade easy to very steep, avg. width 2-4', surface all mostly dirt. For easiest stretches, roots and uneven tread would pose barriers for wheelchair users, except perhaps for the segment of the Assessor's Trail that follows an old railroad grade (though with assistance in

	case of obstructions). 1.3 mi water trail is an easy paddle on a small lake with a good boat landing.

Part II. Trail Level Criteria

**Trail:** Lakeshore Trail Loop					**Type:** Foot			
**Trail info:** https://www.fs.usda.gov/recarea/cnnf/recarea/?recid=27875								
**N visits:** 6					**Seasons:**	**W**	**Sp**	**Su** / **F**
**Score**	**Ease of Travel**	**Attractive Layout**	**Natural Features**	**Built Features**	**Explorable Nature**	**Interp.**	**Total**	**Pct**
**L/M/H 1/2/3**	**2**	**3**	**3**	**3**	**3**	**3**	**17**	**94**

Part II A. Design and Construction

6. **Ease of Travel-** High

**Trailhead Distance**	0.0 mi. from either of 3 trailheads.
**Length**	1.6 mi.
**Surface**	Dirt, some stretches with moss, hemlock needles on trailbed and ROW.
**Width**	2–3 ft, 3 ft modal, with some wider open areas.
**Slope**	15% max, 3% avg.
**Accessibility Barriers**	Easy grade but dirt, roots, and rocks with uneven surface may present challenges.

7. **Attractiveness of Layout-** High

**Alignment**	Gently winding, mostly level.
**Route Type**	2-way loop.
**Views**	Detail views of moss and fungi, deeply enclosed conifer (hemlock) forest, closed canopy hardwood forest, ground level distant and panoramic lake views at several locations along the route.
**Spaces**	Rocks and fallen logs provide sit spots with lake views; also two log benches at lake overlook by Assessor's trail junction. There are a number of more open areas for groups to come together along the trail, and there are also formal group areas at the CCC camp, campground, and picnic area by boat landing.
**Changes**	Easy grade with little elevation change, stretches with moss and spongy forest floor for barefoot walking, forest cover from boat landing to CCC camp relatively homogeneous but micro changes in the forest floor are the main attraction. There are more vegetation and spatial changes on other stretches including rocky point with

	northern white cedar, marshy area, and open sunny stretches along the lake.

Part II B. Key Trailside Features and Opportunities

8. **Natural Features** -High

**Vegetation Cover**	Old-growth (150 yr) hemlock stand, aquatic-riparian lake edge, mature mixed hardwood forest.
**Trees**	Many large hemlocks 20" dbh, some pines 30" and n white cedars 16"; continuous old growth rings at least 50% of lakeshore.
**Water**	Lost Lake 84 ac Dbc 1.36, relatively round with 1 wetland bay, lake is visually accessible immediate foreground at eye level for virtually the entire trail, and several physical access points including two developed sand swimming beaches by campground and CCC cabins. Water is clear and of high quality for swimming and other physical contact. Main sensory effect is wave motion.
**Wildlife**	Inquisitive red squirrels are commonly present in the hemlock area; also woodland birds, occasional eagle sighting, and seasonal ducks.
**Other**	Moss, distinctive exposed tree roots, fungi, ferns, ground flora, needles and spongy soil; large standing and down dead wood, large rocks along shore.

9. **Built and Borrowed Features-** High

**Seating**	Logs and rocks, wood benches by beach and picnic area with tables by boat landing; attractive log benches at overlook by Assessor's trail.
**Gateways**	Trailhead by boat landing into the hemlock forest provides a minimally identifiable visual gateway. Trailhead by Assessor's Trail trailhead is more defined with attractive entry sign at parking lot entrance.
**Shelter**	There is a lodge at the CCC camp with a handsome stone fireplace and a group activity building for organized group use.
**Other Features**	Campground and cabins at CCC Camp both have sites with picnic tables and fire pits; toilet facilities at both locations. The CCC Organization Camp has historical significance and National Register of Historic Places nomination papers have been drafted but not submitted.

10. **Explorable Nature-** High

**Uses and Restrictions**	OK to harvest, go off trail, etc. Fire rings at picnic & campground sites.
**Museumification**	None

**On-Trail Engagement**	The trail corridor is level but moss, fungi and twisted roots in the ROW provide high interest and engagement at the detail level.

11. Interpretation and Stewardship- High

**Signage**	Trailhead informational kiosk, directional trail markers along route
**Learning and Stewardship Opportunities**	CCC camp area has nice interpretive kiosk and lodge has scrapbooks with articles and photos showing history of the site.

**Trail:** Assessor's Interpretive Trail					**Type:** Foot			
**Trail Info:** https://www.fs.usda.gov/recarea/cnnf/recreation/recarea/?recid=27949&actid=50								
**N visits:** 4					**Seasons:**	**W**	**Sp**	**Su**	**F**
**Score**	**Ease of Travel**	**Attractive Layout**	**Natural Features**	**Built Features**	**Explorable Nature**	**Interp.**	**Total**	**Pct**
**L/M/H 1/2/3**	**3**	**2**	**3**	**2**	**2**	**2**	**14**	**78**

Part II A. Design and Construction

6. Ease of Travel- High

**Trailhead Distance**	0.0 mi.
**Length**	0.9 mi.
**Surface**	Mainly dirt, also some stretches with grass.
**Width**	2–3 ft, 3 ft modal, with some wider open areas.
**Slope**	15% max, 3% avg.
**Accessibility Barriers**	Easy grade but dirt, roots and rocks with uneven surface may present challenges. Trail segment that is an old railroad bed may be wheelchair accessible with assistance.

7. Attractiveness of Layout- Moderate

**Alignment**	45' elev change; straight and gently winding sections.
**Route Type**	2-way loop.
**Views**	Mainly closed canopy hardwood and hemlock forest types with open understory; ground level distant and panoramic lake views, long view along old RR grade.
**Spaces**	Two log benches overlooking lake make a good private or small group sitspot; some wider stretches along trail and at trailhead could be used for group invitation activities.
**Changes**	Easy grade with trail going through mature hardwood forest then hemlocks along the lake; some small openings but relatively

	homogeneous full canopy cover; long straightaway along old RR grade back to trailhead/parking lacks mystery.

Part II B. Key Trailside Features and Opportunities

8. **Natural Features** -High

**Vegetation Cover**	Mainly mature northern hardwood forest with small sections of old growth hemlock stand and riparian-aquatic lake zone.
**Trees**	Assessor's Tree was a towering old growth white pine (dbh > 36"), though the top half was broken off by a storm in the 2000s or 2010s; old growth hemlock in band along the lakeshore.
**Water**	Lost Lake 84 ac Dbc 1.36, is visually accessible foreground superior and immediate foreground eye level views along western 1/3 of the trail; physical access is possible at a few points but shoreline is not ideal. Water is clear and of high quality for swimming and other physical contact. Main sensory effect is wave motion.
**Wildlife**	None observed but red squirrels and woodland birds are common at site, along with occasional eagle sighting and seasonal ducks seen along lakeshore.
**Other**	Spring flora in hardwood zone, soft hemlock needles for barefoot walking.

9. **Built and Borrowed Features-** Moderate

**Seating**	Two log benches at overlook.
**Gateways**	Attractive Assessor's Trail Sign and info kiosk at trailhead entrance provides a defined physical gateway.
**Shelter**	None
**Other Features**	None

10. **Explorable Nature-** Moderate

**Uses and Restrictions**	OK to harvest, go off trail, etc. for exploration activities.
**Museumification**	No fencing or other symbolic barriers.
**On-Trail Engagement**	Flat terrain and long straight trail segment though homogeneous forest limits engagement except for a few key points including physical lake access.

11. **Interpretation and Stewardship-** High

**Signage**	Trailhead sign and map; several interpretive signs along the trail.
**Learning and Stewardship Opportunities**	Interpretive signs.

<table>
<tr><td colspan="5">Trail: Lakeshore-Ridge Trail Loop</td><td colspan="5">Type: Foot</td></tr>
<tr><td colspan="10">Trail info: https://www.fs.usda.gov/recarea/cnnf/recreation/recarea/?recid=27949&actid=50</td></tr>
<tr><td colspan="5">N visits: 4</td><td>Seasons:</td><td>W</td><td>Sp</td><td>Su</td><td>F</td></tr>
<tr><td>Score</td><td>Ease of Travel</td><td>Attractive Layout</td><td>Natural Features</td><td>Built Features</td><td>Explorable Nature</td><td colspan="2">Interp.</td><td>Total</td><td>Pct</td></tr>
<tr><td>L/M/H 1/2/3</td><td>2</td><td>3</td><td>3</td><td>3</td><td>3</td><td colspan="2">3</td><td>17</td><td>94</td></tr>
</table>

Part II A. Design and Construction

6. **Ease of Travel-** Moderate

**Trailhead Distance**	0.1 mi.
**Length**	2.6 mi.
**Surface**	Mainly grass-- dirt, grass, wooden boardwalk sections.
**Width**	2' modal width with wider open areas; 15' logging 2-track road.
**Slope**	31% max; 6.2% avg.
**Accessibility Barriers**	Lake section is easy grade but long steep stretches ascending and descending the ridge may present significant challenge for some.

7. **Attractiveness of Layout-** High

**Alignment**	86' elev change; variety of stretches from gently winding to hilly with superior views of forest to straight logging road.
**Route Type**	2-way loop.
**Views**	Closed canopy hardwood and hemlock forest types with open understory; ground level distant and panoramic lake views, unique superior 360-degree panoramic views of upland forest from ridge.
**Spaces**	Some nice sitspots along lake and ridge, and some open group spots to sit in grass in upland or on pine needles or logs along lake.
**Changes**	Easy grade along lake with steep grade going up to Ridge and knoll and down to wetland; veg change from enclosed hemlock to more open hardwood and open wetland areas as well as changes in views from ground level lake to superior views of wetland.

Part II B. Key Trailside Features and Opportunities

8. **Natural Features-** High

**Vegetation Cover**	Old-growth (150 yr) hemlock stand and aquatic-riparian lake edge along shore portion (30%), ridge and logging road portions (60%) mature mixed hardwood forest, minor wetland stretch (10%).
**Trees**	Old-growth (150 yr) hemlock stand along lake, some large hemlock, pine, and maple trees on Ridge Trail portion.

**Water**	Lost Lake 84 ac Dbc 1.36 visible immediate foreground eye level views on 30% of walk, with physical access at sand beach (CCC Camp) and a few rocky areas; wetland/bog boardwalk IFG at short low stretch along Ridge Trail between 6 ac Spruce Lake and 67 ac Grub Hoe Lake-- neither are visible from trail because of extensive dense conifer growth and surrounding wetlands.
**Wildlife**	Red squirrels, woodland birds.
**Other**	Moss, exposed tree roots, fungi, ferns, ground flora, needles and spongy soil; large standing and down dead wood, large rocks along shore; rocks on knoll, diverse ground flora in hardwood forest esp. springtime, a long stretch of trail along a narrow 50' high ridge (braided esker), a wetland/bog with tamaracks along Ridge Trail.

9. **Built and Borrowed Features-** High

**Seating**	Logs and rocks, benches by beach and picnic area with tables by boat landing; log benches at overlook by Assessor's Trail.
**Gateways**	Multiple entry points; sign and trailhead at Assessor's Trail parking lot has best defined gateway.
**Shelter**	There is a lodge at the CCC camp and a group activity building for organized group use.
**Other Features**	Campground and Cabins at CCC Camp both have sites with picnic tables and fire pits, swimming beach; CCC cabins also have boat rentals. Toilet facilities at both locations.

10. **Explorable Nature-** High

**Uses and Restrictions**	OK to harvest, go off trail, etc.
**Museumification**	None.
**On-Trail Engagement**	This longer loop trail has many engaging sections including the lake stretch described above followed by a narrow winding trail along the 50' high ridge/esker, then down through a lowland forest along a boardwalk. Good opportunities for engagement and interactivity including barefoot walking on ridge. Fire rings at picnic & campground sites.

11. **Interpretation and Stewardship-** High

**Signage**	Only directional trail markers along route.
**Learning and Stewardship Opportunities**	CCC camp area has a nice interpretive kiosk and lodge has scrapbooks with articles and photos showing history of the site.

**Trail:** CCC Cabins to Ridge Trail Loop					**Type:** Foot			
**Trail info:** https://www.fs.usda.gov/recarea/cnnf/recarea/?recid=27877								
**N visits:** 6					**Seasons:**	**W** / **Sp**	**Su**	**F**
**Score**	**Ease of Travel**	**Attractive Layout**	**Natural Features**	**Built Features**	**Explorable Nature**	**Interp.**	**Total**	**Pct**
**L/M/H 1/2/3**	**3**	**3**	**3**	**3**	**3**	**3**	**18**	**100**

Part II A. Design and Construction

6. **Ease of Travel-** High

**Trailhead Distance**	0.10 mi.
**Length**	0.90 mi.
**Surface**	Dirt and grass
**Width**	2–4 ft, 2 ft modal, with some wider open areas and a portion that is unmarked.
**Slope**	20% max, 4.5% avg.
**Accessibility Barriers**	Easy grade but dirt, roots and rocks with uneven surface and some steep stretches may present challenges.

7. **Attractiveness of Layout-** High

**Alignment**	64 ft elev. change; gently winding trail along lakeshore to mature interior forest and open wetland/sedge meadow.
**Route Type**	2-way loop.
**Views**	Closed canopy hardwood and hemlock forest types with open understory; ground level distant and panoramic lake views, superior view of wetland from knoll KOP and panoramic view from wetland edge by giant white pine KOP.
**Spaces**	Some nice sitspots along lake, on knoll for private or group space.
**Changes**	Easy grade along lake with moderate grade going up to knoll and down to wetland; veg. change from enclosed hemlock to more open hardwood and open wetland areas as well as changes in views from ground level lake to superior views of wetland.

Part II B. Key Trailside Features and Opportunities

8. **Natural Features-** High

**Vegetation Cover**	Old-growth (150 yr) hemlock stand, aquatic-riparian lake edge, mature mixed hardwood forest, and wetland.

**Trees**	Many large hemlocks by lake, majestic white pine by wetland and some large hardwoods in upland forest on knoll overlooking the wetland.
**Water**	Lost Lake 84 ac Dbc 1.36 visible immediate foreground eye level views on first and last 25% of walk, with physical access at sand beach (CCC Camp) and a few rocky areas, 8 ac wetland-sedge meadow visible FG superior and IFG eye level views from KOPs at midpoint of trail; lake water movement sensory effect.
**Wildlife**	None observed but similar to other lakeshore stretches above.
**Other**	Moss, exposed tree roots, fungi, ferns, ground flora, needles and spongy soil; large standing and down dead wood, large rocks along shore; rocks on knoll, diverse ground flora in hardwood forest esp. springtime.

9. **Built and Borrowed Features-** High

**Seating**	Logs and rocks, benches by beach.
**Gateways**	Informal, begin at CCC camp trailhead.
**Shelter**	Trailhead- there is a lodge at the CCC camp and a group activity building for organized group use.
**Other Features**	Trailhead- cabins at CCC Camp with fire pits and boat rental, restrooms.

10. **Explorable Nature-** High

**Uses and Restrictions**	OK to harvest, go off trail, etc.
**Museumification**	None
**On-Trail Engagement**	This shorter loop trail includes the lake stretch described above followed by a narrow winding trail through open interior forest with large diameter trees to a wetland edge. High engagement.

11. **Interpretation and Stewardship-** High

**Signage**	Directional trail markers along route.
**Learning and Stewardship Opportunities**	Camp area has nice interpretive kiosk and lodge has scrapbooks with articles and photos showing history of the site.

**Trail:** West Lakeshore Trail					**Type:** Foot			
**Trail info:** https://www.fs.usda.gov/recarea/cnnf/recarea/?recid=27875								
**N visits:** 6					**Seasons:**	**W** **Sp**	**Su**	**F**
**Score**	**Ease of Travel**	**Attractive Layout**	**Natural Features**	**Built Features**	**Explorable Nature**	**Interp.**	**Total**	**Pct**
**L/M/H 1/2/3**	**3**	**3**	**3**	**3**	**3**	**3**	**18**	**100**

Part II A. Design and Construction

6. **Ease of Travel-** High

**Surface**	Mainly dirt, some moss sections.
**Trailhead Distance**	0.0 mi.
**Length**	1.0 mi.
**Width**	3 – 4 ft width with wider open areas, 3 ft modal.
**Slope**	10% max; 2% avg.
**Accessibility Barriers**	Easy grade but dirt, roots and rocks with uneven surface may present challenges.

7. **Attractiveness of Layout-** High

**Alignment**	16' elev change; gently winding lakeshore path through open old-growth hemlock forest with lake views and access.
**Route Type**	2-way linear (there and back).
**Views**	Detail views of moss and fungi, deeply enclosed conifer forest, interior views to mixed hardwood forest, ground level lake views.
**Spaces**	Rocks and fallen logs for private sit spots; several more open areas for groups to come together; also group areas at the CCC camp and picnic ground by boat landing.
**Changes**	Easy grade with little elevation change, stretches with moss and spongy forest floor for barefoot walking, forest cover from boat landing to CCC camp relatively homogeneous but micro changes in the forest floor are the main attraction.

Part II B. Key Trailside Features and Opportunities

8. **Natural Features-** High

**Vegetation Cover**	Old-growth (150 yr) hemlock stand, aquatic-riparian lake edge, hardwood interior views entire stretch.
**Trees**	Many large hemlocks, some big pines, too.
**Water**	Lost Lake 84 ac Dbc 1.36 physical access to lake at boat landing and CCC Cabins; continuous eye-level visual access IFG to lake along entire trail; water movement.
**Wildlife**	Red squirrels, woodland birds.

**Other**	Moss, exposed tree roots, fungi, ferns, ground flora, needles and spongy soil; large standing and down dead wood, large rocks along shore.

9. **Built and Borrowed Features-** High

**Seating**	Logs and rocks, benches by beach and picnic tables by boat landing.
**Gateways**	Trailhead by boat landing into the hemlock forest provides a visual gateway.
**Shelter**	There is a lodge at the CCC camp and a group activity building for organized group use.
**Other Features**	Cabins at CCC Camp, with fire pits, boat rental.

10. **Explorable Nature-** High

**Uses and Restrictions**	OK to harvest, go off trail, etc.
**Museumification**	None
**On-Trail Engagement**	Moss, fungi and twisted roots in the ROW provide high interest and engagement at the detail level; ideal stretch for foraging for fungi and ramps, playscapes for children at nearby CCC cabins with roots and fallen branches.

11. **Interpretation and Stewardship-** High

**Signage**	Informational, directional
**Learning and Stewardship Opportunities**	CCC camp area has nice interpretive kiosk and lodge has scrapbooks with articles and photos showing history of the site.

**Trail:** Lost Lake Paddle					**Type:** Paddle			
https://apps.dnr.wi.gov/doclink/lakes_maps/0588000a.pdf								
**N visits:** 1 (plus 1 winter walk across lake surface)					**Seasons:**	**W** / **Sp**	**Su**	**F**
**Score**	**Ease of Travel**	**Attractive Layout**	**Natural Features**	**Built Features**	**Explorable Nature**	**Interp.**	**Total**	**Pct**
**L/M/H 1/2/3**	**3**	**2**	**3**	**2**	**2**	**1**	**15**	**83**

Part II A. Design and Construction

6. **Ease of Travel-** High

**Trailhead Distance**	0.0 mi.
**Length**	1.3 mi.

**Surface**	Flat water; lake bottom along shore is 55% muck, 25% sand, 20% gravel.
**Width**	NA
**Slope**	NA
**Accessibility Barriers**	Easy paddle on small lake; some submerged trees along shore.

7. **Attractiveness of Layout-** Moderate

**Alignment**	Water trail along shoreline of this relatively round lake 84 ac D(bc)=1.36 offers views into forested upland and distant hills.
**Route Type**	2-way shoreline loop.
**Views**	Open panoramic views of water; detail views of shore and distant views of hills.
**Spaces**	Several places to get out for shoreline sitspots or group activity
**Changes**	Shoreline shape is relatively simple but with shoreline type changes include wetland, rocky, sandy areas; detail, distant and panorama views.

Part II B. Key Trailside Features and Opportunities

8. **Natural Features-** High

**Vegetation Cover**	Shore cover is old-growth (150 yr) hemlock stand, aquatic-riparian lake edge, mature mixed hardwood forest.
**Trees**	Many large hemlocks visible along shore; also some large maples, northern white cedars and a few supercanopy pines.
**Water**	84 ac clear water lake w/ sand/rocky shore and relatively simple shoreline (Dbc 1.36); small associated wetland area; physical access IFG eye level entire route; several places to get out for shore/water contact including 2 sand swimming beaches.
**Wildlife**	Loons, eagle, other waterfowl.
**Other**	Large rocks on south shore, fallen trees, reeds, wetland area.

9. **Built and Borrowed Features-** Moderate

**Seating**	Logs and rocks, picnic tables by boat landing.
**Gateways**	Boat landing.
**Shelter**	None.
**Other Features**	Fire pits at picnic area (?), all-season outhouse at boat landing.

10. **Explorable Nature-** Moderate

**Uses and Restrictions**	Harvesting OK on land, fishing as per standard fishing regulations.

**Museumification**	None.
**On-Trail Engagement**	Relatively simple shoreline somewhat reduces opportunities for exploration by boat, although upland shore and sandy/rock bottom provide several places to get out along the shore as well as for swimming.

11. Interpretation and Stewardship- Low

**Signage**	Informational kiosk at boat landing.
**Learning and Stewardship Opportunities**	No.

Part III. Statistical Summary, Map and Photos

Site Name and Scores	Beauty	Integrity	Tranquility	Accessibility	Total	Avg.
Lost Lake Recreation Area	3	3	3	3	12	100%

Nbr	Trail Name	Type	Length (km)	Ease	Layout	Natural	Built	Explore	Interp.	Total	Pct.
1	Lakeshore Trail Loop	Foot	2.6	2	3	3	3	3	3	17	94%
2	Assessor's Interpretive Trail Loop	Foot	1.5	3	2	3	2	2	2	14	78%
3	Lakeshore-Ridge Trail Loop	Foot	4.2	2	3	3	3	3	3	17	94%
4	CCC Cabins to Ridge Trail Loop	Foot	1.5	3	3	3	3	3	3	18	100%
5	West Lakeshore Trail	Foot	1.6	3	3	3	3	3	3	18	100%
6	Lost Lake Paddle	Paddle	1.1	3	2	3	2	2	1	13	72%

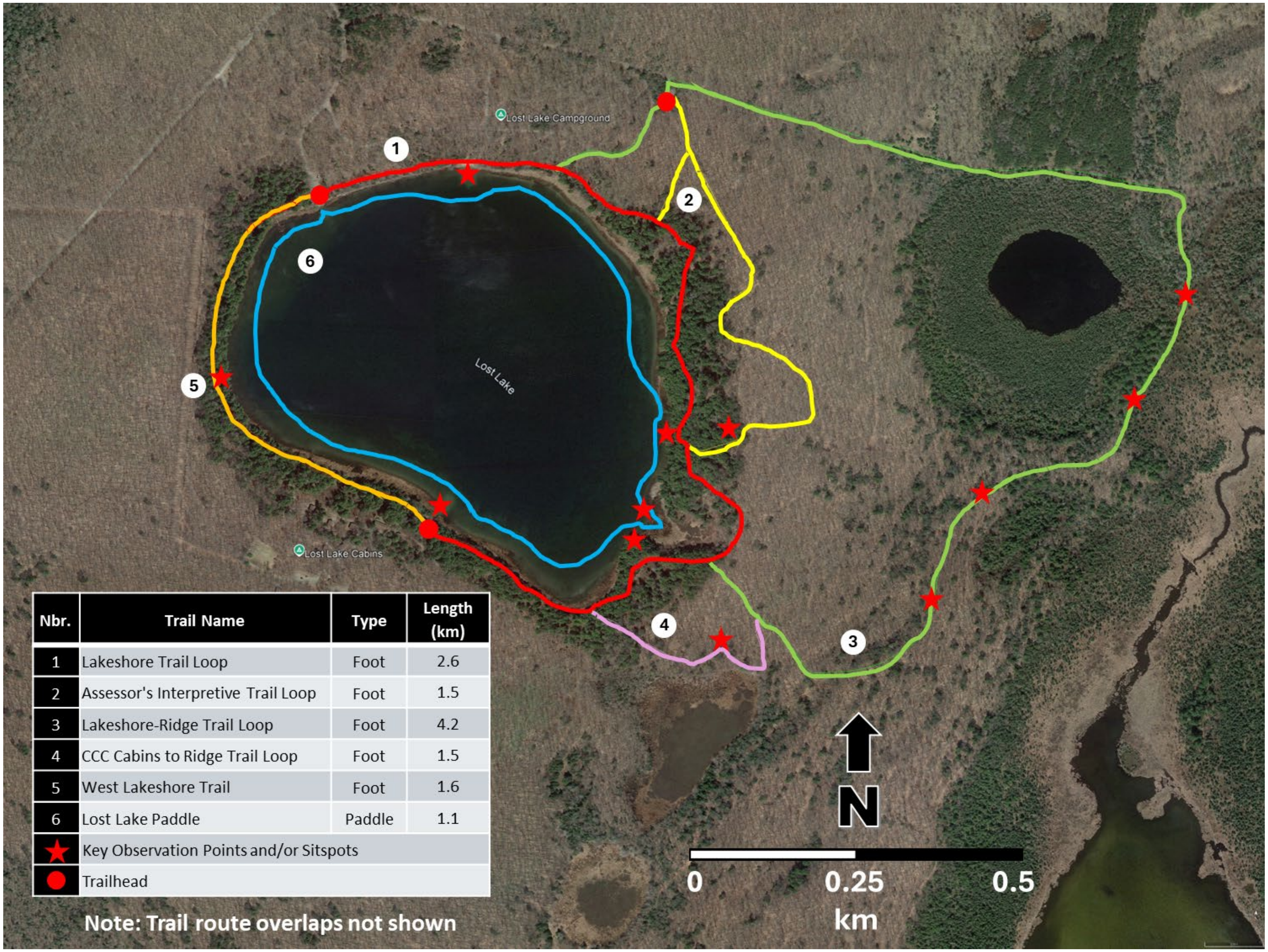

Nbr.	Trail Name	Type	Length (km)
1	Lakeshore Trail Loop	Foot	2.6
2	Assessor's Interpretive Trail Loop	Foot	1.5
3	Lakeshore-Ridge Trail Loop	Foot	4.2
4	CCC Cabins to Ridge Trail Loop	Foot	1.5
5	West Lakeshore Trail	Foot	1.6
6	Lost Lake Paddle	Paddle	1.1
★	Key Observation Points and/or Sitspots		
●	Trailhead		

Figure 1. Lost Lake Recreation Area Site Map showing trail network. Base map Google Earth, image 05/09/2014.

1. Lakeshore Trail Loop

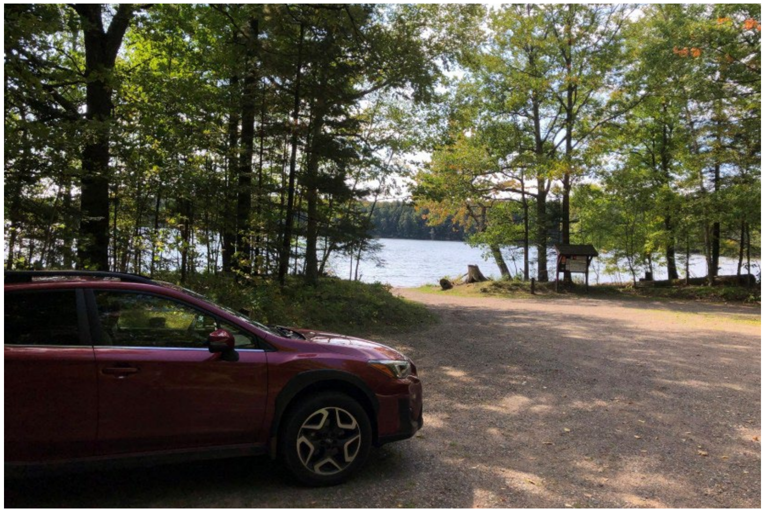

1a. Trailhead parking, outhouse and boat landing.

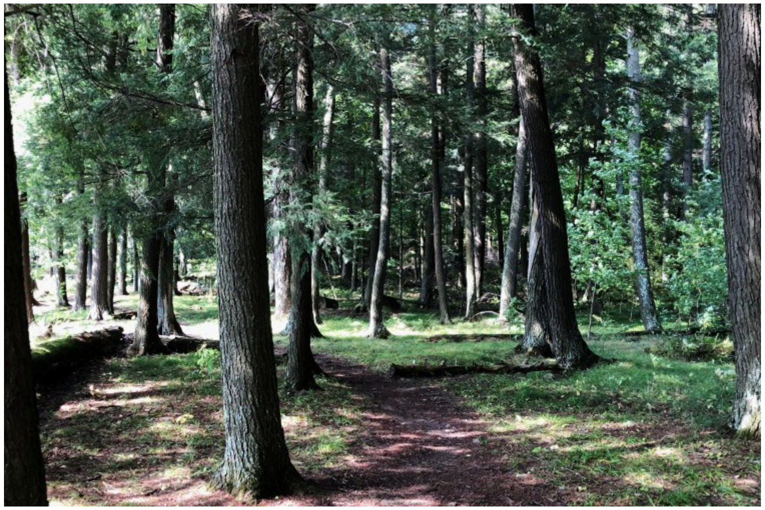

1b. Typical trail profile.

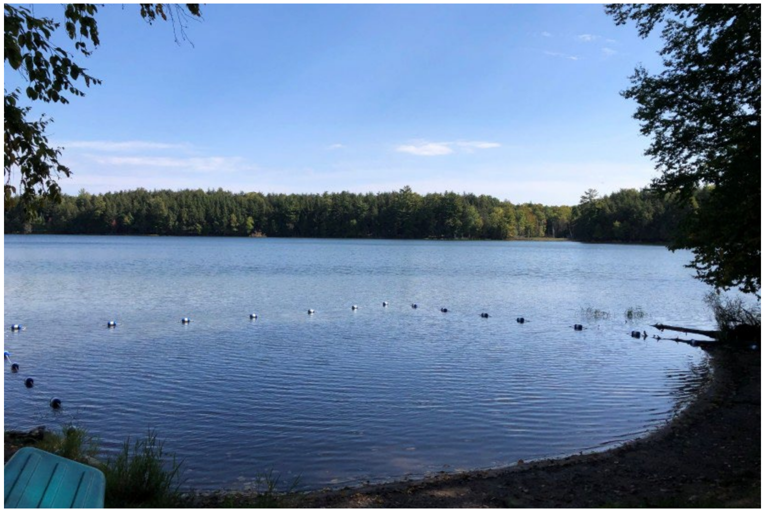

1c. Swimming beach sitspot key observation point.

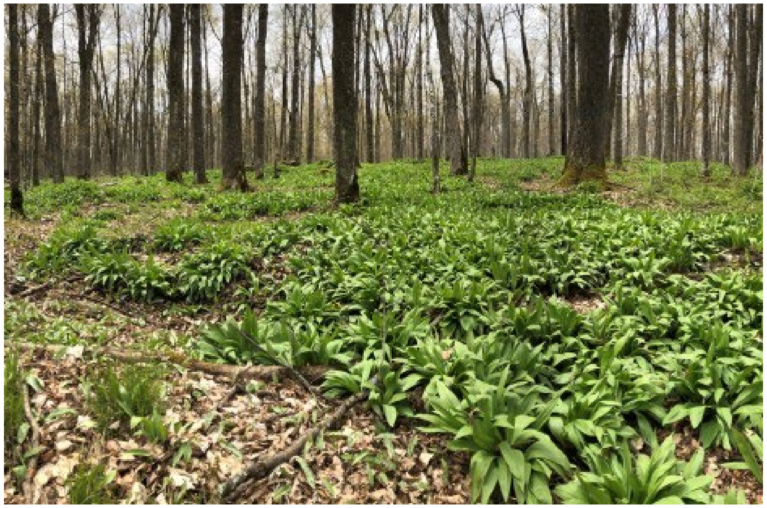

1d. Interior forest view, spring ramp foraging area.

2. Assessor's Interpretive Trail Loop

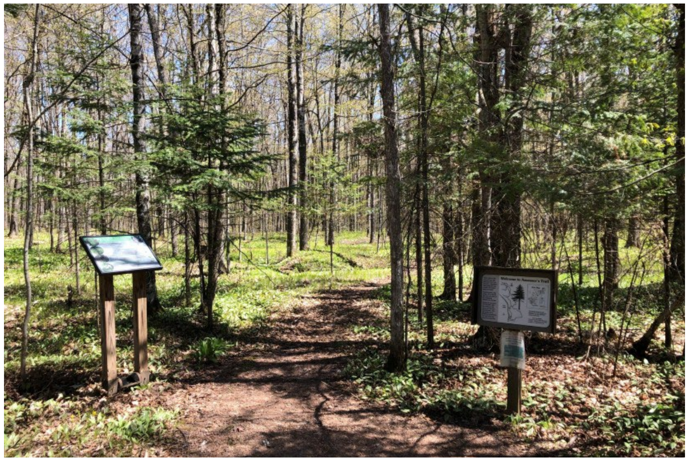

2a. Trailhead gateway with interpretive signage.

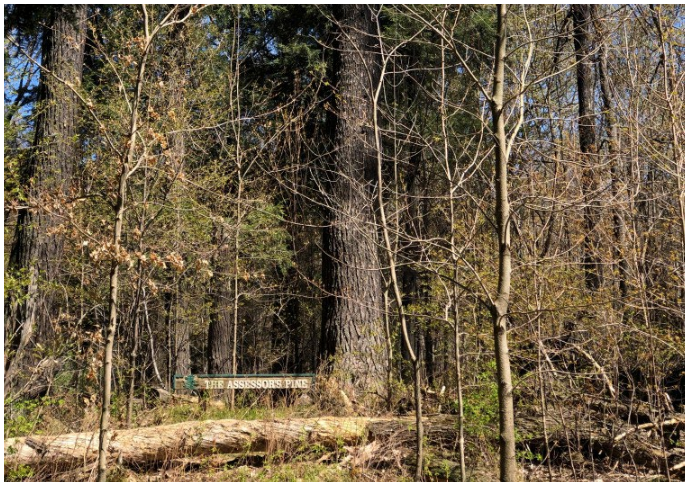

2b. Assessor’s Pine key natural feature.

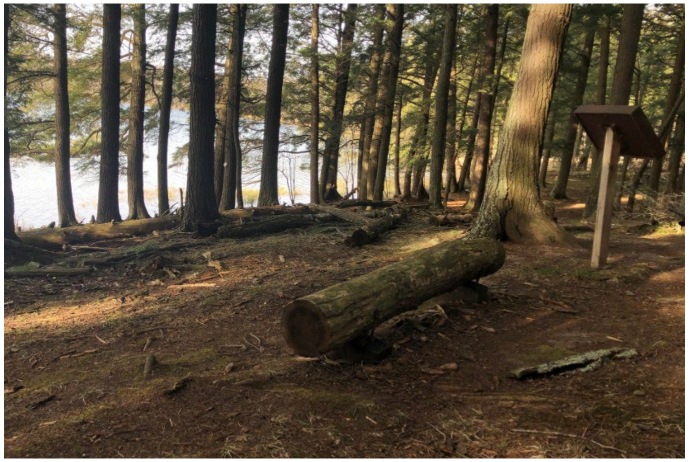

2c. Interpretive sign and log sitspot key observation point.

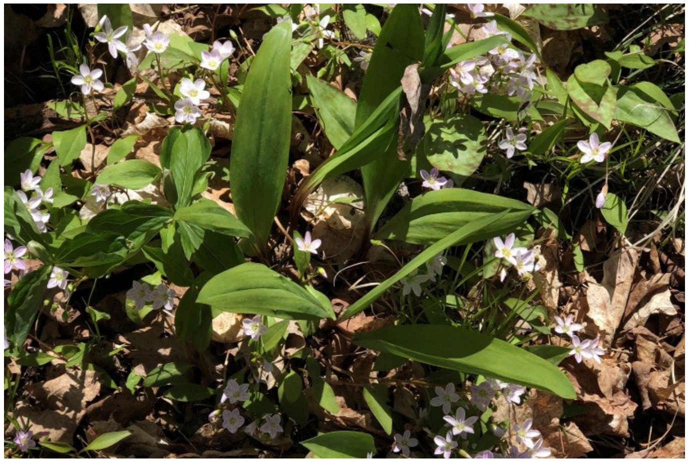

2d. Spring wildflowers detail/ephemeral landscape.

3. Lakeshore-Ridge Trail Loop

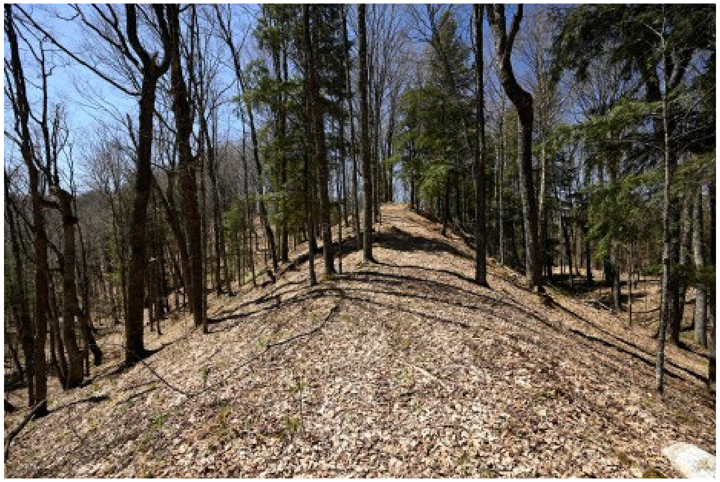

3a. Ridge trail along braided esker.

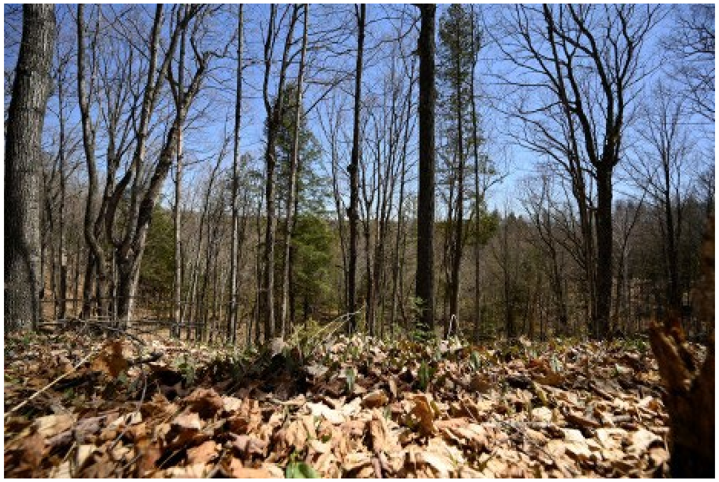

3b. Leaf-off distant view key observation point.

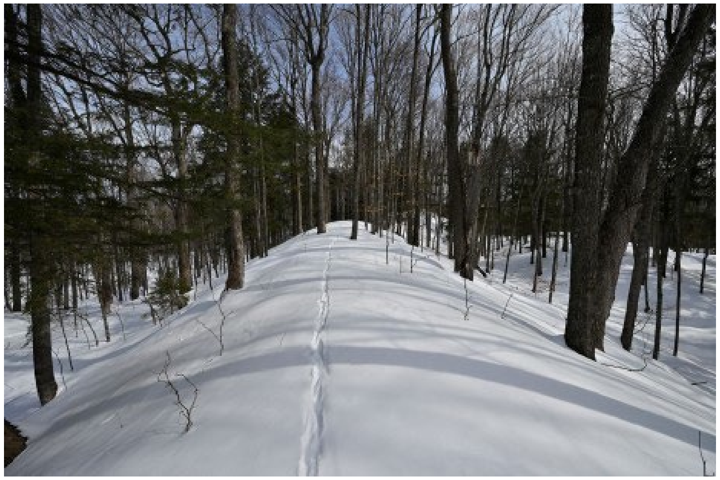

3c. Ridge trail winter view.

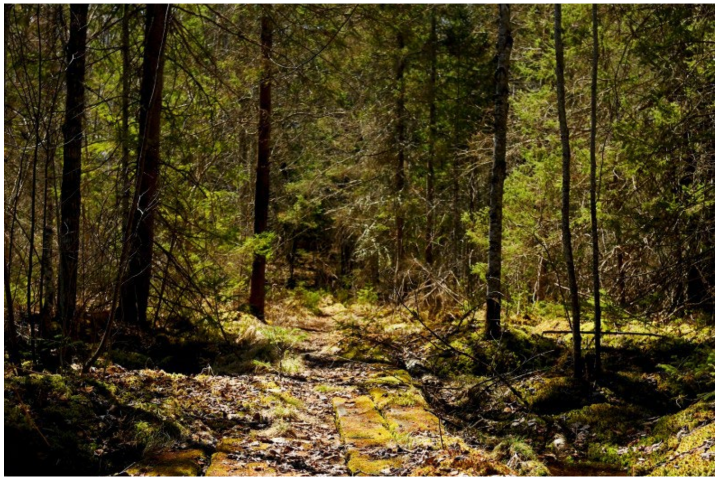

3d. Wetland boardwalk key observation point.

4. CCC Cabins to Ridge Trail Loop

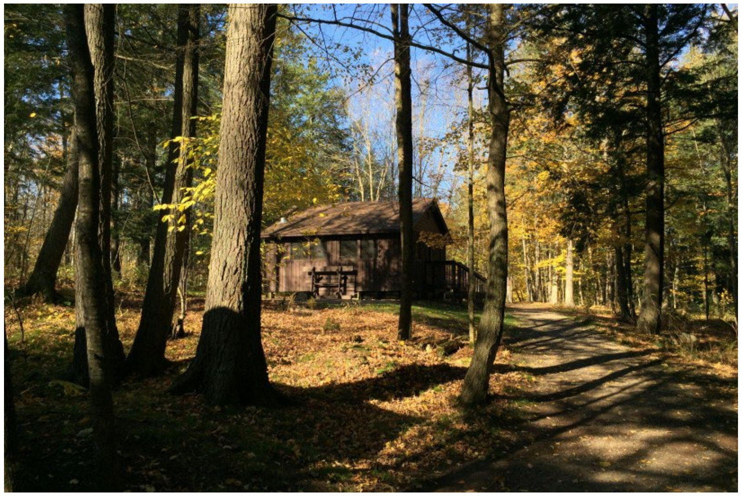

4a. CCC Camp rental cabin.

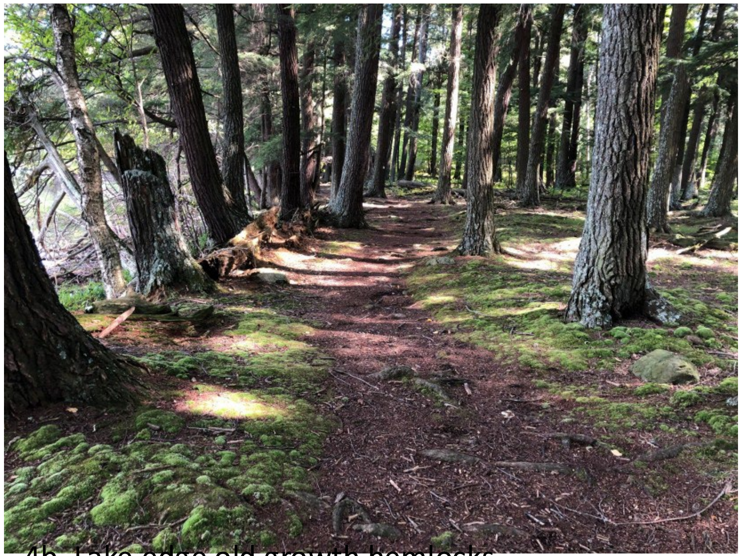

4b. Lake edge old growth hemlocks.

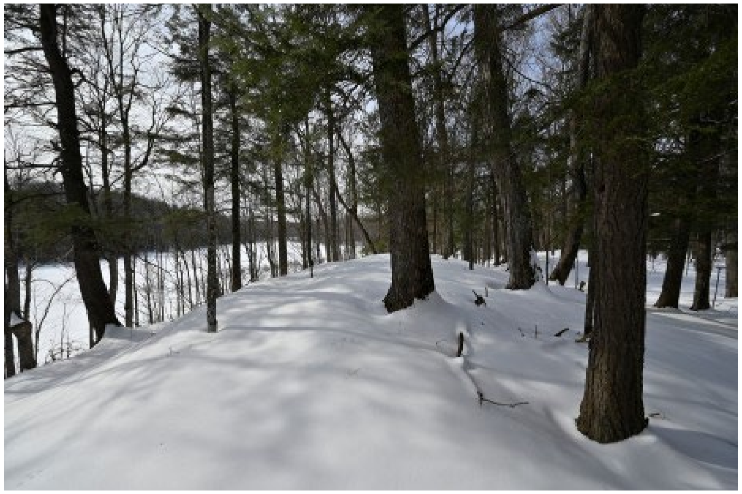

4c. Hilltop sitspot-- wetland overlook key observation point.

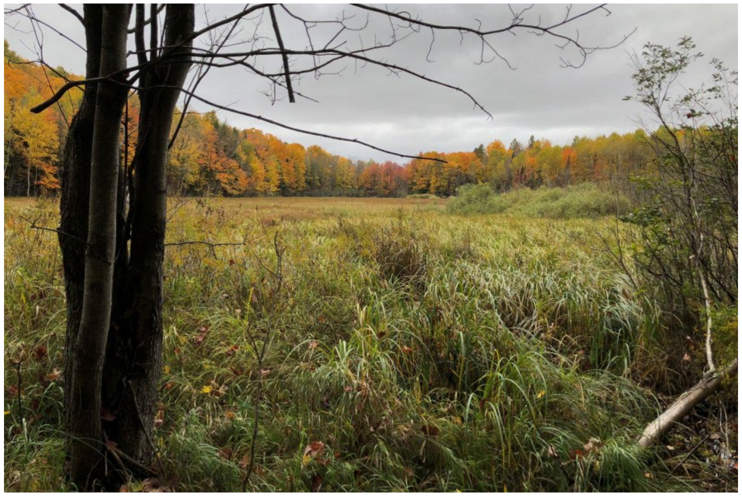

4d. Same wetland at eye level view.

5. West Lakeshore Trail

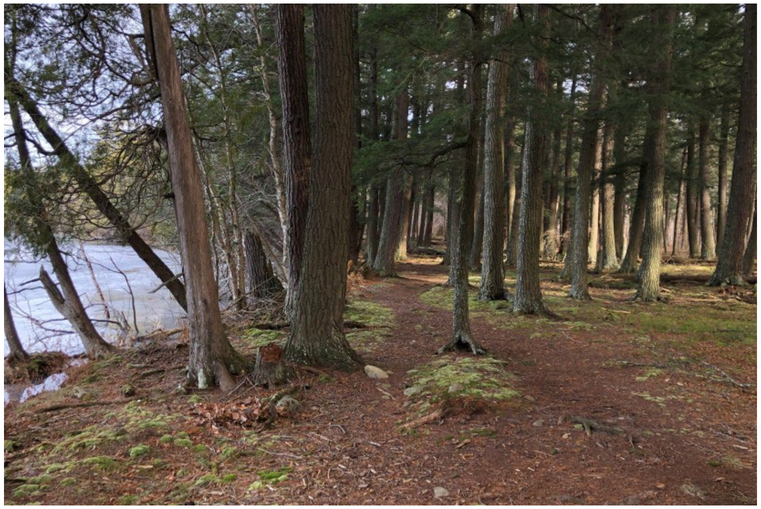

5a. Needle covered path through old growth hemlocks.

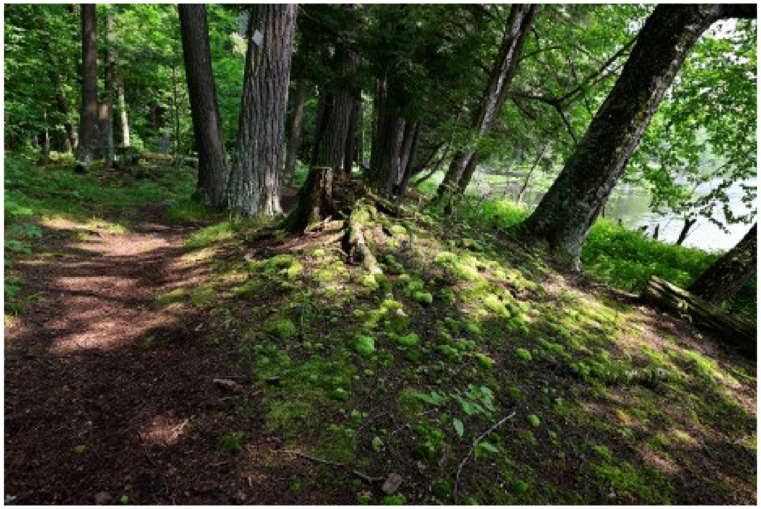

5b. Mossy trail right of way.

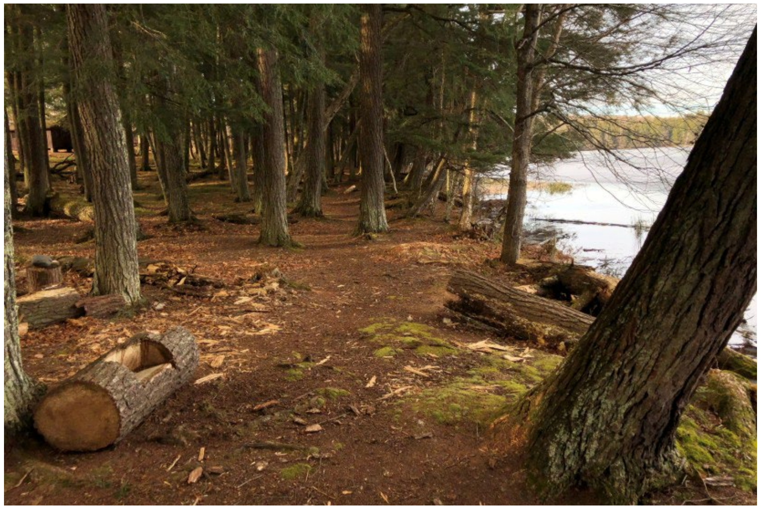

5c. Log sitspot key observation point.

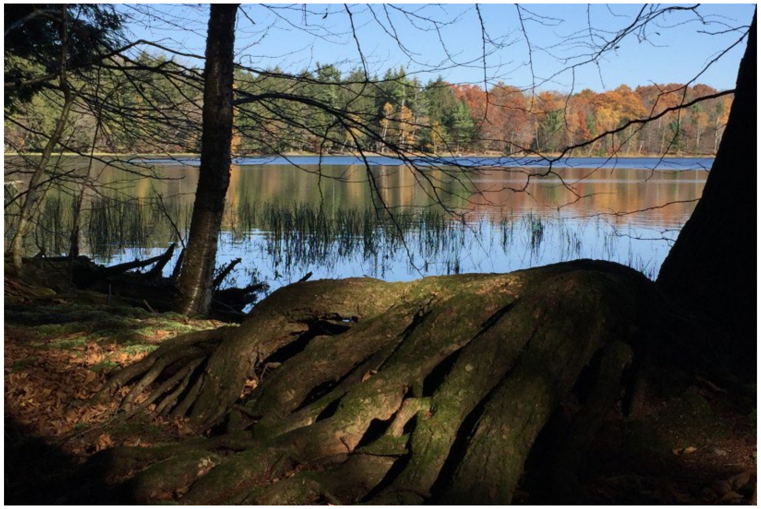

5d. Tree roots part of detail landscape.

6. Lost Lake Paddle

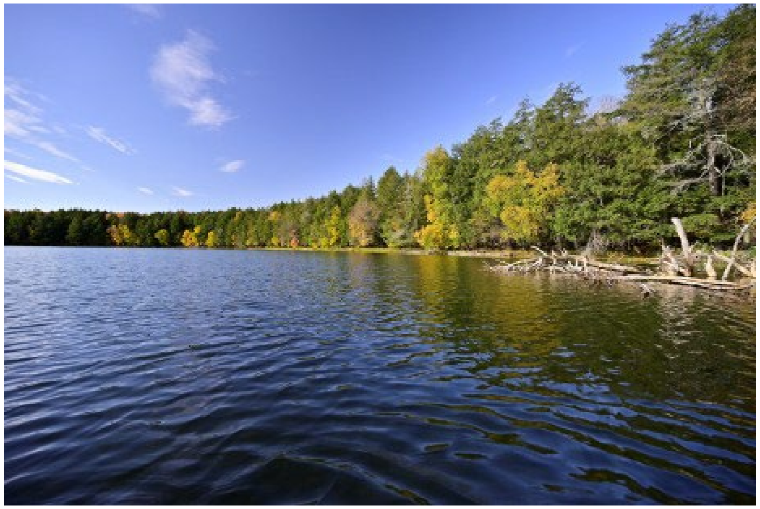

6a. Typical shoreline edge.

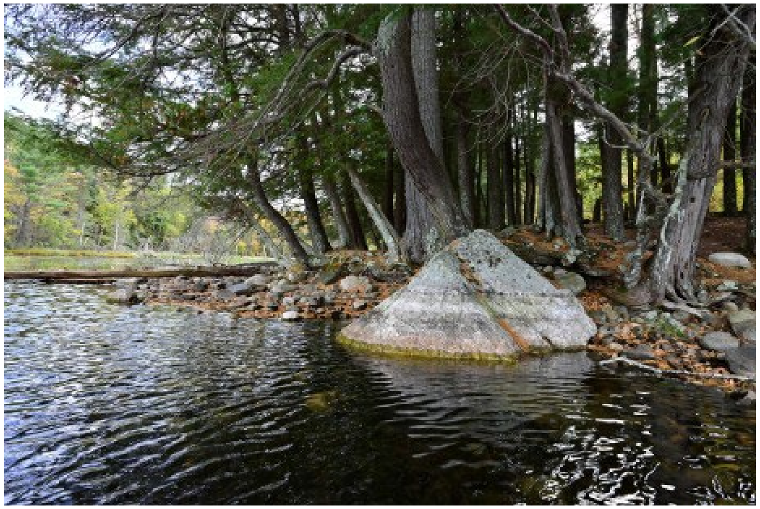

6b. Rocky point key observation point.

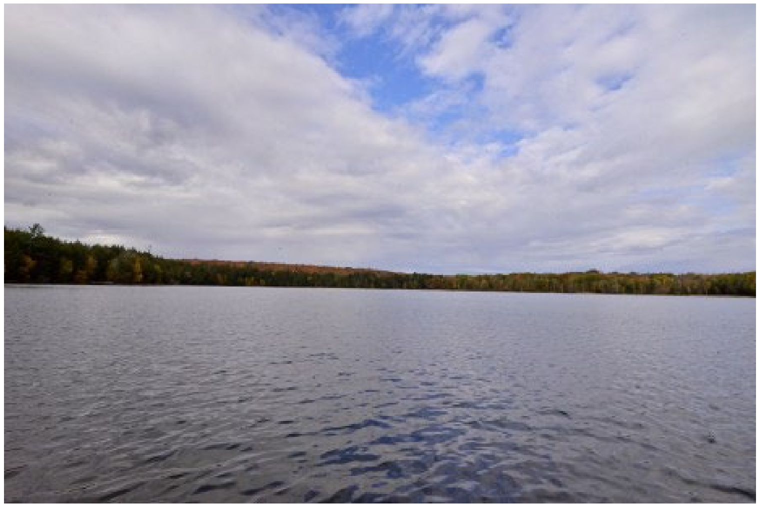

6c. Distant, panoramic views.

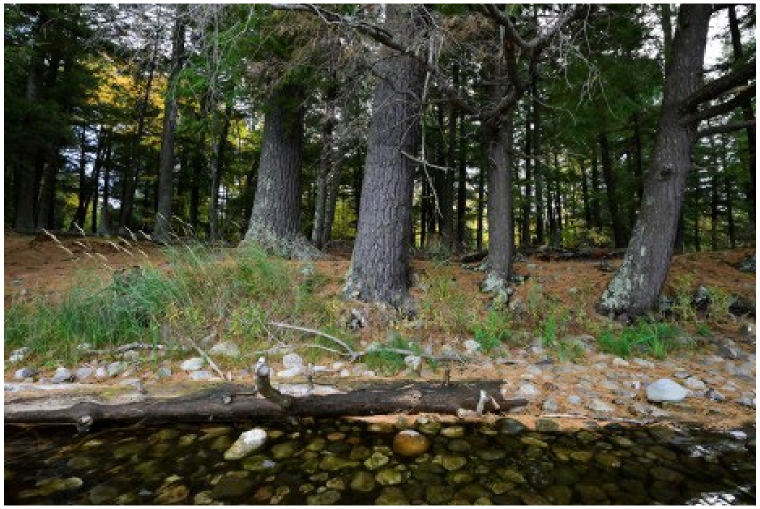

6d. Old growth trees along shore.
