# Supplementary material for: Forest Therapy Trails: Development and Application of an Assessment Protocol
Source: Int J Environ Res Public Health. 2025 Sep 16;22(9):1440. doi: 10.3390/ijerph22091440 (PMC12470198; doi:10.3390/ijerph22091440)
Supplement: Supplementary file 1 [file ijerph-22-01440-s001.zip › Supp Doc S5 Chicago Example.pdf]

Evaluation Worksheet Example for Chicago Study Area

Part I. Site Evaluation Worksheet

**Site Name:** Montrose Point						
**Location:** 200 W Montrose Harbor Dr, Chicago, IL 60640						
**Site Type:** urban park natural area						
**Online Info:** https://www.chicagoparkdistrict.com/parks-facilities/lincoln-park-montrose-point-bird-sanctuary						
**Summary Scores**	**Beauty**	**Integrity**	**Tranquility**	**Accessibility**	**Total**	**Pct**
**Low = 1, Moderate = 2, High = 3**	**3**	**2**	**3**	**3**	**11**	**92%**

1. **Landscape Character and History-** Montrose Point is a 28 ac section of 1188 ac Lincoln Park, the Chicago Park District's largest park, which occupies a continuous 7.5 mi ribbon of land along Chicago's north lakefront. It is part of the Chicago Lake Plain ecoregion, a nearly flat margin paralleling Lake Michigan originally part of the bed of glacial Lake Chicago. Original shoreland vegetation communities included wetland, wet prairie, dune, and sand beach ridges covered by scrub-oak vegetation. The Chicago region was rich in natural resources and for thousands of years was home to many Native American tribes, who inhabited permanent villages on inland uplands and had seasonal shoreland camps for fishing and other activities. The Pokagon Band of Potawatomi Indians held the Chicago region at the time of white exploration and initial settlement, ceding it in the 1833 Treaty of Chicago. In subsequent years the city grew rapidly, and Lincoln Park was created in several phases as submerged lands along the shore were filled to accommodate leisure needs and extend DuSable Lake Shore Drive as a major transportation artery. Montrose Point was designed in a naturalistic style in the late 1930s by noted Prairie School landscape architect Alfred Caldwell, but soon after, its 10 ac core was closed to the public for use as a WWII Nike missile base. When the base closed in the late 1960s the site received little park management attention or recreational use, and due to its prominent location along the lake it evolved into an important migratory bird stopover, especially along an old fenceline which became known as the Magic Hedge. Formal consideration of the site as a natural area began in the 1990s under a park master planning effort, and initial restoration improvements were made from 1994–2002. In 2001 the Park District stopped grooming a 12.4 ac section of beach immediately north of the site and dunes and habitat slowly evolved, and in 2002 the eastern edge of the site was expanded by 4 ac as part of an Army Corps of Engineers shoreline stabilization project. In 2015 a master plan for Montrose Bird Sanctuary was completed and 1.5 ac of parkland was added to the western edge of the site for a current total of 28 ac. Ecological restoration, trail, and accessibility

improvements were also made as part of the plan, including asphalt paving of the 0.35 mi main loop trail.

2. **Beauty-** The site has a high degree of vegetative land cover diversity, including prairie-meadow, shrub, woodland, wetland, open water (Lake Michigan), and dune areas. The core area of the site is flat topographically and largely enclosed by perimeter woodland vegetation, but the dune area includes a gentle rise that affords commanding views along the shoreline and out to the lake. The lake itself is a highly prominent and dynamic source of movement for waves, vegetation, and human and wildlife interaction. The point and especially breakwater extend out into the lake which also enhances views of the lake and city in all directions. The Caldwell design and Magic Hedge add important visible cultural elements of the site's design and aesthetic expression and are interpreted with signage at points along the trail network.

3. **Integrity-** Ecological restoration efforts beginning in 1994 have steadily increased the habitat value of the site for migratory birds, and ecological integrity is moderate to good; there is a fair level of vegetation species diversity though still a substantial weedy component. Non-native conifer and fruit trees are planted in a few spots to provide bird shelter and food. Understory in heavy shrub areas is bare in many places. Trail woodchip and dirt surfaces are generally in good condition with low erosion; asphalt on the center loop trail is new as of 2021, and the breakwater was rebuilt in 2002. The pier on the other hand is original from the 1930s and sections of concrete are damaged and could pose difficulties for walking. Cultural landscape elements include the Magic Hedge and the basic vegetative structure of the original Caldwell Plan, both of which are highlighted by onsite interpretive signage. While ecological management, trail paving for accessibility, and roping along trails to reduce trampling have considerably changed Caldwell's original vision for the core site, in balancing the various needs and goals for the site it can be said to have moderate overall integrity.

4. **Tranquility-** Tranquility is relatively high for this urban site due to its lakeshore location and distance from the very high volume, 8-lane DuSable Lake Shore Drive (DLSD), particularly along the outermost (lakeward) sections of the trails. This distance also mitigates the visual impacts of high-rise residential development on the west edge of DLSD, while distant views of downtown skyscrapers are generally viewed favorably. Use levels along most trail sections are low to moderate and nature oriented, though noise from adjacent beach use, motorboat use, and aircraft overflights can be distracting during peak times.

**Setting**	28 ac, surrounded by Lake Michigan, with adjacent land uses including Montrose Harbor and Montrose Beach.
**Visual**	few- beach use, boats, distant city views (most would consider positive).
**Sound**	Trails are 0.65–0.85 mi from very high volume 8-lane arterial (91K AADT); potentially frequent aircraft overflights every 2 minutes at 4600' (avg Lmax 60db); portions close to high-use beach and park areas, park roadways, sailboat marina, and shoreline fishing breakwater.
**Other**	None.
**Social**	Onsite use is low–moderate, mostly nature-oriented (birding). Adjacent park use is far enough away that it is rarely a distraction.
**Environmental**	Minor; wooded areas rarely buggy due to lake breeze; side trails can be overgrown with brushing against vegetation.

5. **Accessibility-** Accessibility is high; the site is a 21 min drive and is a 2/3 mi walk through the park from the nearest bus line. Access is free and parking lot and road parking is $2.50/hr., with free spaces available within a 10 min walking distance. Restroom and water facilities are available seasonally at a nearby beach house, where there is also a beachside restaurant. There are four walking trail options with lengths ranging from 0.35–1.5 mi plus a 4.75 mi bike and a 1 or 4 mi paddle route. The surface of the main loop is asphalt and fully wheelchair accessible, the dune section is sand, and the breakwater (wheelchair accessible) and are pier concrete. Widths vary from 4' (sand), 8' asphalt, 12' and wider concrete, and wide open on sand beach. All grades are easy; barriers include loose sand, steps in one area, and deteriorated concrete on outer pier. Wind and waves can make pier hazardous, including winter freezing.

**Proximity**	Site is 21 min (6.6 mi) from Nature Center origin.
**Facilities**	Parking along park roads; restrooms, drinking water, swimming beach, and beachside restaurant are open seasonally at nearby Montrose Beach.
**Fees**	Free site access; parking lot and road parking is $2.50/hr., with free spaces available within a 10 min walking distance.
**Options**	Four foot trail options identified with several variations, with lengths ranging from 0.35–1.5 mi, as well as a 4.75 mi bike loop route and a 1 or 4 mi paddle route along the Lake Michigan shore. Surface of main loop is asphalt (fully wheelchair accessible), dune section is sand, breakwater (wheelchair accessible) and are pier concrete. Widths vary from 4' (sand), 8' asphalt, 12' and wider concrete, and wide open on sand beach. All grades are easy; barriers include loose sand, steps in one area, and deteriorated concrete on outer

	pier. Wind and waves can make pier hazardous, including winter freezing.

Part II. Trail Level Criteria

**Trail:** Bird Sanctuary Main Loop					**Type:** Foot			
**Trail info:** https://www.chicagoparkdistrict.com/parks-facilities/lincoln-park-montrose-point-bird-sanctuary								
**N visits:** 6					**Seasons:**	**W**	**Sp**	**Su**	**F**
**Score**	**Ease of Travel**	**Attractive Layout**	**Natural Features**	**Built Features**	**Explorable Nature**	**Interp.**	**Total**	**Pct**
**L/M/H 1/2/3**	**3**	**2**	**3**	**1**	**2**	**3**	**14**	**78**

Part II A. Design and Construction

6. **Ease of Travel-** High

**Trailhead Distance**	0.10 mi.
**Length**	0.35 mi.
**Surface**	Main loop is asphalt with dirt and woodchip spur trails.
**Width**	8 ft trail modal width.
**Slope**	11% max slope, 2.8% avg.
**Accessibility Barriers**	None, main loop is fully wheelchair accessible.

7. **Attractiveness of Layout-** Moderate

**Alignment**	Mostly flat (12' elev change), winding path through open prairie and enclosed woodland, with view corridor openings to lake.
**Route Type**	2-way loop.
**Views**	Enclosed, open, semi-open, focal, distant views from a few KOPs esp. leaf-off.
**Spaces**	There are small spur trails that provide some privacy, in both open and enclosed areas off main trail, and a few bigger spaces at trail intersections but tend to be busier with birder activity.
**Changes**	Topographically homogeneous but vegetatively and spatially the path goes through a sequence of enclosed, edge, and open areas of woodland, savanna, and prairie-meadow.

Part II B. Key Trailside Features and Opportunities

8. **Natural Features-** High

**Vegetation Cover**	Mainly prairie-savanna with woodland edge, small wetland.

**Trees**	A few mature trees but not huge.
**Water**	Lake Michigan shoreline, small artificial spring and surrounding wetland. Lake is visual access FG-MG-BG at a few KOPs around the loop, esp. leaf off. Spring/wetland is largely hidden by vegetation but you can see/hear it IFG if you look for the feature.
**Wildlife**	Birds! Key birding area for seasonal bird migrations; also many butterflies, bees.
**Other**	Planted conifers, fruit trees and shrubs for bird habitat; flowering prairie plants.

9. **Built and Borrowed Features-** Low

**Seating**	None.
**Gateways**	Attractive overhead entry sign with gate at main entrance to prevent bikes from coming in.
**Shelter**	None, though beach house is < 10 min walk from site.
**Other Features**	Small naturalistic fountain but is invisible unless you know it's there.

10. **Explorable Nature-** Moderate

**Uses and Restrictions**	CPD natural area policies restrict any harvesting, collecting, or going off trail in fenced area.
**Museumification**	Rope fencing w/ wood posts along entire length of main and spur trails.
**On-Trail Engagement**	Natural area is roped off from the main trail loop through point area somewhat limiting interaction and engagement, though narrower spur trails provide a more engaging, intimate experience.

11. **Interpretation and Stewardship-** High

**Signage**	Info, regulatory, nice ecological and cultural interpretive signs.
**Learning and Stewardship Opportunities**	Demonstration garden; there is also a Montrose Point volunteer stewardship group that meets regularly.

**Trail:** Bird Sanctuary and Montrose Beach Dunes Loop					**Type:** Foot			
**Trail info:** https://www.chicagoparkdistrict.com/parks-facilities/lincoln-park-montrose-beach-dunes-natural-area								
**N visits:** 6					**Seasons:**	**W**	**Sp** **Su**	**F**
**Score**	**Ease of Travel**	**Attractive Layout**	**Natural Features**	**Built Features**	**Explorable Nature**	**Interp.**	**Total**	**Pct**
**L/M/H 1/2/3**	**3**	**3**	**3**	**1**	**2**	**3**	**15**	**83**

Part II A. Design and Construction

6. **Ease of Travel-** High

**Trailhead Distance**	0.10 mi.
**Length**	0.75 mi.
**Surface**	Major portion of trail is sand (dune loop) with asphalt and dirt adjoining trails.
**Width**	4–8 ft, 8 ft trail modal width plus open sand beach at lake.
**Slope**	12% max slope, 2.8% avg
**Accessibility Barriers**	Sand, steps.

7. **Attractiveness of Layout-** High

**Alignment**	25' elev. chg. but mostly flat, main loop path is winding with open prairie and enclosed woodland views; dunes path is mostly straight, and gradual elevation on dune provides elevated view of natural area, lakeshore and distant lake views.
**Route Type**	2-way loop.
**Views**	Enclosed, open, semi-open, focal, distant, panoramic, urban skyline.
**Spaces**	On dune rise there is a nice private sitspot plus open lakeshore spots for group activities.
**Changes**	There are a few gentle elevation changes coming down to the beach and up to the top of a dune; vegetatively and spatially the path goes through a sequence of enclosed, edge, and open areas of woodland, savanna, prairie-meadow, open grassland, tree-covered dune, open sand beach, wetland, and prairie-woodland edge.

Part II B. Key Trailside Features and Opportunities

8. **Natural Features-** High

**Vegetation Cover**	Dune grass through most of dune area with some aspen overstory plus prairie-savanna with woodland edge on Point portion of trail.
**Trees**	A few mature trees on Point, dunes are mostly open with a grove of cottonwood trees—wind through the aspen leaves is very pleasant.
**Water**	Lake Michigan shoreline FG eye level visual access along 0.2 mi dune stretch of trail. Swimming beach adjacent to natural area, wave watching, boats.
**Wildlife**	Birds! key birding area for seasonal bird migrations; also many butterflies, bees.
**Other**	Attractive driftwood along shore, lake breeze is a major feature of dune and lakeshore, waving grasses.

9. **Built and Borrowed Features-** Low

**Seating**	Driftwood log on dune is only seating.
**Gateways**	Attractive overhead entry sign with gate at main entrance to prevent bikes from coming in.
**Shelter**	None, though beach house is < 10 min walk from site.
**Other Features**	None.

10. **Explorable Nature-** Moderate

**Uses and Restrictions**	CPD natural area policies restrict any harvesting, collecting, going off trail in fenced area.
**Museumification**	Rope fencing w/ wood posts.
**On-Trail Engagement**	Natural areas are roped off from rather wide trail through point and dune area somewhat limiting interaction and engagement, though some narrower spur trail provide a more intimate experience and interactions with water and sand along the immediate shore outside of natural area are possible at adjacent swimming beach.

11. **Interpretation and Stewardship-** High

**Signage**	Info, regulatory, nice ecological and cultural interpretive signs.
**Learning and Stewardship Opportunities**	Demonstration garden at Point gateway entrance; there is also a Montrose Point/Dunes volunteer stewardship group that meets regularly.

**Trail:** Point-Dunes-Lake-Prairie Loop					**Type:** Foot			
**Trail info:** https://www.chicagoparkdistrict.com/parks-facilities/lincoln-park-montrose-beach-dunes-natural-area; https://www.chicagoparkdistrict.com/parks-facilities/lincoln-park-montrose-point-bird-sanctuary								
**N visits:** 6					**Seasons:**	**W** **Sp**	**Su**	**F**
**Score**	**Ease of Travel**	**Attractive Layout**	**Natural Features**	**Built Features**	**Explorable Nature**	**Interp.**	**Total**	**Pct**
**L/M/H 1/2/3**	**3**	**3**	**3**	**1**	**2**	**3**	**15**	**83**

Part II A. Design and Construction

6. **Ease of Travel-** High

**Trailhead Distance**	0.10 mi.
**Length**	0.85 mi.
**Surface**	Asphalt, concrete, dirt, sand, woodchips (segments ~ 20% each).
**Width**	2–12 ft plus open beach; modal is 8 ft.

**Slope**	12% max slope, 2.4% avg.
**Accessibility Barriers**	Sand, steps.

7. **Attractiveness of Layout-** High

**Alignment**	22' elev. chg.; loop combines winding trail through open enclosed prairie and woodland on the main Sanctuary loop with straight to gently curving trail and open views along lake, dune and prairie edge, and spectacular views of lake and distant downtown skyline.
**Route Type**	2-way loop.
**Views**	Enclosed, open, semi-open, focal, distant, panoramic, urban skyline.
**Spaces**	On dune rise there is a nice private sit spot plus open lakeshore spots for group activities.
**Changes**	There are a few gentle elevation changes coming down to the beach and up to the top of a dune; vegetatively and spatially the path goes through a sequence of enclosed, edge, and open areas of woodland, savanna, prairie-meadow, open grassland, tree-covered dune, open sand beach, wetland, prairie, and concrete lake edge.

Part II B. Key Trailside Features and Opportunities

8. **Natural Features-** High

**Vegetation Cover**	Woodland-savanna 30%, prairie 30%, dune 30%, lakeshore, wetland.
**Trees**	A few mature trees on Point, dunes are mostly open with a grove of cottonwood trees—wind through the aspen leaves is very pleasant.
**Water**	Lake Michigan shoreline FG eye level visual access along .2 mi dune stretch of trail; visual access FG superior along length of prairie/concrete revetment. Swimming beach adjacent to natural area, wave watching, boats.
**Wildlife**	Birds! key birding area for seasonal bird migrations; also many butterflies, bees esp. in prairie sections, seagulls along revetment and in air.
**Other**	Attractive driftwood along shore, lake breeze is a major feature of dune and lakeshore, waving grasses and forbs in dune and prairie.

9. **Built and Borrowed Features-** Low

**Seating**	Driftwood log on dune is only seating.
**Gateways**	Attractive overhead entry sign with gate at main entrance to prevent bikes from coming in.
**Shelter**	None, though beach house is < 10 min walk from site.
**Other Features**	None.

10. Explorable Nature- Moderate

**Uses and Restrictions**	CPD natural area policies restrict any harvesting, collecting, going off trail in fenced area.
**Museumification**	Rope fencing w/ wood posts.
**On-Trail Engagement**	Natural areas are roped off from rather wide trail through point and dune area somewhat limiting interaction and engagement, though some narrower spur trail provide a more intimate experience and interactions with water and sand along the immediate shore outside of natural area are possible at adjacent swimming beach.

11. Interpretation and Stewardship- High

**Signage**	Info, regulatory, nice ecological and cultural interpretive signs.
**Learning and Stewardship Opportunities**	Demonstration garden at Point gateway entrance; there is also a Montrose Point/Dunes volunteer stewardship group that meets regularly.

**Trail:** Breakwater-Pier					**Type: Foot**			
**Trail info:** https://www.chicagoparkdistrict.com/parks-facilities/lincoln-park-montrose-point-bird-sanctuary								
**N visits:** 2					**Seasons:**	**W**	**Sp**	**Su** / **F**
**Score**	**Ease of Travel**	**Attractive Layout**	**Natural Features**	**Built Features**	**Explorable Nature**	**Interp.**	**Total**	**Pct**
**L/M/H 1/2/3**	**2**	**1**	**2**	**1**	**1**	**1**	**8**	**44**

Part II A. Design and Construction

6. Ease of Travel- Moderate

**Trailhead Distance**	0.10 mi.
**Length**	1.5 mi.
**Surface**	Concrete.
**Width**	12 ft.
**Slope**	5% max slope, 1.4% avg.
**Accessibility Barriers**	Concrete on pier portion of route has some deteriorated sections; there is a railing in the center as you get further out toward the end. Wind and waves can make pier hazardous, including winter freezing. Most of the length of the route is wheelchair accessible.

7. **Attractiveness of Layout-** Low

**Alignment**	10' elev. chg., straight to gently curving .5 mi breakwater revetment and winding .25 mi fishhook pier provide eye-level views of open prairie and superior views of dunes and lakeshore along with spectacular views of open lake and distant downtown skyline.
**Route Type**	2-way linear (there and back).
**Views**	Open, distant, panoramic, urban skyline.
**Spaces**	No private spaces; the entire length of route is open for potential group use but it's in a very public setting.
**Changes**	Mostly a concrete lake edge.

Part II B. Key Trailside Features and Opportunities

8. **Natural Features-** Moderate

**Vegetation Cover**	Concrete
**Trees**	No.
**Water**	Lake Michigan shoreline, wetland area between dune and breakwater visual access FG-IFG superior and level along entire route. Swimming beach nearby.
**Wildlife**	Birds! key birding area for seasonal bird migrations.
**Other**	No.

9. **Built and Borrowed Features-** Low

**Seating**	None.
**Gateways**	None.
**Shelter**	None.
**Other Features**	None.

10. **Explorable Nature-** Low

**Uses and Restrictions**	Standard park use restrictions, no swimming off pier.
**Museumification**	None.
**On-Trail Engagement**	Experience is mainly one of visual/remote engagement with the water which is well below the pier and breakwater; swimming beach is nearby.

11. **Interpretation and Stewardship-** Low

**Signage**	None.
**Learning and Stewardship Opportunities**	None.

<table>
<tr><td colspan="5">Trail: Montrose Waterfront Paddle</td><td colspan="5">Type: Paddle</td></tr>
<tr><td colspan="10">Trail info: https://www.chicagoparkdistrict.com/parks-facilities/lincoln-park-montrose-beach-dunes-natural-area</td></tr>
<tr><td colspan="5">N visits: 1</td><td>Seasons:</td><td>W</td><td>Sp</td><td>Su</td><td>F</td></tr>
<tr><td>Score</td><td>Ease of Travel</td><td>Attractive Layout</td><td>Natural Features</td><td>Built Features</td><td>Explorable Nature</td><td colspan="2">Interp.</td><td>Total</td><td>Pct</td></tr>
<tr><td>L/M/H
1/2/3</td><td>2</td><td>2</td><td>2</td><td>1</td><td>2</td><td colspan="2">1</td><td>10</td><td>56</td></tr>
</table>

Part II A. Design and Construction

6. **Ease of Travel-** Moderate

**Trailhead Distance**	0.10 mi.
**Length**	1 mi loop from beach (outside seasonal swimming hours only) or 4 mi there and back from harbor boat landing.
**Surface**	Flatwater; beach loop has shallow sand bottom (inside buoys).
**Width**	4 – 8 ft, 8 ft trail modal width plus open sand beach at lake.
**Slope**	12% max slope, 2.8% avg.
**Accessibility Barriers**	Loop route requires a portage across a wide sand beach or put in and take out at the formal boat landing. Paddling is subject to wind and wave conditions that can make navigation difficult.

7. **Attractiveness of Layout-** Moderate

**Alignment**	Flatwater paddle follows shore in a loop west of the fishhook pier or a longer circuit up and around it, offering panoramic views of the lake and shoreline. The longer trip included harbor and skyline views of the city.
**Route Type**	2-way loop.
**Views**	Panorama and distant views of lake and skyline.
**Spaces**	Wide open; by yourself on the water but group paddles are possible.
**Changes**	Flat water, changes in view of park shoreline from hard edge to natural area to swimming beach.

Part II B. Key Trailside Features and Opportunities

8. **Natural Features-** Moderate

**Vegetation Cover**	Shoreline cover includes concrete revetment with park lawn, prairie, dune, and beach areas visible from FG across open water.
**Trees**	None prominent.
**Water**	Lake Michigan shoreline 2 mi length-I found this a less pleasant way to experience the Montrose area site; being out beyond the buoys puts you too far away from the low land to appreciate its qualities.

	On the other hand, being on the wide-open water has its own beauty, with sounds of waves, and more distant views of the city are wonderful from the water. The waterfront paddle has good water quality but may have compatibility issues with other boaters in the harbor area and offshore if the longer route is taken and with swimmers on the shorter route (early morning hours advised).
**Wildlife**	Key birding area for seasonal migrations, shorebirds; piping plover nesting area.
**Other**	None.

9. **Built and Borrowed Features-** Low

**Seating**	No.
**Gateways**	No gateway at beach; harbor boat landing and outlet to lake makes a spectacular gateway.
**Shelter**	None.
**Other Features**	Harbor boat landing; fishook pier is dominant feature.

10. **Explorable Nature-** Moderate

**Uses and Restrictions**	Boating policies restrict boating to outside buoys during swim season and hours.
**Museumification**	No.
**On-Trail Engagement**	Interaction with water and waves mostly in boat; buoys are some distance from shore, limiting shoreline interaction.

11. **Interpretation and Stewardship-** Low

**Signage**	None.
**Learning and Stewardship Opportunities**	None.

**Trail:** Montrose Area Bike Loop					**Type:** Bike			
**Trail info:** https://www.chicagoparkdistrict.com/parks-facilities/lincoln-park-montrose-beach-dunes-natural-area								
**N visits:** 2					**Seasons:**	**W** / **Sp**	**Su**	**F**
**Score**	**Ease of Travel**	**Attractive Layout**	**Natural Features**	**Built Features**	**Explorable Nature**	**Interp.**	**Total**	**Pct**
**L/M/H 1/2/3**	**3**	**3**	**3**	**3**	**2**	**1**	**15**	**83**

Part II A. Design and Construction

6. **Ease of Travel-** High

**Trailhead Distance**	0.0 mi.
**Length**	4.75 mi.
**Surface**	Asphalt (50%), concrete (30%), crushed gravel (20%).
**Width**	12 ft.
**Slope**	3% max slope, 1.3% avg.
**Accessibility Barriers**	A portion of the route uses the busy Lakefront Trail but pedestrians and bikes have separate directionally marked lanes. Most of the route can be done by wheelchair.

7. **Attractiveness of Layout-** High

**Alignment**	10' elev change. Loop trail through Lincoln Park follows a straight to gently curving alignments on level terrain offering in-park views of harbor, playfields, golf course, passive use and natural area facilities and outer views to lake, DuSable Lake Shore Dr and adjacent highrise residential development, and distant views of the downtown skyline.
**Route Type**	2-way loop.
**Views**	Feature views of park facilities, panorama and distant views of lake and skyline.
**Spaces**	Private and group spaces mostly within natural areas that this trail connects to-- see above listings.
**Changes**	Many changes in views of park along the route from passive open space to active play areas to busy trail use to wide open lake views to harbor.

Part II B. Key Trailside Features and Opportunities

8. **Natural Features-** High

**Vegetation Cover**	Land cover includes highway, parkland/trail corridor, golf course, ballfields and playing courts, concrete pier, beach/dune, and boat harbor areas.
**Trees**	A few large park trees.
**Water**	Lake Michigan shoreline 1.6 mi length visual FG superior access for 1/3 of route.
**Wildlife**	Key birding area for seasonal migrations, shorebirds.
**Other**	None.

9. **Built and Borrowed Features-** High

**Seating**	Standard park benches.

**Gateways**	Lacking a defined gateway due to multiple entry points along route.
**Shelter**	Clock Tower building has covered outdoor shelter space, indoor space (not always open) and restrooms.
**Other Features**	Waveland café at south end of golf course has food and drink with indoor and outdoor seating; also Kwagulth Totem Pole.

10. Explorable Nature- Moderate

**Uses and Restrictions**	Standard city park trail use restrictions; CPD natural area policies restrict any harvesting, collecting, going off trail in fenced area.
**Museumification**	Stretch between shoreline and golf course is walled off on both sides; see also sites above.
**On-Trail Engagement**	The loop route connects each of the Lincoln Park North Natural Areas, providing a delightful way to visit these sites and additionally offers exceptional views of the lakeshore, golf course, Montrose Harbor, and fishhook pier on an easy, level route that makes for an enjoyable, immersive park visit.

11. Interpretation and Stewardship- Low

**Signage**	Park informational and regulatory signage along route.
**Learning and Stewardship Opportunities**	No route specific opportunities known.

Part III. Statistical Summary, Map and Photos

Site Name and Scores	Beauty	Integrity	Tranquility	Accessibility	Total	Avg.
**Montrose Point**	**3**	**2**	**3**	**3**	**11**	**92%**

Nbr	Trail Name	Type	Length (km)	Ease	Layout	Natural	Built	Explore	Interp.	Total	Pct.
1	Bird Sanctuary Main Loop	Foot	0.6	3	2	3	1	2	3	14	78%
2	Bird Sanctuary & Montrose Beach Dunes Loop	Foot	1.2	3	3	3	1	2	3	15	83%
3	Point-Dunes-Lake-Prairie Loop	Foot	1.4	3	3	3	1	2	3	15	83%
4	Breakwater-Pier	Foot	2.4	2	1	2	1	1	1	8	44%
5	Montrose Waterfront Paddle	Paddle	1.6	2	2	2	1	2	1	10	56%
6	Montrose Area Bike Loop	Bike	7.6	3	3	3	3	2	1	15	83%

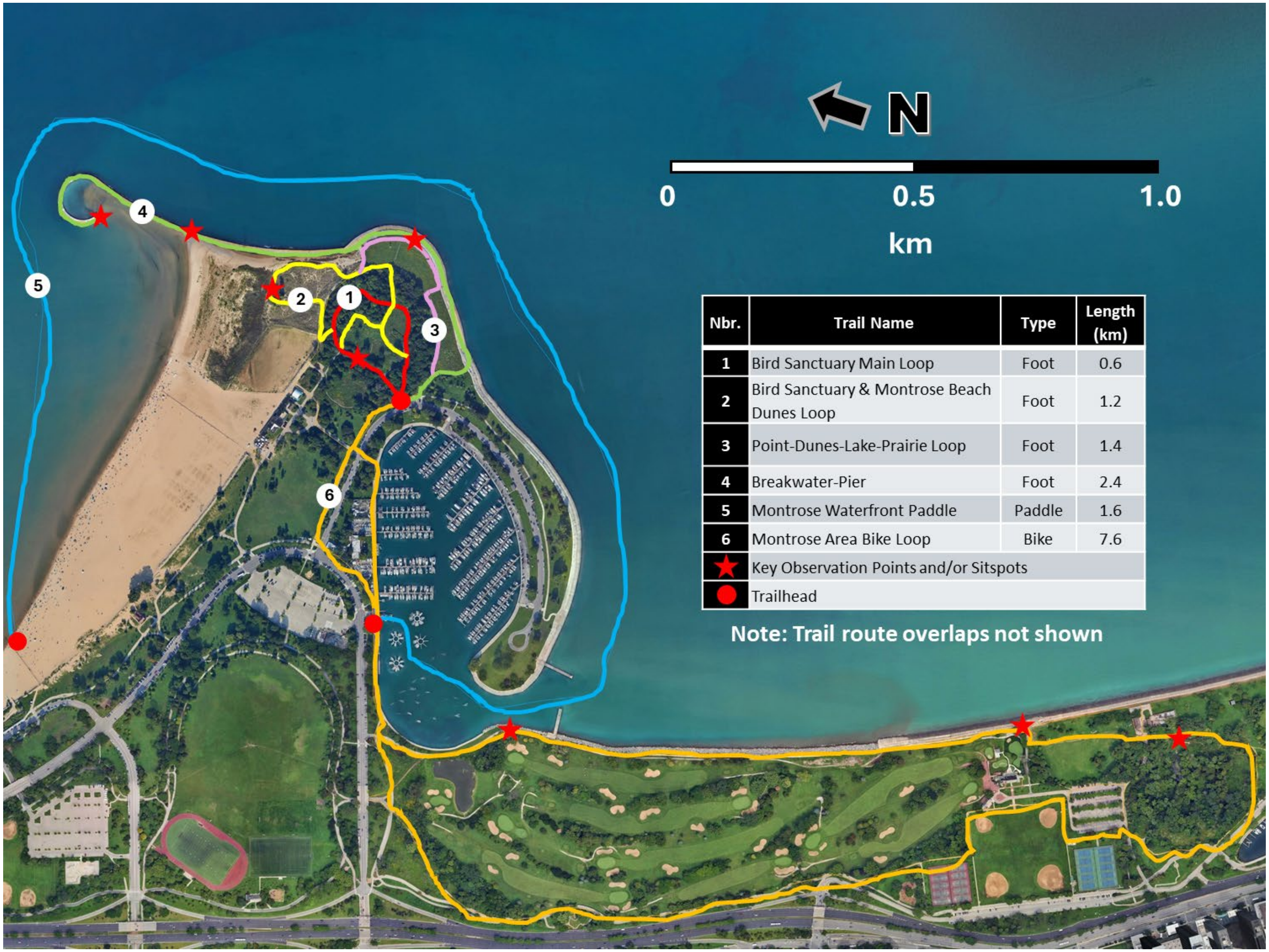

Fig. 1. Montrose Point site map showing trail network. Base map Google Earth, image 07/17/2023.

1. Bird Sanctuary Main Loop

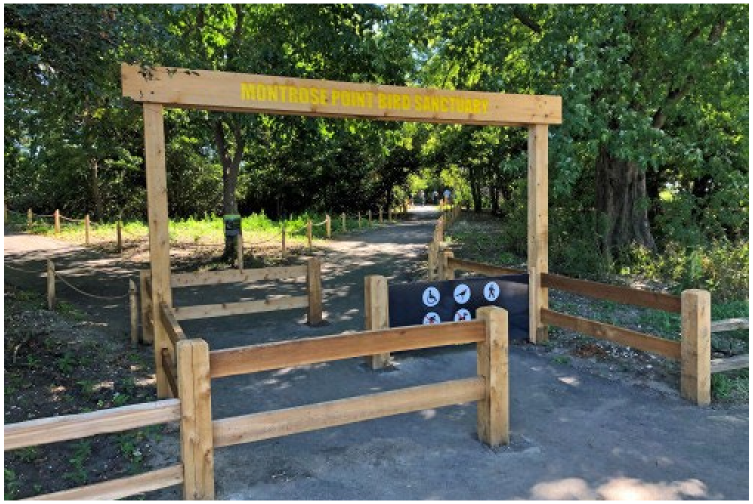

1a. Gateway entrance.

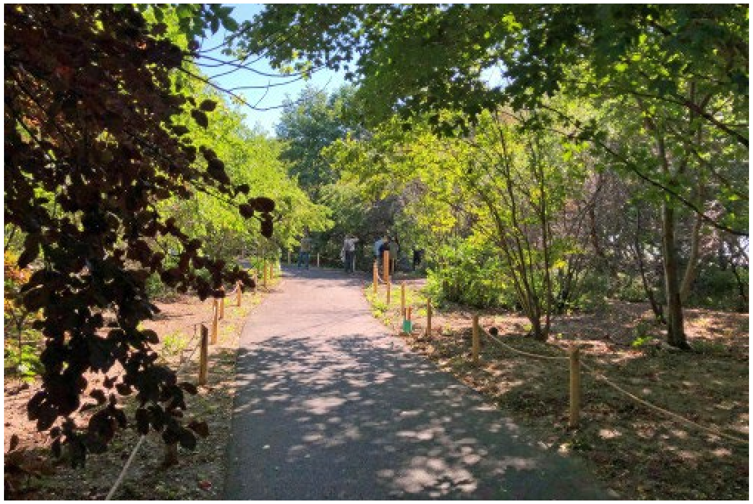

1b. “Magic Hedge” key observation point.

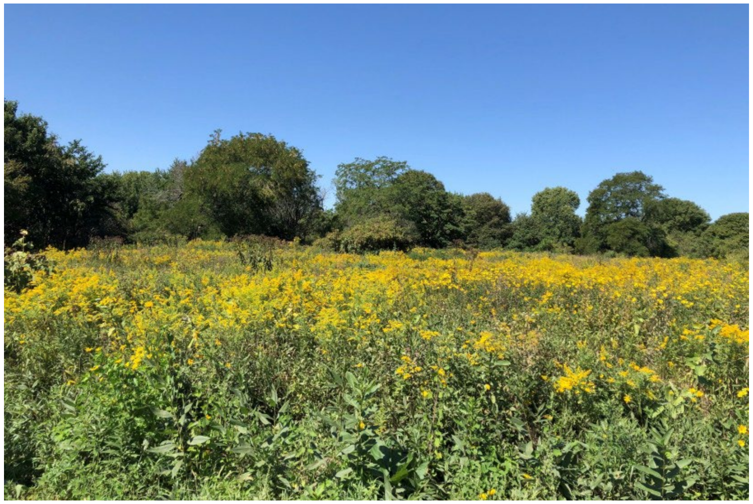

1c. Central prairie opening.

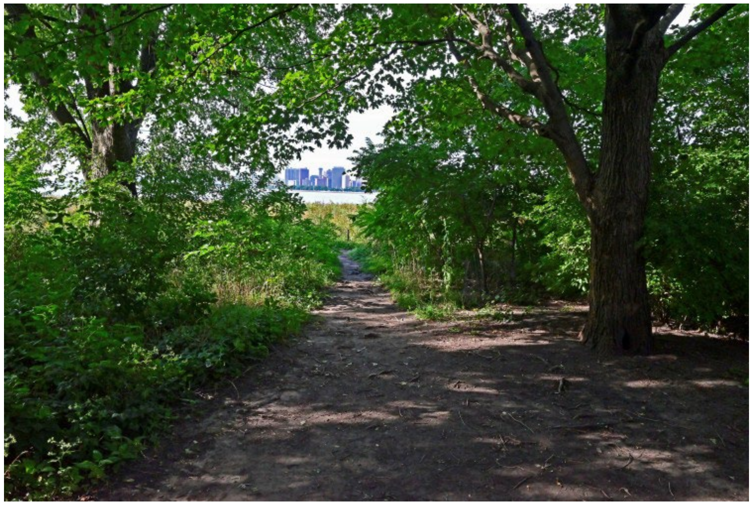

1d. Spur trail view corridor key observation point.

2. Bird Sanctuary & Montrose Beach Dunes Loop

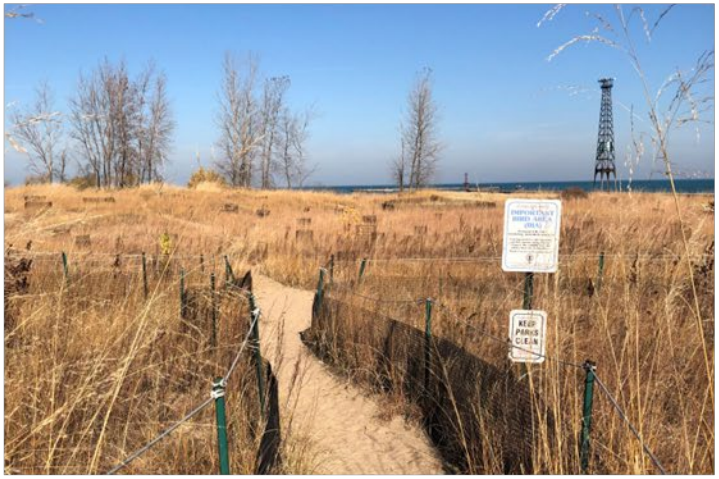

2a. Central path looking toward lake.

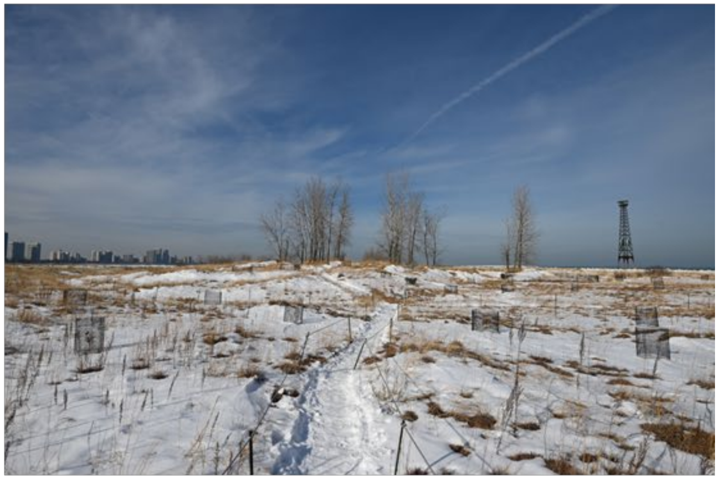

2a. Central path looking toward lake, winter.

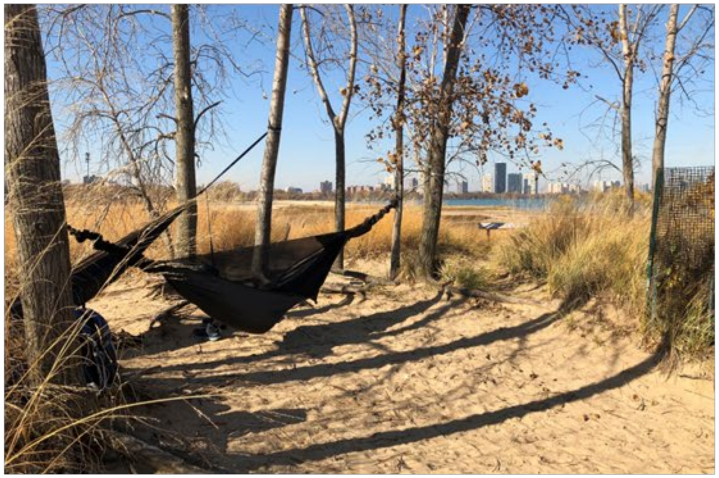

2c. Dune sitspot key observation point with interpretive sign, fall.

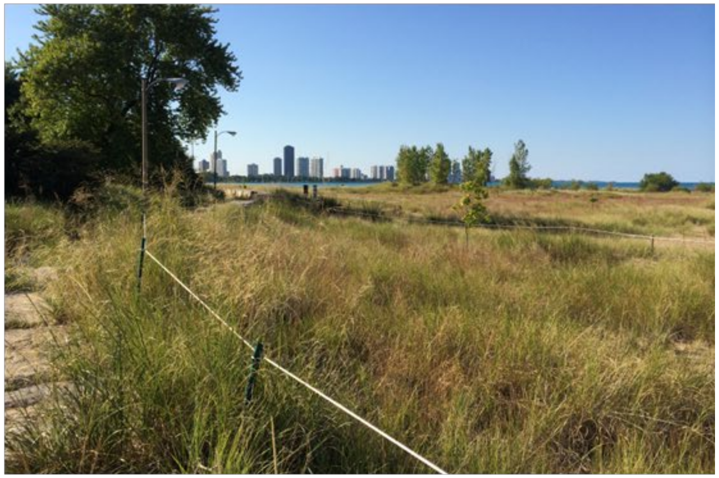

2d. Edge between Bird Sanctuary and Dune areas.

3. Point - Dunes - Lake - Prairie Loop

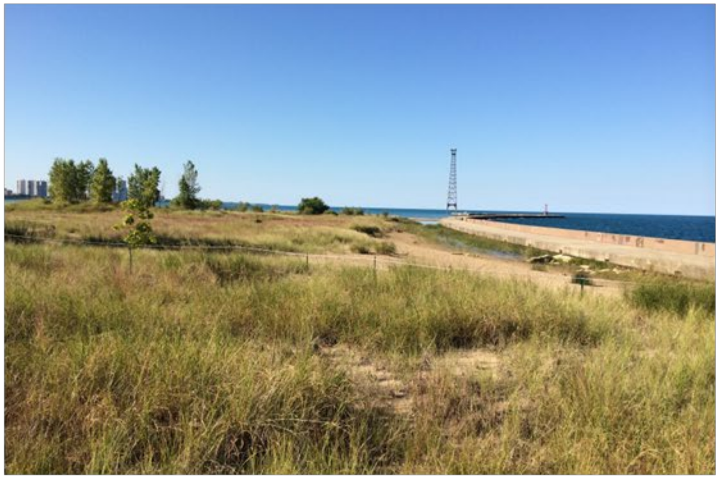

3a. Backwater wetland between dunes and pier.

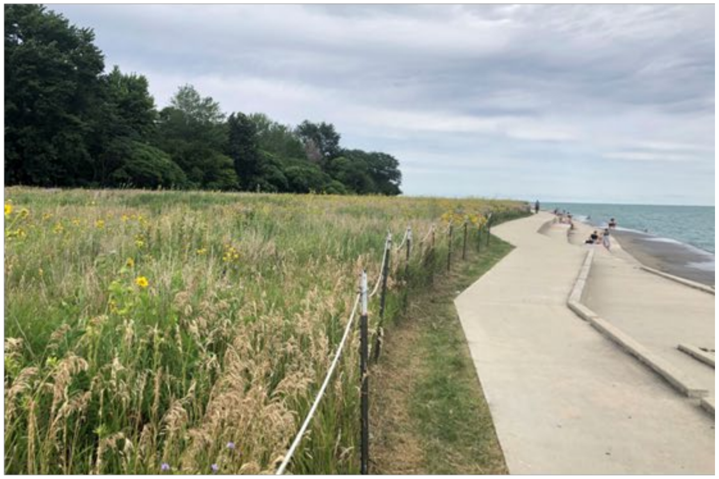

Lake-prairie edge.

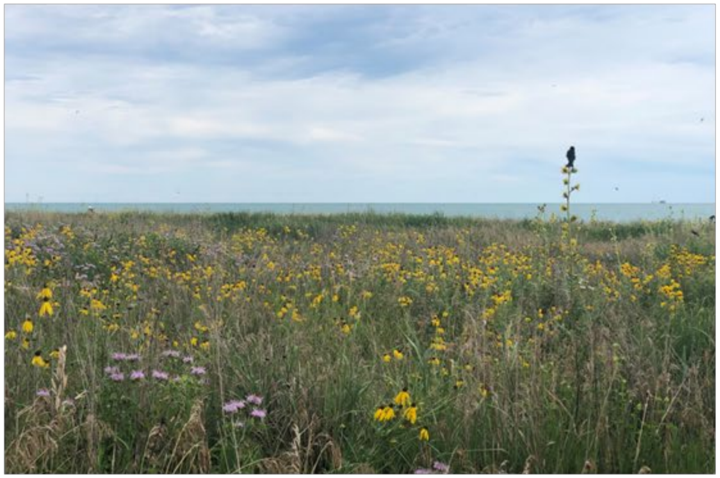

3c. Prairie interior path with view to lake.

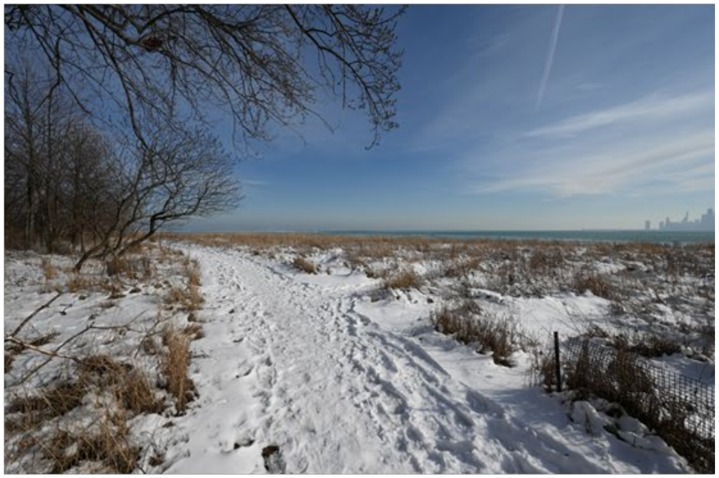

Bird Sanctuary-prairie edge, winter.

4. Breakwater - Pier

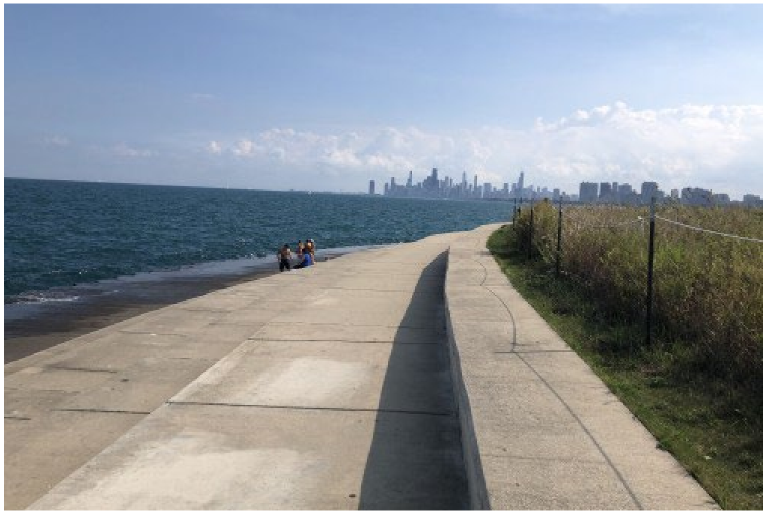
4a. Breakwater revetment key observation point.

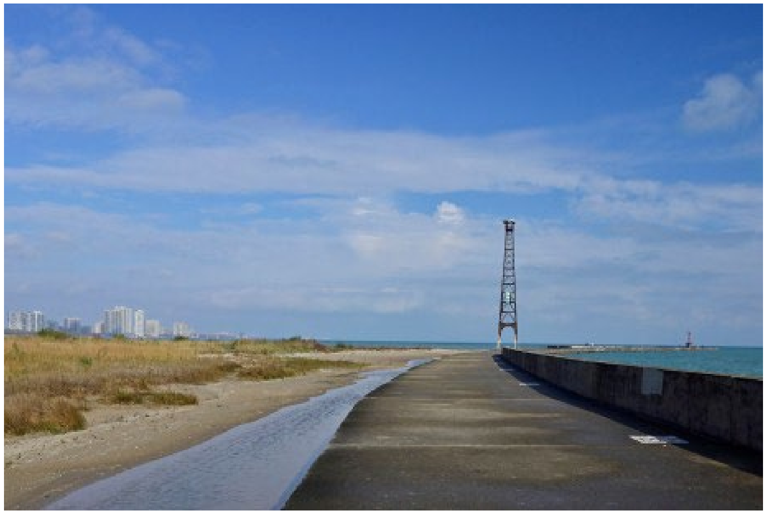
4b. Tower gateway to pier.

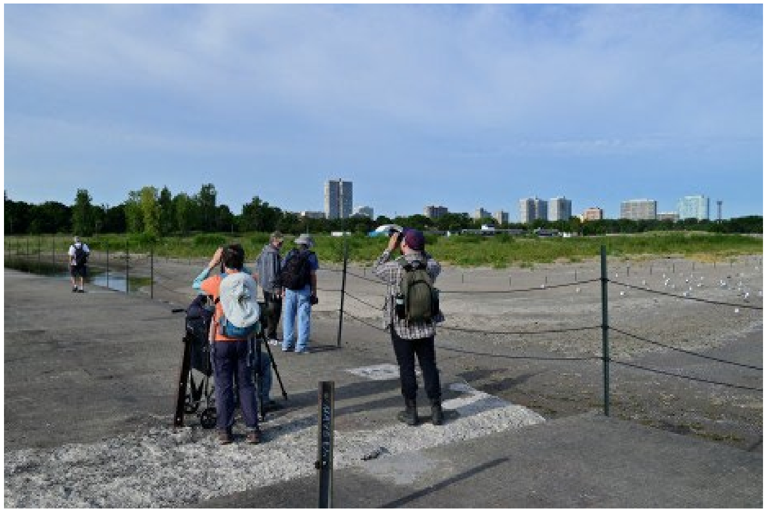
4c. View to dunes key birding observation point.

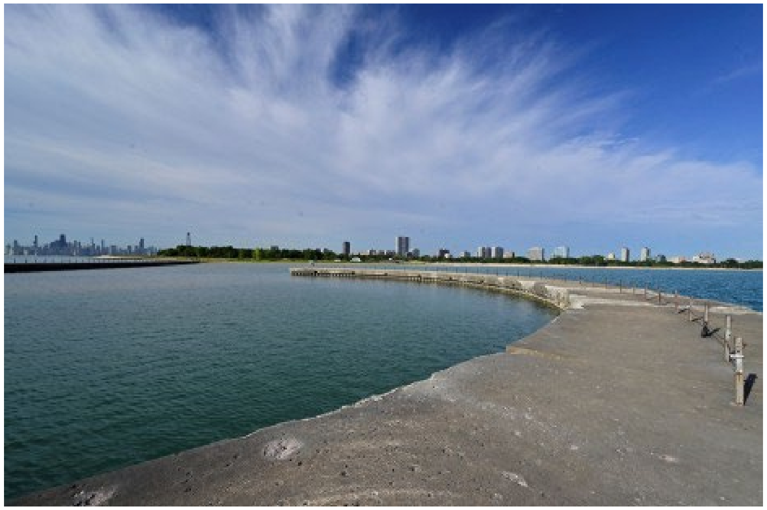
4d. Fish-hook pier end key observation point.

5. Montrose Waterfront Paddle

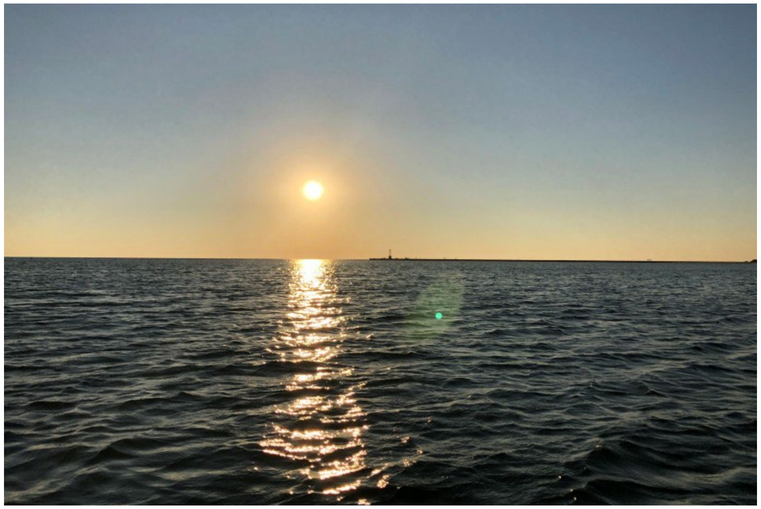

5a. Open water panoramic view.

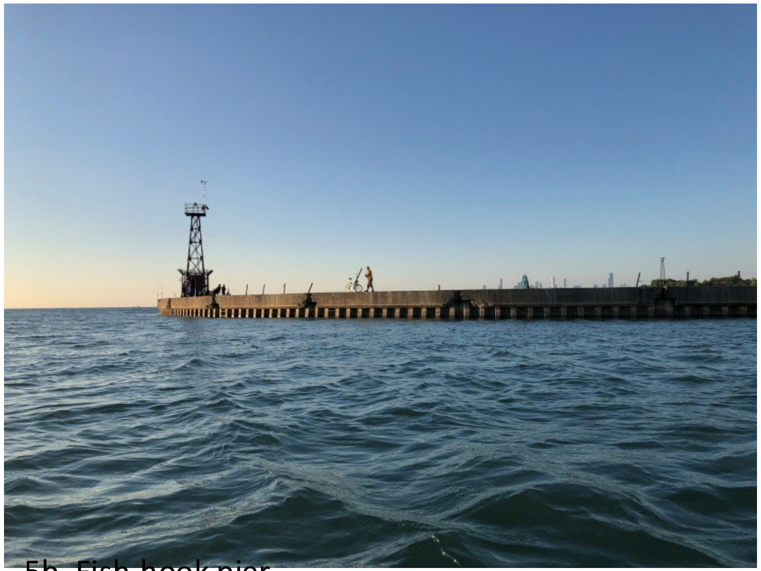

5b. Fish-hook pier.

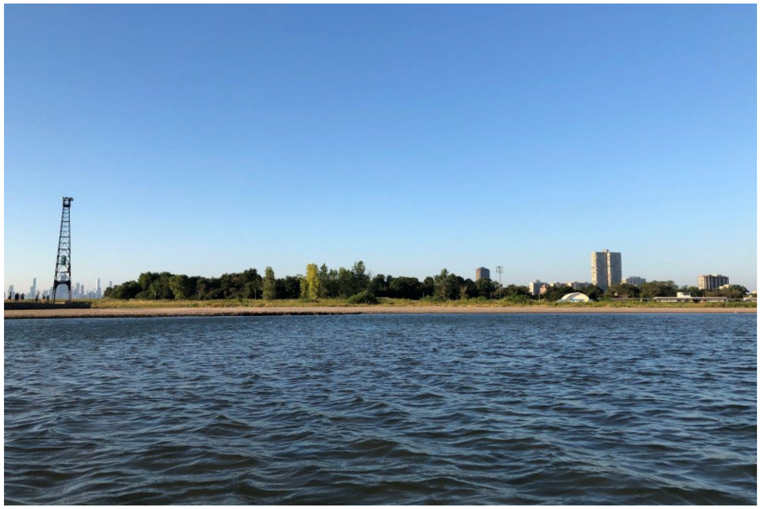

5c. Montrose Point shoreline.

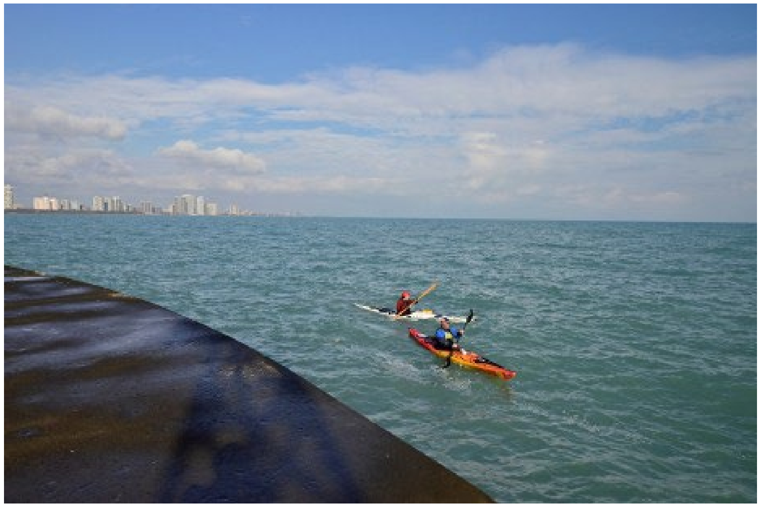

5d. Montrose Point paddlers.

6. Montrose Area Bike Loop

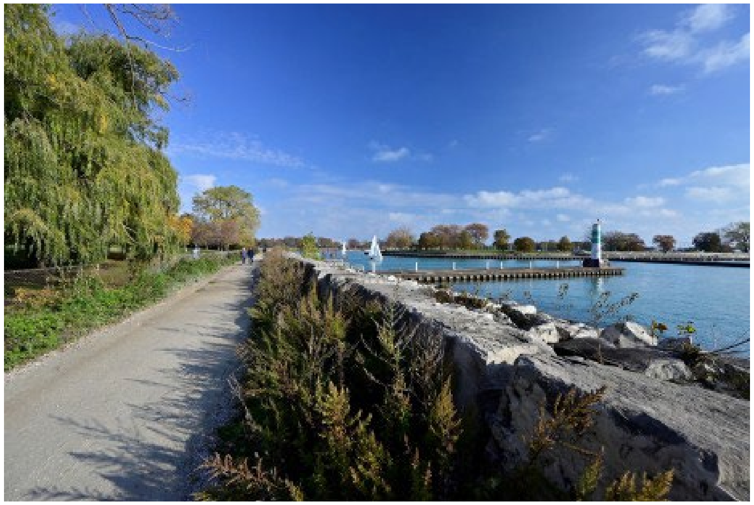

6a. Montrose Harbor key observation point.

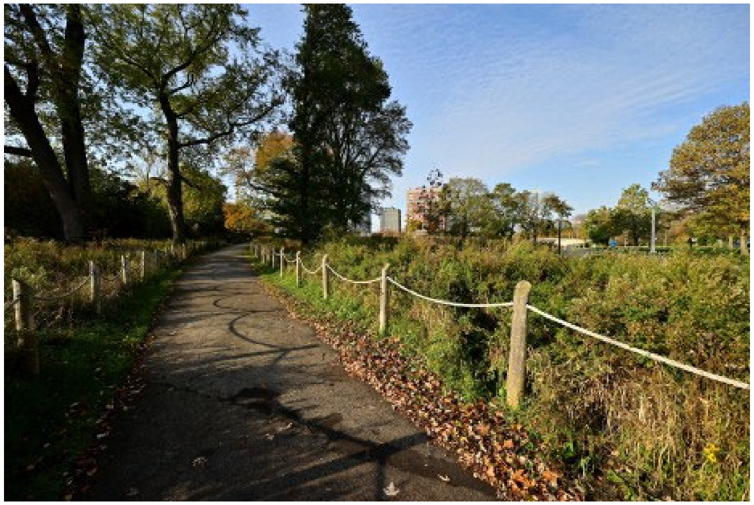

6b. Marovitz Savanna.

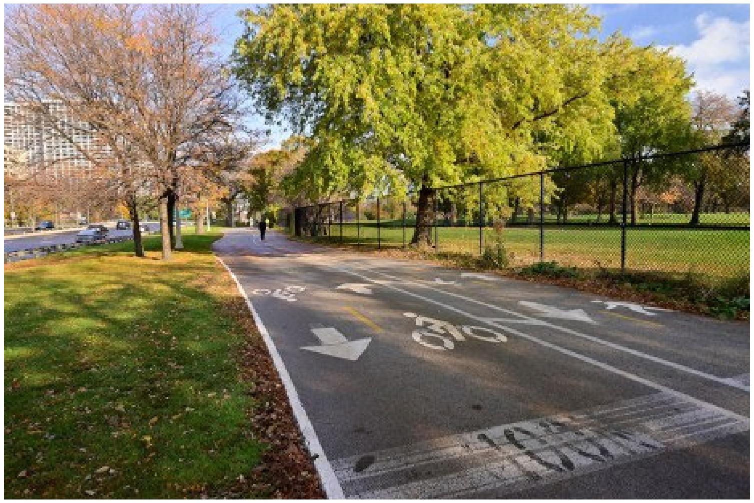

6c. Lakefront bike path section.

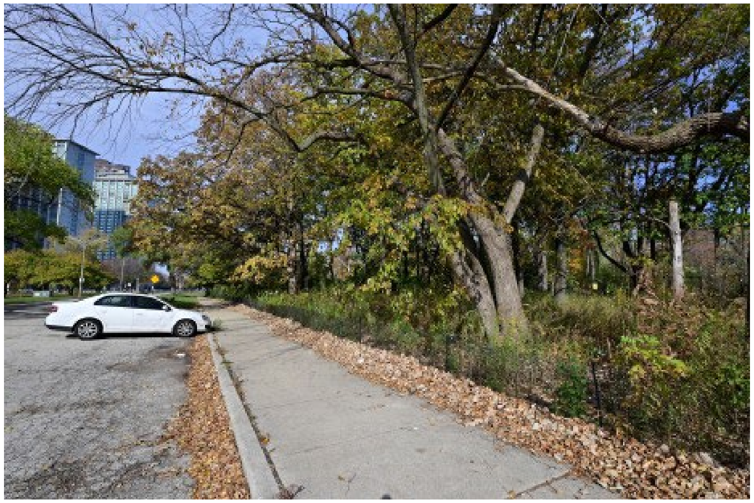

6d. Bill Jarvis Bird Sanctuary.
