# Supplementary material for: Forest Therapy Trails: Development and Application of an Assessment Protocol
Source: Int J Environ Res Public Health. 2025 Sep 16;22(9):1440. doi: 10.3390/ijerph22091440 (PMC12470198; doi:10.3390/ijerph22091440)
Supplement: Supplementary file 1 [file ijerph-22-01440-s001.zip › Supp Doc S6 Illustrated Criteria.pdf]

Illustrated Examples of Selected Trail Level Criteria and Sub-Criteria from the Two Study Areas

A Supplementary Document to

Forest Therapy Trails: Development and Application of an Assessment Protocol

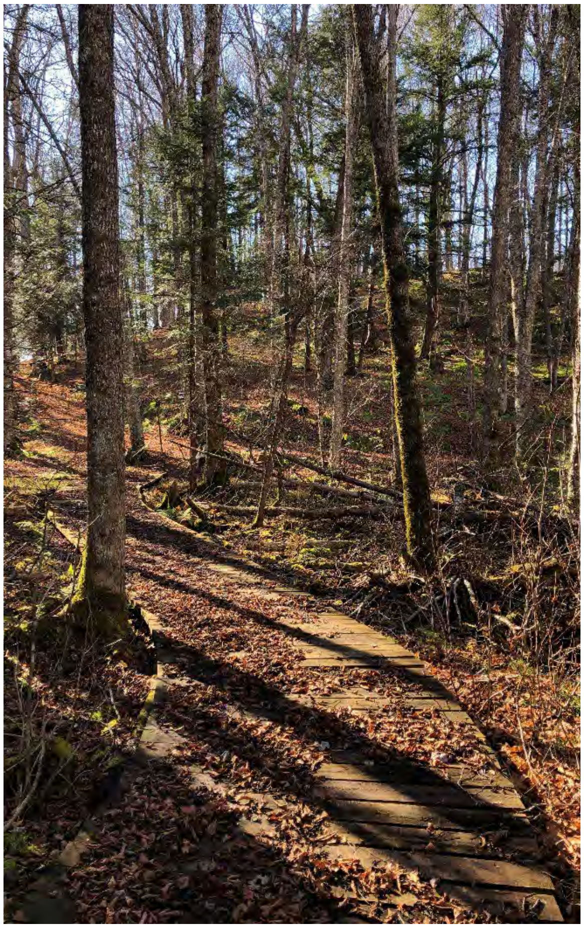

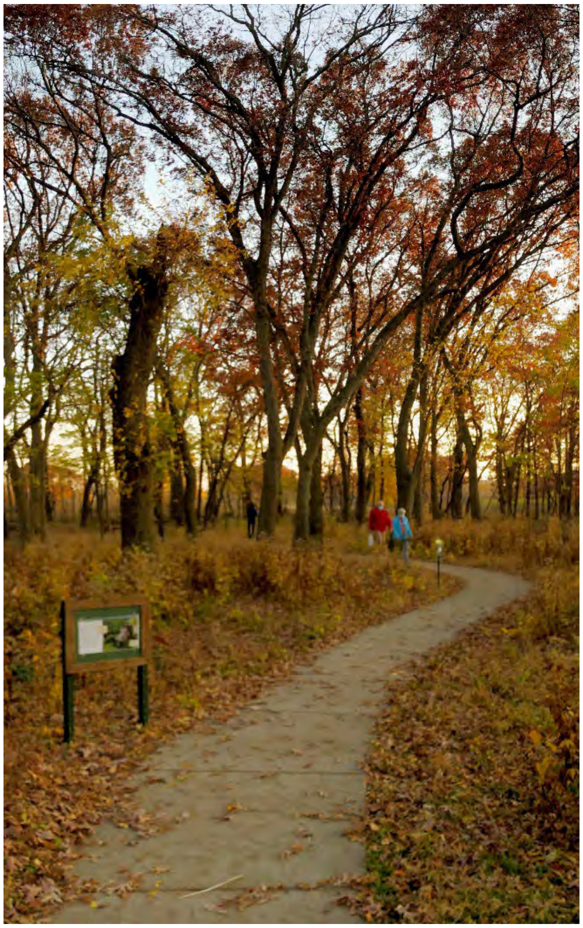

Left: Lauterman Lake Trail Loop, Lauterman National Recreation Trail (4.12); right: Woodland Loop, West Ridge Nature Park (11.21)

Paul H. Gobster, PhD, FASLA
Senior Research Landscape Architect Emeritus
USDA Forest Service, Northern Research Station
People and Their Environments Research Unit
Evanston, Illinois

Preface

This supplementary document provides illustrated examples of key features and opportunities relating to the trail level criteria of the assessment protocol. The examples are limited to trail level sub-criteria that can be readily represented photographically; sub-criteria such as trail length, slope, and route type are not included for this reason, nor are any site level sub-criteria due to the difficulty of portraying the more complex and non-visual aspects of integrity, tranquility, and accessibility. While the site level criterion of beauty has a strong visual orientation, I decided that the trail level sub-criteria relating to layout attractiveness and natural and built features sufficiently captured how the variety, vividness, and uniqueness of physical, biological and/or cultural-historic patterns and features relating to beauty are manifested at the trail level.

The document draws on the catalog of images I collected during the field surveys and in a few cases from my earlier photographs of the same trails. Locating appropriate images among the 1000s of photos I collected was in part done from my memories of the field and subsequent coding for the protocol, but also involved several combs through the catalog, which were organized in folders on my hard drive by study area, place, site, and trail. Further identification of features such as trail widths and surfaces was done with the aid of the spreadsheet I used to code inventory data for the protocol, which I could sort and search to locate examples.

While my emphasis here is on features and opportunities that enhance forest therapy experiences, when warranted I have included examples such as accessibility and museumification barriers that can detract from experiences for some people. I have also tried to illustrate the range of conditions and examples of sub-criteria such as trail surface types and widths; big trees, seating types, and other natural and built features; and opportunities for nature exploration, interpretation and stewardship. While these span a fairly wide range across my Northwoods and Chicago study areas, they are limited by the conditions and examples existing within the trail sites and regions and readers should gauge how they apply to their own situations.

The images are organized by numbered criteria and sub-criteria shown in the Table of Contents, with additional divisions described by the page titles. Individual photos (4 to each page) are labeled a–d and identified by trail name and number in parentheses corresponding to the trail number used in the main paper and other supplementary documents. While the photos and these title and caption descriptions are self-explanatory in many cases, I have added text at the start of each criterion section as needed to further explain what is being depicted and why, with references to related sub-criteria where appropriate. This text is based largely on my personal observations and reflections and is supplied without supporting citations to the research and planning literature; that is provided in the body of the main paper and the two related papers that precede it. Recommendations stated here should not be construed to represent any official USDA or U.S. Government determination or policy. Plant identification for some of the photo captions was done using the PictureThis phone app Version 5.21.0; any errors in labeling are my mistakes.

Finally, a note on the photography. Most of the images were captured either with a Nikon Z5 (14–30mm wide angle, 50mm normal/macro, and 24–200mm lenses) or iPhone 8 Plus camera. Image post processing was done with Adobe Photoshop Elements, including cropping and exposure corrections to improve image readability of the features portrayed in the small page image frames. This pdf document was saved in reduced size to keep the file size reasonable for publication, resulting in some loss of resolution; those desiring higher quality images or permission to reproduce them can contact me directly.

Paul Gobster

July 18, 2025 Chicago, Illinois

Table of Contents

Design and Construction

1. Ease of Travel......3
   1.1. Surface......4
   1.2. Width......9
   1.3. Accessibility Barriers......16

2. Attractiveness of Layout......19
   2.1. Alignment......20
   2.2. Views......23
   2.3. Spaces......28
   2.4. Changes......30

Key Features and Opportunities

3. Natural Features......36
   3.1. Trees......37
   3.2. Water......48
   3.3. Wildlife......57
   3.4. Flora, Rock, Moss, and Fungi......60

4. Built and Borrowed Features......68
   4.1. Seating......69
   4.2. Gateways......74
   4.3. Shelter......78
   4.4. Bridges and Boardwalks......81
   4.5. Miscellaneous Features......84

5. Explorable Nature......90
   5.1. Uses and Restrictions......91
   5.2. Museumification......93
   5.3. On-Trail Engagement......95

6. Interpretation and Stewardship......97
   6.1. Interpretive Signage......98
   6.2. Demonstration Gardens......102
   6.3. Programs and Information......103
   6.4. Volunteer Opportunities......104

1. Ease of Travel

1.1. Surface- Native ground surfaces of dirt and turf were common to foot trails in the Northwoods and Chicago forest preserve study areas. So long as they provide a firm, stable bed for walking, they are well-suited for forest therapy because of their natural look and fit to the surrounding landscape. Such trails, either unmodified (1.11a–b) or maintained by mowing and vegetation clearance (1.11c–d), work well in low-use situations where drainage, soils, and slopes are not concerns for erosion. Otherwise, some type of imported materials such as woodchips or crushed gravel (1.12a–b) may be warranted, and in urban and high-use situations, hard paving with asphalt or concrete (1.12c–d) may be needed. Similar considerations apply to bike and shared use trails (1.13). While surface type is included in this paper mainly as a functional sub-criterion, there are definite aesthetic considerations for enhancing forest therapy experiences. In particular, trail designers, planners, and providers can take advantage of indigenous (1.14) and introduced (1.15) soft and hard surfaces along trail segments and spurs that provide visitors with multisensory experiences, for barefoot walking or other visual and textural appeal.

1.2. Width- The first four pages in this section illustrate foot trails of increasingly wider widths under each of the four seasons experienced in the study areas. Narrow widths provide for intimate interaction with the surrounding landscape (see also On-Trail Engagement below) but their tracks can fade or disappear altogether in snow or as vegetation or leaf litter accumulates, especially in low use situations (1.21). Narrow trails can also breed discomfort or anxiety for some users fearful of contact with insects, mosquitos and ticks which are of particular concern in the two study areas. Wide trails (1.24) help alleviate such concerns, but with some tradeoff of distancing the visitor from landscape details and experiences. For most situations, medium width trails (1.22–1.23) of 3–8 ft (1–2.5 m) provide a good balance for comfort and experience. Similar considerations apply for bike (1.25) and water trails on rivers (1.26) and lakes (1.27).

1.3. Accessibility Barriers- This section illustrates a range of physical barriers that can limit the accessibility of trails for some forest therapy visitors, including young children and older adults, those with balance issues, those lacking physical endurance or navigation skills, and anyone looking for an easeful route with a minimum of disruptions. These barriers include muddy, low lying stretches and steep grades with exposed roots and rocks (1.31a–b), unmaintained areas with brushy incursions or fallen trees (1.31c–d). Trail slopes with unstable soils are prone to erosion in high-use areas (1.32a), while narrow trails in landscapes with fast growing ground vegetation such as prairies can become quickly overgrown in the summer (1.32b). I visited one popular Chicago trail with a plastic boardwalk shortly after it had rained and found the trail surface extremely slippery (1.32c), while bike and paddle trails have their own unique barriers including busy street crossings (1.32d), poor landings, fallen trees, rapids, and waves (1.33a–d).

1.11. Surface– Native Surfaces

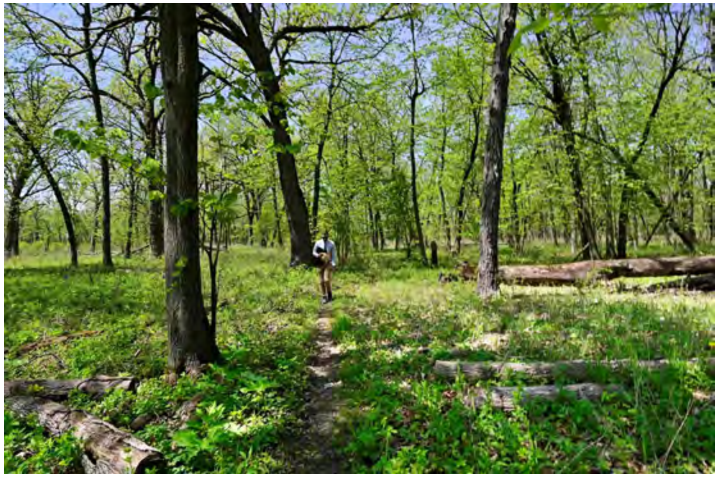

a. Dirt, West Inner Loop, Somme Woods (16.31)

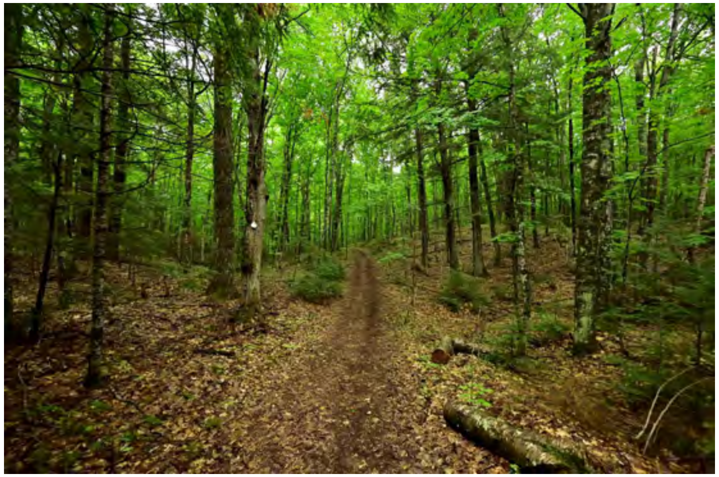

b. Dirt Two-Track, Franklin Nature Trail–Two Dutchmen Lake Loop, Hidden Lakes Trail (6.12)

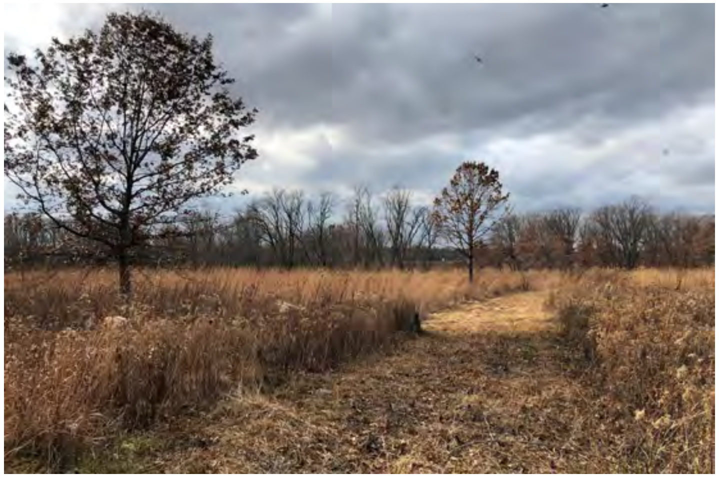

c. Mown turf, Prairie Inner Loop, Somme Prairie Grove (16.23)

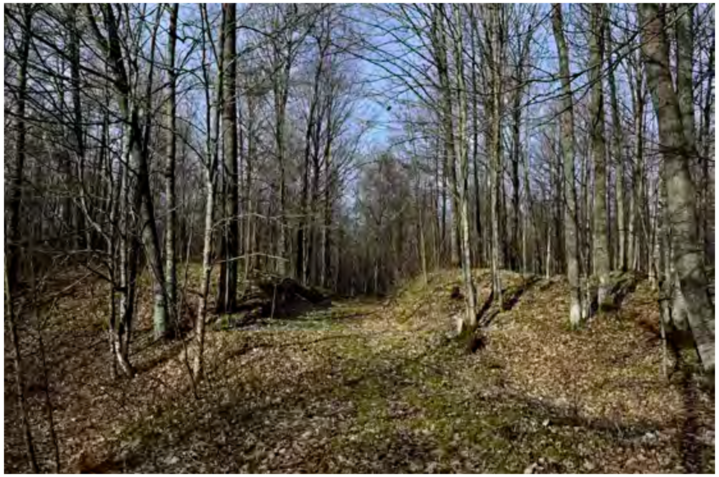

d. Mown turf, Middle Loop, Rainbow Hunter Walking Trails (3.52)

1.12. Surface– Imported Materials

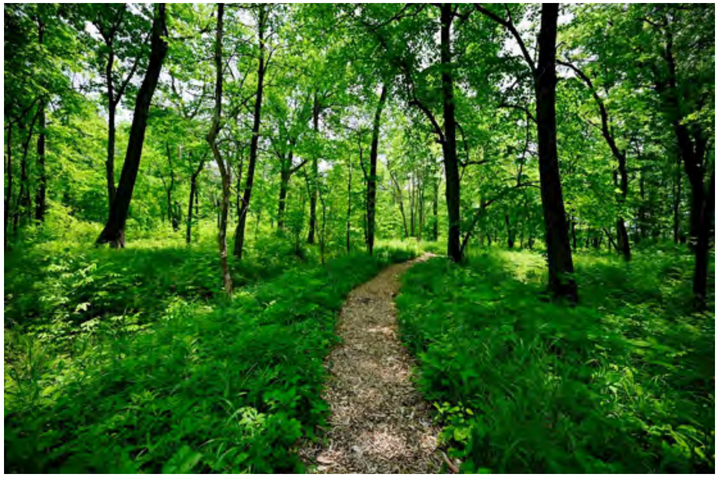

a. Woodchip, McDonald Woods North Loop, Chicago Botanic Garden (15.22)

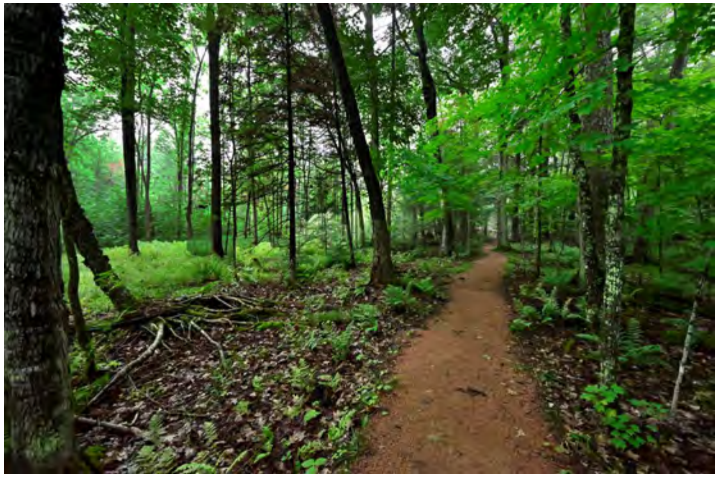

b. Crushed gravel, Franklin Nature Trail, Hidden Lakes Trail (6.11)

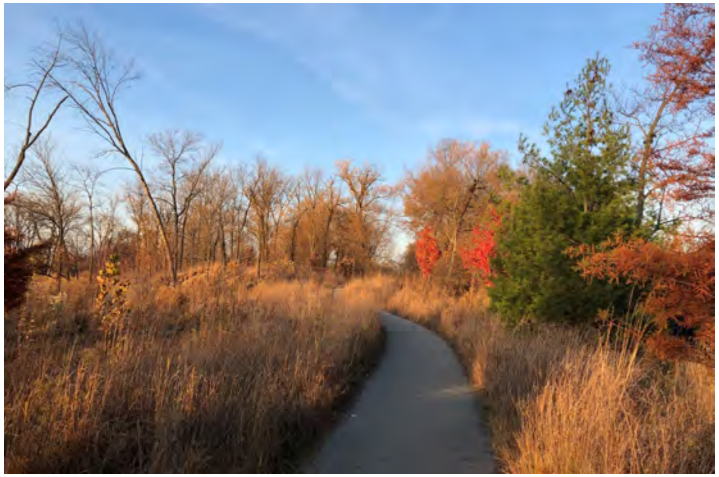

c. Asphalt, Lake Loop, West Ridge Nature Park (9.22)

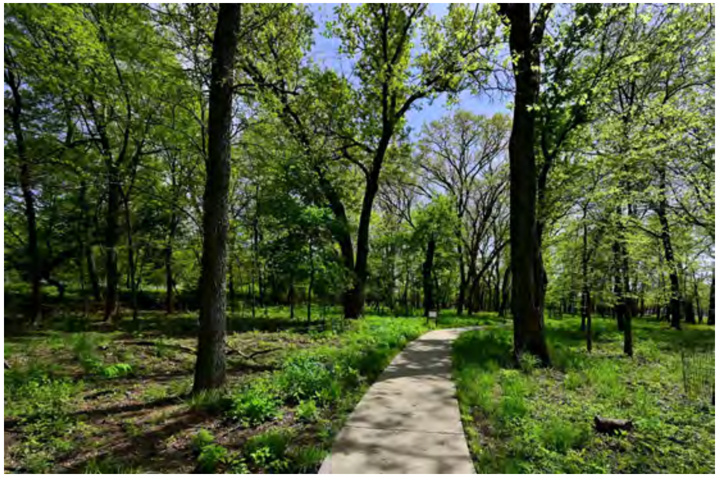

d. Concrete, Woodland Loop, West Ridge Nature Park (9.21)

1.13. Surface– Bike Trails

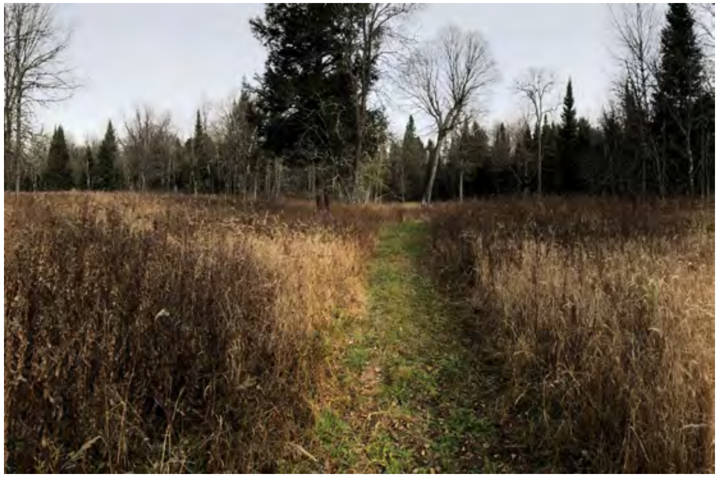

a. Mown turf, Chipmunk-Little Porky Loop, Lauterman National Recreation Trail (4.13)

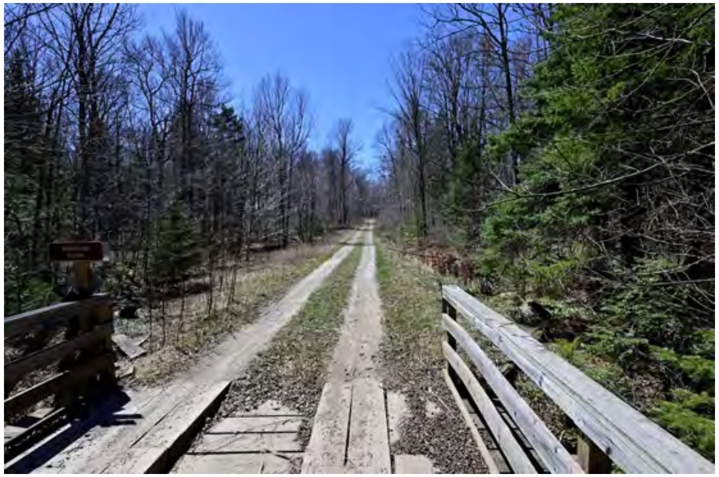

b. Dirt two-track, Brule River Cliffs Bike Loop, Brule River Cliffs (5.14)

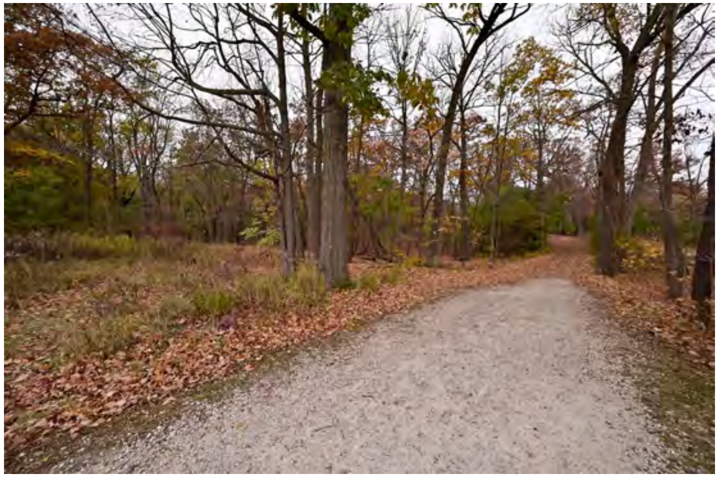

c. Crushed gravel, Harms Woods Bike Loop, Harms Woods Preserve Multi-Site (14.22)

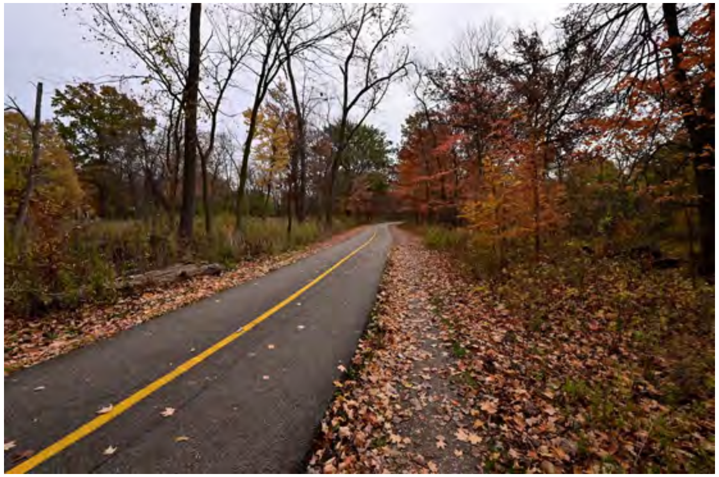

d. Asphalt, Bike Tour, Caldwell Preserves Multi-Site (13.42)

1.14. Surface– Sensuous Soft Surfaces

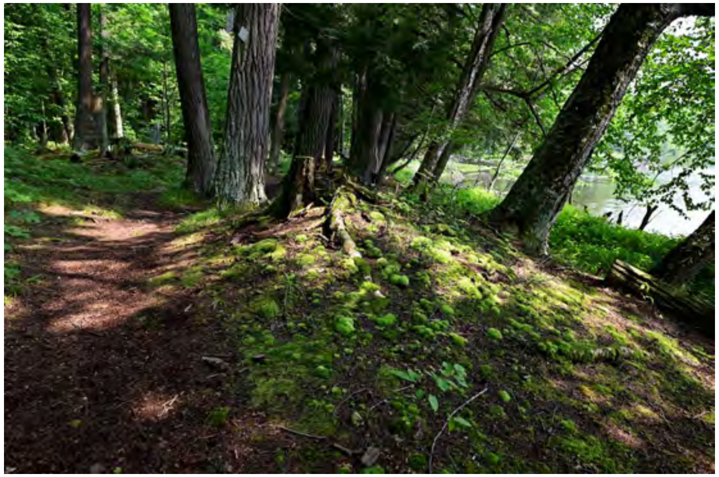

a. Moss along portion of West Lakeshore Trail, Lost Lake Recreation Area (4.25)

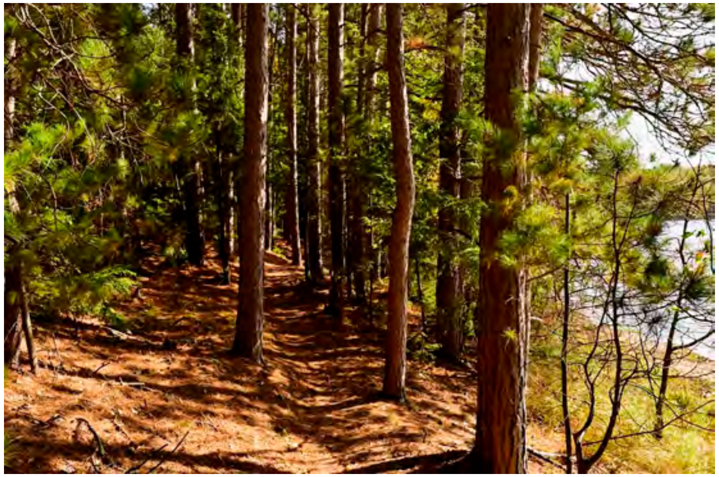

b. Pine needles along portion of McKinley Lake–Three Johns Lake segment, Hidden Lakes Trail (6.13)

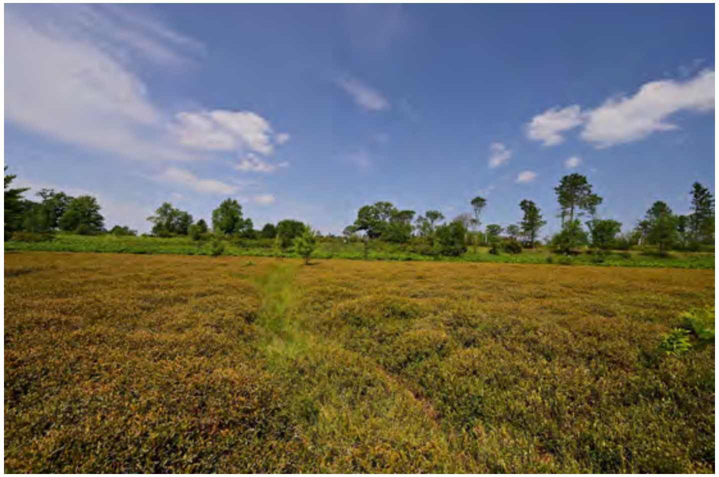

c. Bog mat, Bog Loop, Lake Anna (7.34)

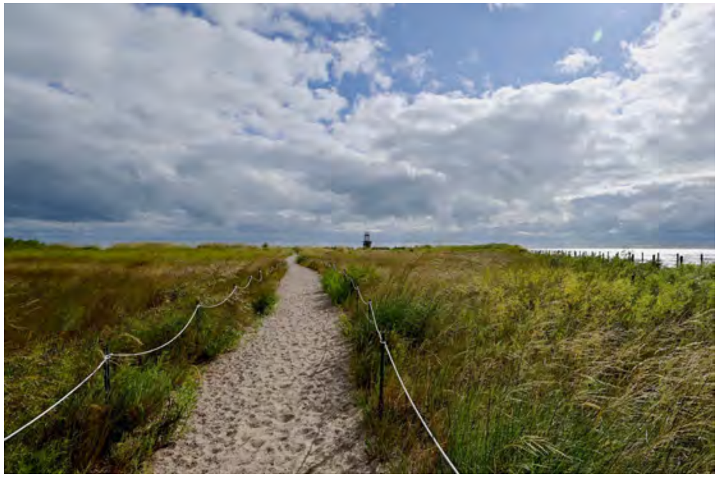

d. Sand, Loyola Natural Area-Pier Loop, Loyola-Leone Parks (10.22)

1.15. Surface– Sensuous Hard Surfaces

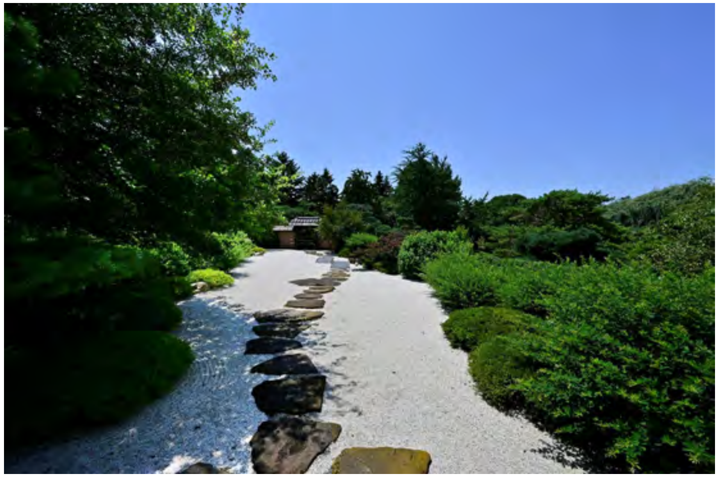

a. Stepping-stones, Japanese Garden, Chicago Botanic Garden (15.24)

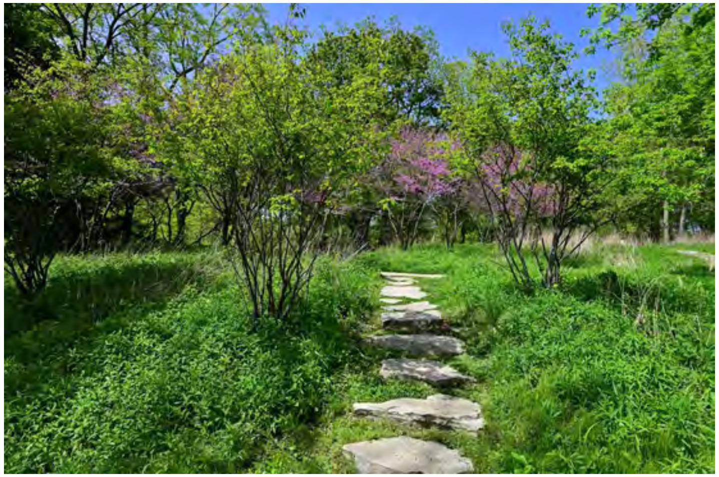

b. Stepping-stones, Woodland Loop, West Ridge Nature Park (9.21)

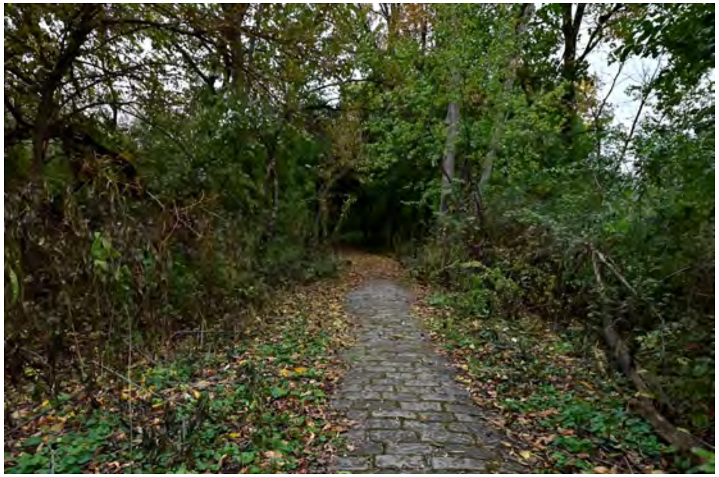

c. Brick, Walking Stick Woods Trail, North Park Village Nature Center (9.15)

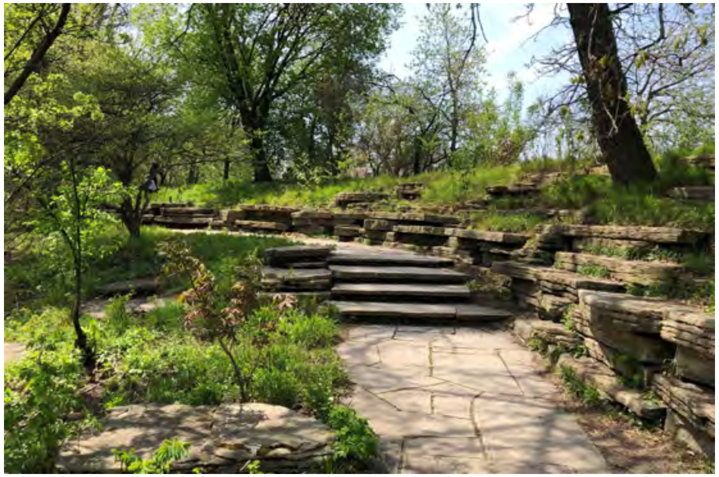

d. Stone, Lily Pool Loop, Alfred Caldwell Lily Pool (12.11)

1.21. Width– 1–2' Foot Trails

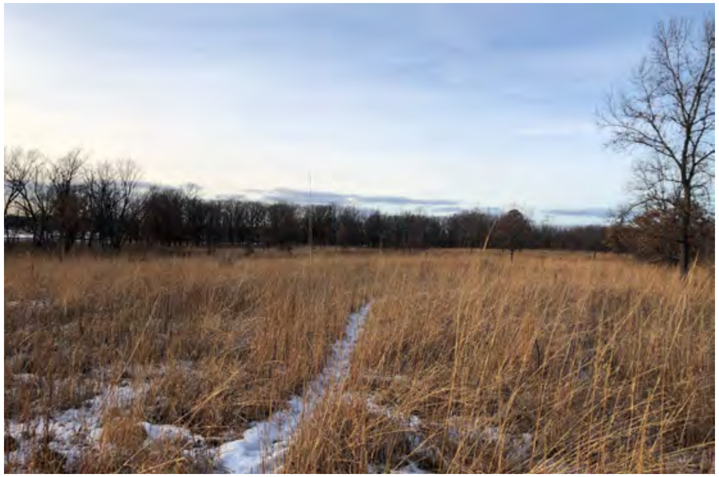

a. West Inner Loop (Winter), Somme Woods (16.31)

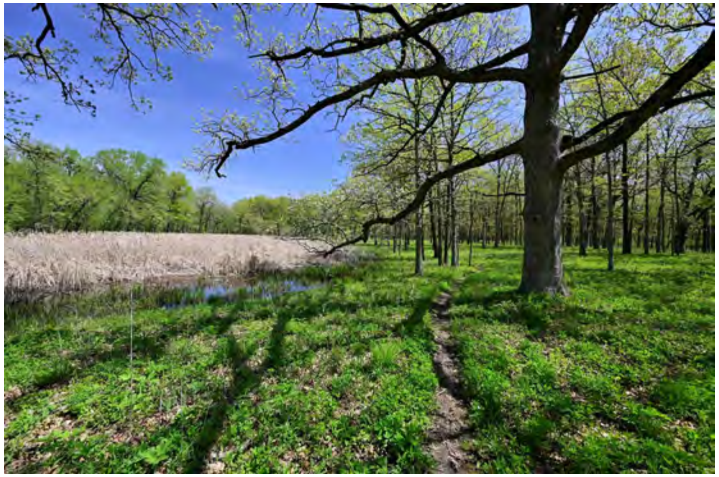

b. West Inner Loop (Spring), Somme Woods (16.31)

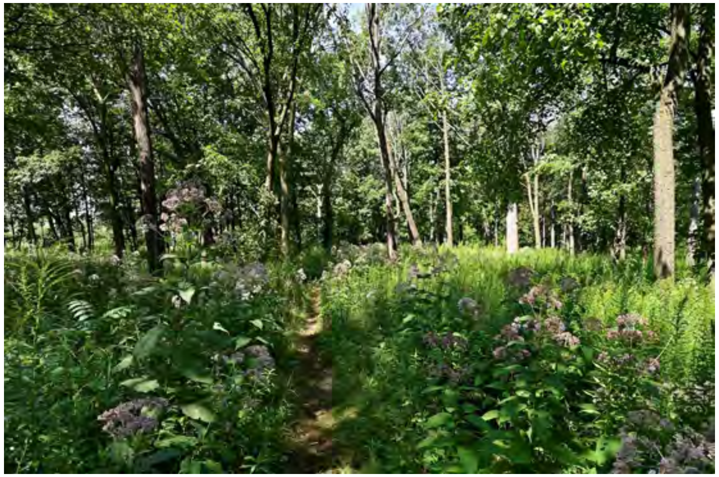

c. West Inner Loop (Summer), Somme Woods (16.31)

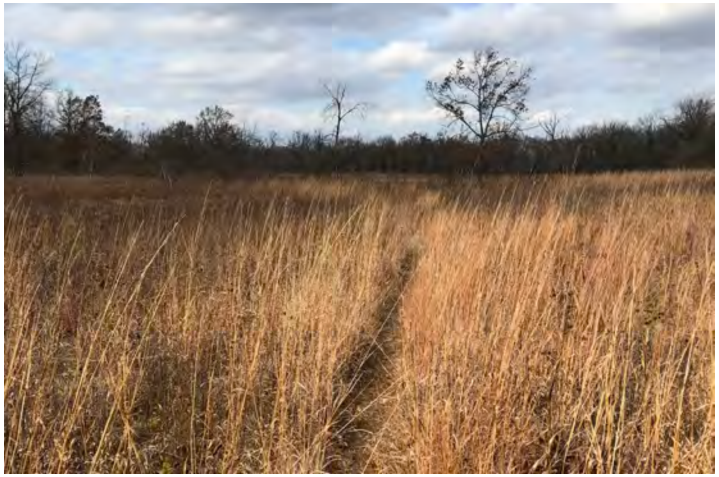

d. West Inner Loop (Fall), Somme Woods (16.31)

1.22. Width– 3–4' Foot Trails

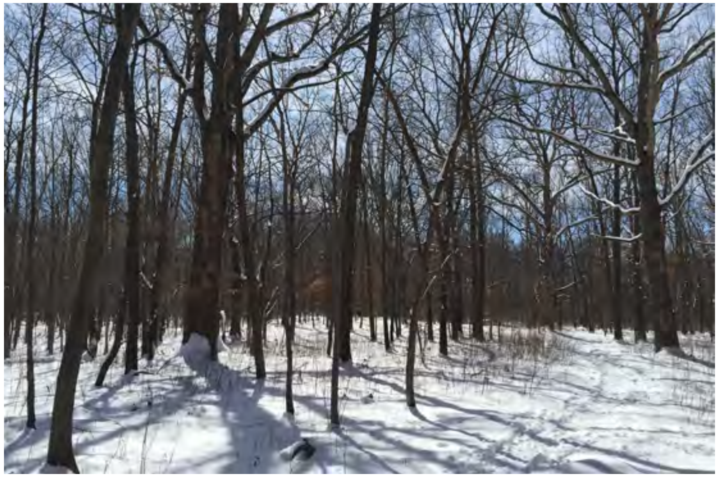

a. Woodland-River West Loop (Winter), Harms Woods Nature Preserve (14.11)

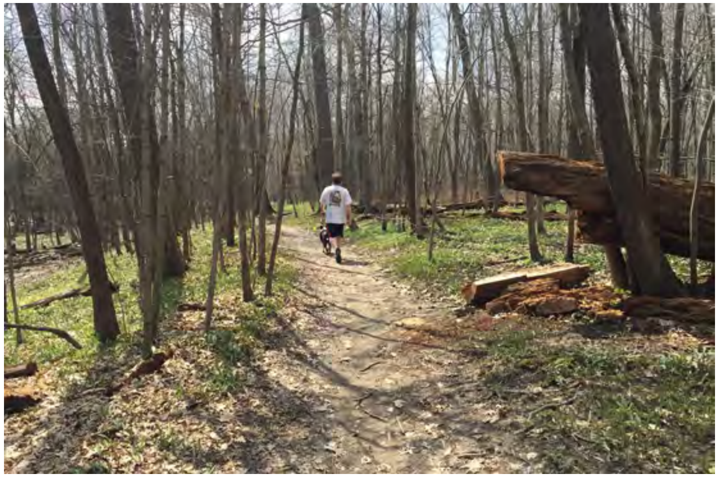

b. Woodland-River West Loop (Spring), Harms Woods Nature Preserve (14.11)

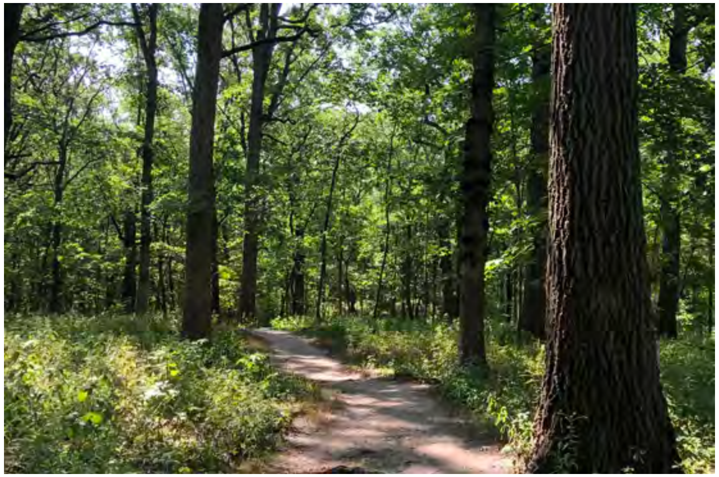

c. Woodland-River West Loop (Summer), Harms Woods Nature Preserve (14.11)

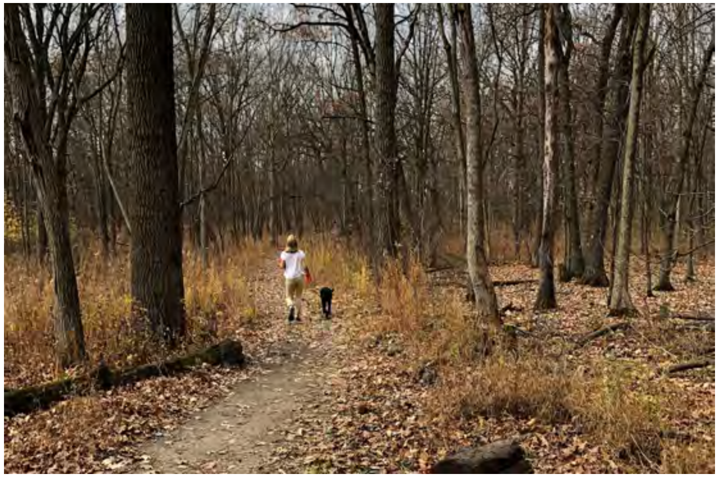

d. Woodland-River West Loop (Fall), Harms Woods Nature Preserve (14.11)

1.23. Width– 6–8' Foot Trails

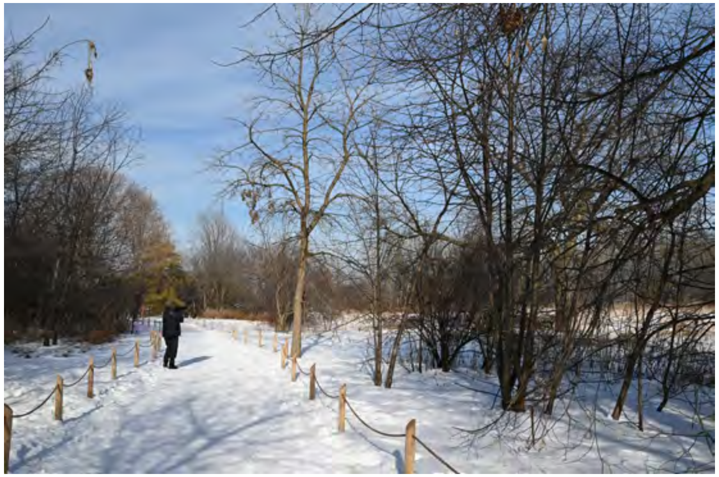

a. Bird Sanctuary Main Loop (Winter), Montrose Point (11.11)

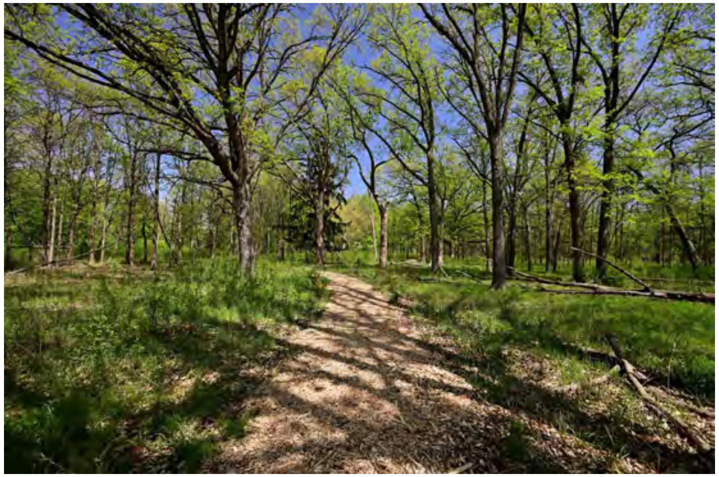

b. Wetland-Woodland-Savanna Loop (Spring), North Park Village Nature Center (9.13)

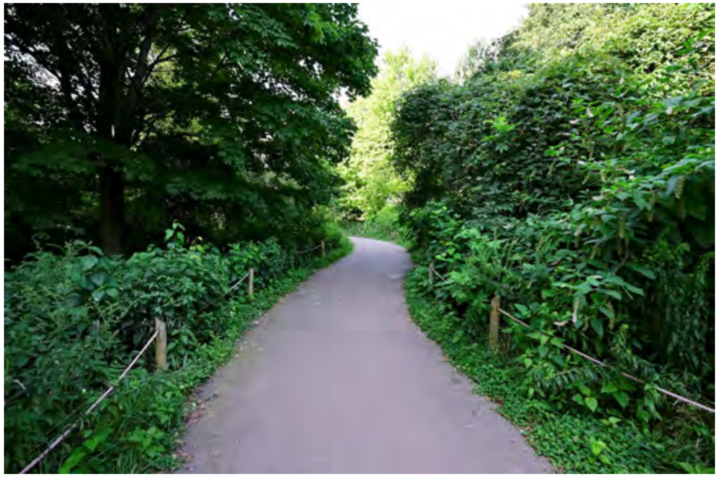

c. Bird Sanctuary Main Loop (Summer), Montrose Point (11.11)

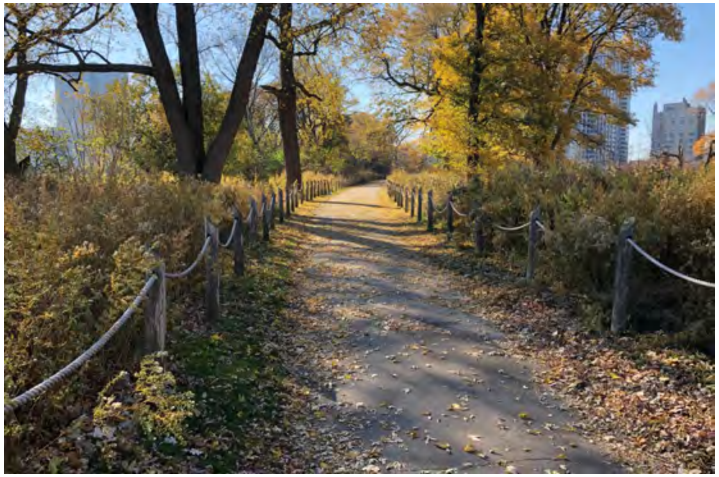

d. Savanna Loop (Fall), Marovitz Savanna (11.21)

1.24. Width– 10–15’ Foot Trails

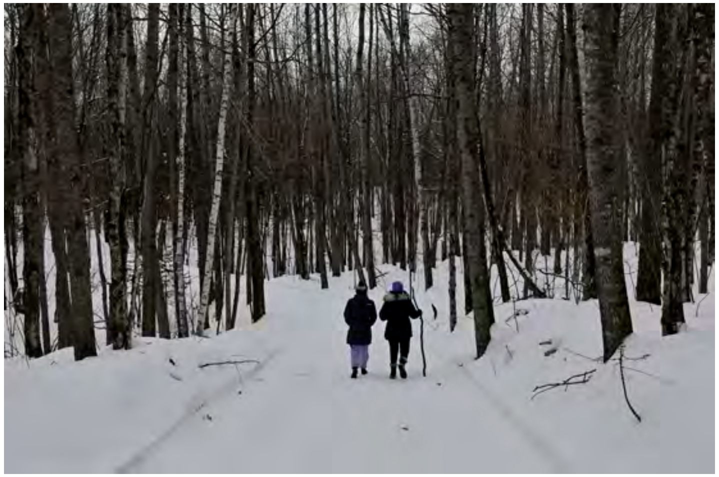

a. North Loop (Winter), Lake Emily Recreation Trail (2.21)

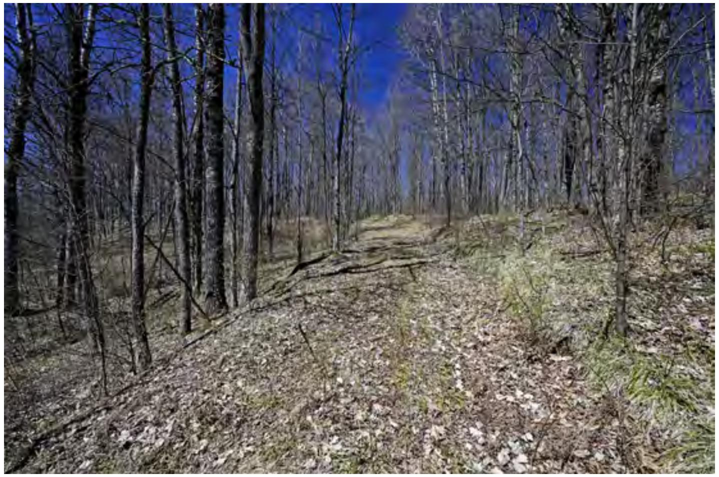

b. North Loop (Spring), Rainbow Hunter Walking Trail (3.51)

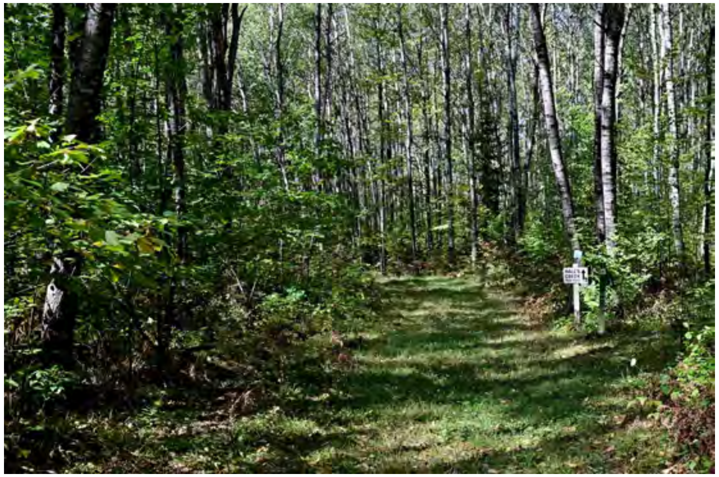

c. Green Loop (Summer), Halls Creek Trails (2.32)

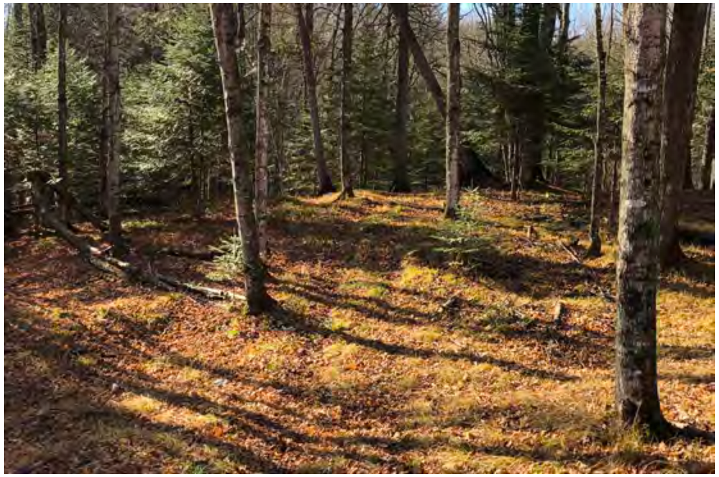

d. Lauterman Lake Trail Loop (Fall), Lauterman National Recreation Trail (4.1)

1.25. Width– 10–20’ Bike/Shared Use Trails

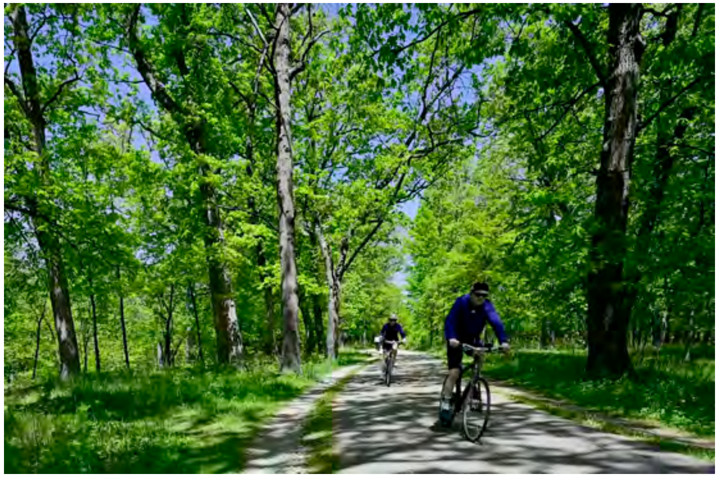

a. Bike Tour, Caldwell Preserves Multi-Site (13.42)

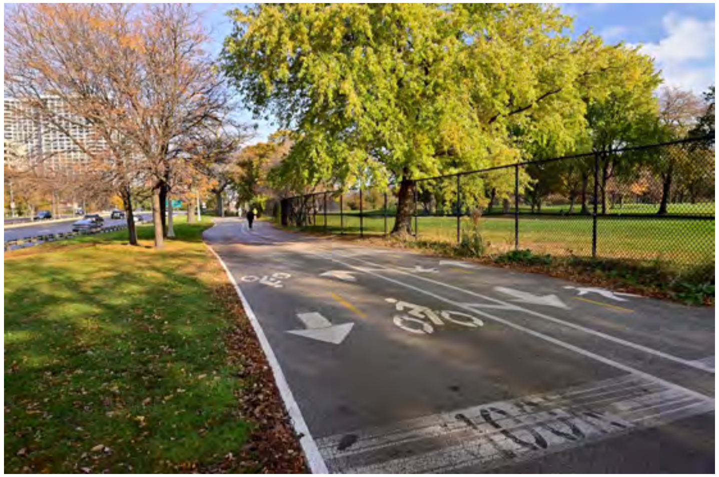

b. Montrose Area Bike Loop, Lincoln Park North Natural Areas Multi-Site (11.42)

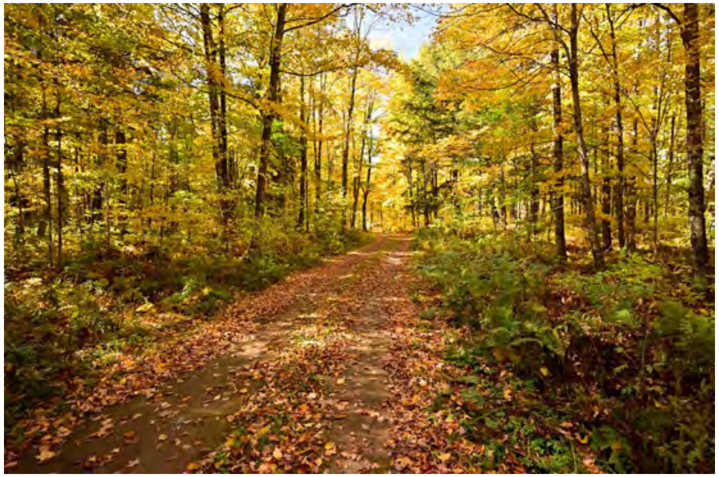

c. Savage Lake Road, Savage-Robago Wild Lakes Complex (1.85)

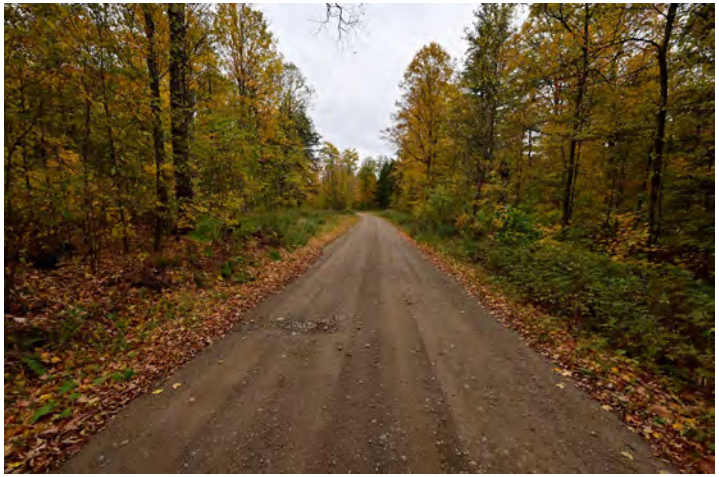

d. Wild Lakes Bike Loop, Savage-Robago Wild Lakes Complex (1.83)

1.26. Width– River Paddle Trails

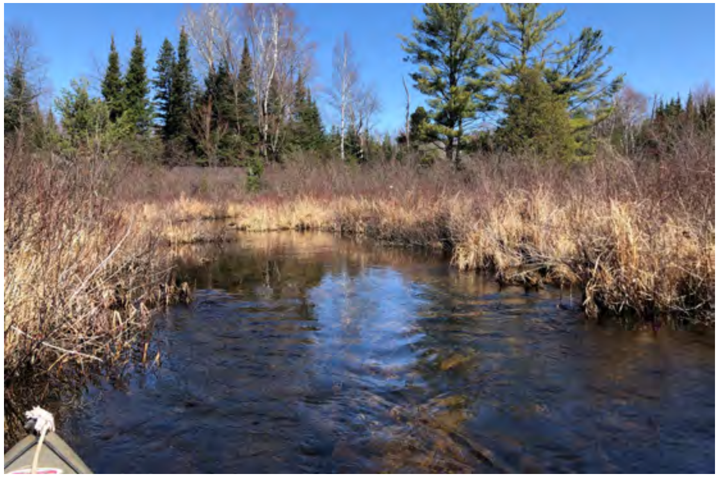

a. Islands and Bays Paddle, Sea Lion Lake (2.14)

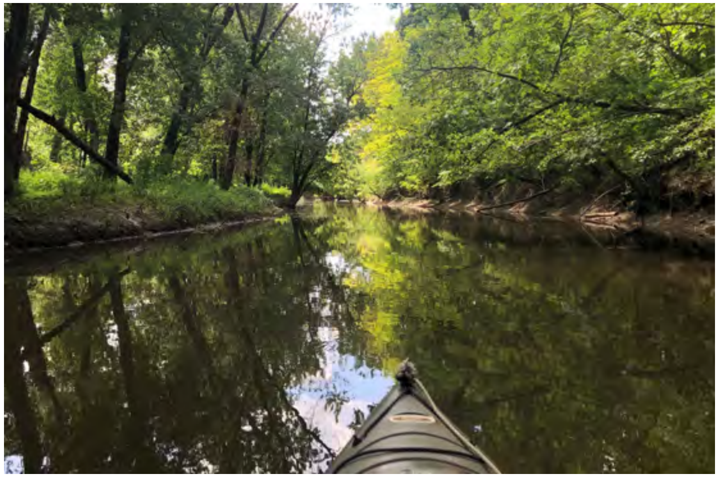

b. Chicago River Paddle, Harms Woods Preserves Multi-Site (13.41)

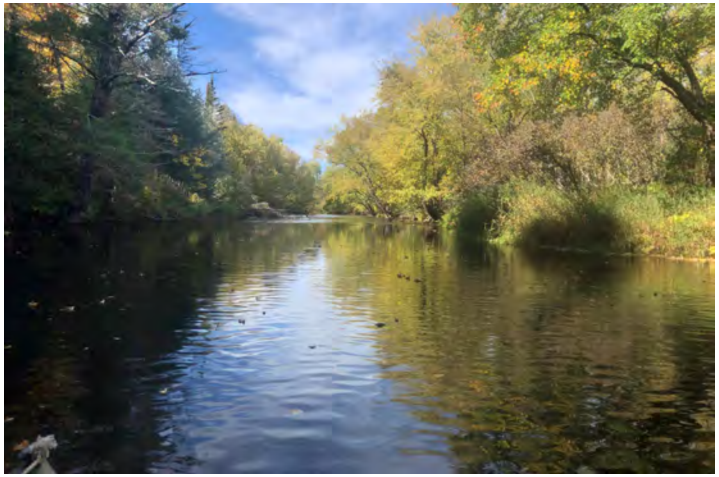

c. Flats Paddle, Pine River Flats (1.21)

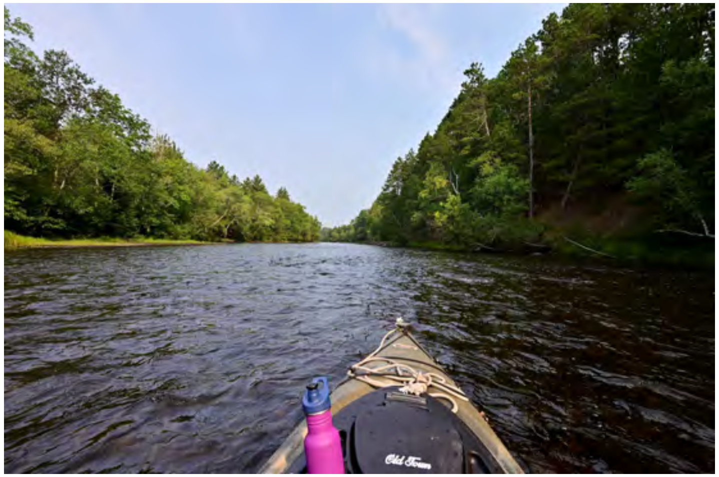

d. Oxbow Paddle, Pine River Oxbow (1.71)

1.27. Width– Lake Paddle Trails

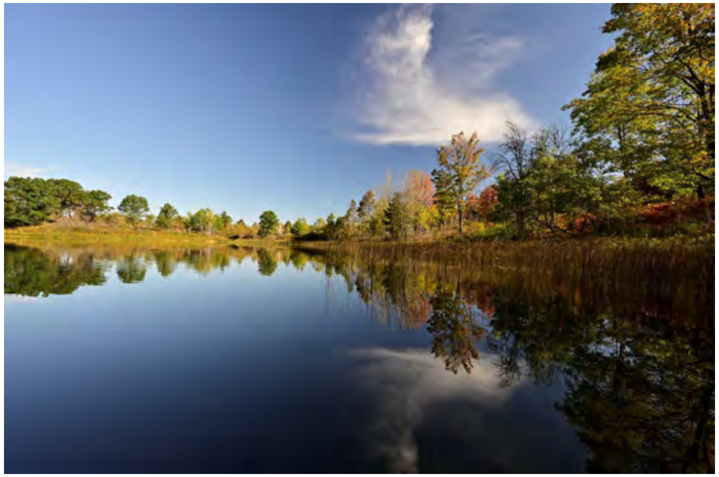

a. Barrens Lake Paddle, Barrens Lake (7.23)

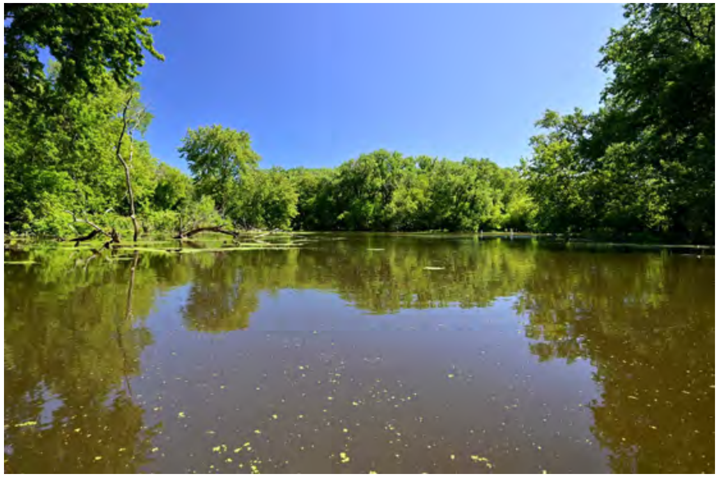

b. Lagoons 1-3 Loop, Skokie Lagoons Forest Preserve (15.17)

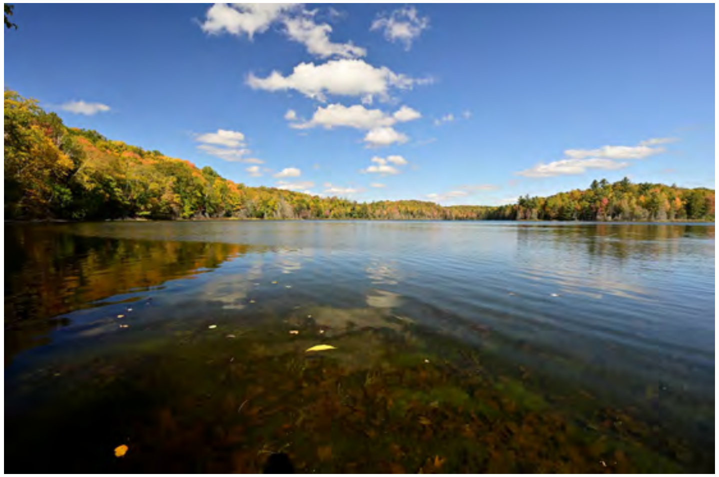

c. Quartz Lake Paddle, Hidden Lakes Dispersed Sites (6.24)

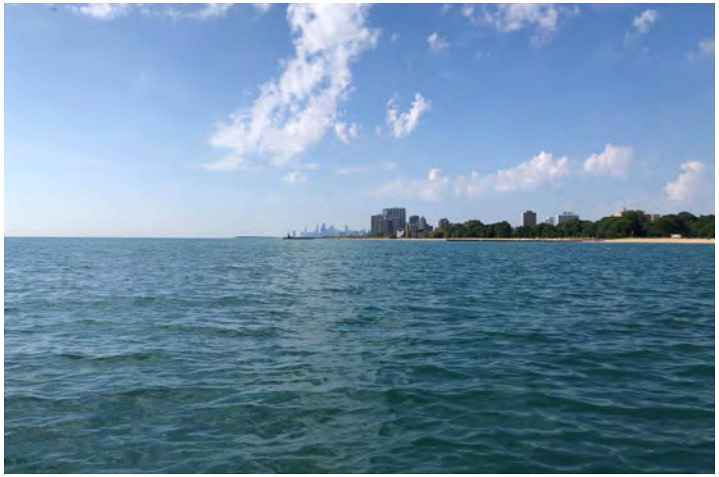

d. Waterfront Paddle, Loyola Lakeshore Natural Areas Multi-Site (11.41)

1.31. Barriers– Foot and Bike Trails

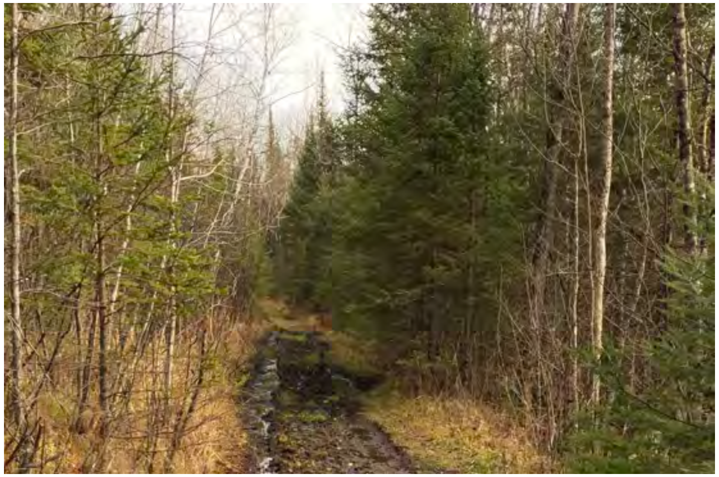

a. Mud, Chipmunk-Little Porky Loop, Lauterman Lake National Recreation Trail (4.13)

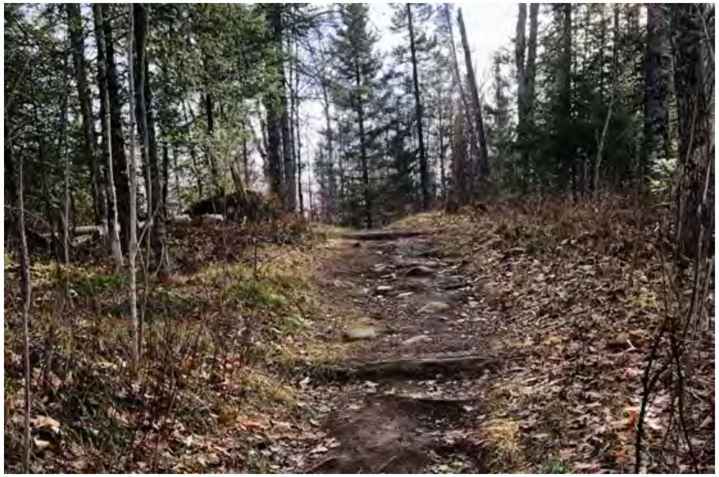

b. Exposed Roots and Stones, LaSalle Falls Trail, Pine River-La Salle Falls (1.51)

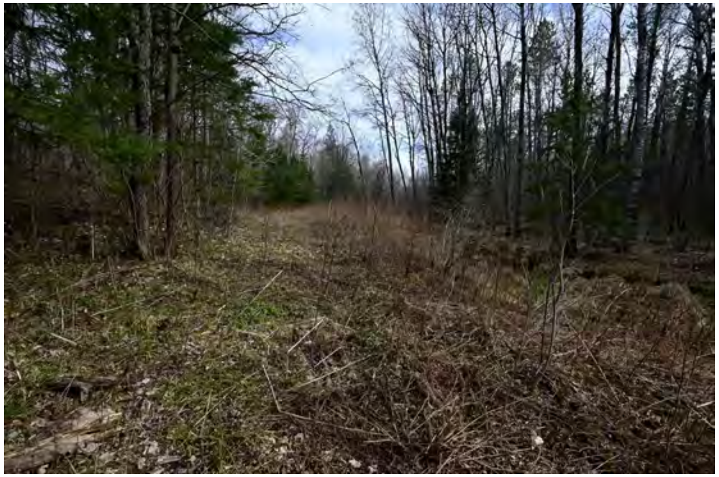

c. Brush, South Loop, Rainbow Hunter Walking Trails (3.53)

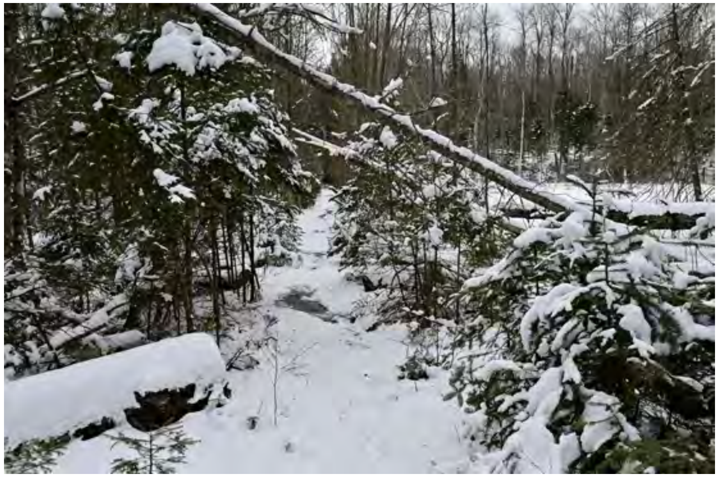

d. Deadfall, Riley Lake Trail portion, Whisker Lake Wilderness (3.42)

1.32. Barriers– Foot and Bike Trails

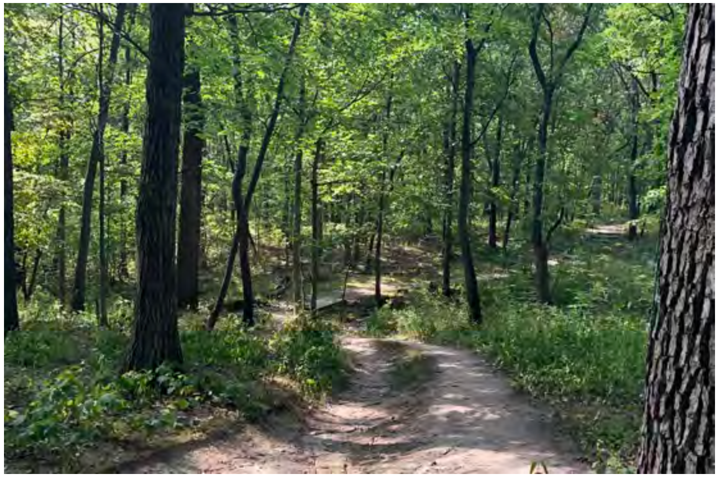

a. Erosion, Full Loop, Harms Woods Nature Preserve (14.14)

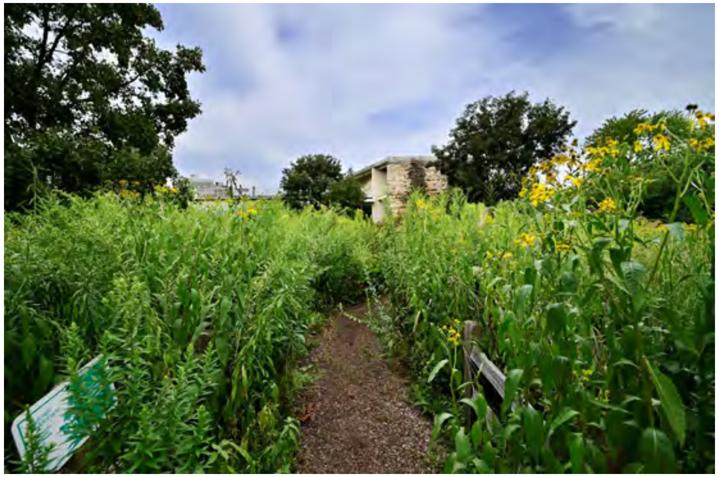

b. Overgrown Vegetation, Deb Lahey Nature Trails, Nature Museum (12.21)

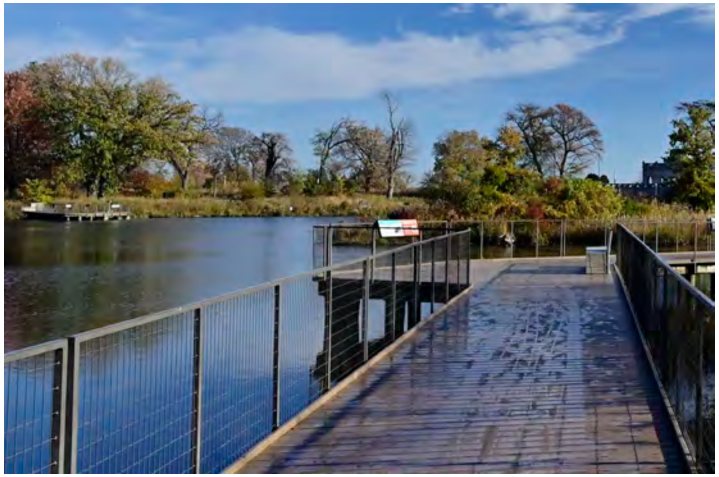

c. Slippery Wet Surface, Nature Boardwalk, Lincoln Park Zoo (12.41)

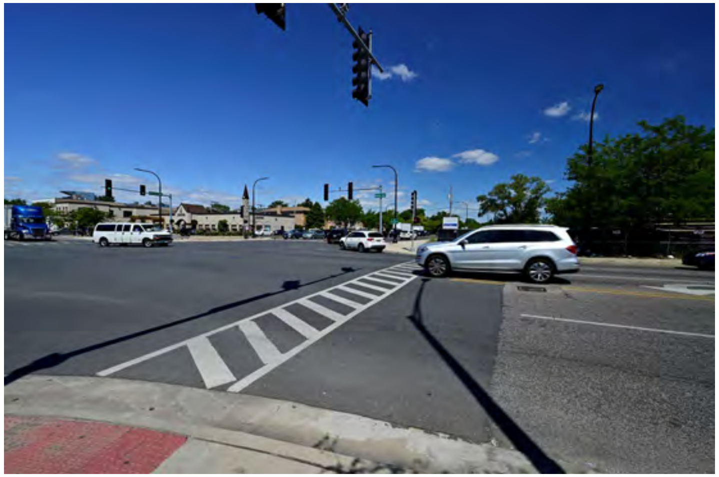

d. Busy Street Crossing, Bike Loop, West Ridge/ North Park Neighborhood Natural Areas Multi-Site (9.41)

1.33. Barriers– Paddle Trails

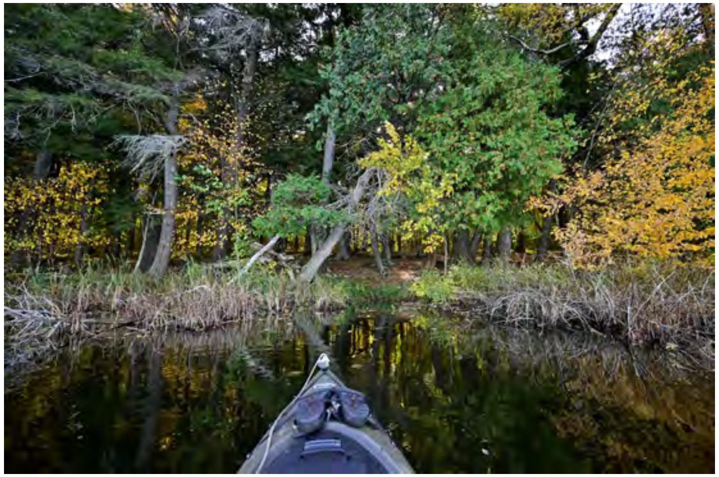

a. Unimproved Muddy Landing, Savage Lake Paddle, Savage-Robago Wild Lakes Complex (1.82)

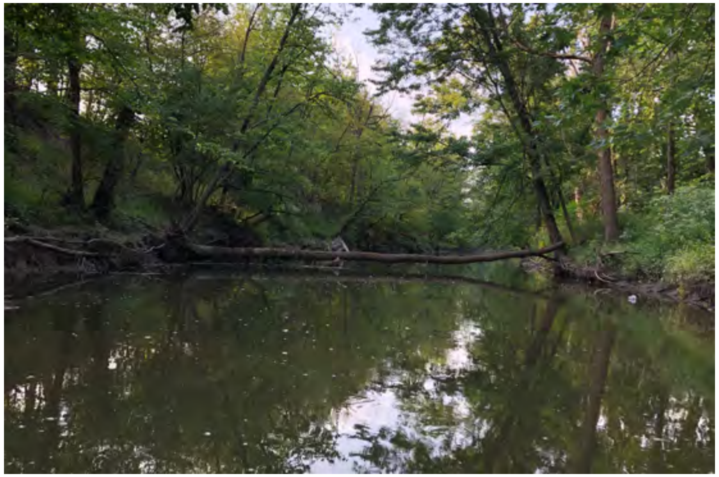

b. Fallen Trees, Chicago River Paddle, Caldwell Preserves Multi-Site (13.41)

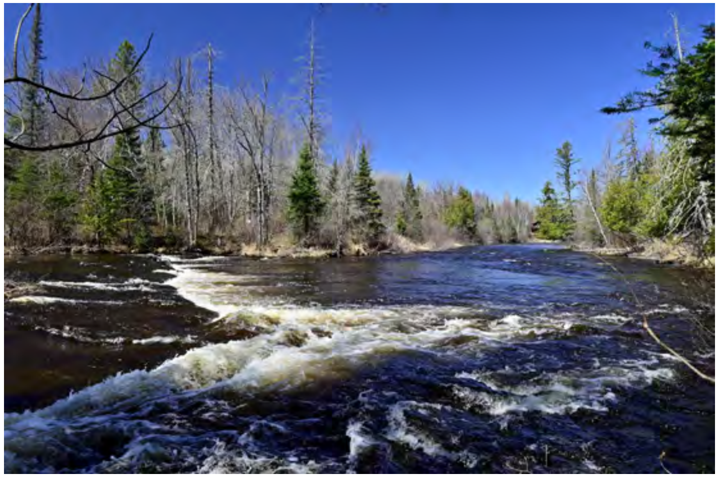

c. Rapids, Brule River Paddle, Brule River Cliffs (5.13)

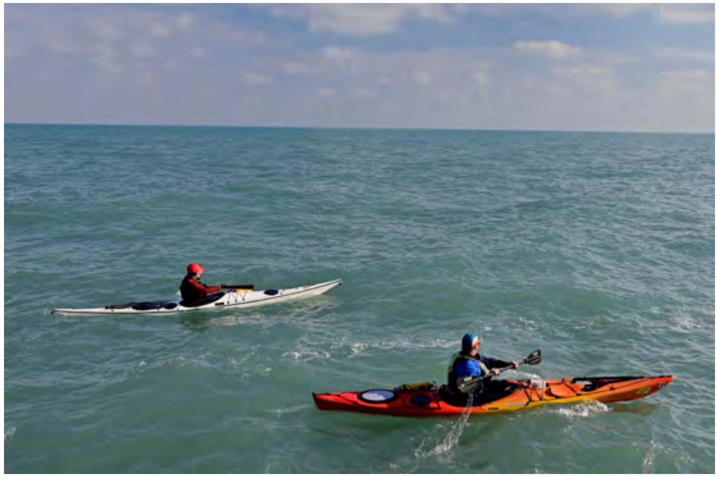

d. Waves, Waterfront Paddle, Lincoln Park North Natural Areas Multi-Site (11.41)

2. Attractiveness of Layout

2.1. *Alignment-* Alignment is an important aesthetic characteristic in the design or selection of trails for forest therapy. While former railroad (2.11a) and road (2.11b) corridors with linear alignments may have interpretive value as historic features, their long straight stretches have limited experiential value as foot trails, though other changes in landcover and views may minimize monotony when linear stretches form part of a bike trail (2.11c–d). In contrast, curvilinear alignments add a sense of mystery to the journey, anticipation of what lies ahead on foot and bike trails (2.12a–c) and an added dimension of motion in the case of bike trails (2.12d). The same reasoning applies to paddle trails, where simple lake and river shorelines can be monotonous (2.13a–b) while more complex ones provide mystery and opportunities for exploration (2.13c–d).

2.2. *Views-* This sequence illustrates major view types along trails in the two study areas. Trees and other tall vegetation in sufficient density can create canopy and enclosed views (2.21), affording a secure feeling of refuge and leading the eye down the trail corridor. Landforms can do the same, putting the visitor in an “inferior” position looking up into the surrounding landscape (2.22). In contrast, land and waterscapes such as flat prairies and large lakes can provide open panoramic views (2.23) for long distance prospects and skywatching but afford little refuge from weather and are vulnerable to impacts from adjacent land use. Hilltops and other elevated, “superior” views (2.24) can also offer distant views, especially during leaf-off periods (2.24b), while in flatter areas elevations created by bridges, berms, and observation decks (2.24c–d) provide good substitutes (see also Built Features below). Finally, many natural features (see also below) afford detail views (2.25) of distinctive colors, patterns, and textures of objects when seen close-up.

2.3. *Spaces-* Private spaces (2.31) in the form of grassy hilltops, rocks, logs, benches and other natural and built seating areas, located on spurs off the main flow of trails, provide good “sitspots” for observation and reflection, especially when they afford both qualities of prospect and refuge. Larger grassy openings and seating areas along or off the trail are needed for group spaces (2.32) for guided “invitations” (group activities conducted as part of a forest therapy walk), and while privacy may be less of an issue, some separation from traffic flow is desirable so people do not feel inhibited in participating.

2.4. *Changes-* The first four panels in this sequence illustrate changes along the routes of four different trails in the two study areas. The experience of spatial changes can be accomplished in various ways, by landcover and vegetation types (2.41), landform changes (2.42), trail surfaces (2.43), rock and water patterns (2.44) and other patterns and features that, alone or in combination, create noticeably different “rooms” or "reaches." Change can also be experienced temporally, either short-term (2.45) or over successive visits (2.46), from key observation points at a site or along a trail.

2.11 Alignment– Linear Foot and Bike Trails

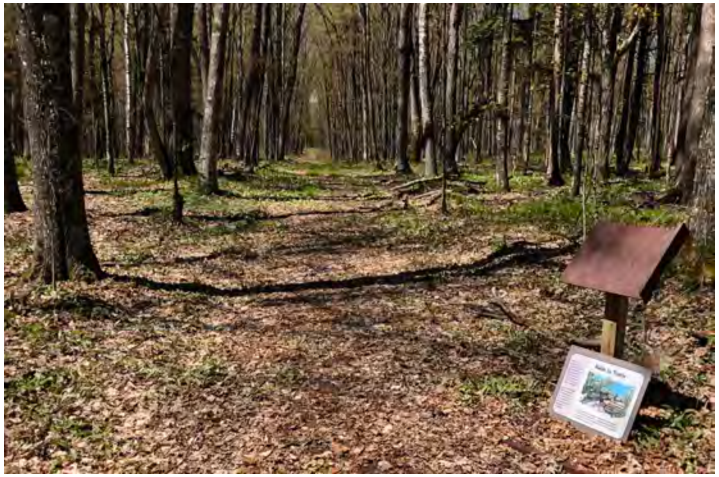

a. Assessor's Interpretive Trail Loop, Lost Lake Recreation Area (4.22)

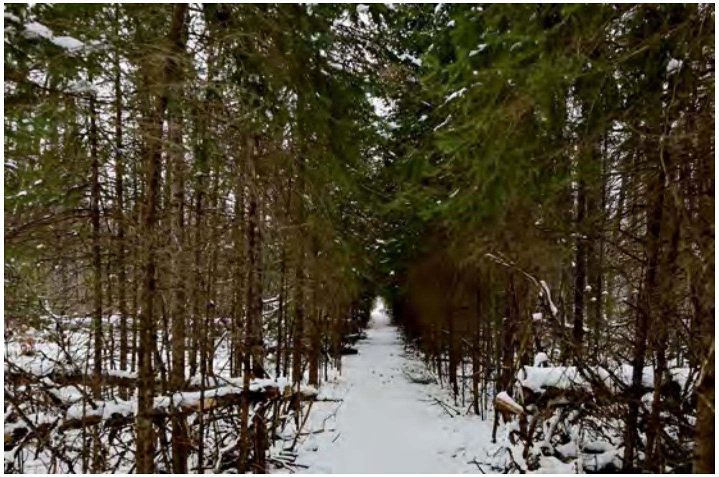

b. Riley Lake Trail portion, Whisker Lake Wilderness (3.42)

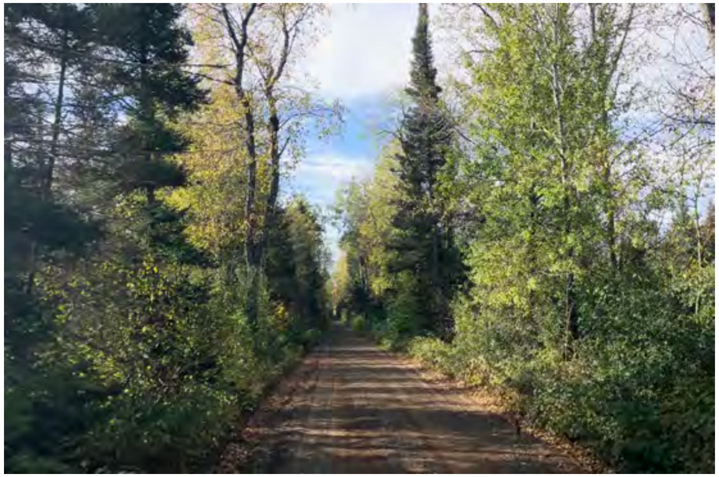

c. Bike Loop, Brule River Cliffs (5.14)

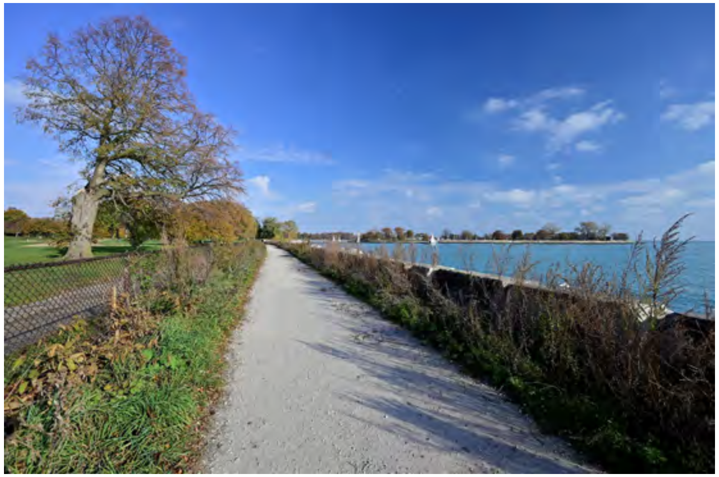

d. Montrose Area Bike Loop, Lincoln Park North Natural Areas Multi-Site (11.42)

2.12. Alignment– Curvilinear Trails

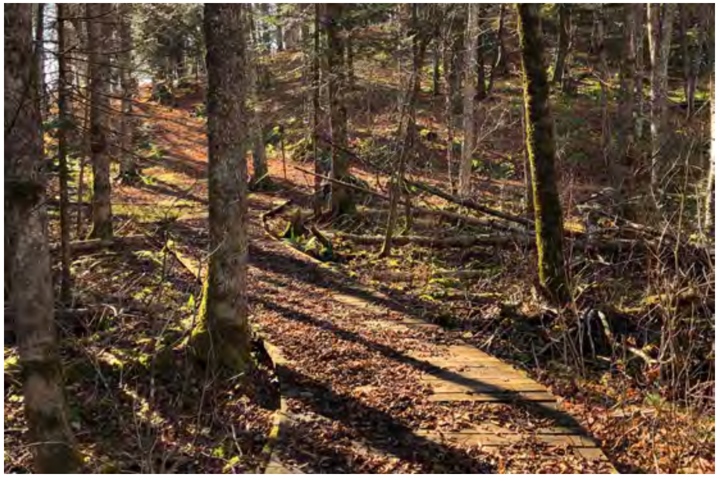

a. Lauterman Lake Trail Loop, Lauterman National Recreation Trail (4.12)

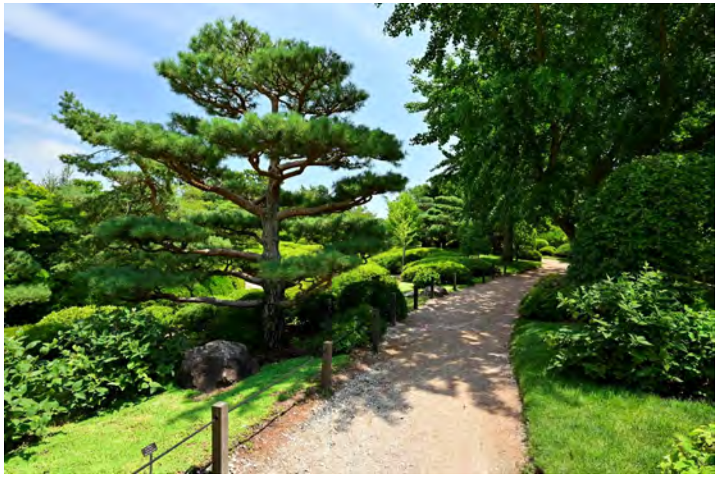

b. Japanese Garden, Chicago Botanic Garden (15.24)

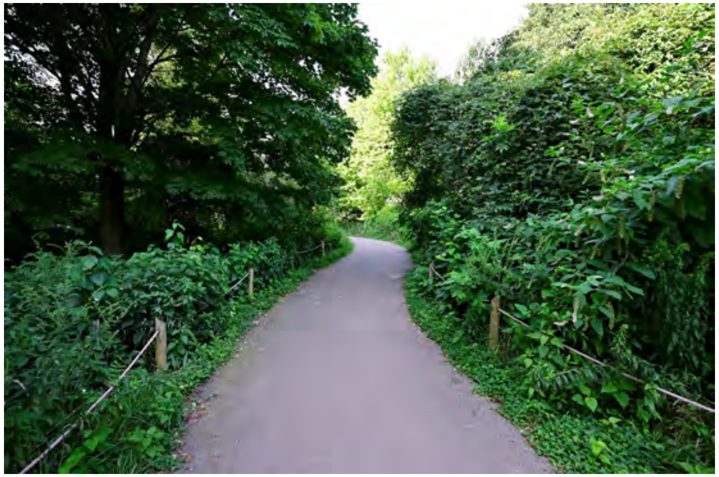

c. Bird Sanctuary Main Loop, Montrose Point (11.11)

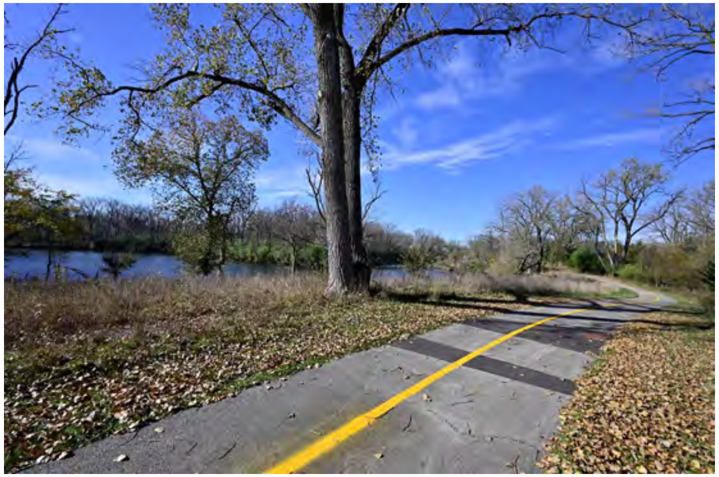

d. North Branch Trail Bike Loop, Skokie Lagoons Forest Preserve (15.18)

2.13. Alignment– Simple & Complex Shoreline Paddle Trails

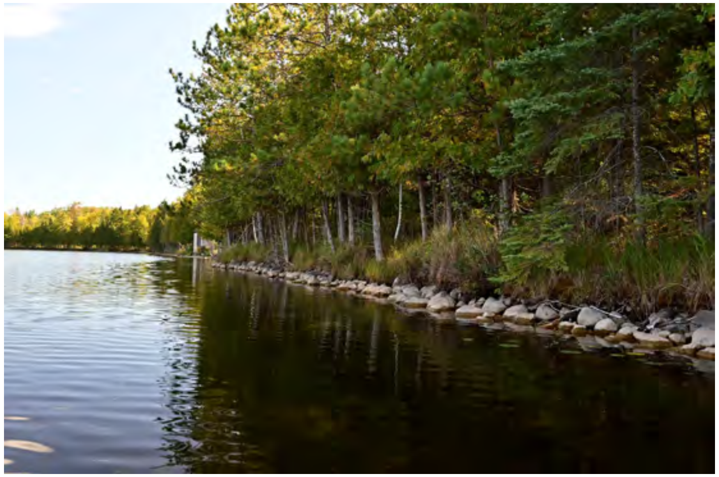

a. Shoreline Paddle, Little Fumee Lake (8.12)

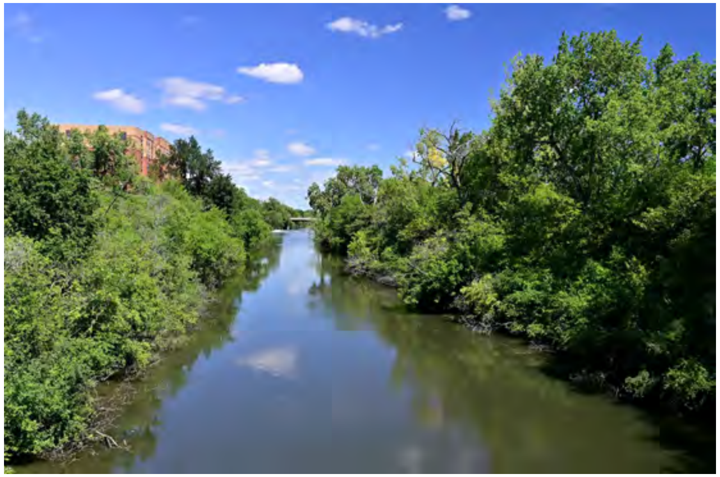

b. North Shore Channel as seen from Bike Loop, West Ridge/North Park Neighborhood Natural Areas Multi-Site (9.41)

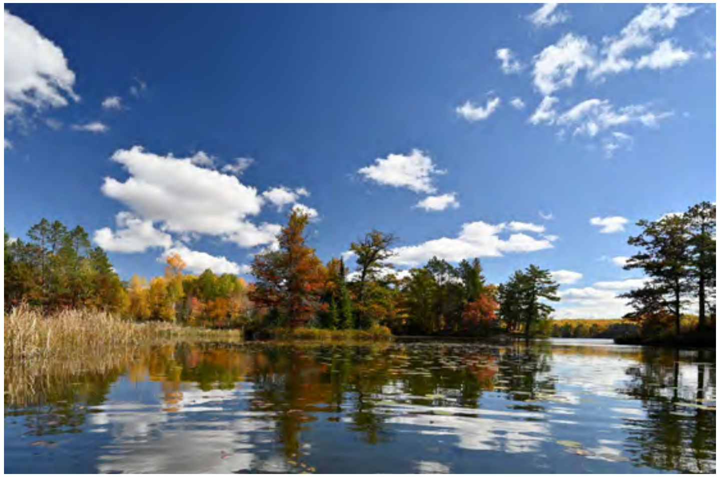

c. Islands and Bays Paddle, Sea Lion Lake (2.14)

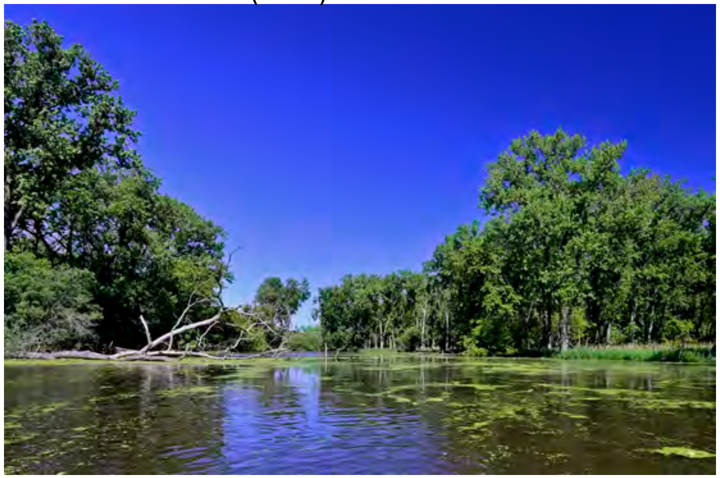

d. Lagoons 4-5 Loop, Skokie Lagoons Forest Preserve (15.15)

2.21. Views– Canopy and Enclosed Views

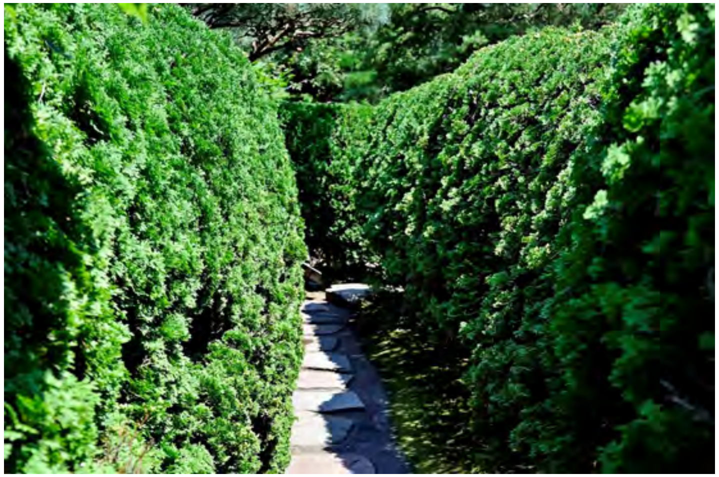

a. Japanese Garden, Chicago Botanic Garden (15.24)

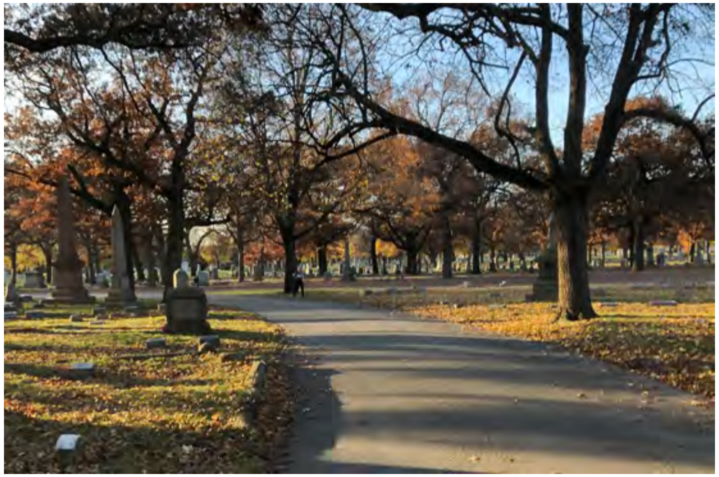

b. Tree Meander, Rosehill Cemetery (9.24)

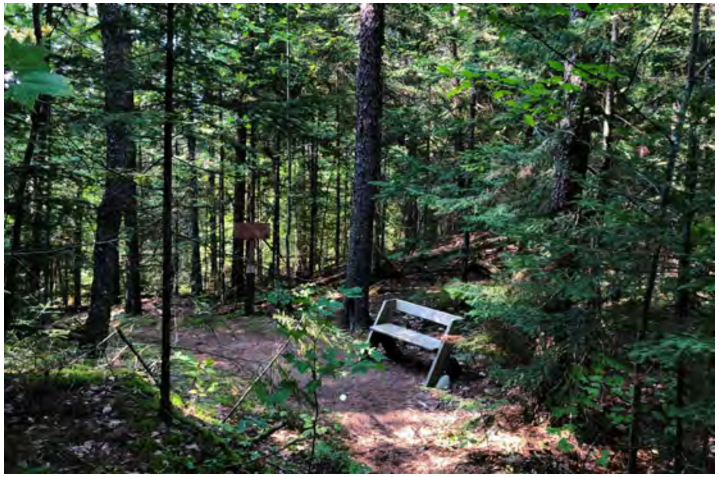

c. Sam Campbell Memorial Trail, Hidden Lakes Other Trails of Interest (6.32)

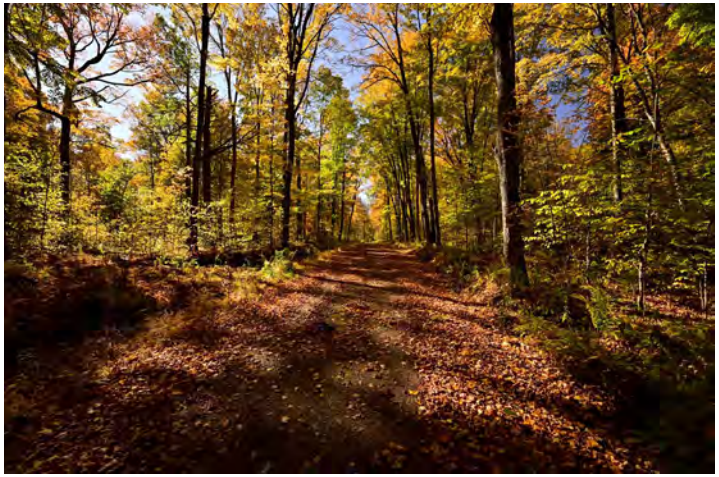

d. Savage Lake Road, Savage-Robago Wild Lakes Complex (1.85)

2.22. Views– Inferior Enclosed Views

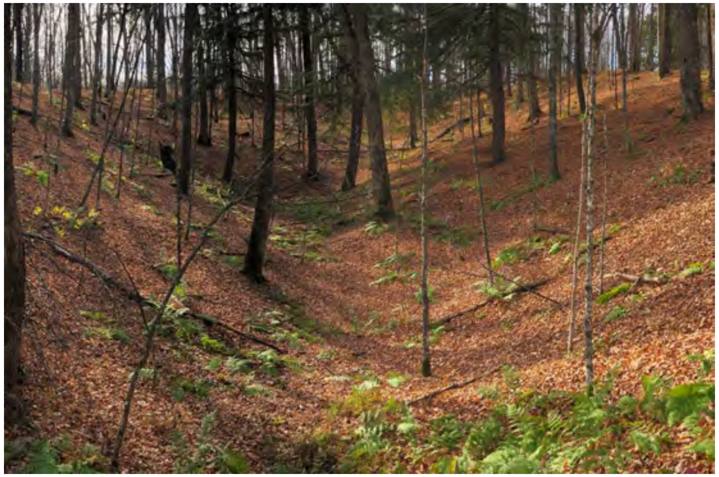

a. Chipmunk-Little Porky Loop, Lauterman National Recreation Area (4.13)

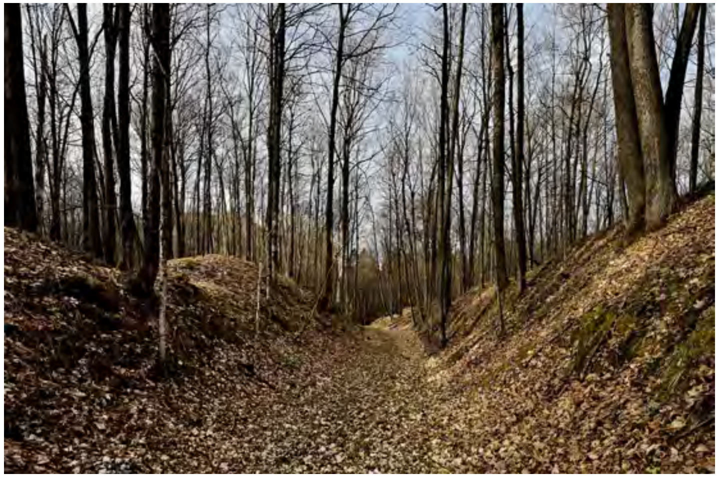

b. South Loop, Rainbow Hunter Walking Trails (3.53)

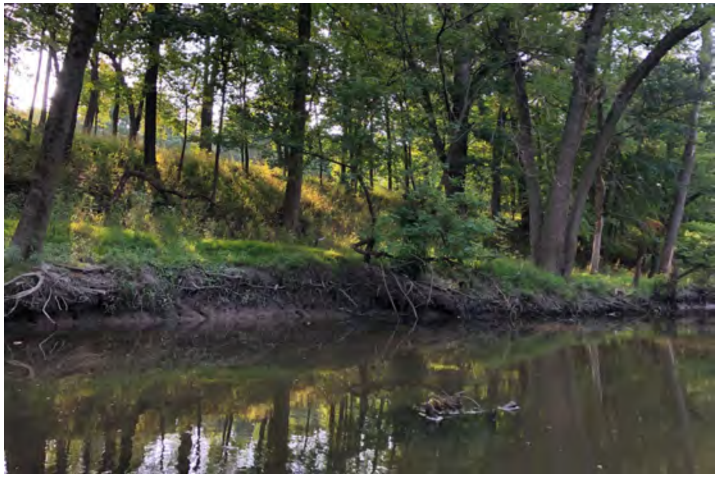

c. Chicago River Paddle, Caldwell Preserves Multi-Site (13.41)

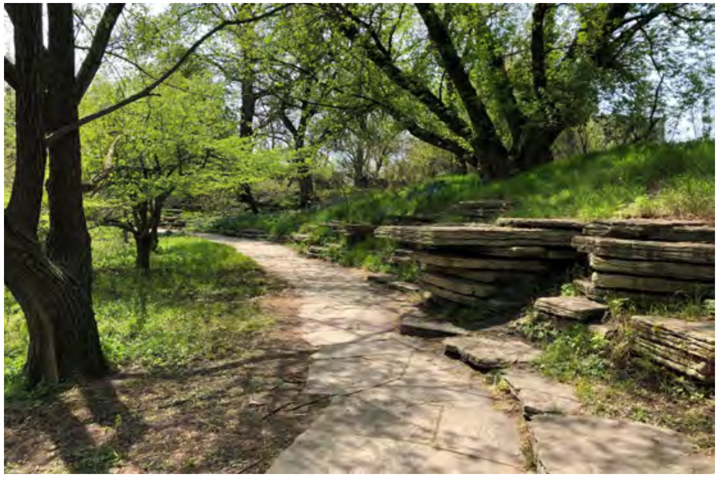

d. Lily Pool Loop, Alfred Caldwell Lily Pool (12.11)

2.23. Views– Open Panoramic Views

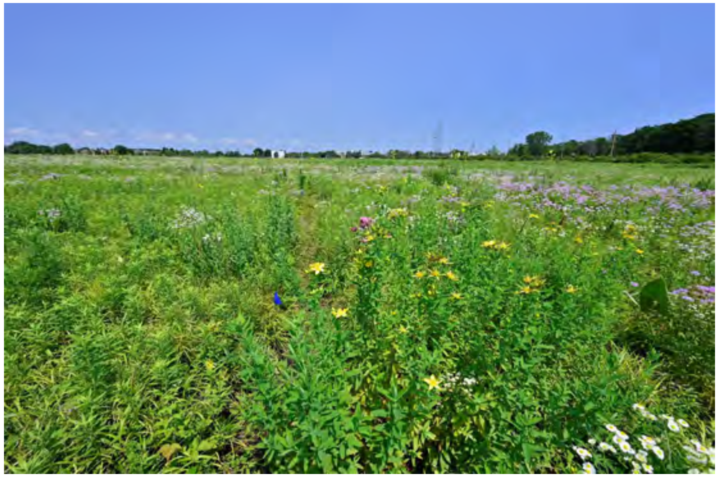

a. Prairie Loop, Somme Prairie (16.11)

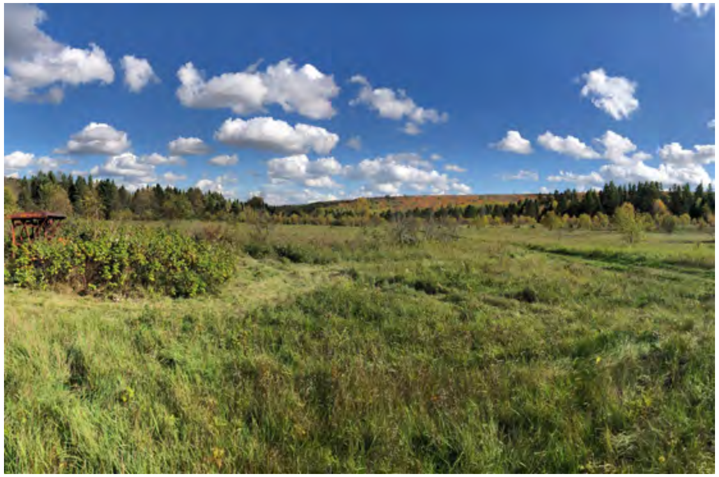

b. Old Field Loop, Brule River Cliffs (5.11)

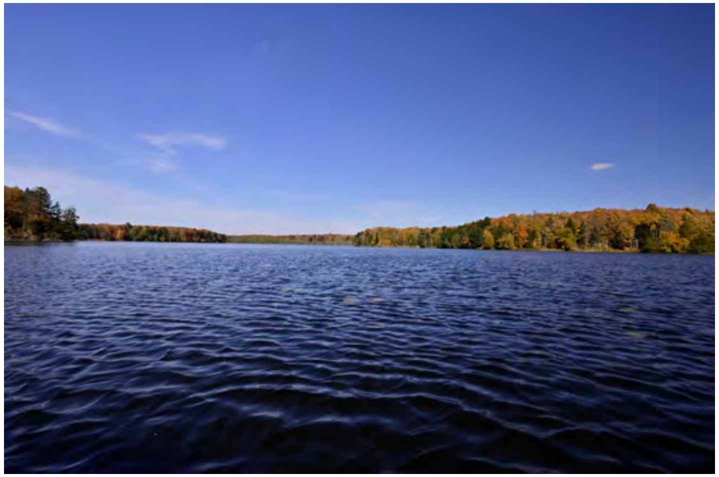

c. Savage Lake Paddle, Savage-Robago Wild Lakes Complex (1.82)

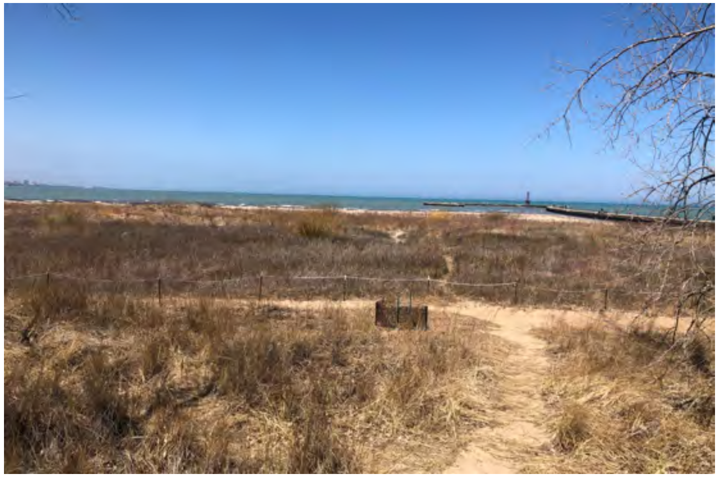

d. Point-Dunes-Lake-Prairie Loop, Montrose Point (11.13)

2.24. Views– Superior Elevated Views

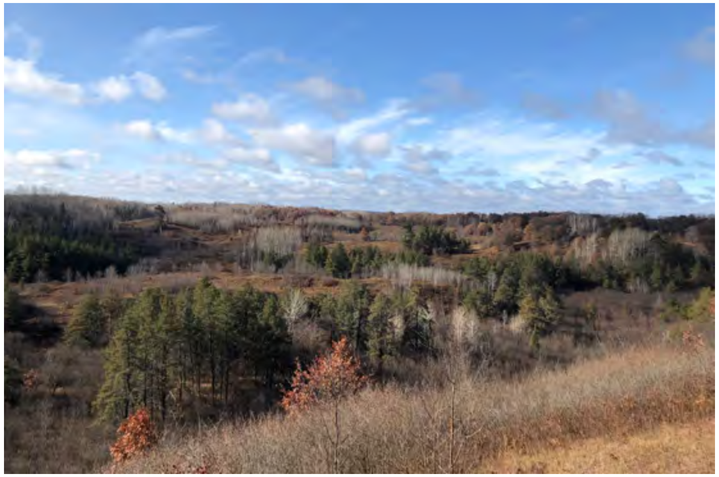

a. Lepage Creek Overlook, Lake Anna (7.35)

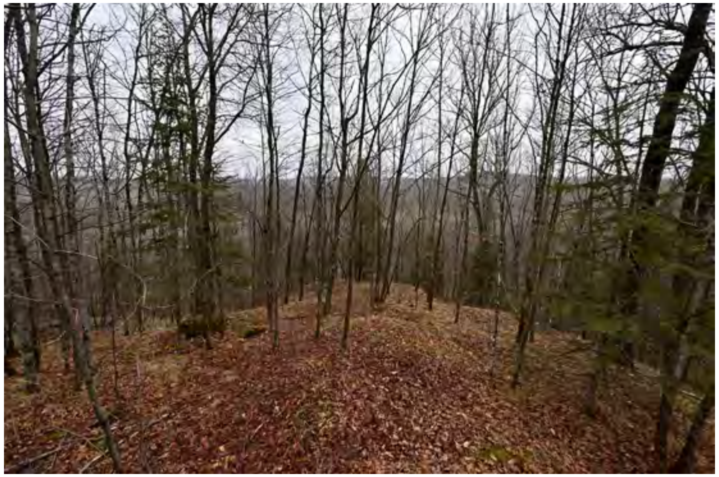

b. Lakeshore-Ridge Trail Loop, Lost Lake Recreation Area (4.23)

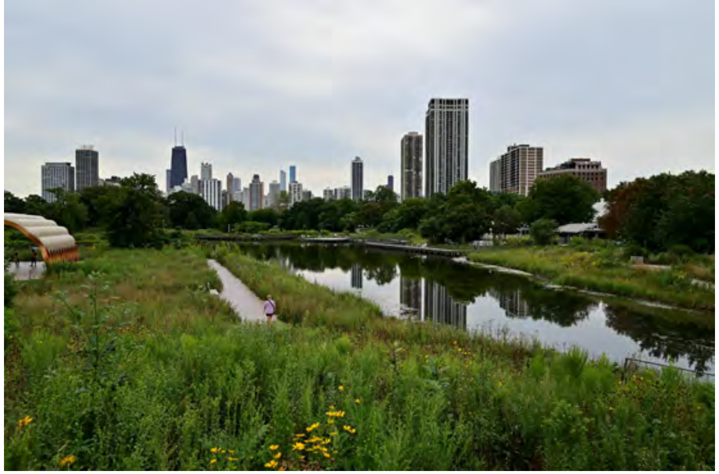

c. Nature Boardwalk, Lincoln Park Zoo (12.41)

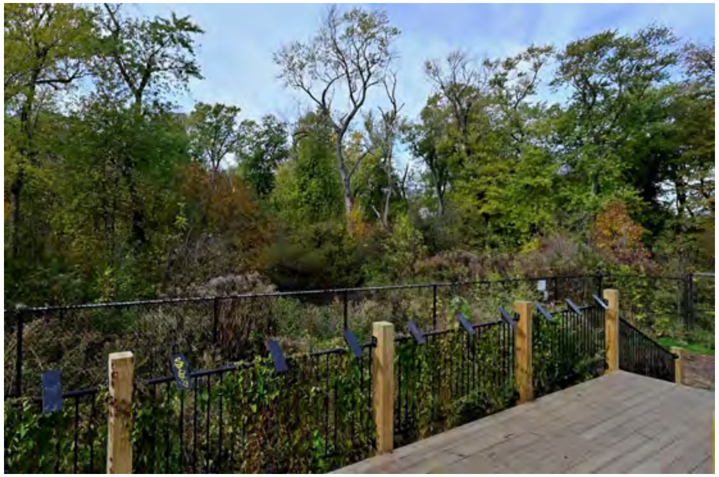

d. Bird Sanctuary Loop, Bill Jarvis Migratory Bird Sanctuary (11.31)

2.25. Views– Detail Views

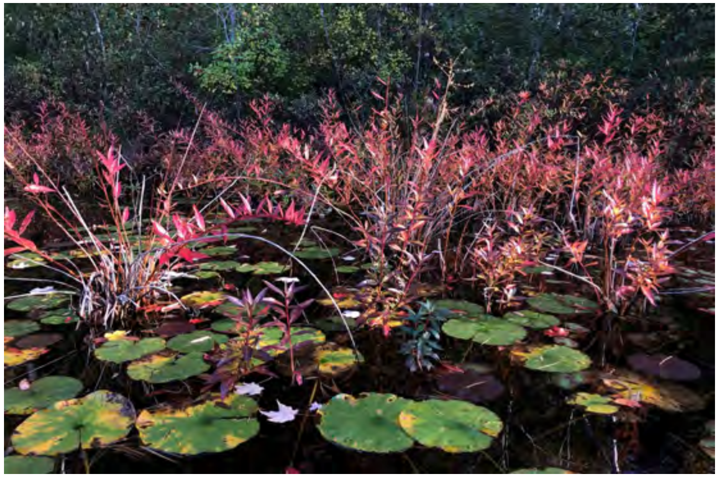

a. Islands and Bays Paddle, Sea Lion Lake (2.14)

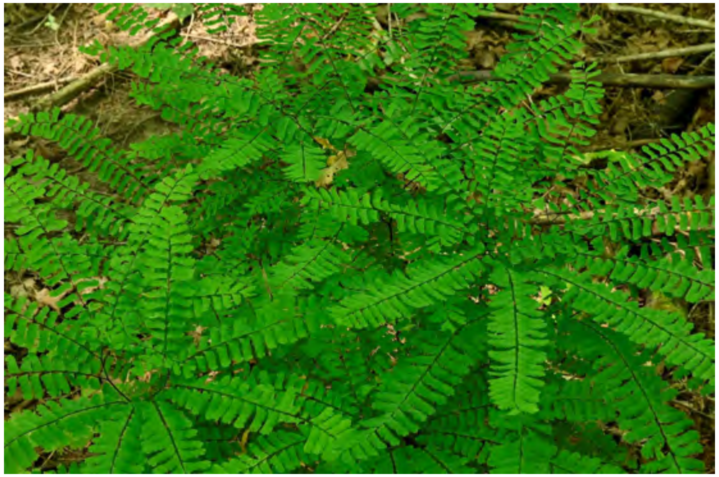

b. Franklin Nature Trail, Hidden Lakes Trail (6.11)

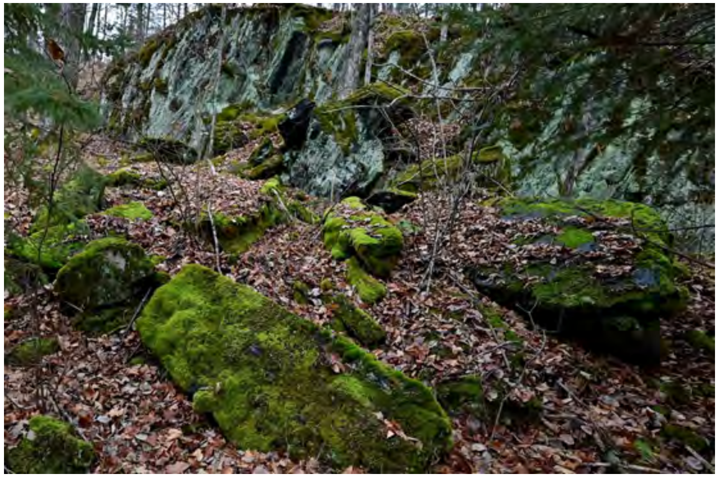

c. Outcrops-River Loop, Pine River Outcrops (1.31)

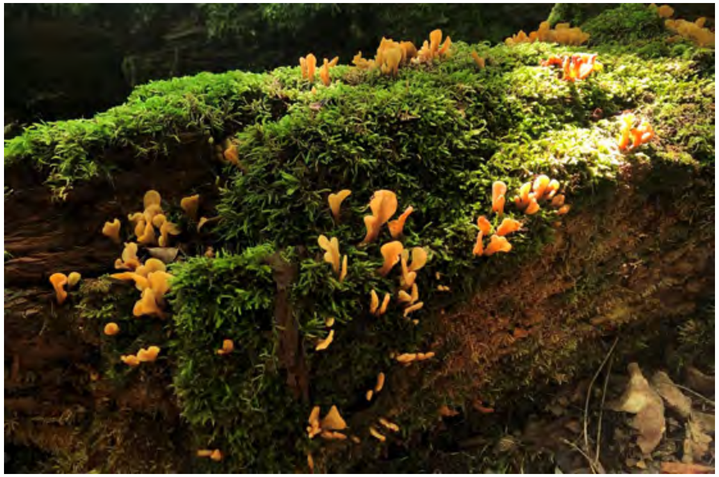

d. Lauterman Lake Trail Loop, Lauterman National Recreation Trail (4.12)

2.31. Spaces– Private

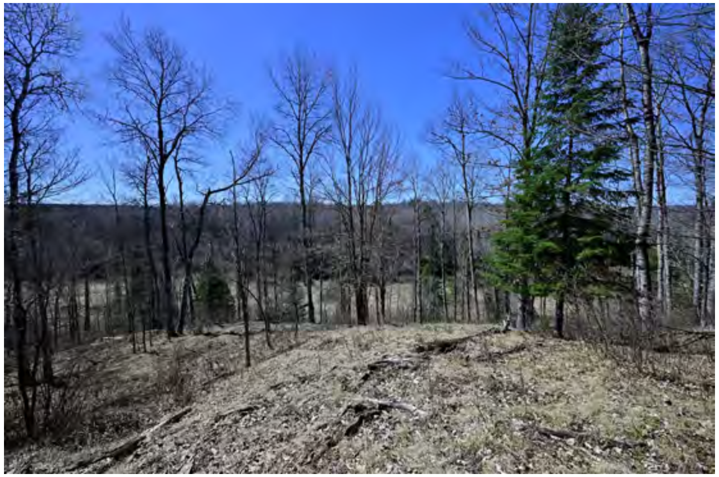

a. North Loop, Rainbow Hunter Walking Trails (3.51)

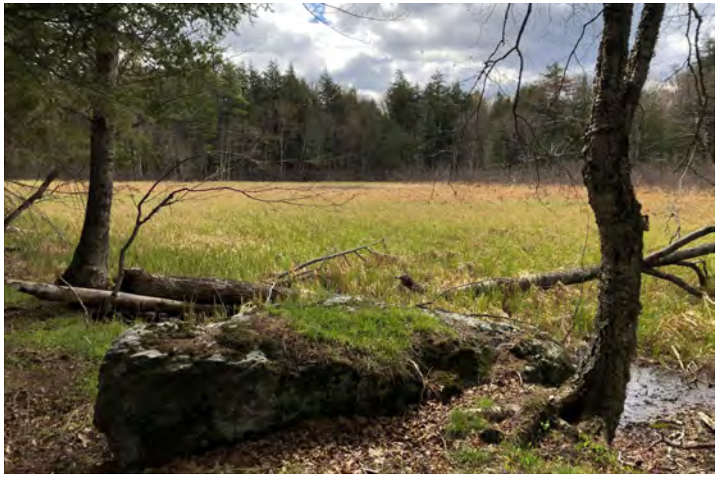

b. Marsh Loop, Fox Maple Woods (3.12)

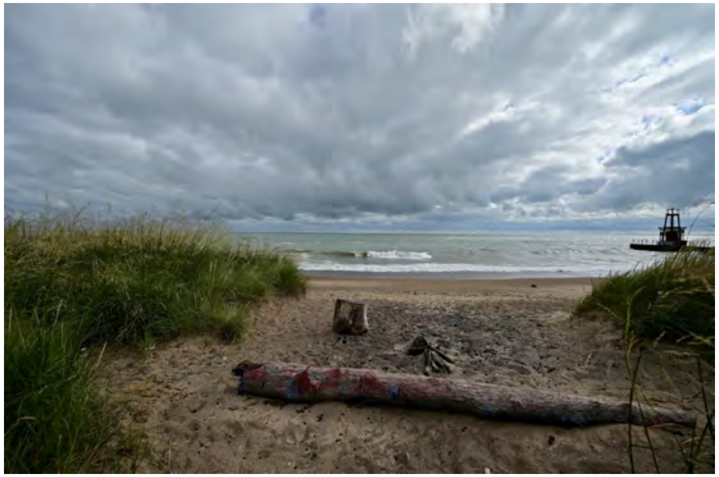

c. Loyola Natural Area-Pier Loop, Loyola-Leone Parks (10.22)

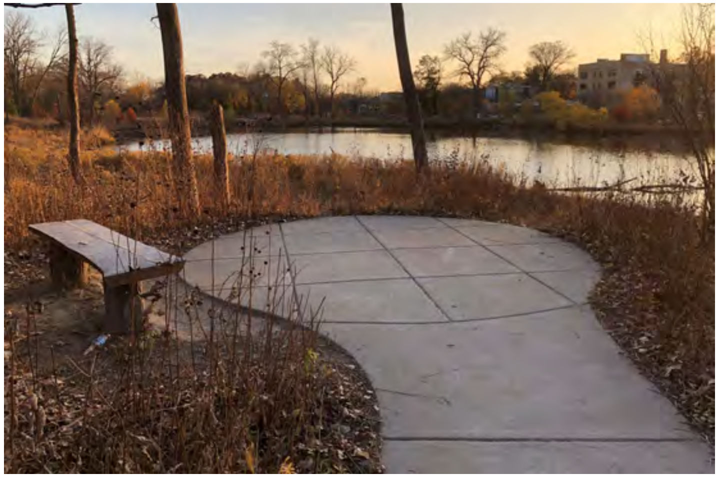

d. Lake Loop, West Ridge Nature Park (9.22)

2.32. Spaces– Group

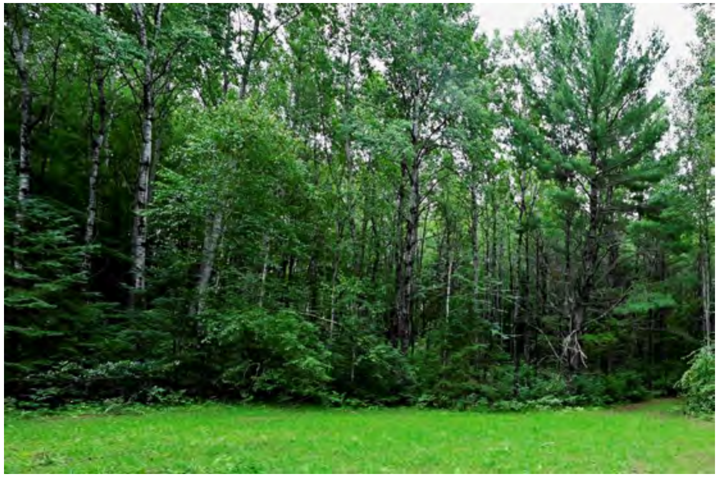

a. North Loop, Lake Emily Recreation Trail (2.21)

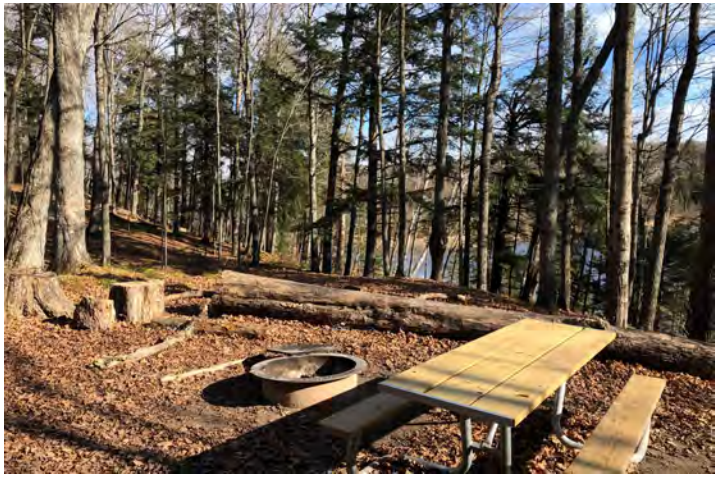

b. Lauterman Lake Loop, Lauterman National Recreation Trail (4.12)

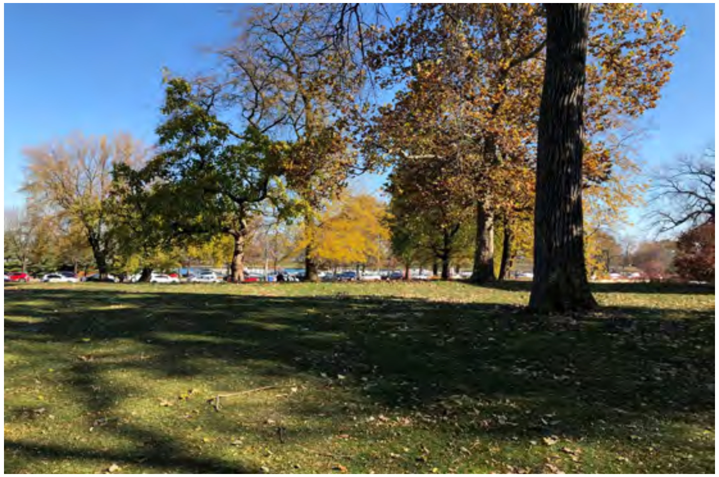

c. Natural Area Loop, North Pond (12.31)

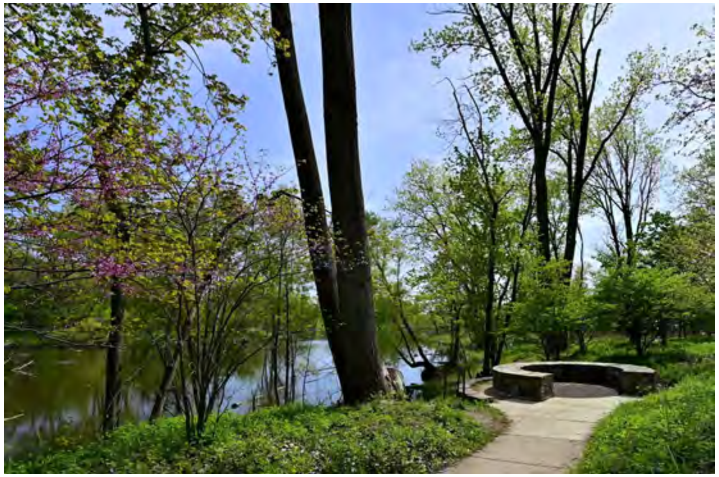

d. Lake Loop, West Ridge Nature Park (9.22)

2.41. Spatial Change– Somme Prairie Grove

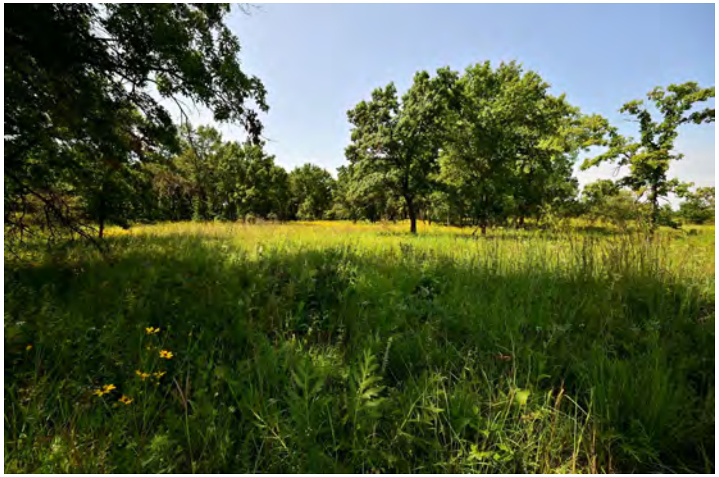

a. Open Savanna, Vestal Grove Savanna Loop (16.21)

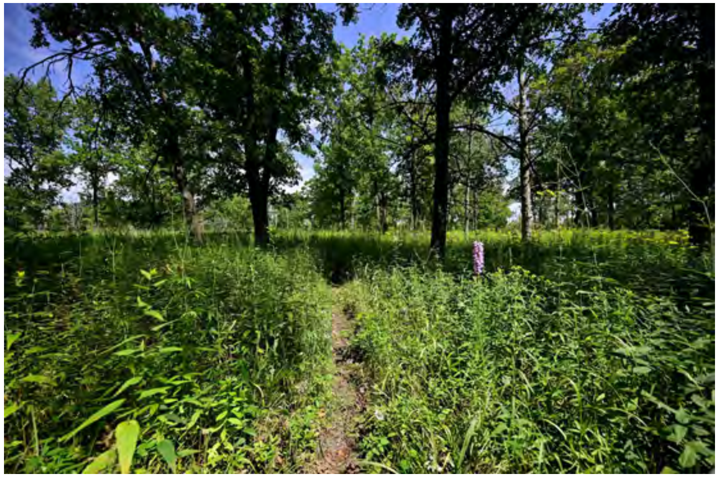

b. Closed Savanna, Vestal Grove Savanna Loop (16.21)

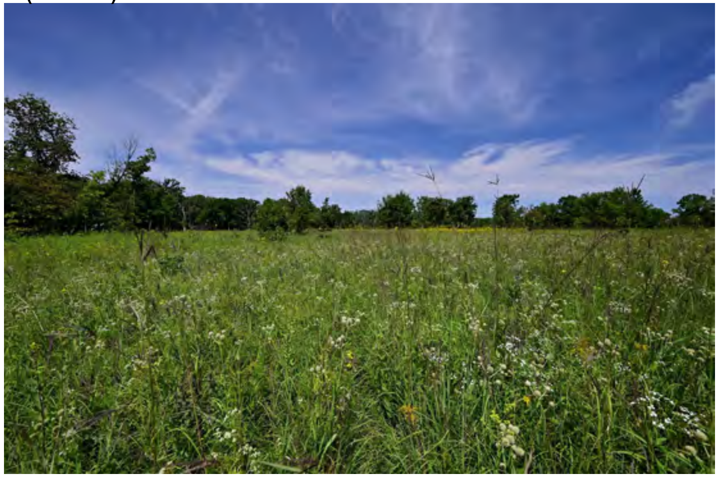

c. Prairie, Vestal Grove Savanna Loop (16.21)

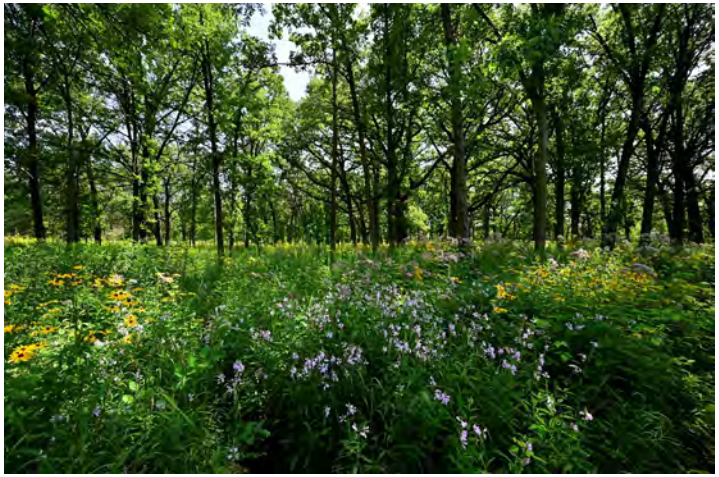

d. Open Woodland, Vestal Grove Savanna Loop (16.21)

2.42. Spatial Change– Lake Anna, Spread Eagle Barrens

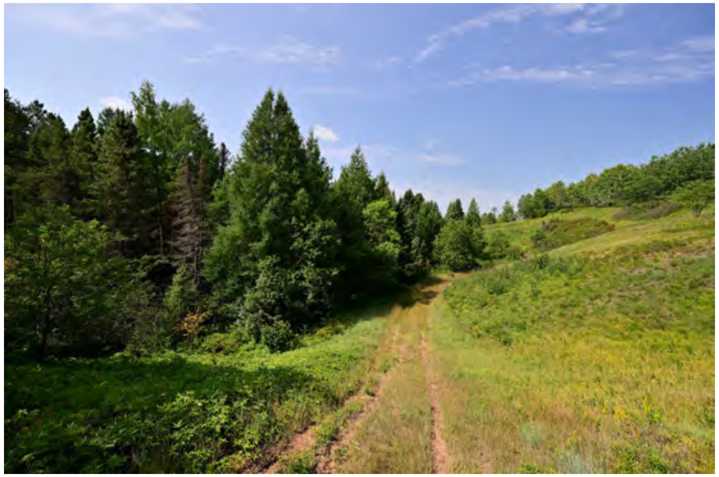

a. Woodland/Barrens Edge, Lake Anna West Loop (7.31)

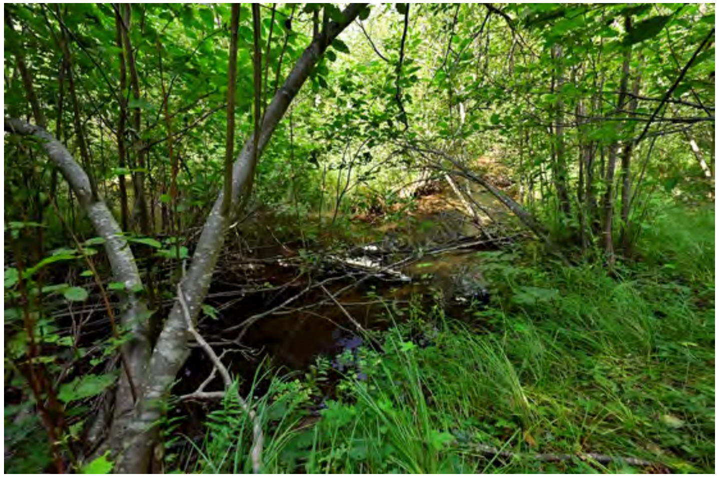

b. LePage Creek, Lake Anna West Loop (7.31)

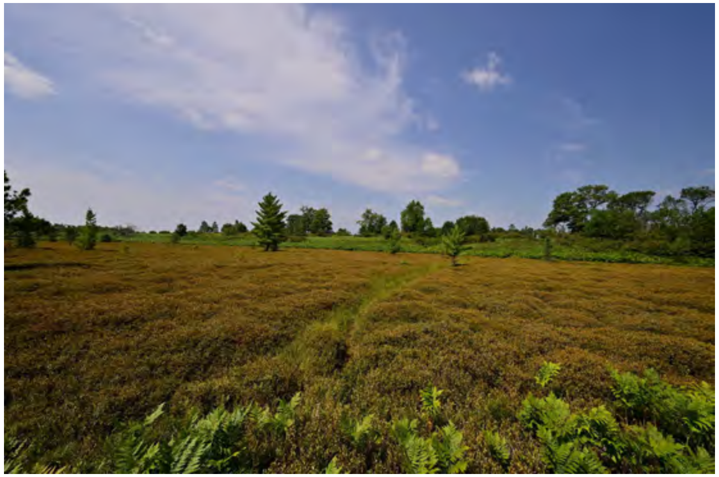

c. Unnamed Bog, Lake Anna Bog Loop (7.34)

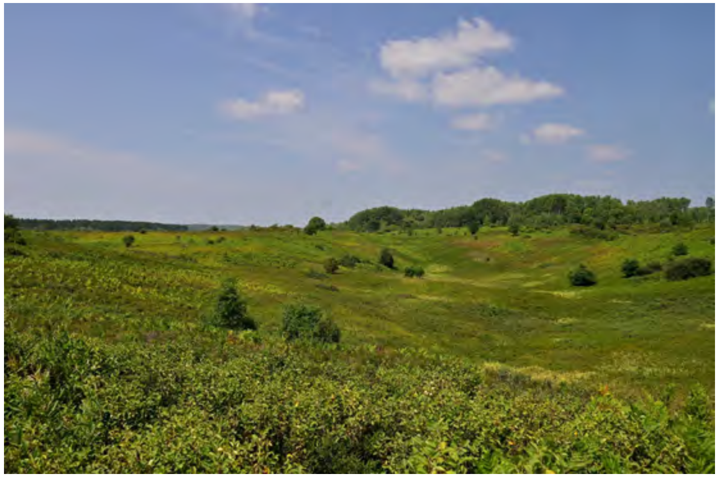

d. Open Barrens, Lake Anna West Loop (7.31)

2.43. Spatial Change– West Ridge Nature Park

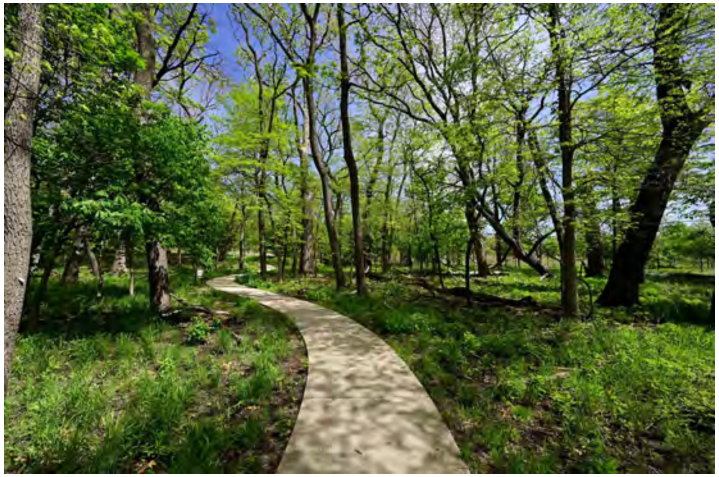

a. Woodland, Woodland-Lake Loop (9.23)

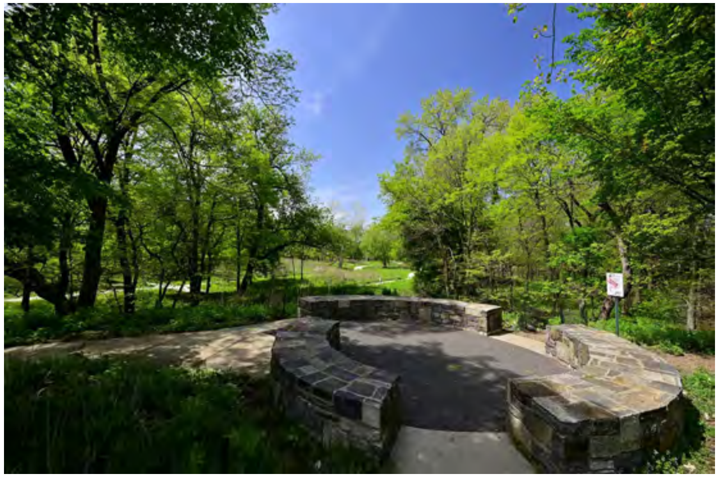

b. Elevated View, Woodland-Lake Loop (9.23)

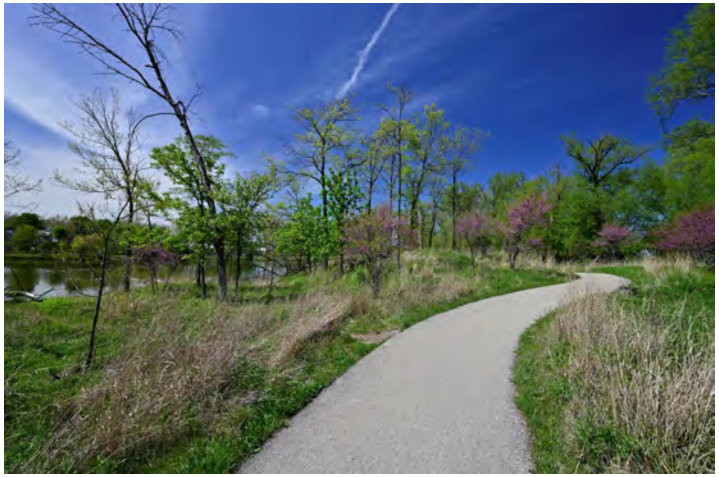

c. Savanna, Woodland-Lake Loop (9.23)

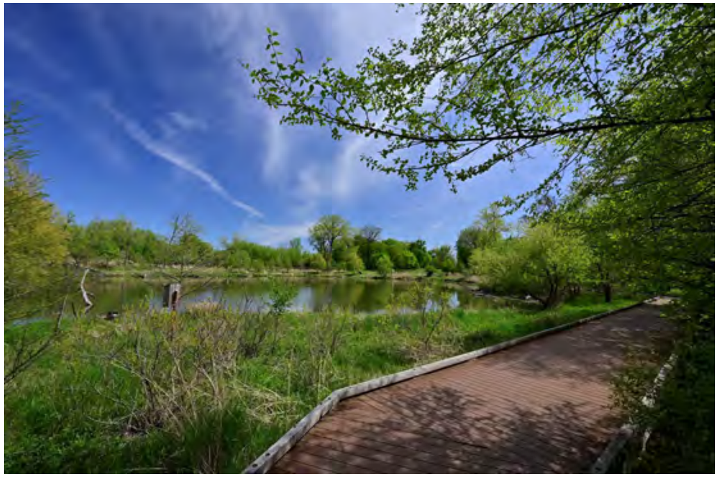

d. Lake View, Woodland-Lake Loop (9.23)

2.44. Spatial Change– Breakwater Falls, Pine River

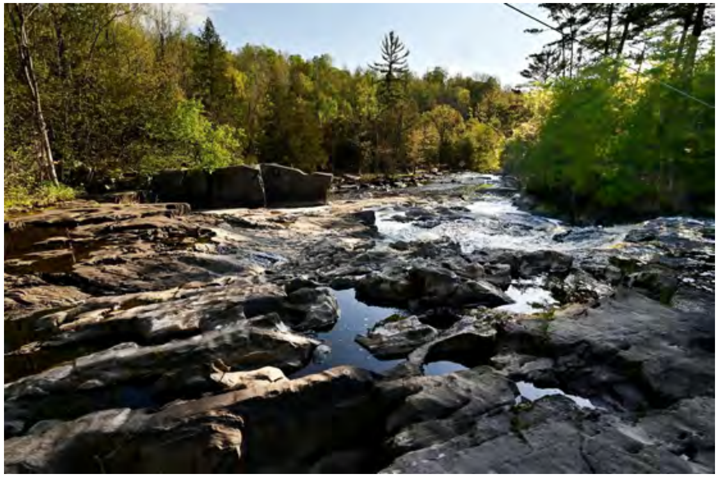

a. 2nd Drop, Breakwater Falls North Bank (1.61)

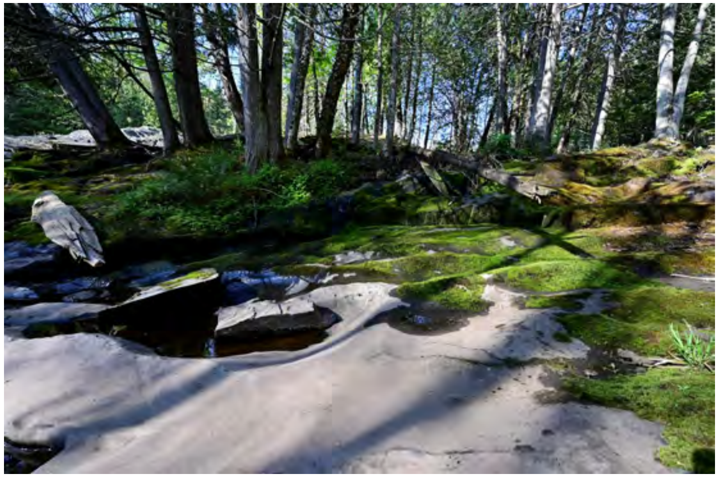

b. Backwater area, Breakwater Falls North Bank (1.61)

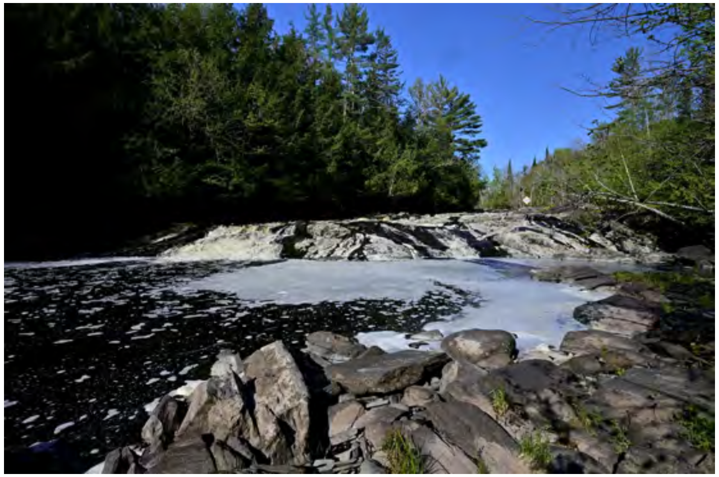

c. 3rd Drop, Breakwater Falls North Bank (1.61)

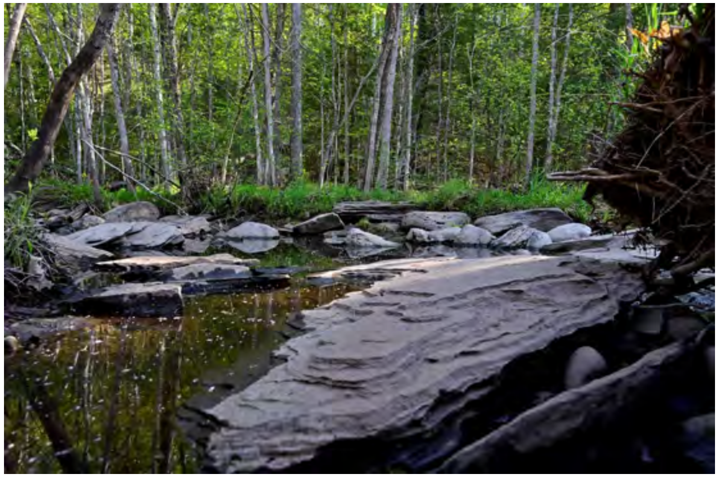

d. Slate Formation, Breakwater Falls North Bank (1.61)

2.45. Temporal Change– Spread Eagle Barrens

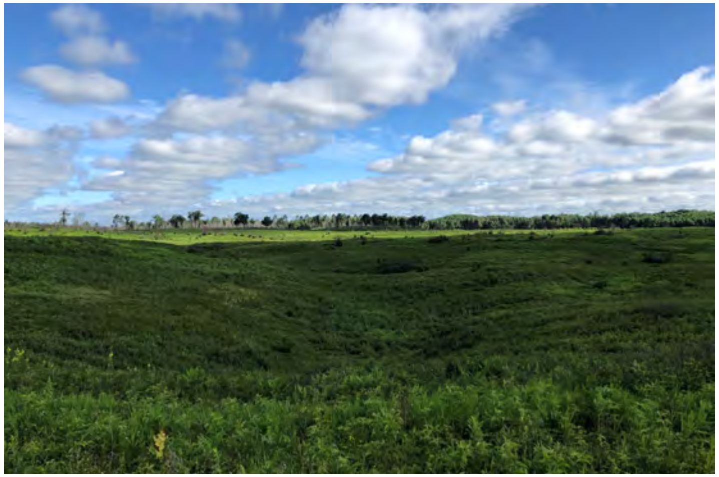

a. July 20, 2020 9:12:45 AM, Fire Lane Rd. Loop (7.12)

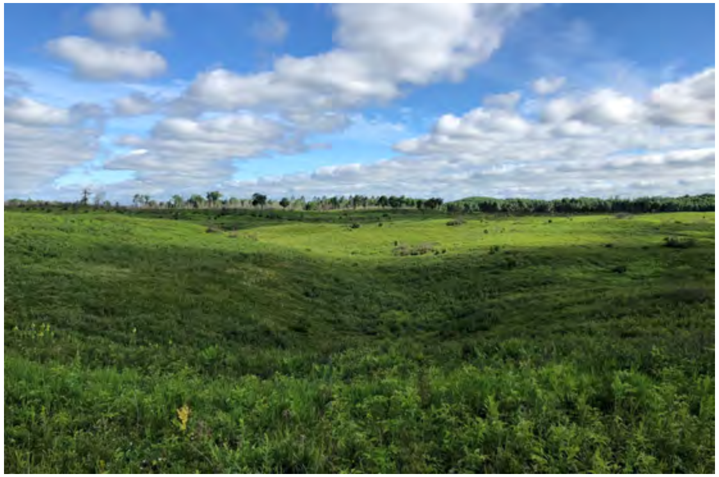

b. July 20, 2020 9:13:00 AM, Fire Lane Rd. Loop (7.12)

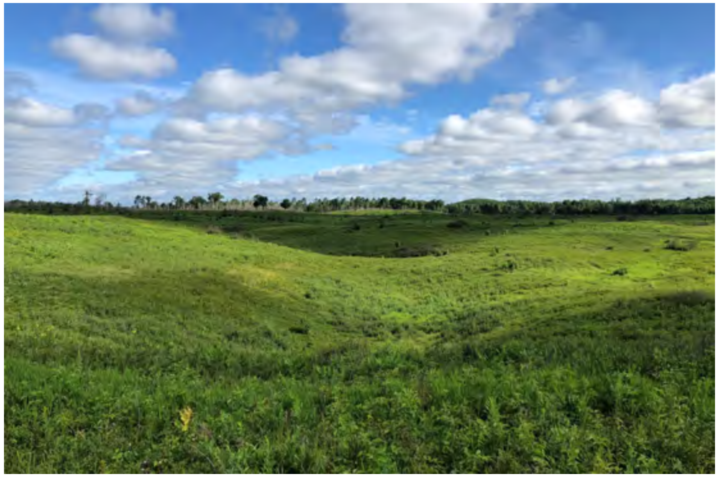

c. July 20, 2020 9:13:15 AM Fire Lane Rd. Loop (7.12)

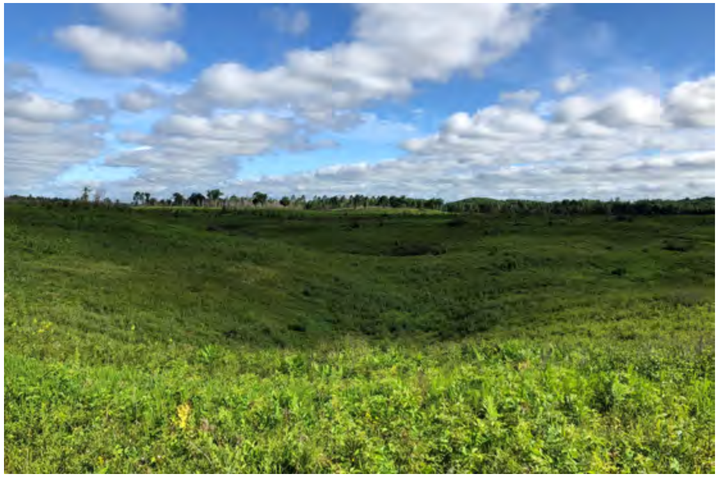

d. July 20, 2020 9:13:30 AM, Fire Lane Rd. Loop (7.12)

2.46. Temporal Change– Spread Eagle Barrens

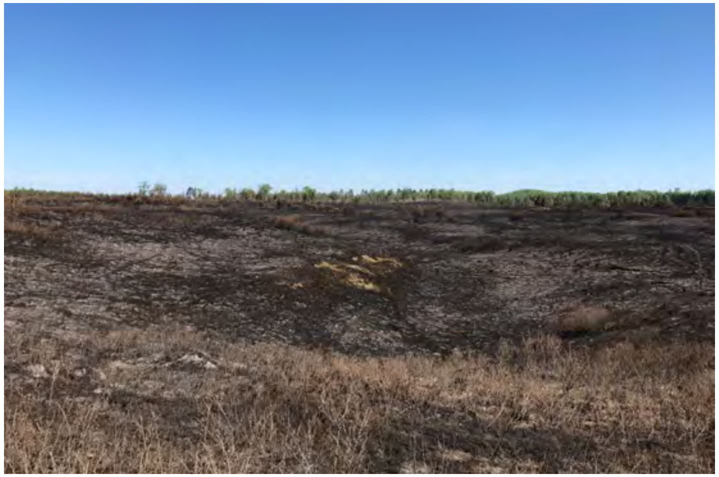

a. June 8, 2019, Fire Lane Rd. Loop (7.12)

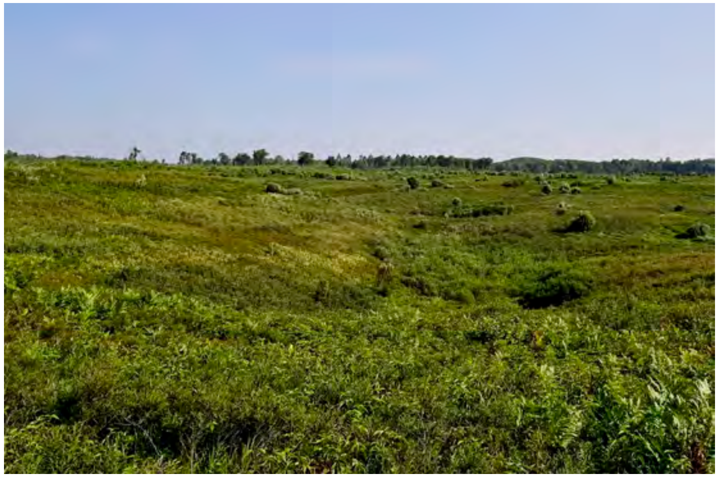

b. July 14, 2019, Fire Lane Rd. Loop (7.12)

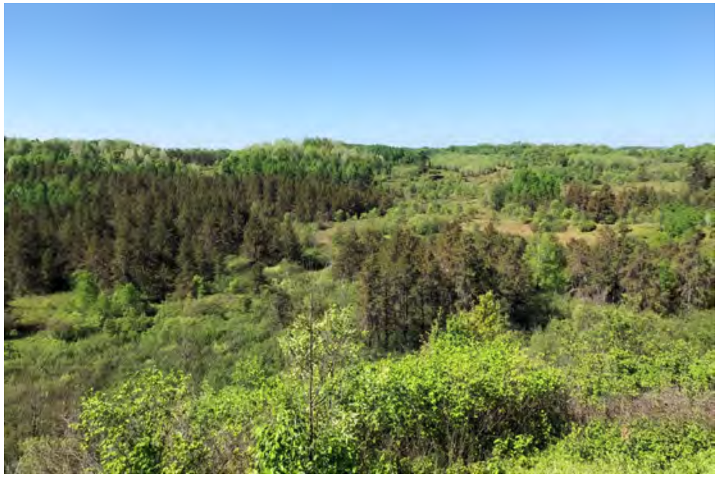

c. June 8, 2019, LePage Creek Overlook (7.35)

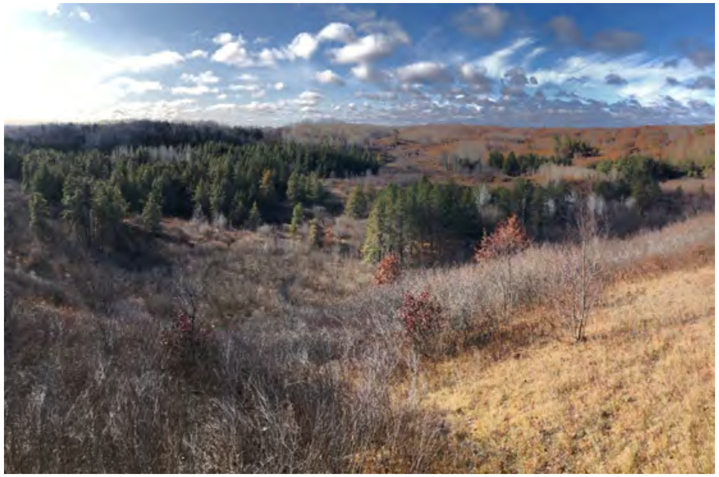

d. November 7, 2019, LePage Creek Overlook (7.35)

3. Natural Features

3.1. Trees- Trees are the main event in many forest therapy walks, and this sequence shows some of the many ways they can be experienced and appreciated. While very few old growth forests remain within the two study areas, several of the sites I identified included impressive patches of mature and old growth forests accessible along their trails (3.11, 3.12). These trees as well as those that occurred singly or in groups along other trails (3.13, 3.14) can instill a sense of awe in the beholder, creating an appreciation for their existence and a larger connection with the natural world. Contact with trees—feeling the texture of their bark (3.15, 3.16), gazing into their canopies (3.17), and exploring their distinctive forms (3.18, 3.19) and sculptural qualities (3.110, 3.111) can be deeply rewarding.

3.2. Water- Water is another key natural feature that can enhance forest therapy experiences, and both study areas were richly endowed with surface waters of various types. Rivers and lakes of different sizes (3.21–3.24; see also Width above) offer different experiences and uses, from small creeks one can jump across (3.22c) to the vastness of Lake Michigan (3.24d). Wetlands were abundant in the Northwoods and included bogs and forested wetlands and lake edges (3.25) and while less frequent in Chicago also included wooded areas, ephemerals ponds, and a rain garden (3.26). The Pine River flowing through the Northwoods study area had several waterfalls along its course and tributaries (3.27), and while the bigger falls provided grandeur and excitement, smaller falls were more approachable and interactive, including a few ideal for soaking during low flow periods on hot summer days. In Chicago, waterfalls were mostly artificial but can still be exquisite (3.28a–c), while brisk days on the shores of Lake Michigan can bring large waves (3.28d). Finally, winter can be a wonderful way to experience the many forms of frozen water (3.29).

3.3. Wildlife- Wildlife can be an elusive natural feature to experience directly, but trails that pass through rich wildlife habitat offer better chances to come into contact with wildlife big and small (3.31). And when not present, good habitat offers more reliable opportunities to see nests and other wildlife structures (3.32) as well as behavioral traces (3.33).

3.4. Ground Flora, Rock, Moss, and Fungi- A host of smaller natural features graced the forest floor, trees, and open landscapes of the two study areas and offered myriad opportunities to experience nature close-up (see also Views, above). Forest and grassland flora (3.41–3.44) provides vibrant color, distinctive textures, and sometimes edible feasts during seasonal blooming periods, while aquatic flora (3.45) adds interest to the paddling or shoreline trail experience. Rocks (3.46), from 100’ (30 m) rock cliffs (3.46a) to hand held chunks of granite (3.46d), were unique features of the Northwoods and connect visitors with Precambrian events more than 2 billion years ago. And mosses, lichen, and fungi (3.47, 3.48) offer sensuous textures, vibrant colors, and micro-landscapes that spur the imagination.

3.11. Trees– Old Growth Forests Northwoods

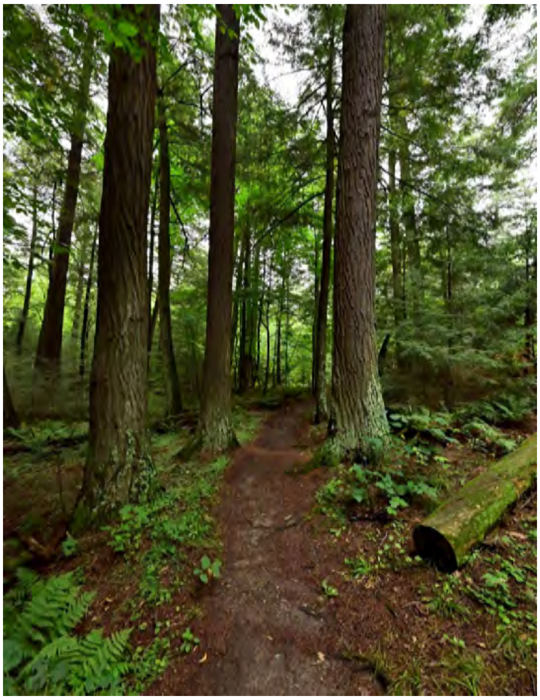

a. Franklin Nature Trail, Hidden Lakes Trail (6.11)

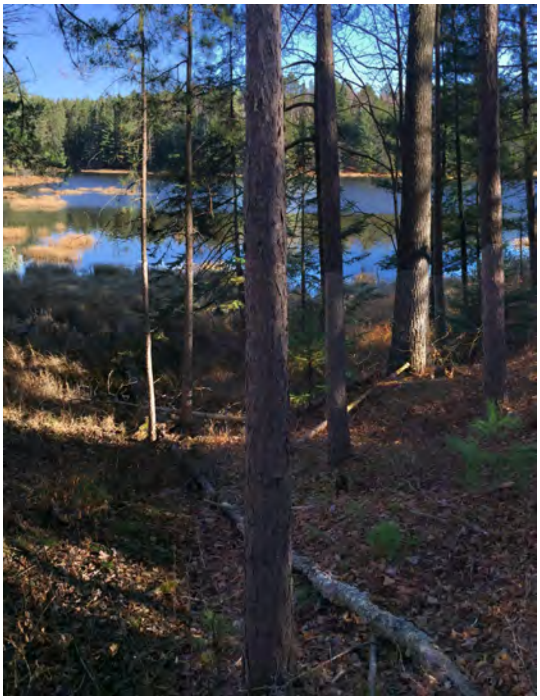

b. Whisker Lake Trail portion, Whisker Lake Wilderness (3.41)

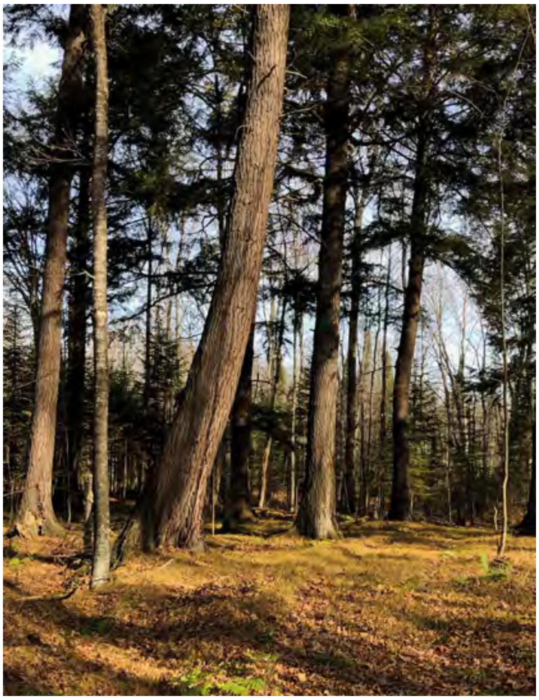

c. Beginner's Trail Loop, Lauterman National Recreation Trail (4.11)

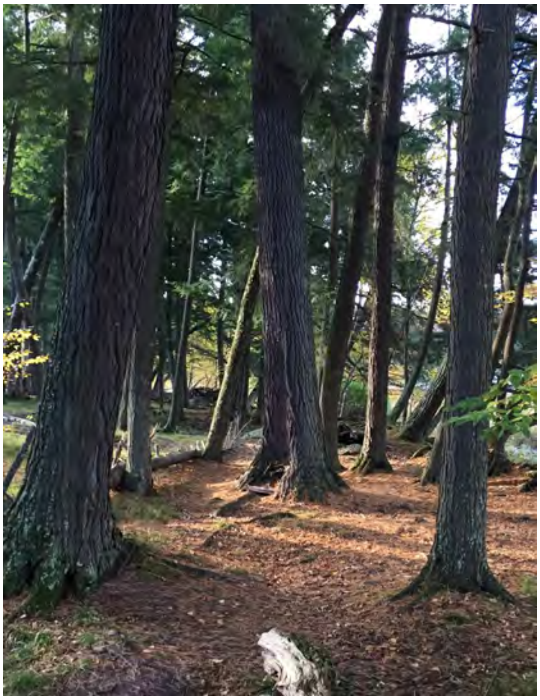

d. Lakeshore Trail Loop, Lost Lake Recreation Area (4.21)

3.12. Trees– Old Growth Forests Chicago

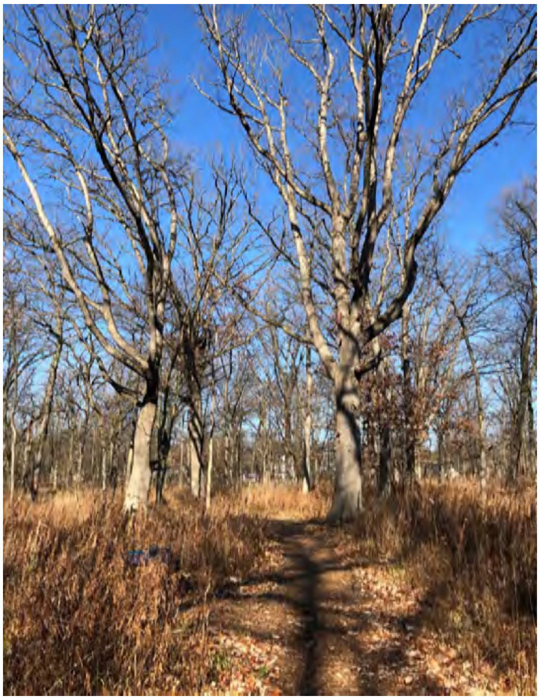

a. McDonald Woods South Loop, Chicago Botanic Garden (15.21)

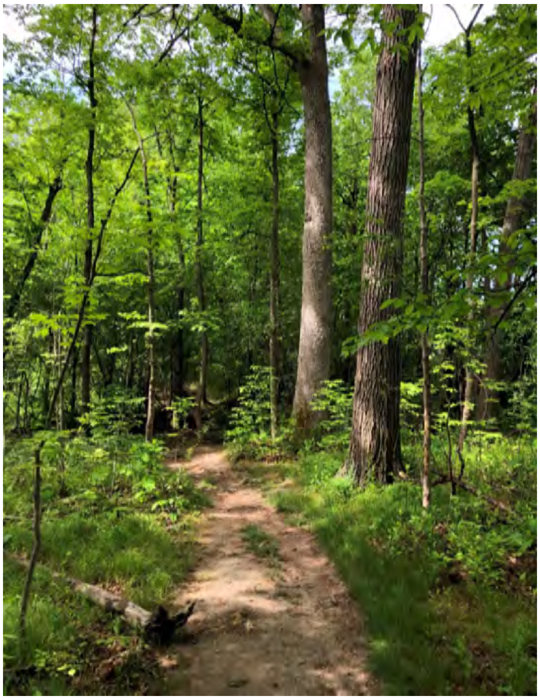

b. Full Loop, Harms Woods Preserves (14.14)

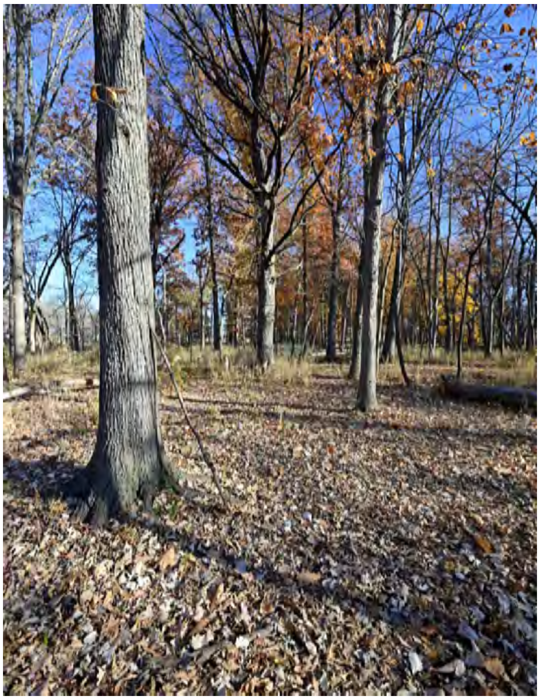

c. Flatwoods Meander, Bunker North Flatwoods (13.31)

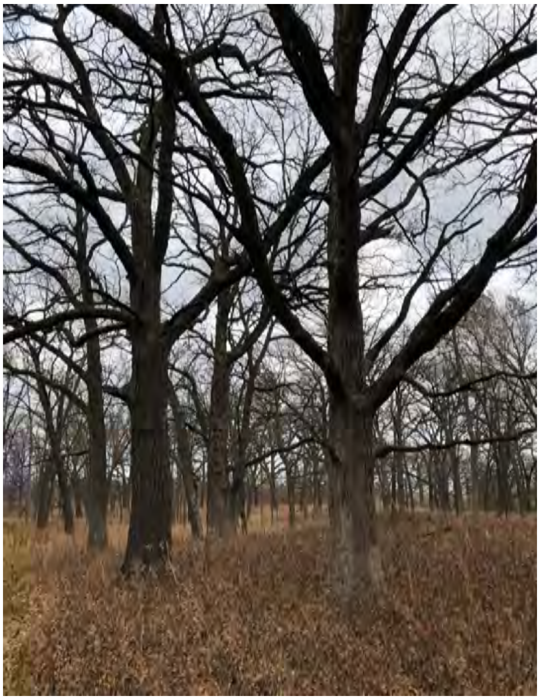

d. Vestal Grove Savanna Loop, Somme Prairie Grove (16.21)

3.13. Trees– Big Single & Group Trees Northwoods

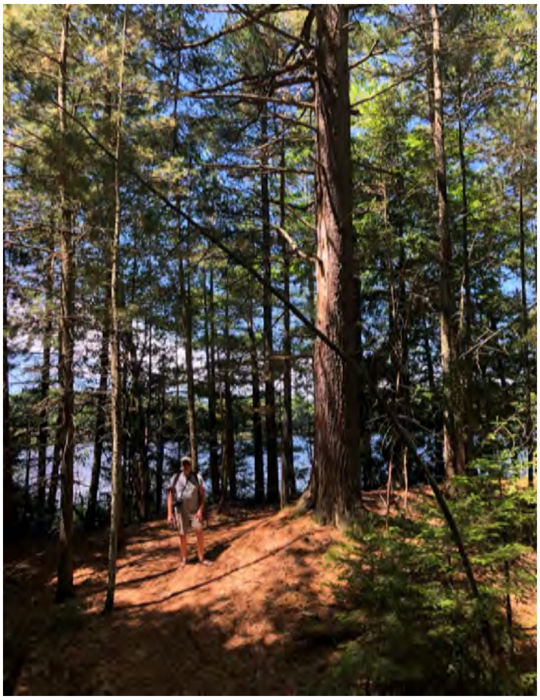

a. White Pine, McKinley Lake–Three Johns Lake segment, Hidden Lakes Trail (6.13)

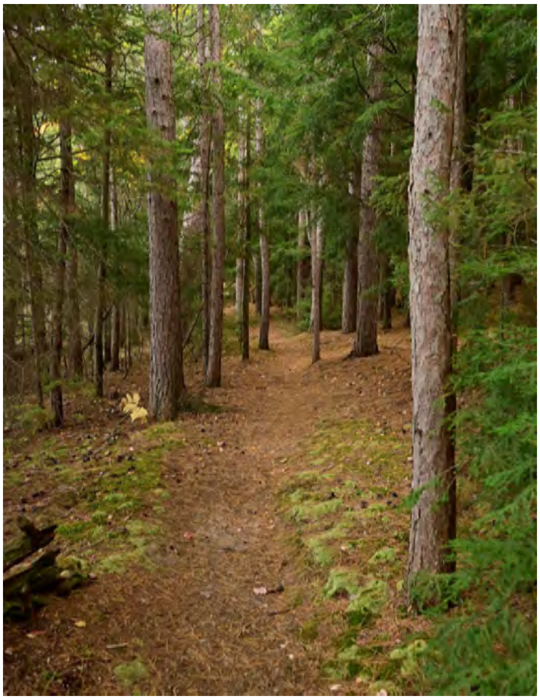

b. Red Pines, White Deer Lake Trail, Hidden Lakes Trail (6.16)

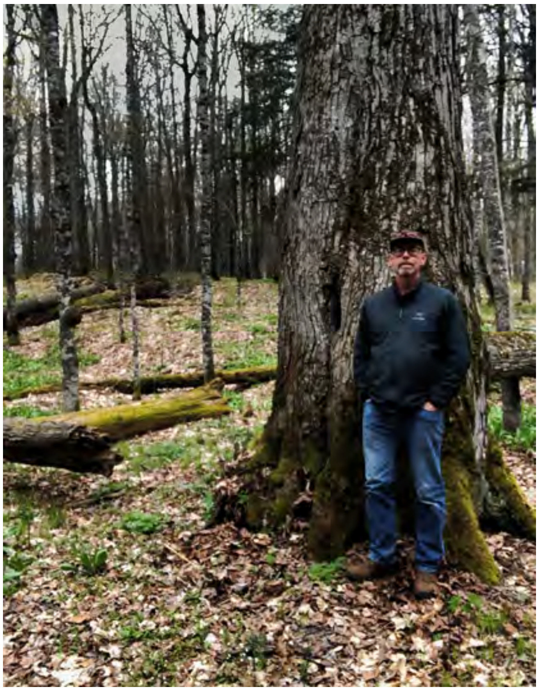

c. Sugar Maple, Main Trail, Fox Maple Woods (3.11)

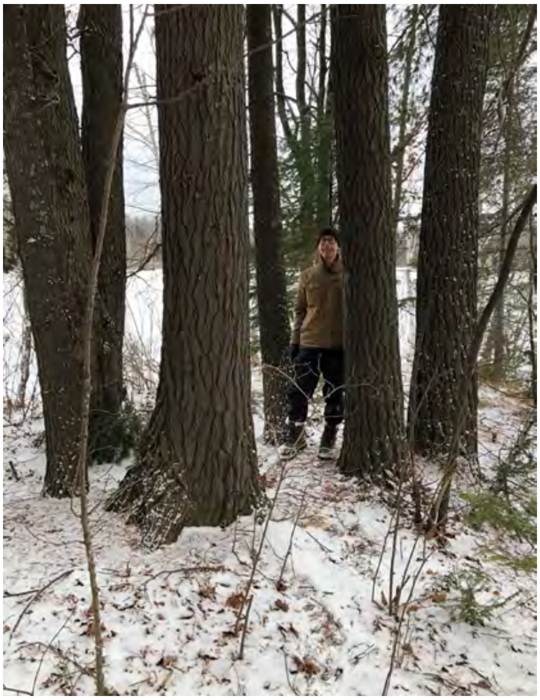

d. White Pines, Loop Trail North Portion, Perch Lake (3.21)

3.14. Trees– Big Single & Group Trees Chicago

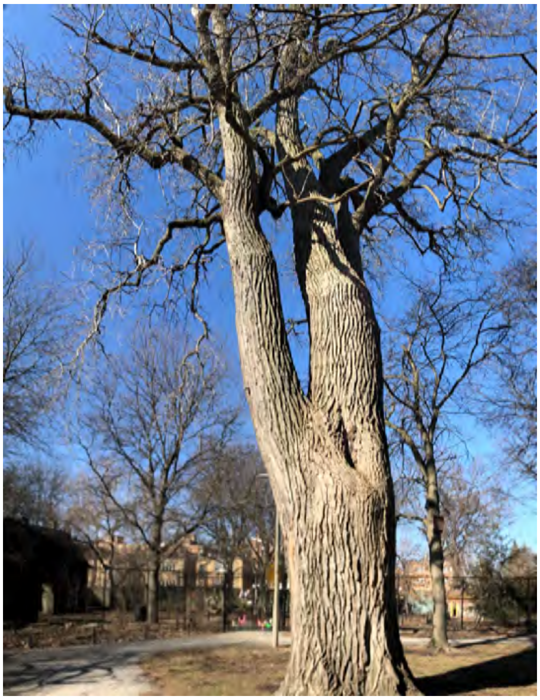

a. Cottonwood, Park Loop, Indian Boundary Park (9.32)

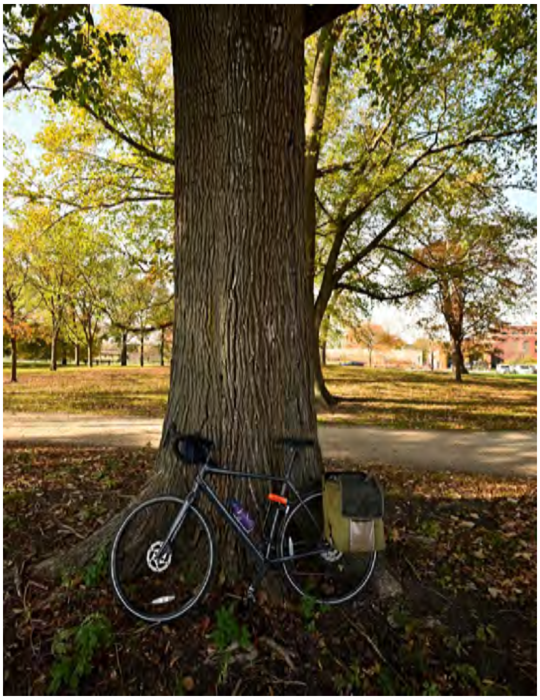

b. Basswood, Fullerton Area Bike Loop Multi-Site (12.51)

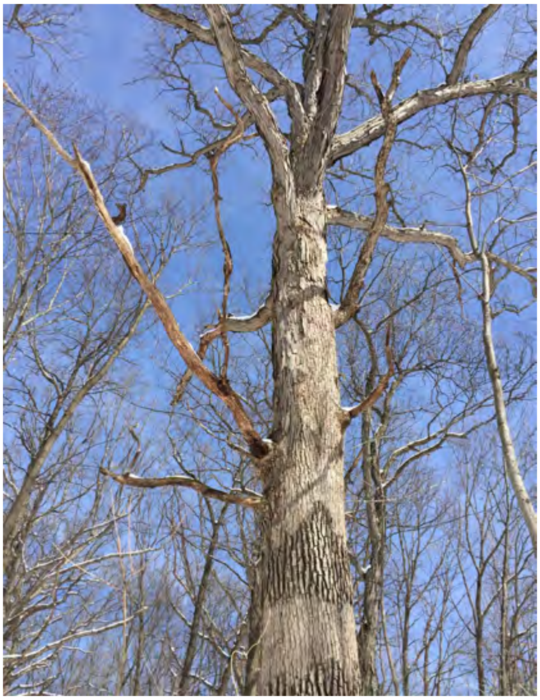

c. White Oak, Full Loop, Harms Woods Preserves (14.14)

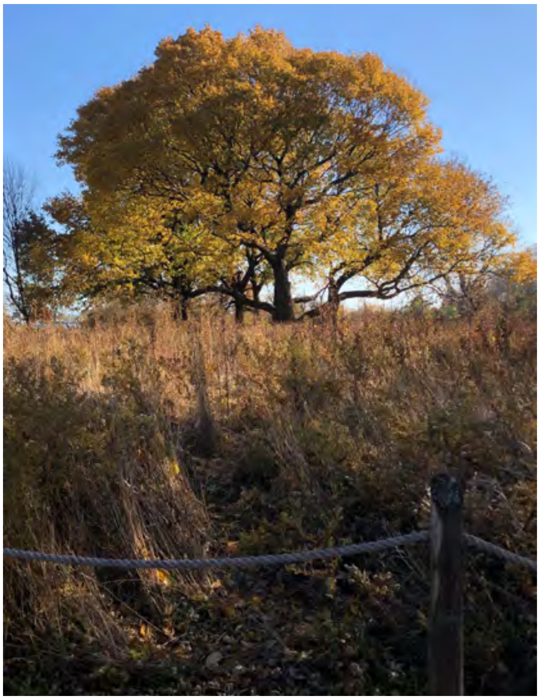

d. Burr Oak, Savanna Loop, Marovitz Savanna (11.21)

3.15. Trees– Bark Northwoods

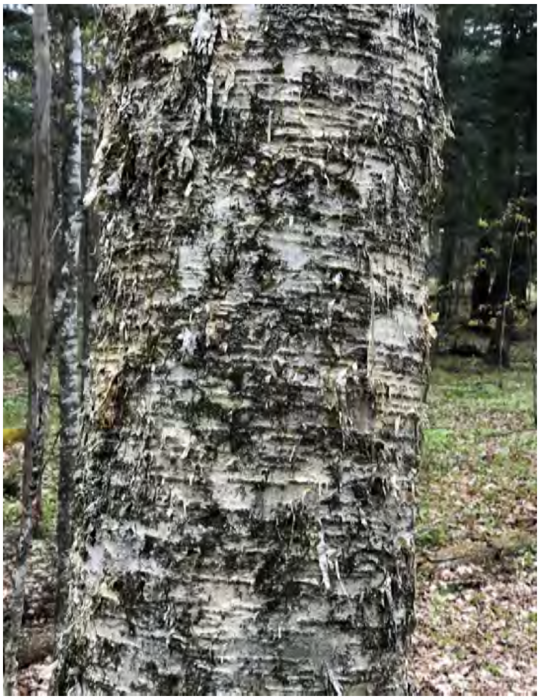

a. Yellow Birch, Upland Loop, Fox Maple Woods (3.13)

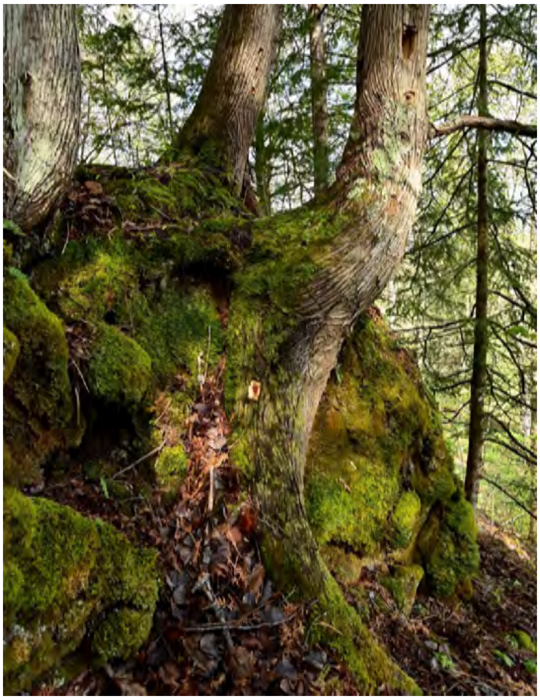

b. Northern White Cedar, Cliffs-River Loop, Brule River Cliffs (5.12)

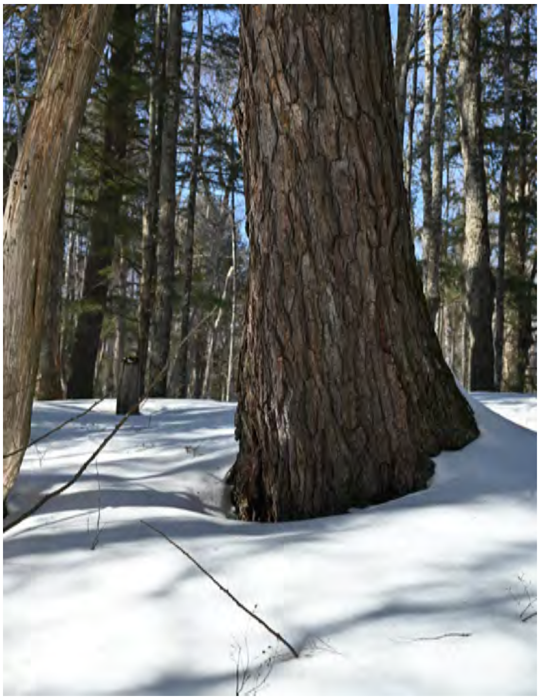

c. White Pine, Marsh Loop, Fox Maple Woods (3.12)

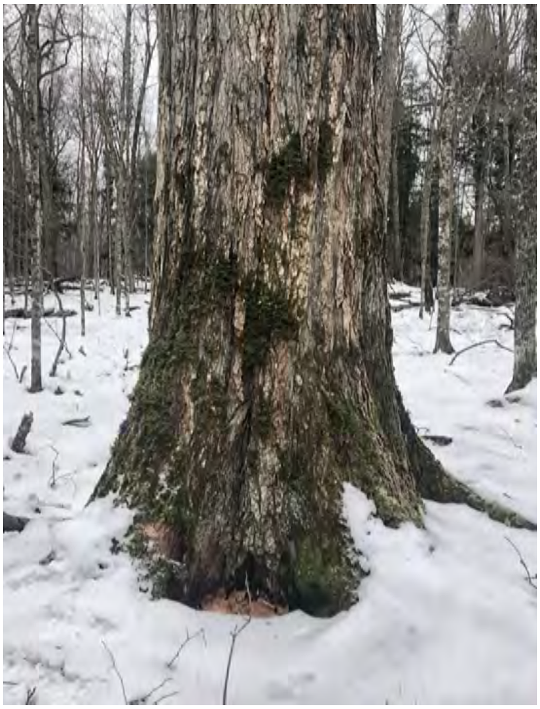

d. Sugar Maple, Main Loop, Fox Maple Woods (3.12)

3.16. Trees– Bark Chicago

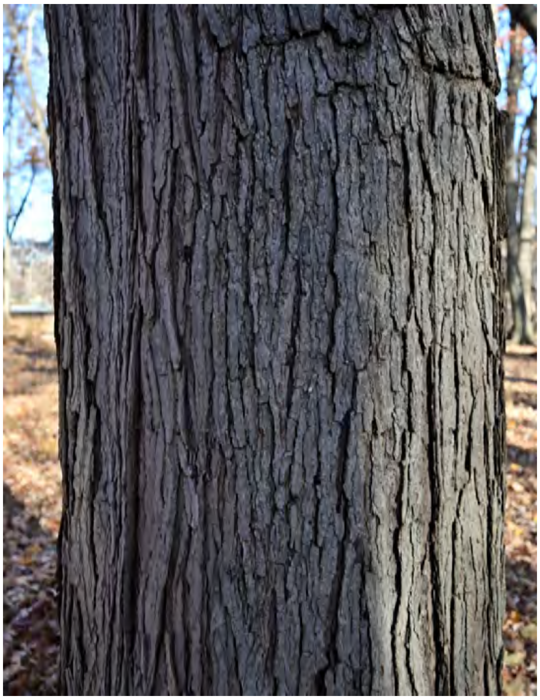

a. White Oak, Flatwoods meander, Bunker North Flatwoods (13.31)

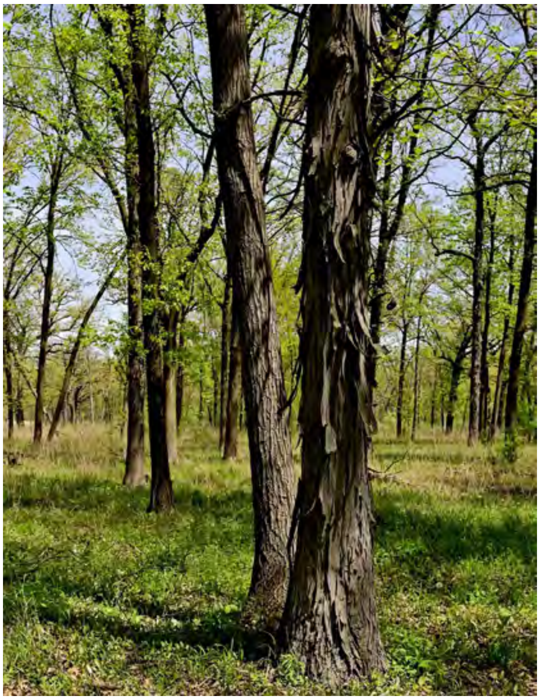

b. Shagbark Hickory, West Outer Loop, Somme Woods (16.32)

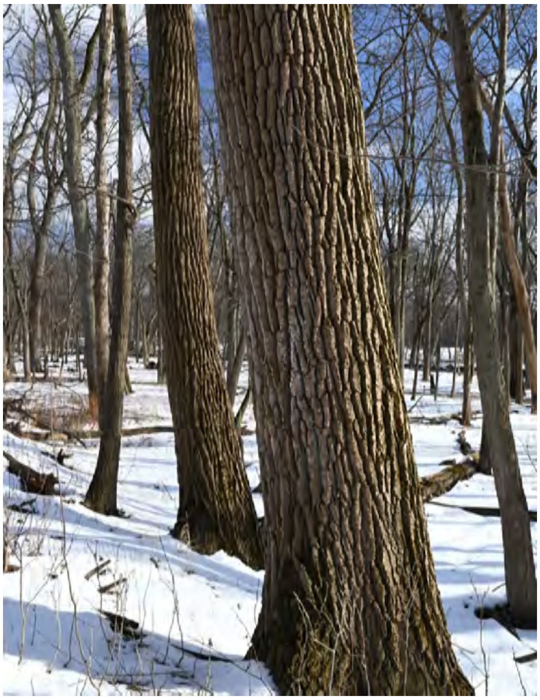

c. Cottonwood, Woodland-River East Loop, Harms Woods Preserves (14.12)

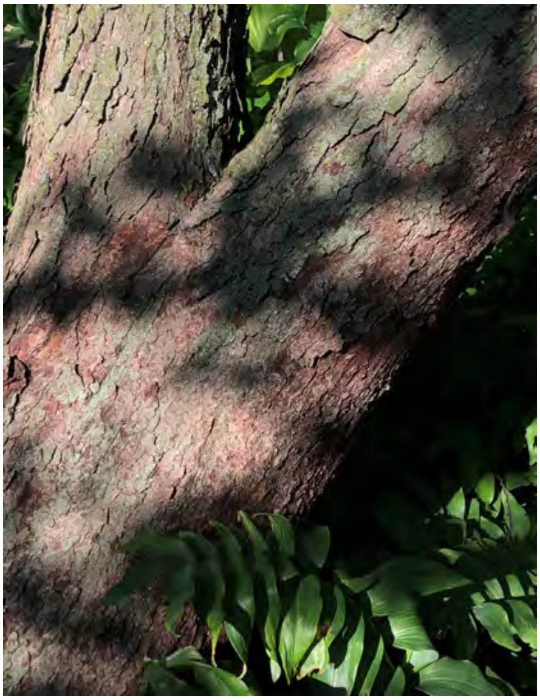

d. Redbud, Lily Pool Loop, Alfred Caldwell Lily Pool (12.11)

3.17. Trees– Canopy Views

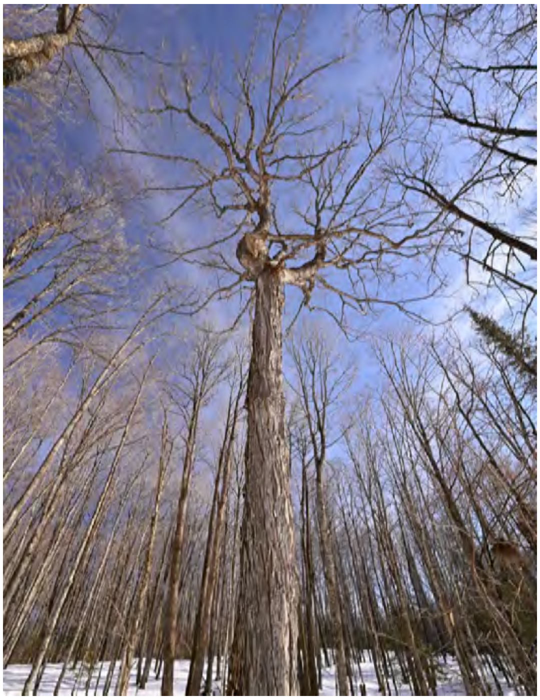

a. Sugar Maples in Mixed Hardwood Canopy, Loop Trail North Portion, Perch Lake (3.21)

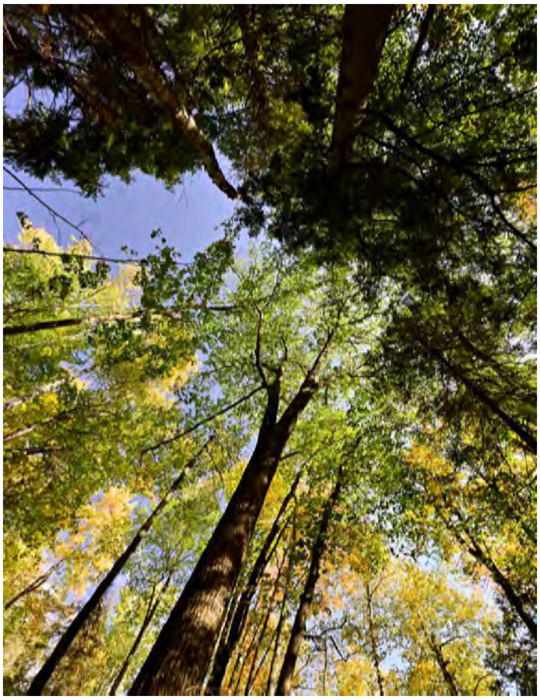

b. Mixed Hardwood Forest, Luna Lake Trail, Hidden Lakes Trail (6.15)

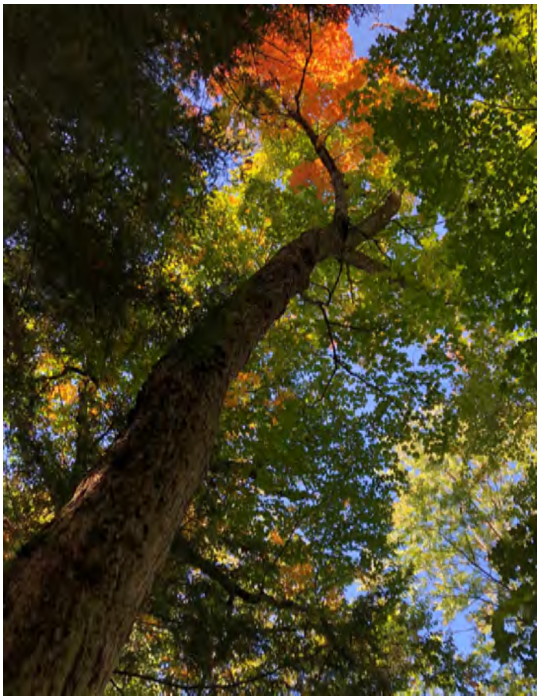

c. Hemlock Hardwood Forest, Main Loop, Fox Maple Woods (3.11)

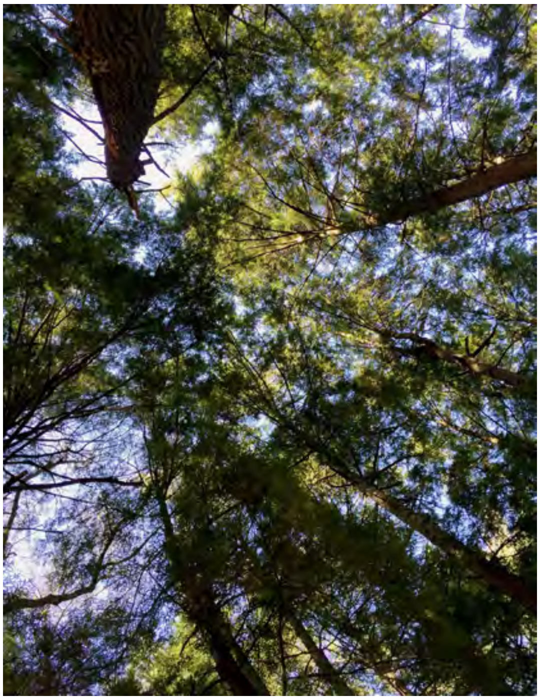

d. Hemlock Forest, West Lakeshore Trail, Lost Lake Recreation Area (4.25)

3.18. Trees– Distinctive Shapes

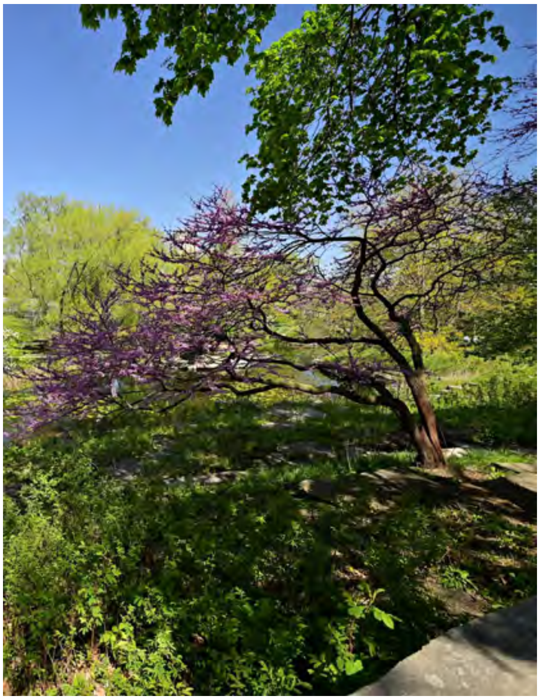

a. Redbud, Lily Pool Loop, Alfred Caldwell Lilly Pool (12.11)

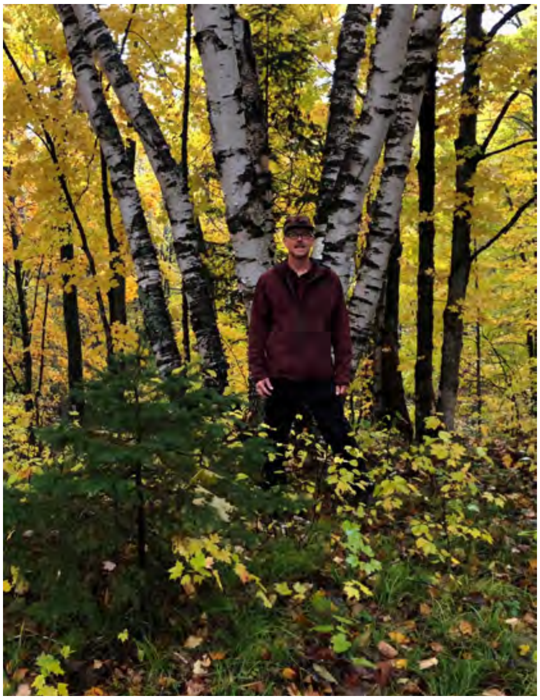

b. White Birch, Lakeshore-Ridge Trail Loop, Lost Lake Recreation Area (4.23)

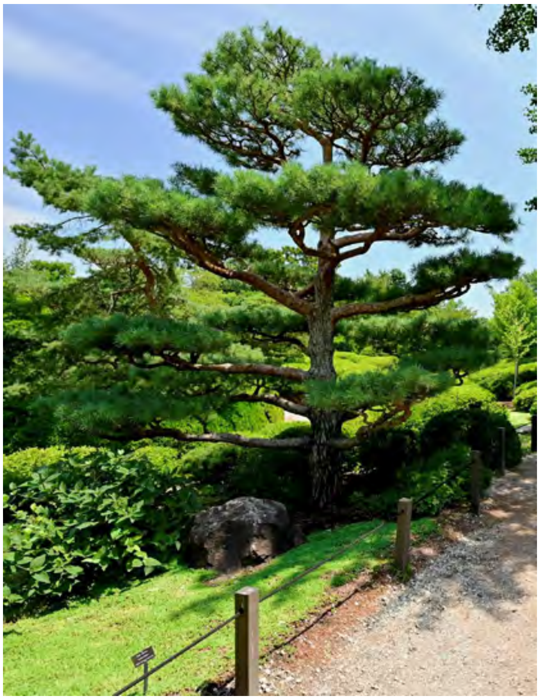

c. Pruned Scots Pine, Japanese Garden, Chicago Botanic Garden (15.24)

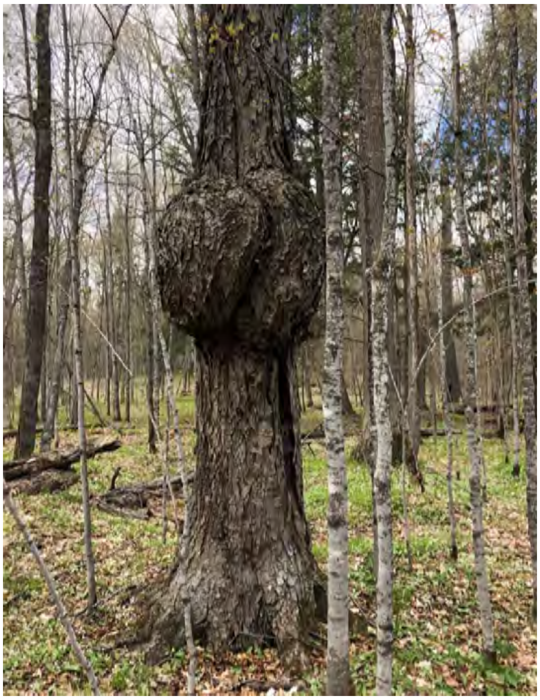

d. Sugar Maple Burl, Marsh Loop, Fox Maple Woods (3.12)

3.19. Trees– Shoreline Trees

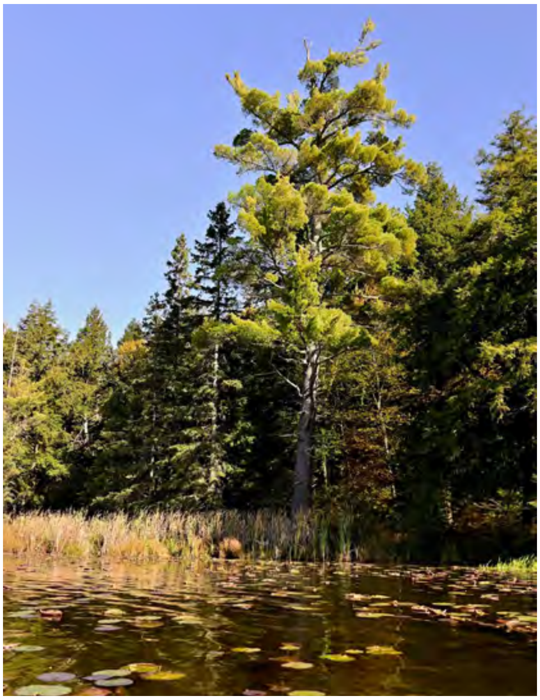

a. Supercanopy White Pine, Savage Lake Paddle, Savage-Robago Wild Lakes Complex (1.82)

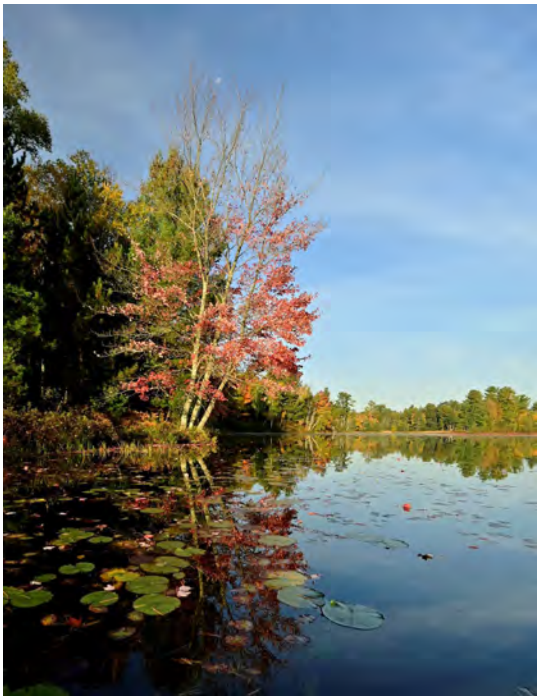

b. Sugar Maples, Islands and Bays Paddle, Sea Lion Lake (2.14)

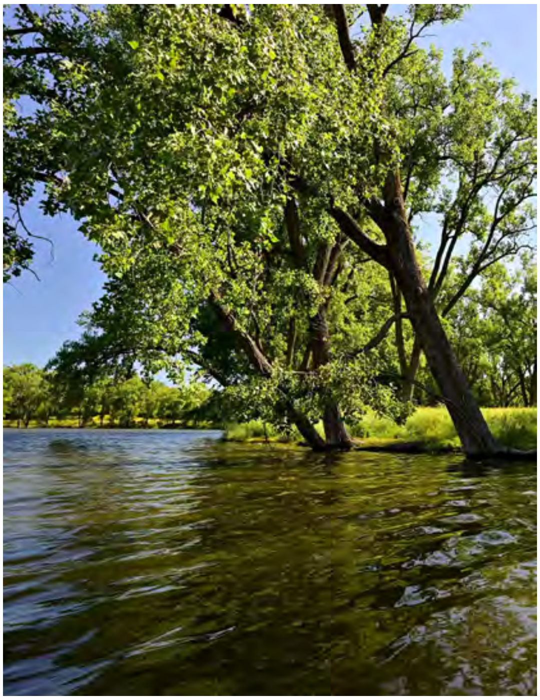

c. Cottonwoods, Lagoons 4-5 Loop, Skokie Lagoons Forest Preserve (15.15)

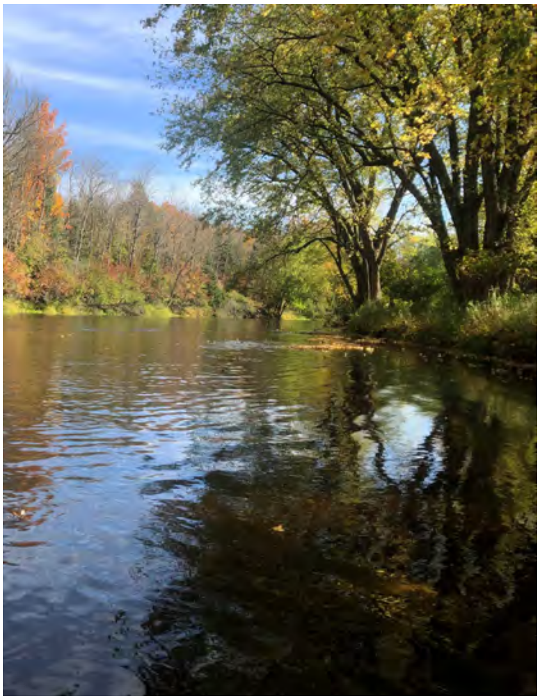

d. Silver Maples, Pine River Flats Paddle, Pine River Flats (1.21)

3.110. Trees– Dead and Downed

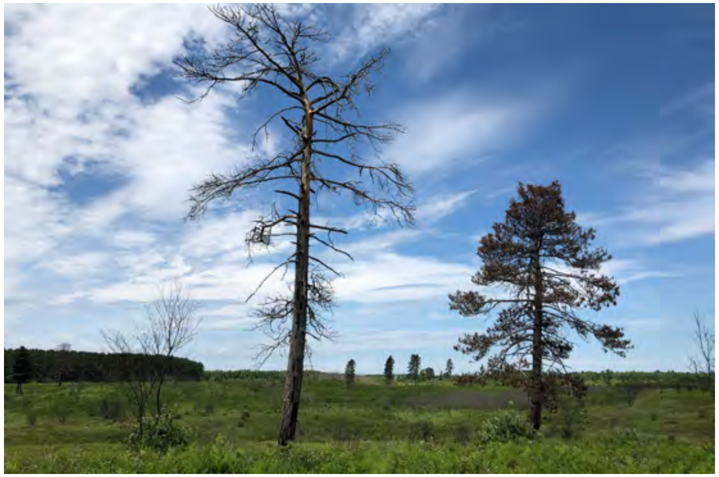

a. Fire Lane Rd. Loop, Spread Eagle Barrens (7.12)

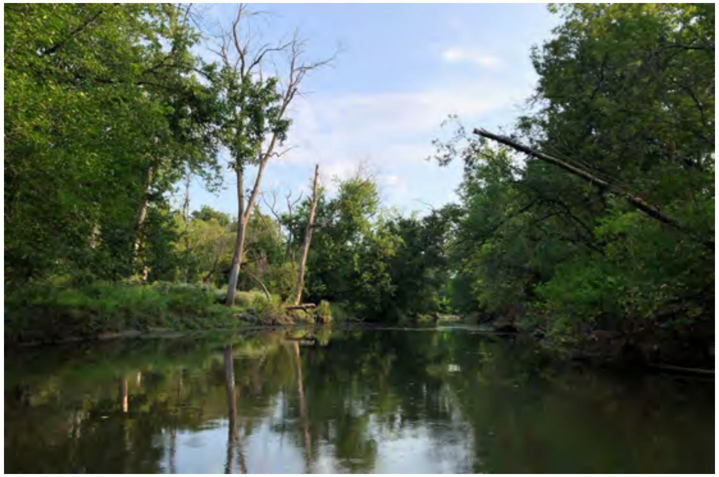

b. Chicago River Paddle, Caldwell Preserves (13.41)

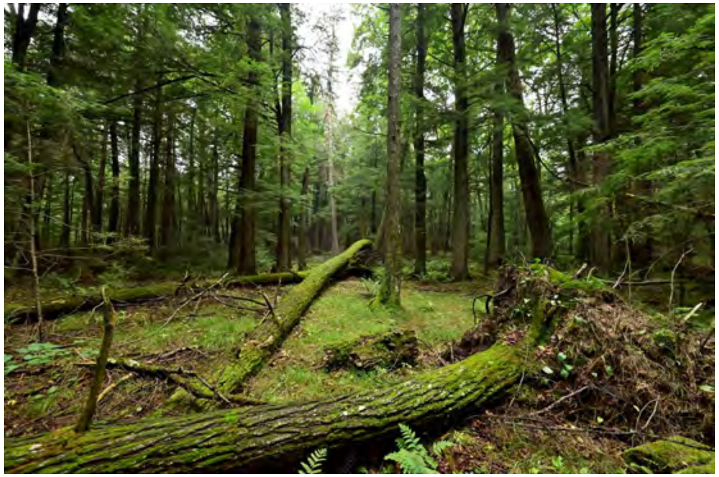

c. Big Loop, Fox Maple Woods (3.14)

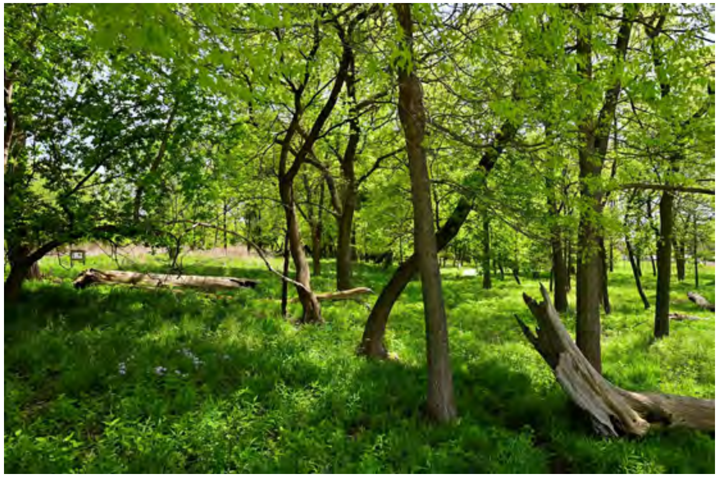

d. Woodland Loop, West Ridge Nature Park (9.21)

3.111. Trees– Stumps and Roots

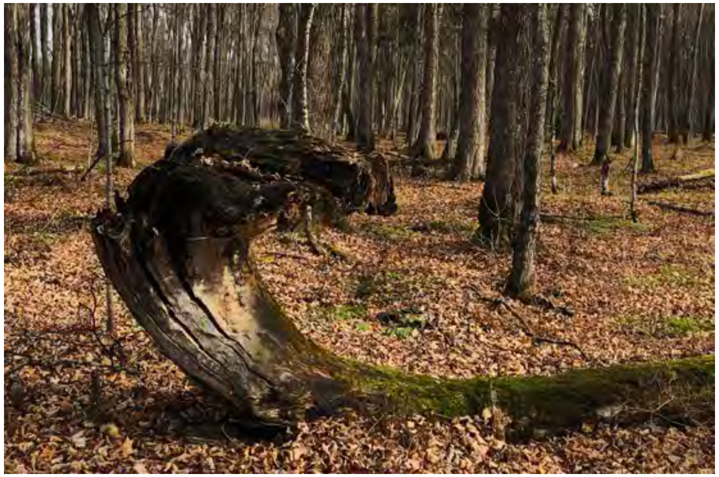

a. Beginner's Trail Loop, Lauterman National Recreation Trail (4.11)

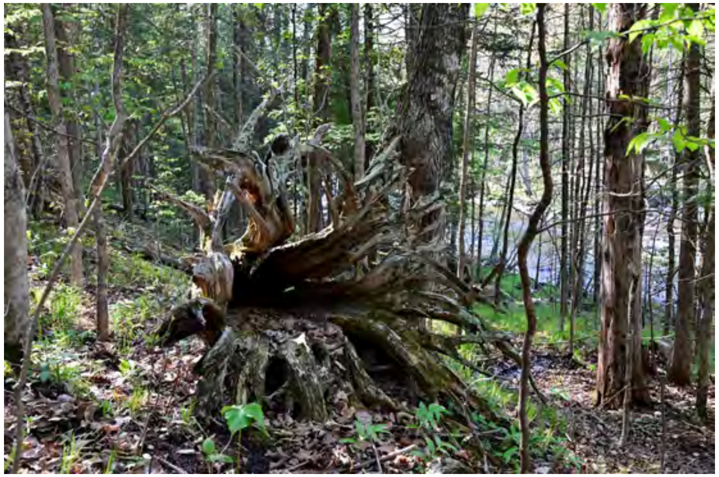

b. North Bank Trail, Pine River-Breakwater Falls (1.61)

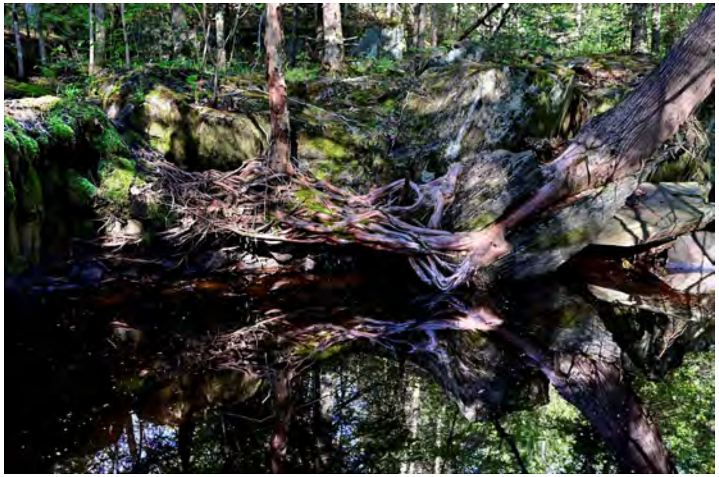

c. North Bank Trail, Pine River-Breakwater Falls (1.61)

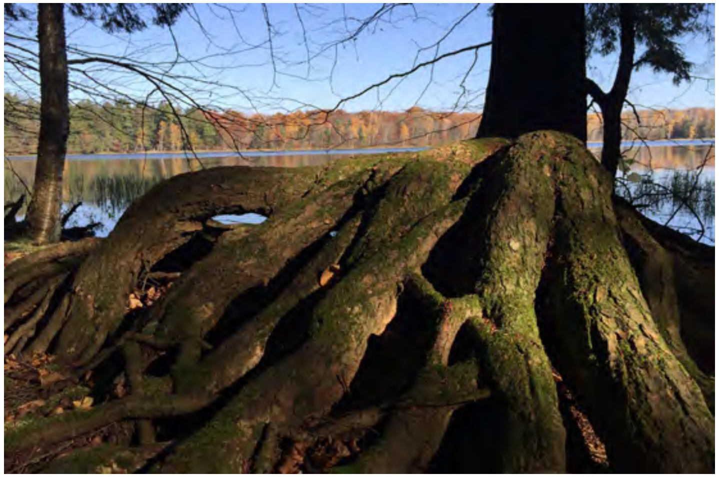

d. West Lakeshore Trail, Lost Lake Recreation Area (4.25)

3.21. Water– Rivers and Streams Northwoods

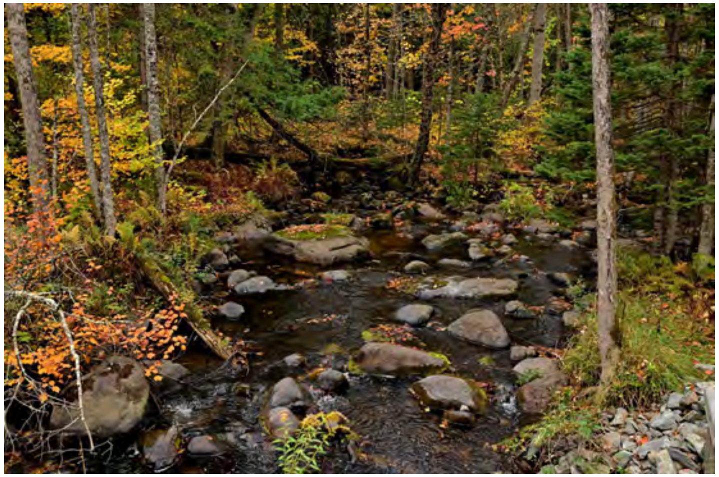

a. Woods Creek, Wild Lakes Bike Loop, Savage-Robago Wild Lakes Complex (1.84)

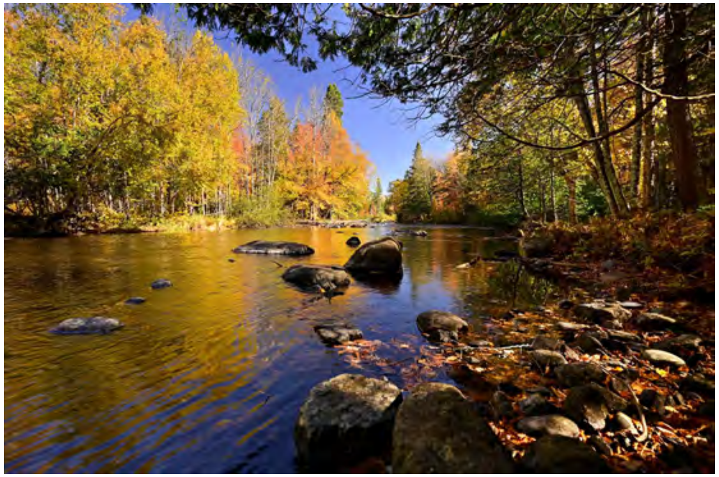

b. Pine River, Savage Lake Road, Savage-Robago Wild Lakes Complex (1.85)

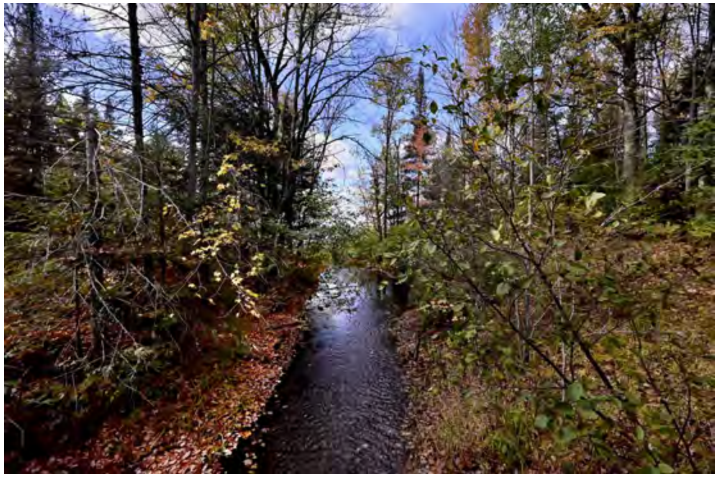

c. Pine River headwaters, McKinley Lake–Luna Lake segment, Hidden Lakes Trail (6.14)

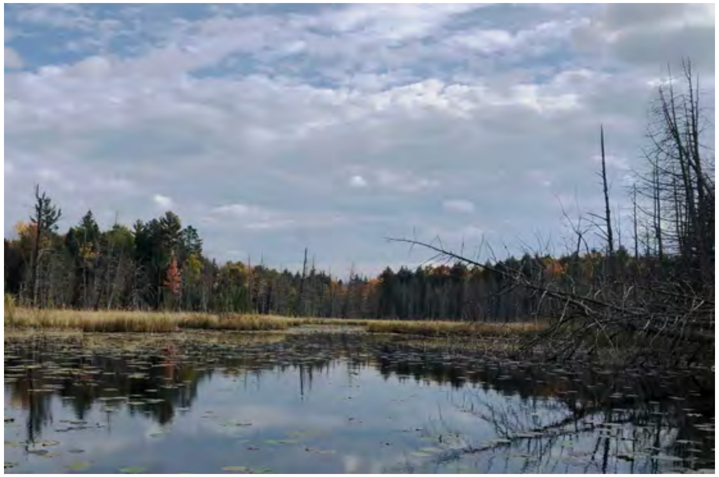

d. Montagne Creek, Edith Lake–Montagne Creek Paddle, Whisker Lake Wilderness (3.44)

3.22. Water– Rivers and Streams Chicago

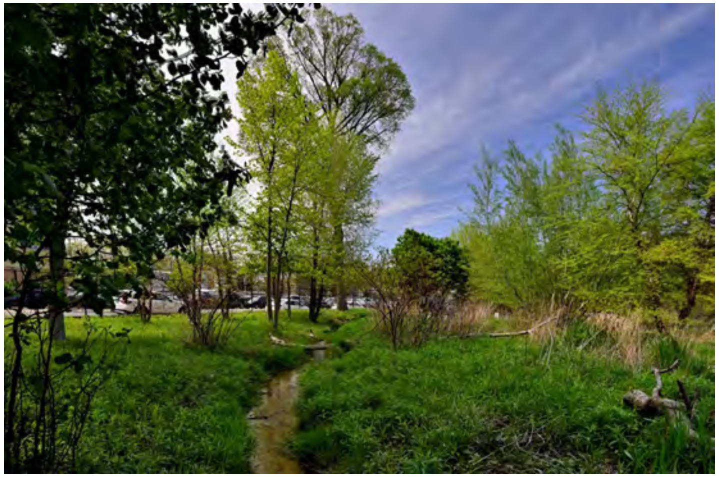

a. Unnamed creek (artificial), Lake Loop, West Ridge Nature Park (9.22)

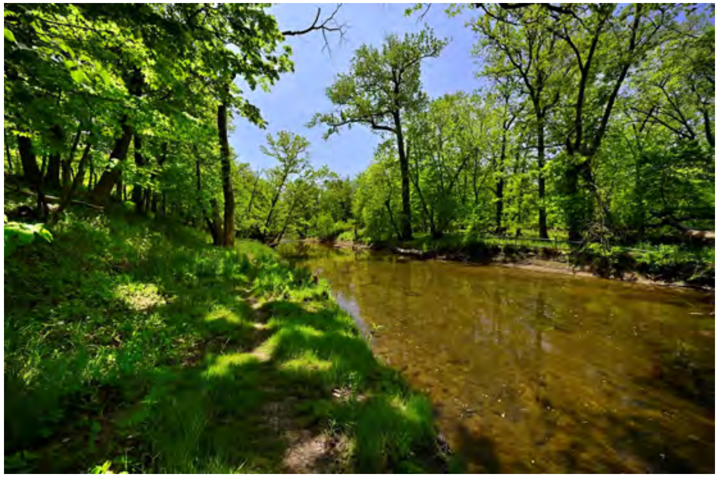

b. North Branch Chicago River, Savanna-Flatwoods-River Loop, Caldwell Preserves (13.11)

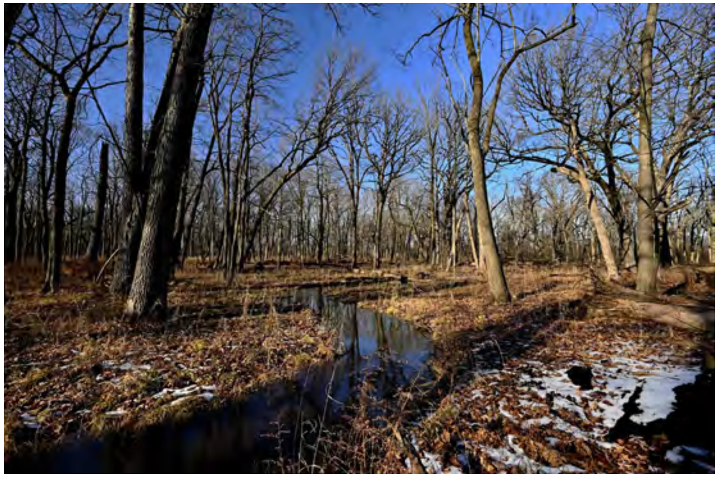

c. North Fork of the Middle Brook of the North Branch Chicago River, East Loop, Somme Woods (16.33)

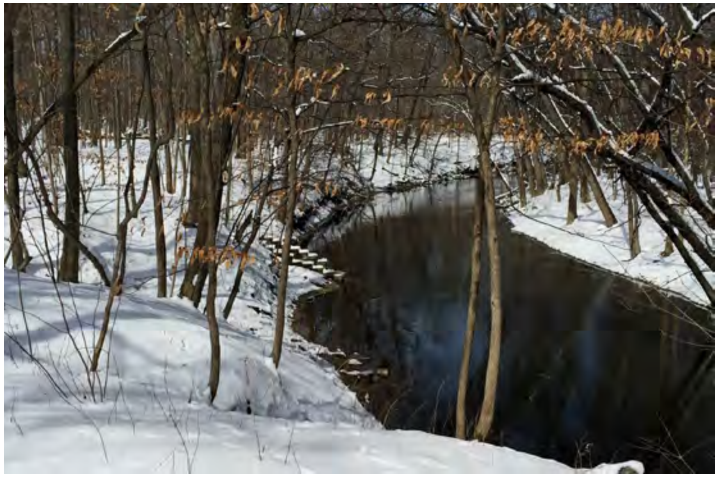

d. North Branch Chicago River, Woodland-River East Loop, Harms Woods Nature Preserve (14.12)

3.23. Water– Lakes and Ponds Northwoods

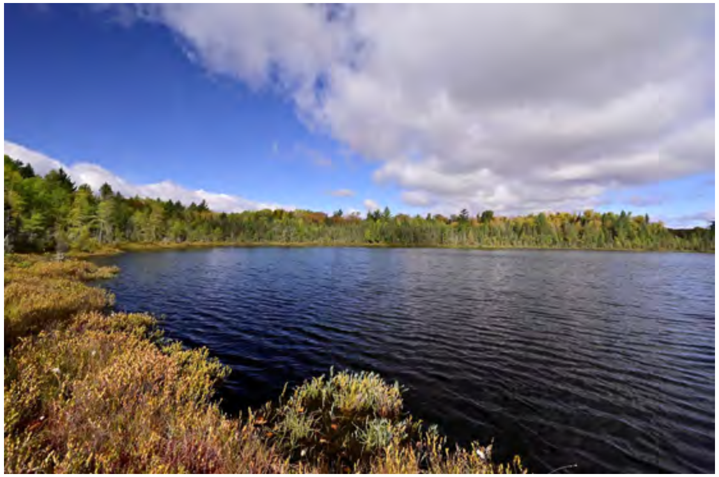

a. Savage Lake, Savage Lake Shoreline Walk (1.81)

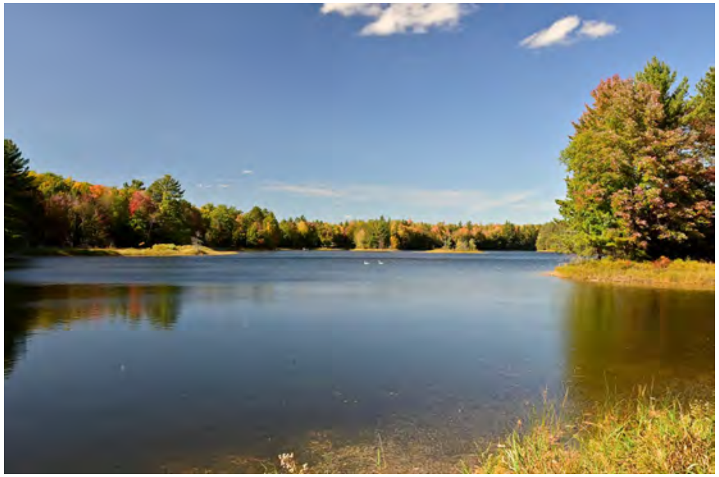

b. Three Johns Lake, Hidden Lakes Dispersed Sites (6.2)

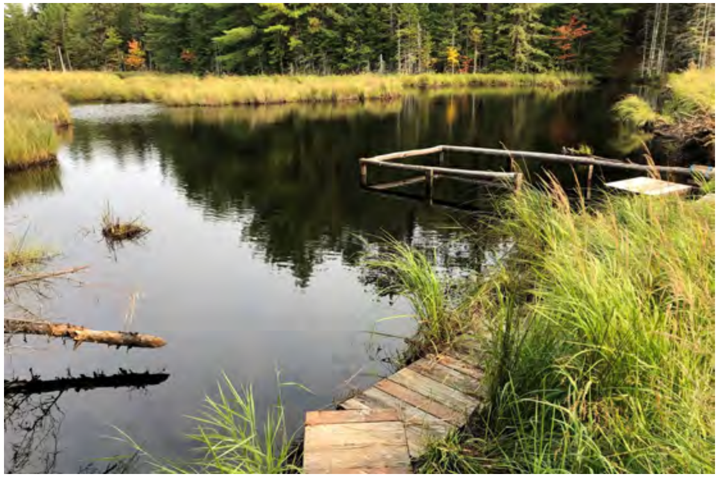

c. Aquatic Labyrinth, Healing Nature Center (6.31)

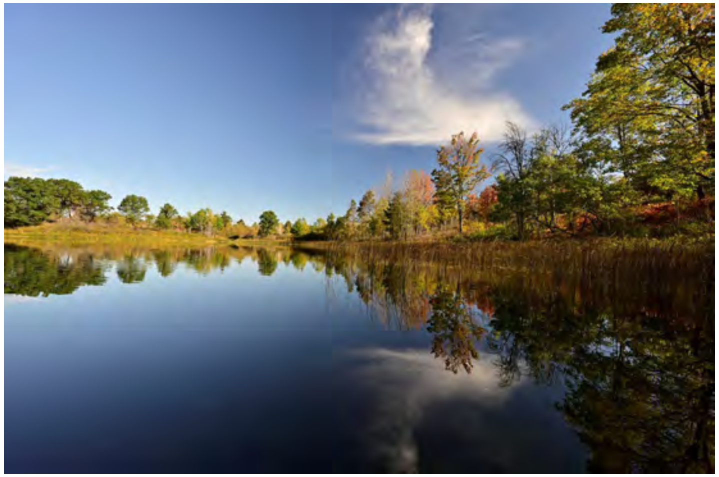

d. Barrens Lake, Spread Eagle Barrens (7.2)

3.24. Water– Lakes and Ponds Chicago

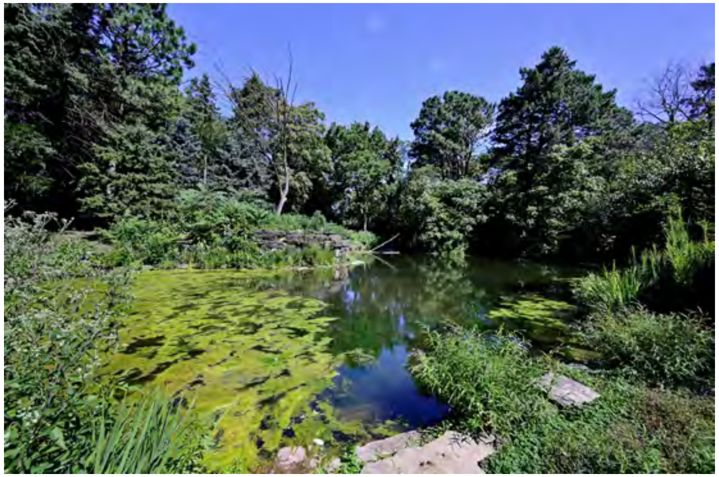

a. Wetland Pond, North Park Village Nature Center (9.13)

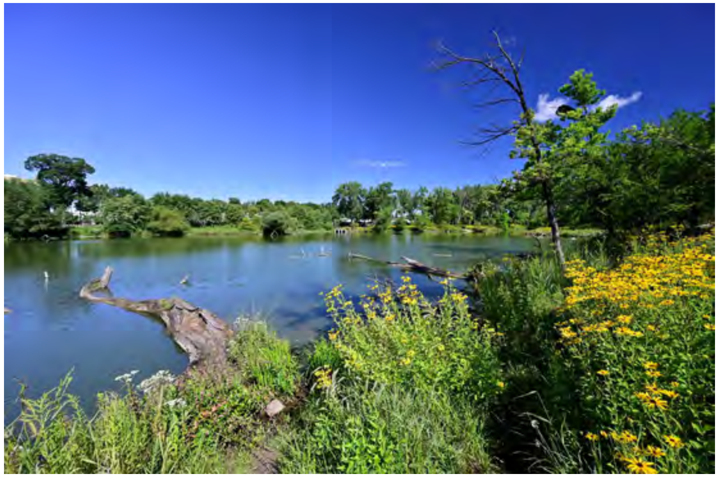

b. Unnamed Pond, West Ridge Nature Park (9.22)

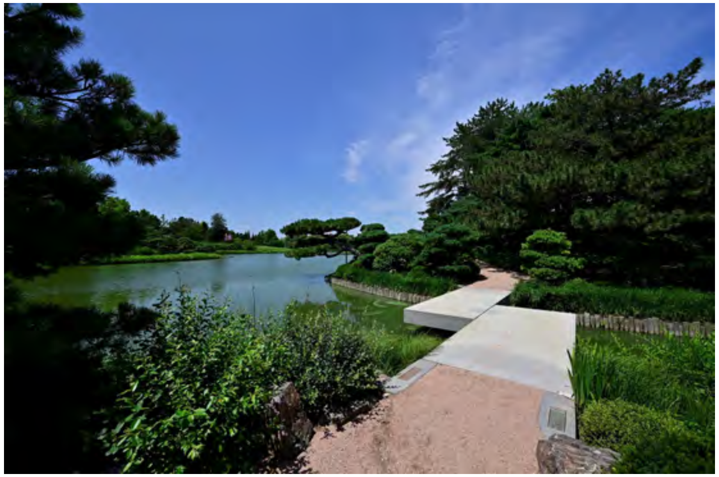

c. Lake at Japanese Garden, Chicago Botanic Garden (15,24)

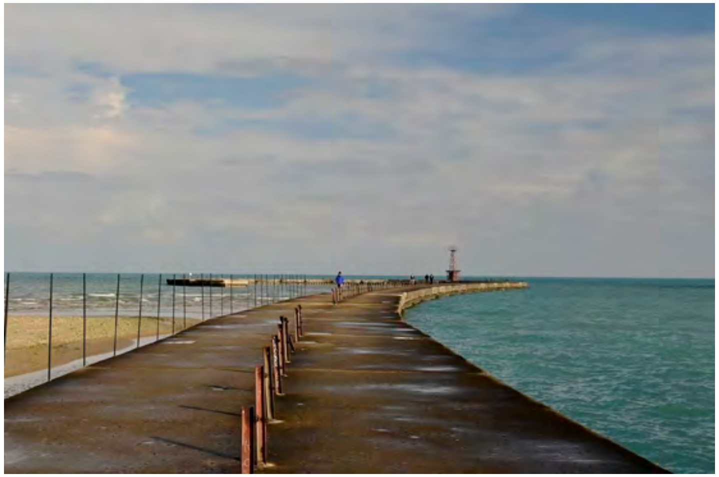

d. Lake Michigan, Breakwater-Pier, Montrose Point (11.14)

3.25. Water– Wetlands and Bogs Northwoods

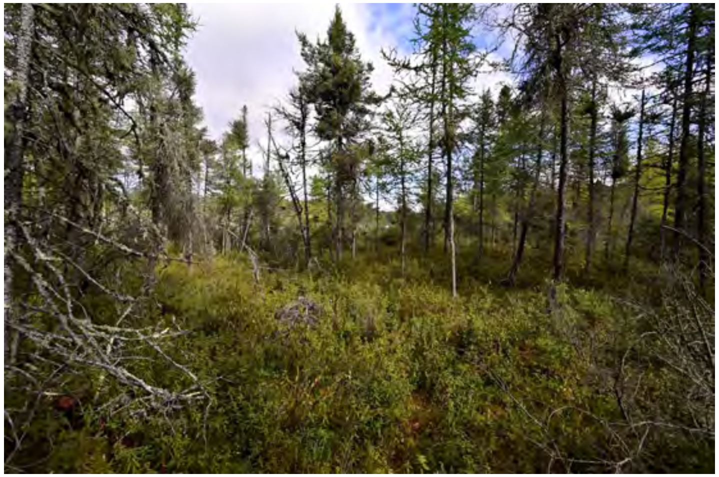

a. Two Sisters Lake Bog Walk, Hidden Lakes Dispersed Sites (6.23)

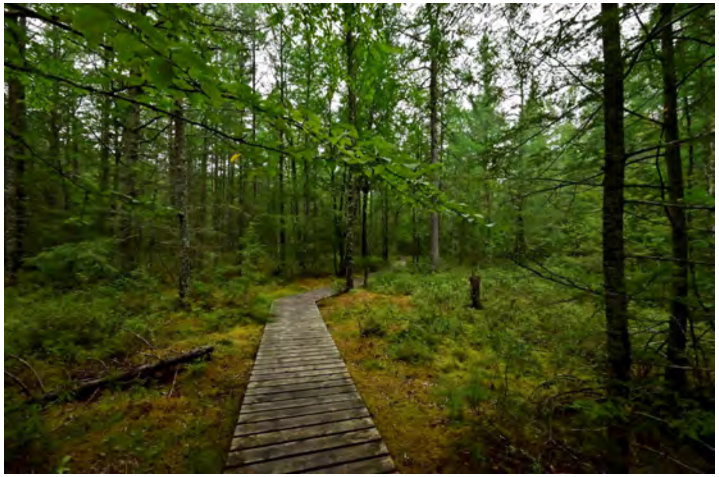

b. Bog at Franklin Nature Trail, Hidden Lakes Trail (6.11)

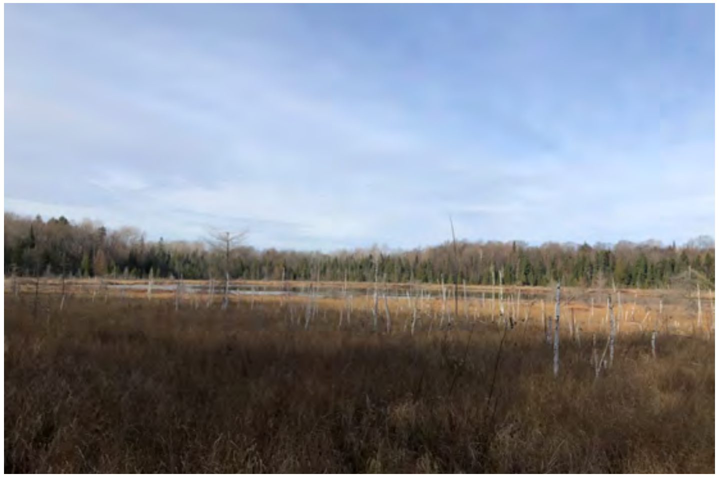

c. Little Porcupine Lake, Chipmunk-Little Porky Loop, Lauterman National Recreation Trail (4.13)

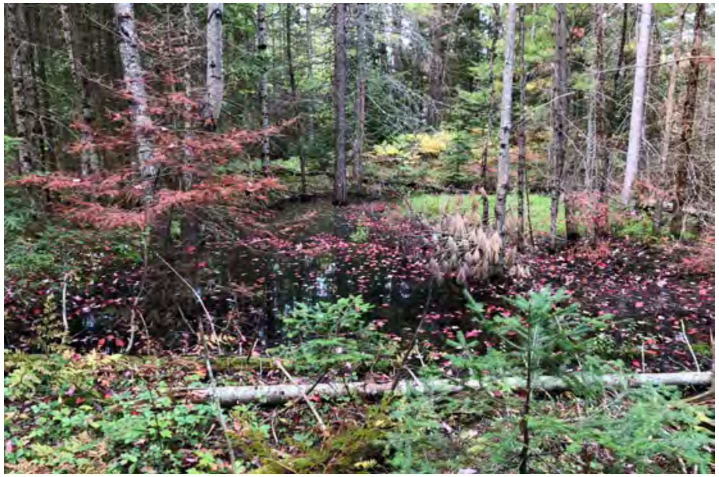

d. Wetland at Healing Nature Trail (6.31)

3.26. Water– Wetlands and Rain Gardens Chicago

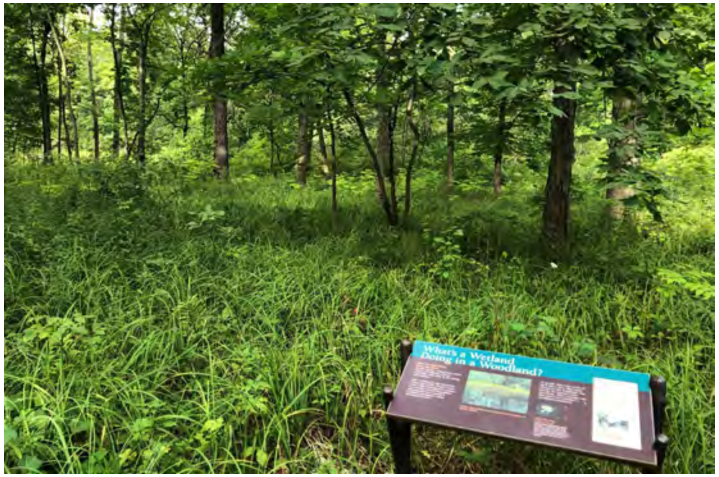

a. Woodland Wetland, McDonald Woods North Loop, Chicago Botanic Garden (15.22)

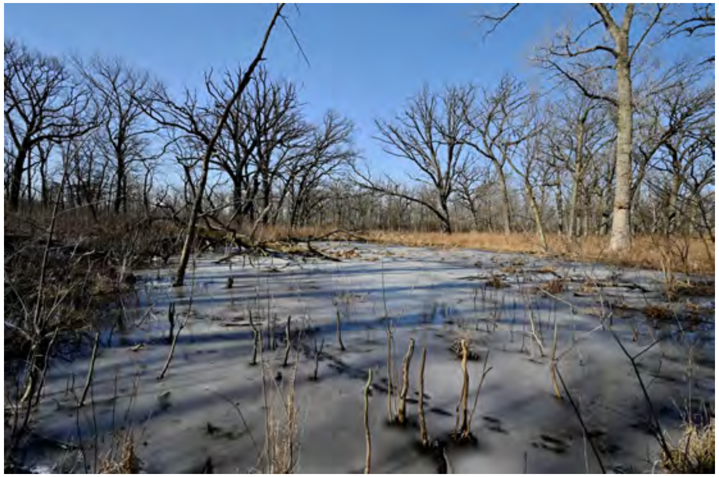

b. Wetland Pond, West Inner Loop, Somme Woods (16.31)

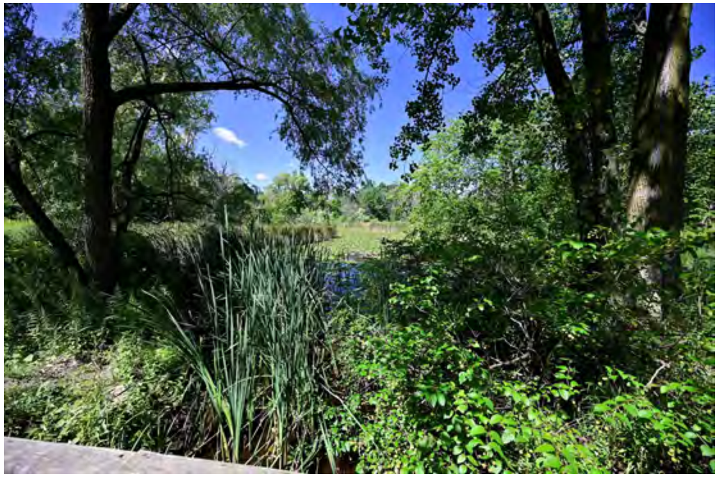

c. Wetland Pond, Wetland Loop, North Park Village Nature Center (19.11)

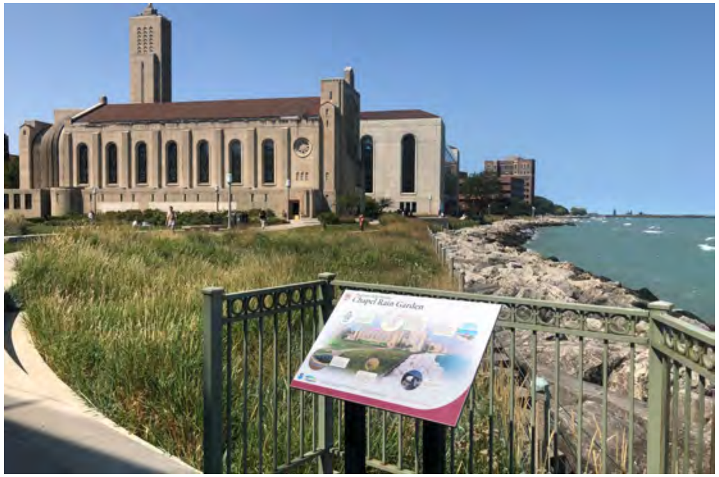

d. Rain Garden, Campus Loop Walk, Loyola University Lakeshore Campus (10.11)

3.27. Water–Moving Water Northwoods

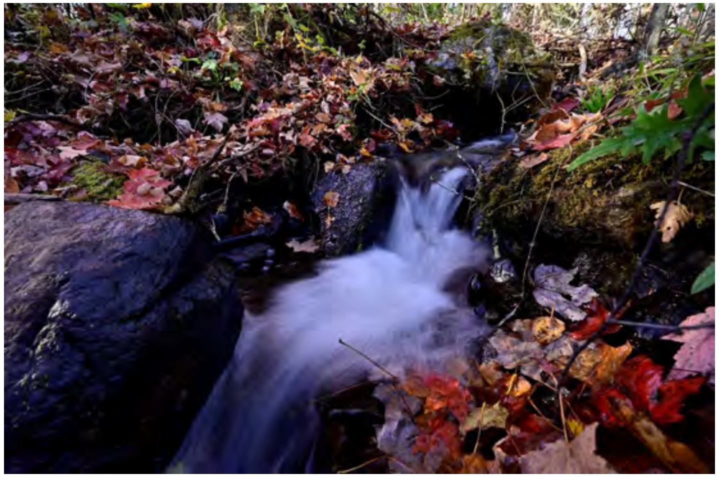

a. Unnamed Creek, Outcrops-Western Trail, Pine River Outcrops (1.32)

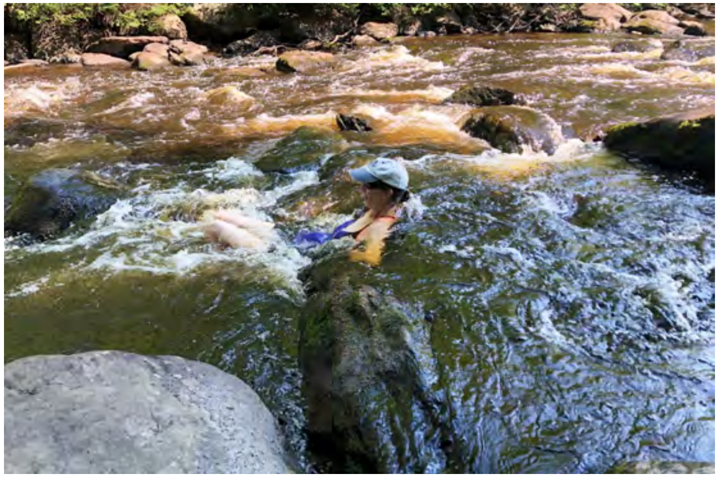

b. Bull Falls, Pine River, Bull Falls Trail (1.42)

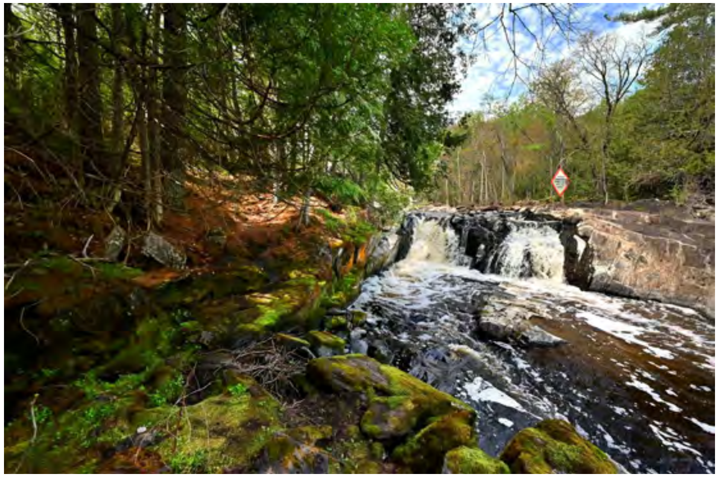

c. 2nd Drop, Breakwater Falls Pine River, South Bank Trail, Breakwater Falls (1.62)

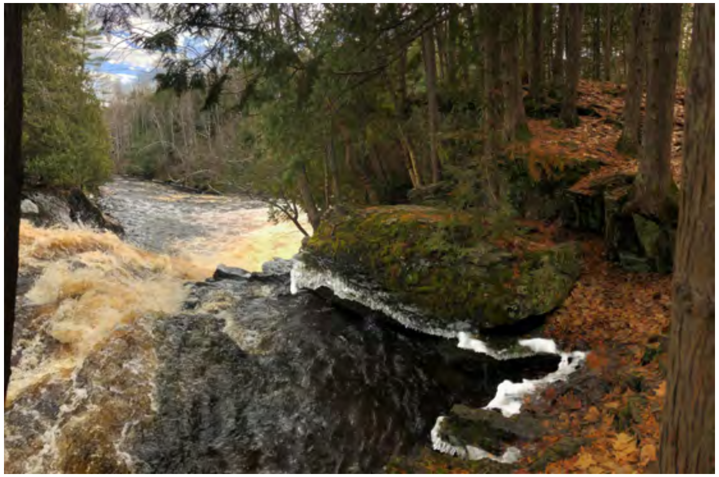

d. LaSalle Falls, Pine River, LaSalle Falls Trail (1.51)

3.28. Water– Moving Water Chicago

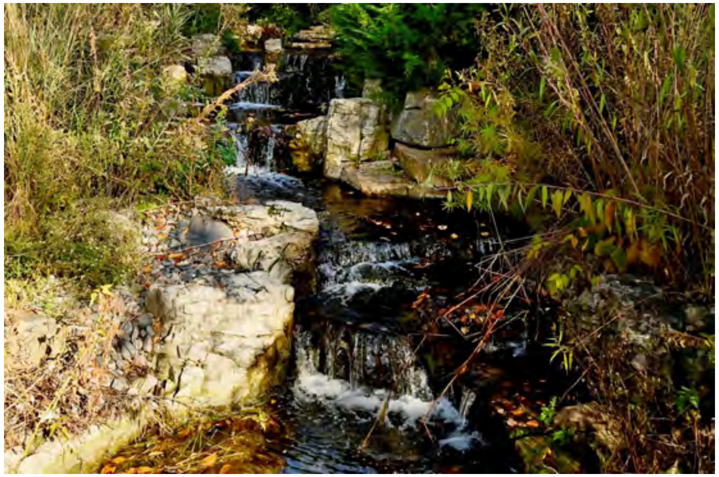

a. Waterfall Fountain, Nature Boardwalk, Lincoln Park Zoo (12.41)

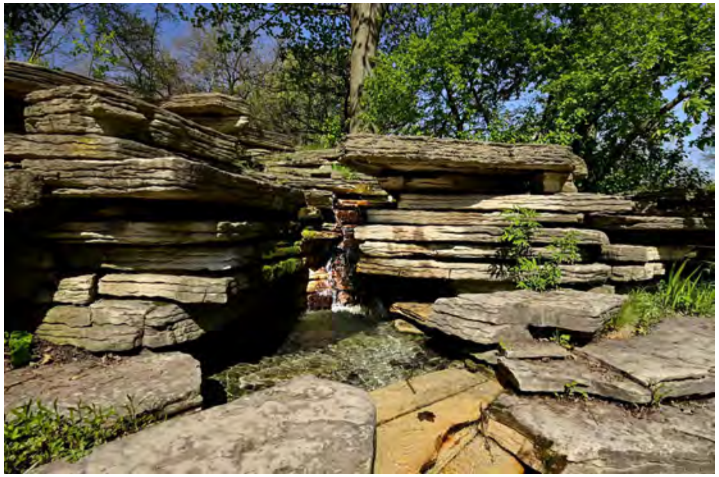

b. Waterfall Fountain, Lily Pool Loop, Alfred Caldwell Lily Pool (12.11)

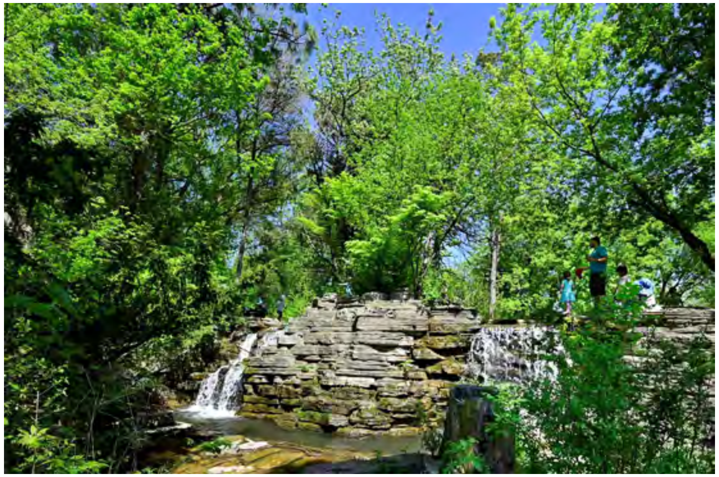

c. Waterfall Fountain, Rock Garden Loop, North Park Village Nature Center (9.14)

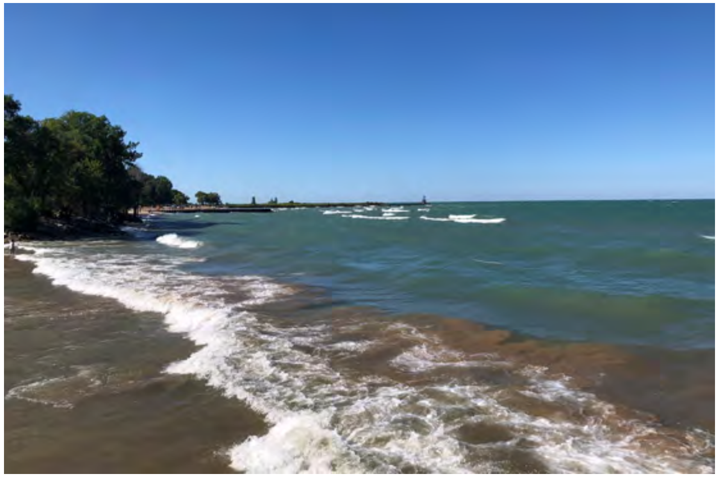

d. Lake Michigan, Hartigan-Leone Beach Walk, Loyola-Leone Parks, (10.31)

3.29. Water– Frozen Water

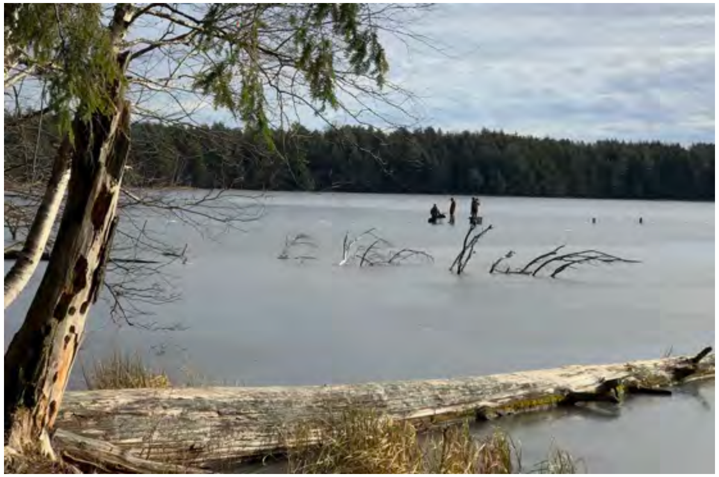

a. Ice fishing at Lost Lake, Lost Lake Recreation Area (4.2)

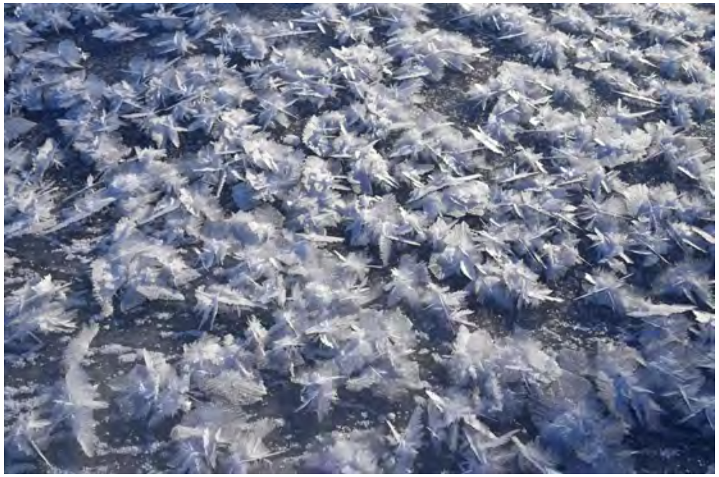

b. Ice crystals, Sea Lion Lake (2.1)

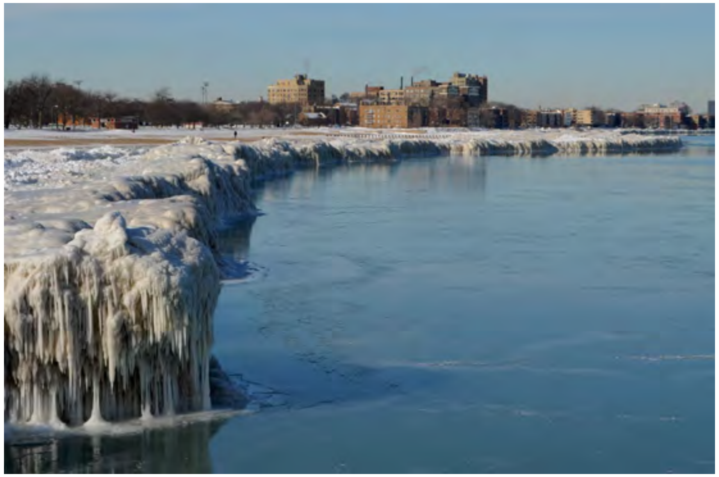

c. Ice formations along Lake Michigan, Hartigan-Leone Beach Walk, Loyola-Leone Parks, (10.31)

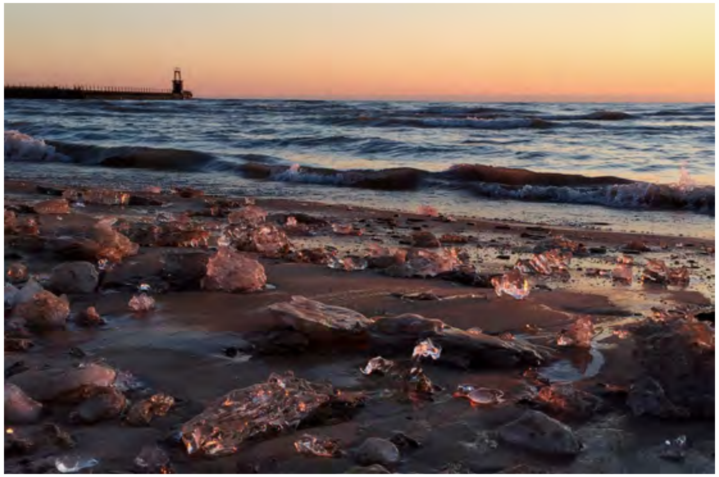

d. Ice chunks along beach of Lake Michigan, Hartigan-Leone Beach Walk, Loyola-Leone Parks, (10.31)

3.31. Wildlife– Big and Small

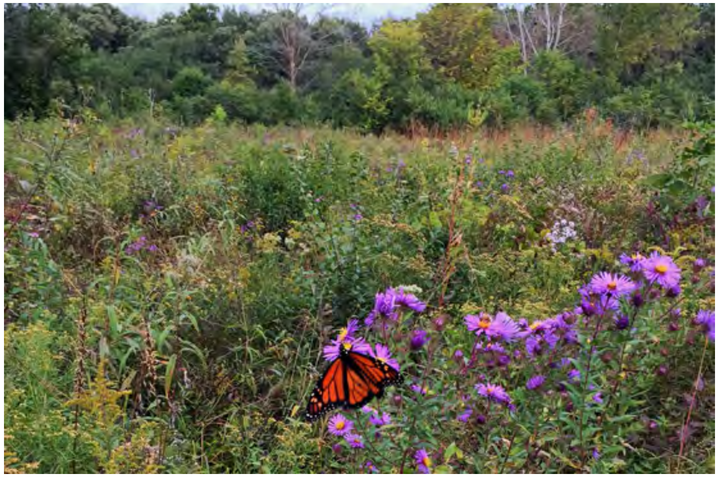

a. Monarch, Prairie Inner Loop, Somme Prairie Grove (16.23)

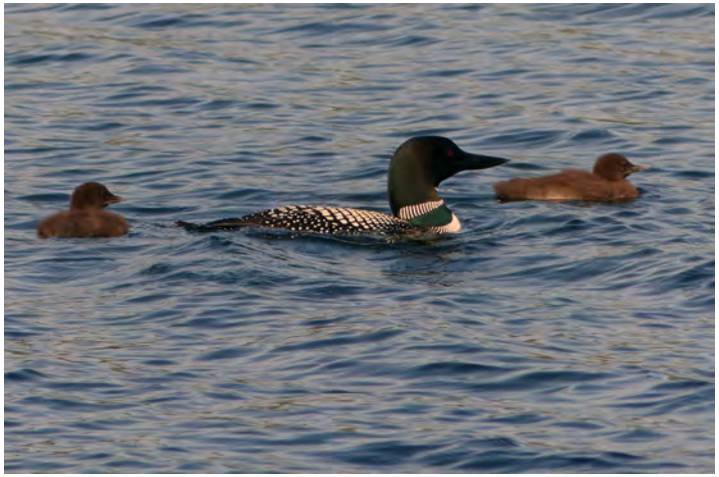

b. Loons, Islands and Bays Paddle, Sea Lion Lake (2.14)

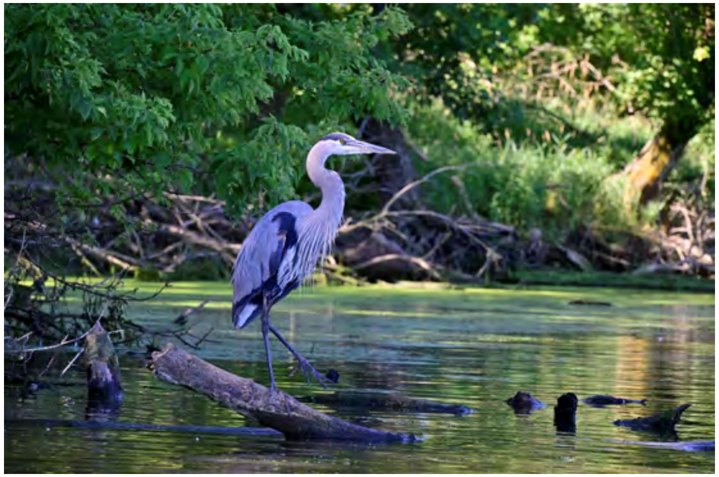

c. Great Blue Heron, Skokie Lagoons (15.17)

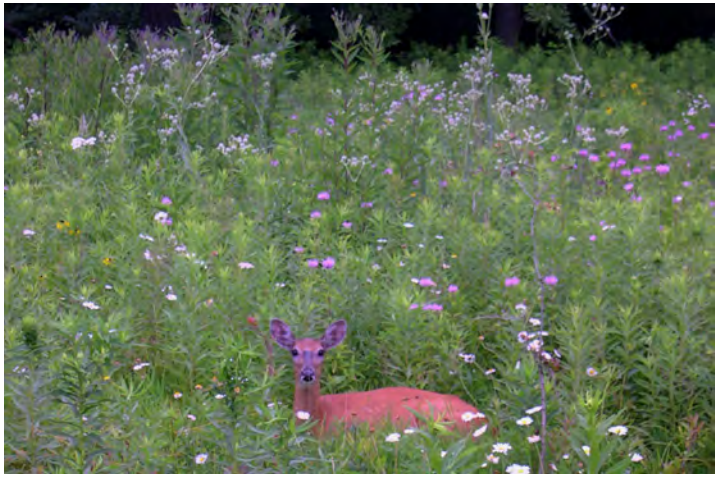

d. White-tailed deer, Prairie Inner Loop, Somme Prairie Grove (16.23)

3.32. Wildlife– Homes and Habitats

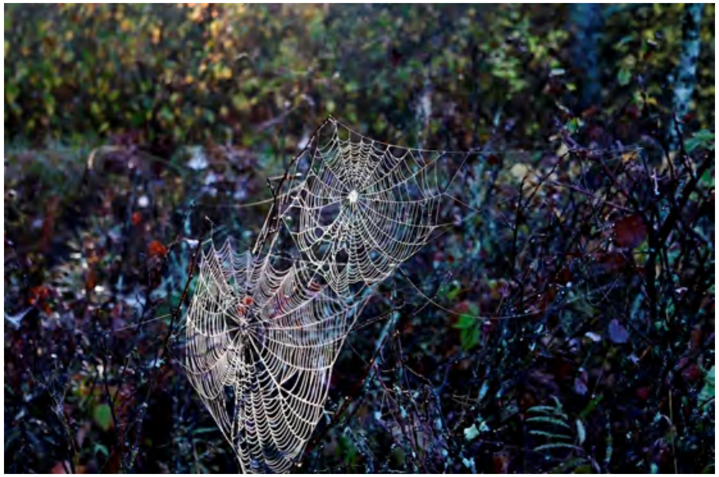

a. Spider web, Barrens Lake Short Loop, Spread Eagle Barrens (7.21)

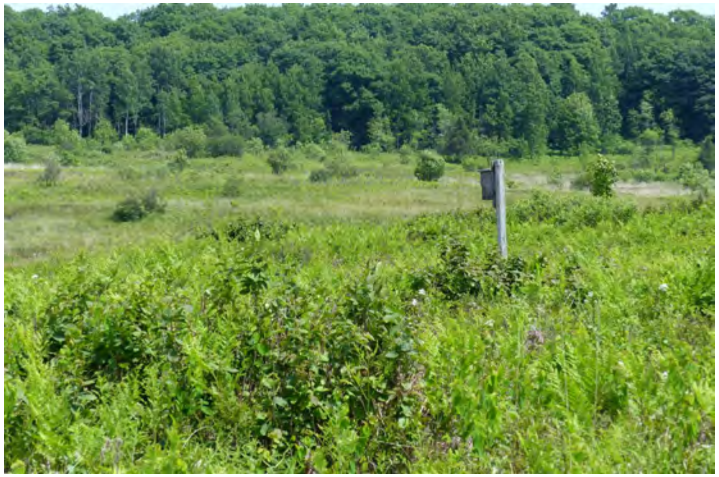

b. Nesting box, Fire Lane Rd. Loop, Spread Eagle Barrens (7.12)

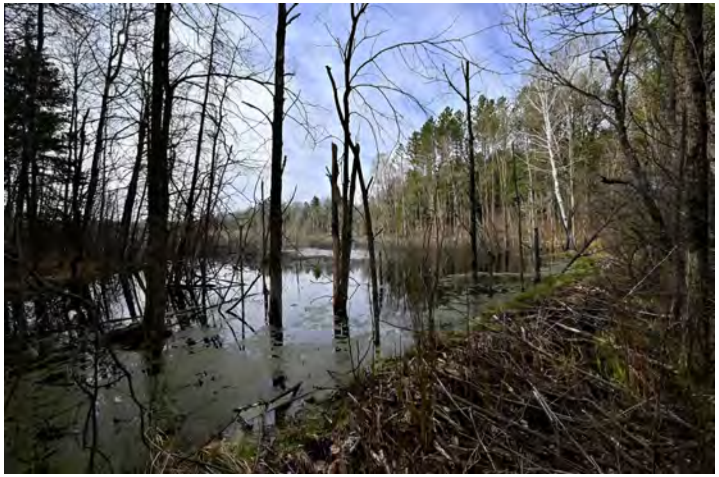

c. Beaver dam, South Loop, Rainbow Hunter Walking Trails (3.53)

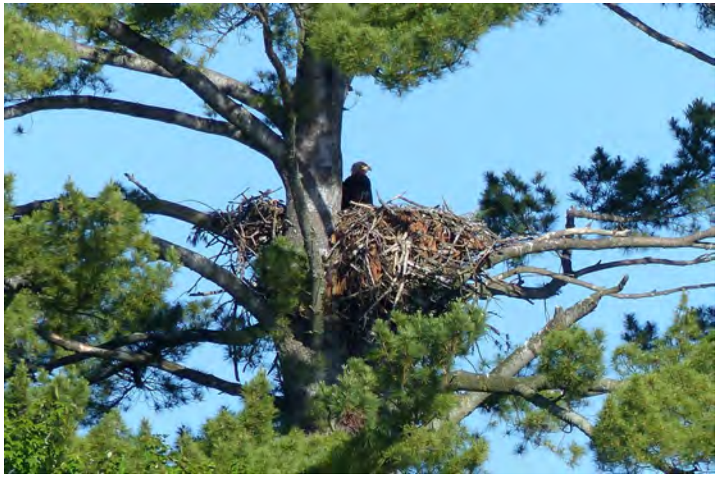

d. Eagle’s nest, Islands and Bays Paddle, Sea Lion Lake (2.14)

3.33. Wildlife– Tracks and Traces

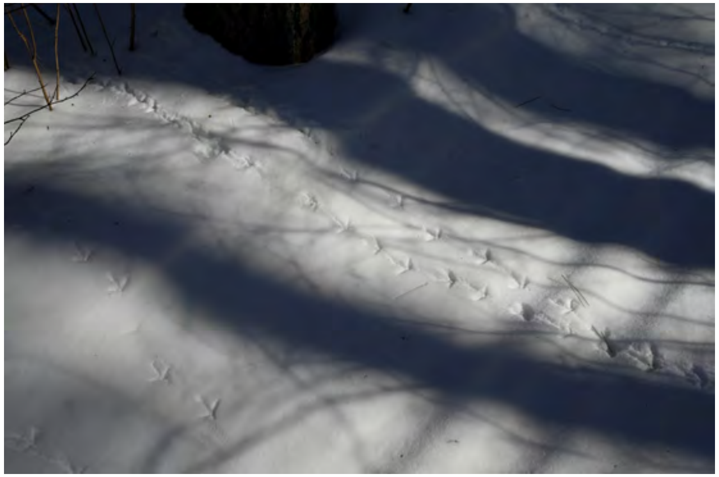

a. Ruffed Grouse tracks, Chipmunk-Little Porky Loop, Lauterman National Recreation Trail (4.13)

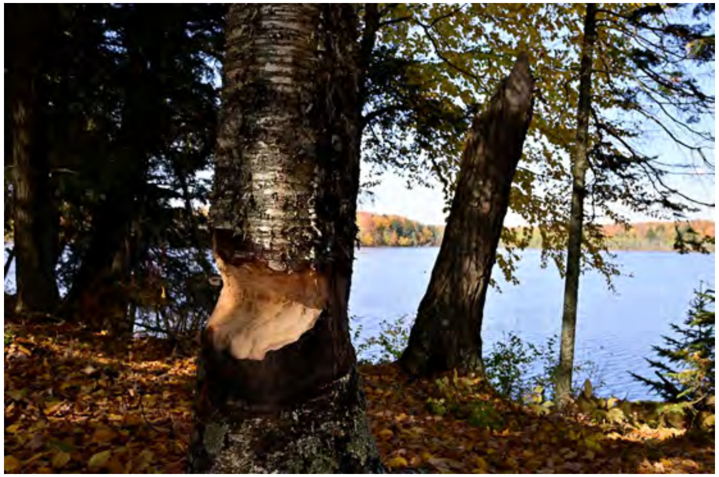

b. Beaver gnawing traces, Wild Lakes Land and Water Loop, Savage-Robago Wild Lakes Complex (1.83)

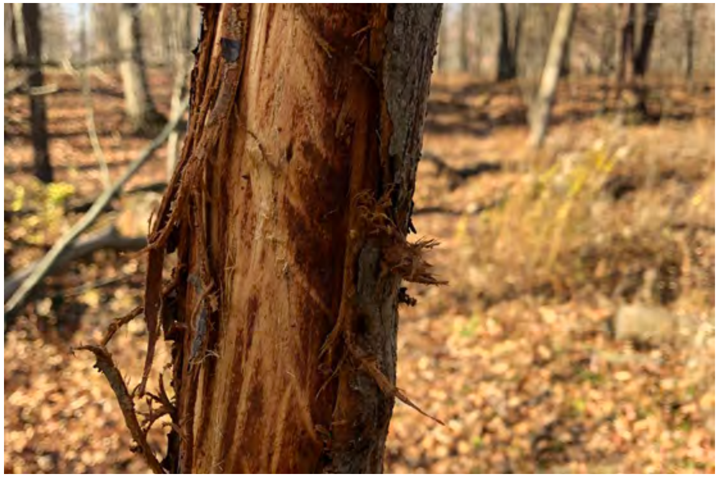

c. Deer antler rubbing, Savanna-Flatwoods-Loop, Caldwell Preserves (13.11)

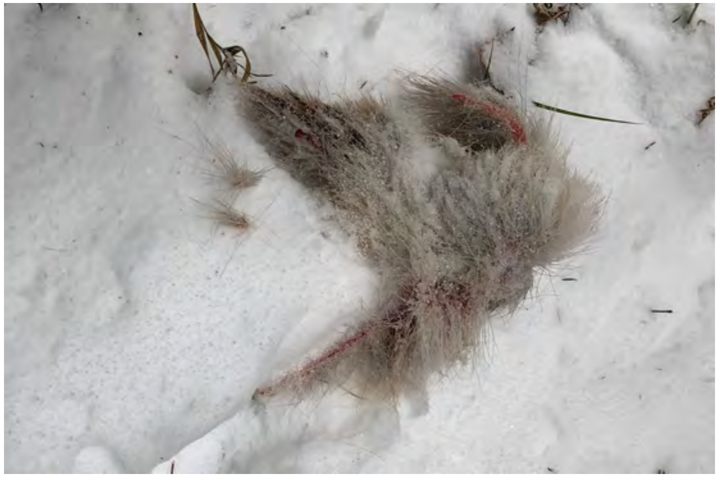

d. Snowshoe Hare fur and blood, Pine River Flats (1.21)

3.41. Groundcover Flora– Woodland Spring Ephemerals Northwoods

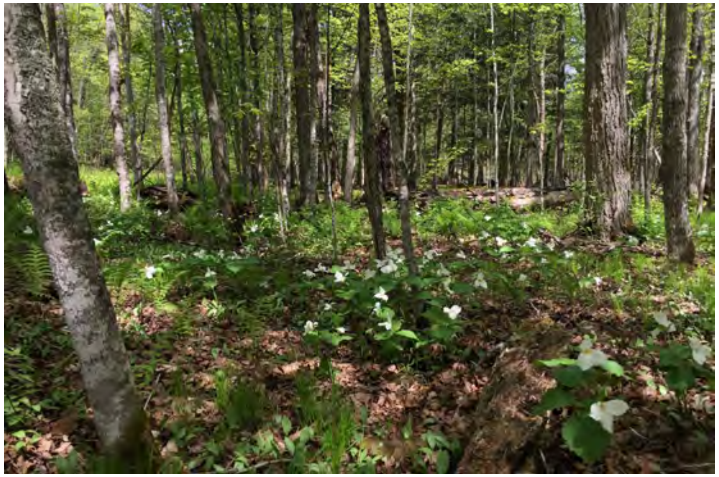

a. Trillium, Upland Loop, Fox Maple Woods (3.13)

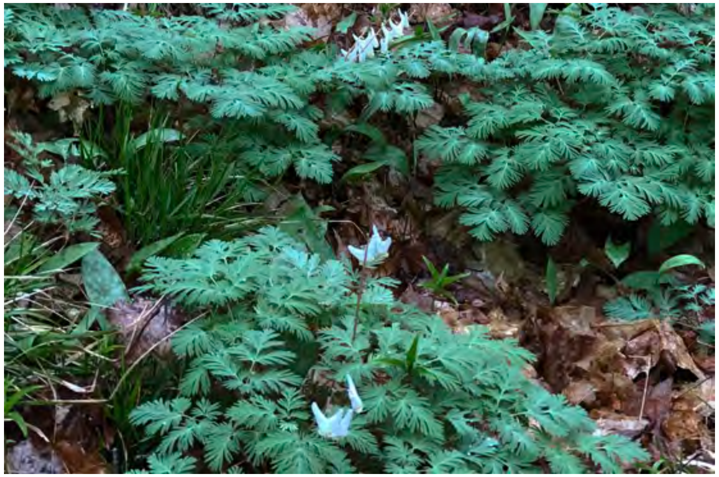

b. Dutchmen’s Breeches, Marsh Loop, Fox Maple Woods (3.12)

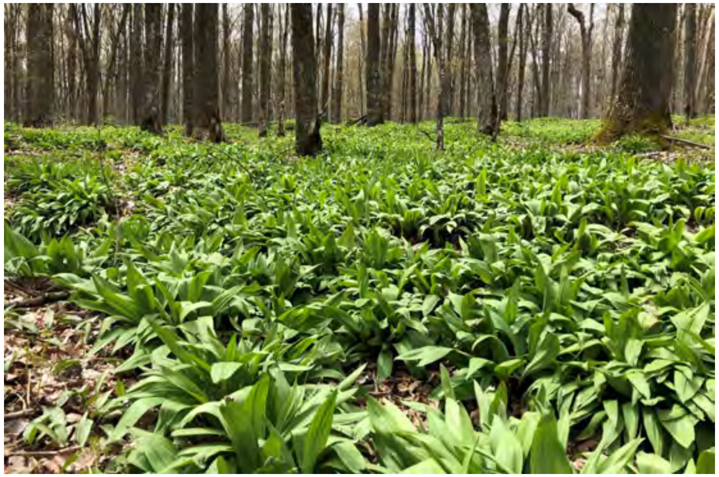

c. Ramps, Lakeshore Trail Loop, Lost Lake Recreation Area (4.21)

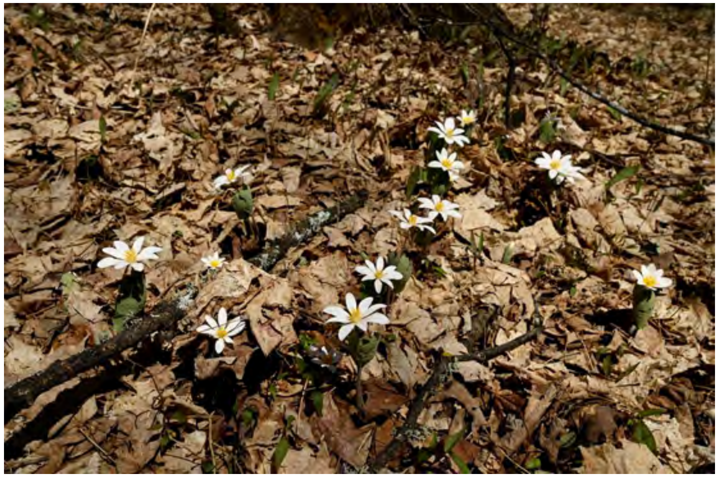

d. Bloodroot, Lakeshore-Ridge Trail Loop, Lost Lake Recreation Area (4.23)

3.42. Groundcover Flora– Woodland Spring Ephemerals Chicago

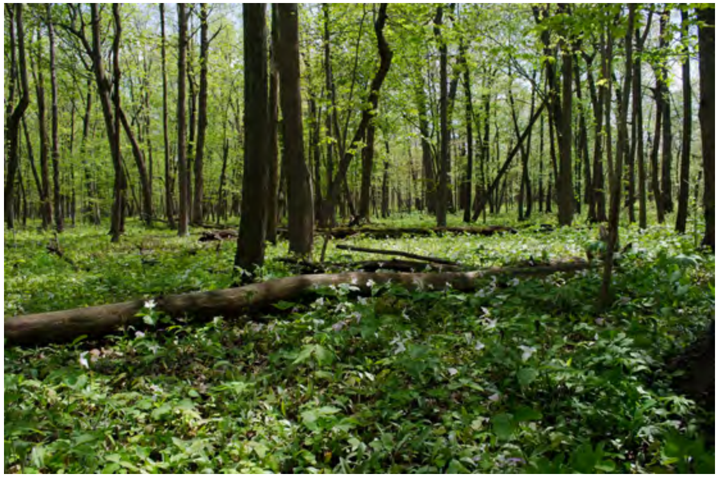

a. Trillium, Woodland-River West Loop, Harms Woods Nature Preserve (14.11)

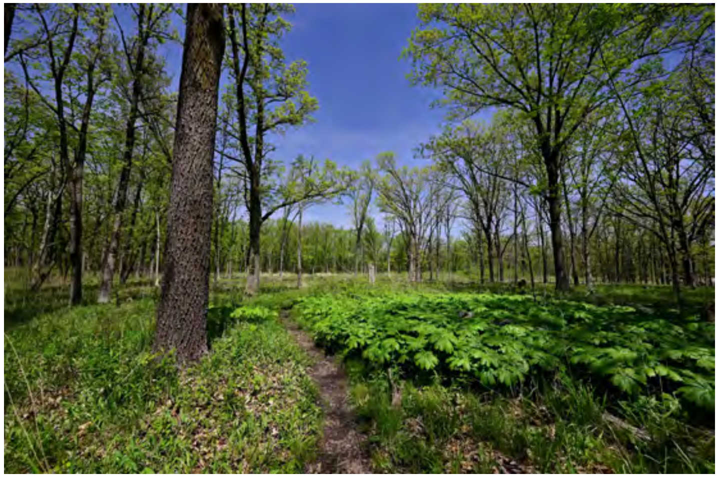

b. Mayapple, West Inner Loop, Somme Woods (16.31)

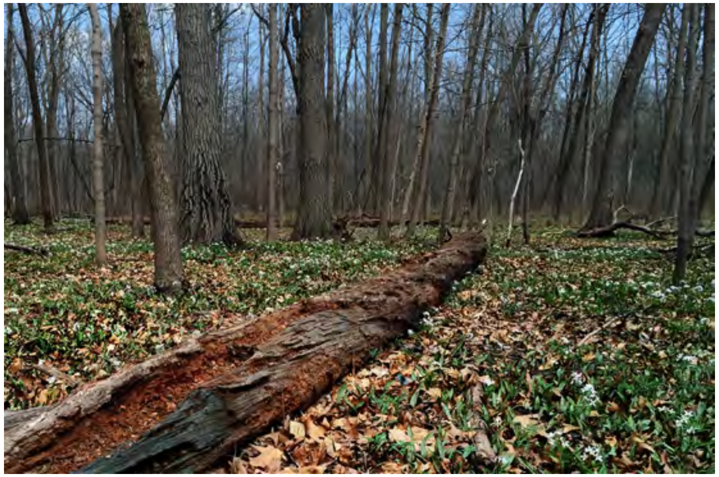

c. Spring Beauty, Woodland-River West Loop, Harms Woods Nature Preserve (14.11)

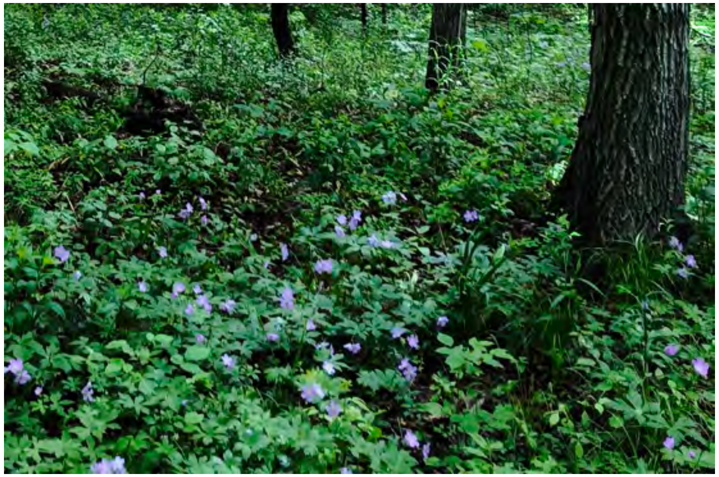

d. Wild Geranium, Woodland-River West Loop, Harms Woods Nature Preserve (14.11)

3.43. Groundcover Flora– Pine Barrens & Old Field Northwoods

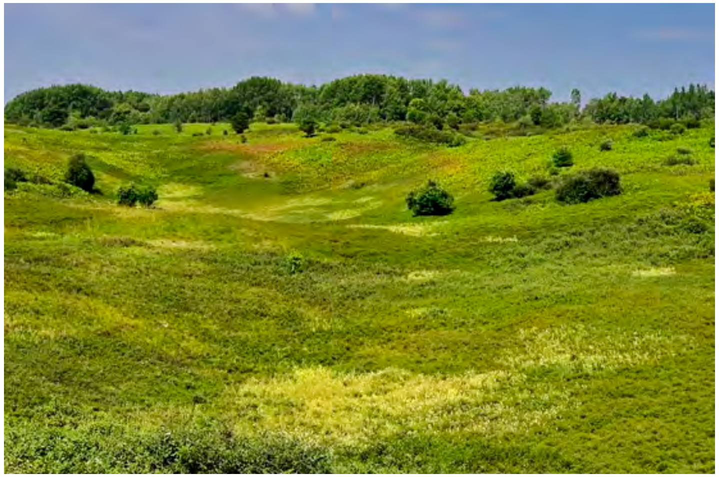

a. Yellow Toadflax, Lake Anna Full Loop, Spread Eagle Barrens (7.33)

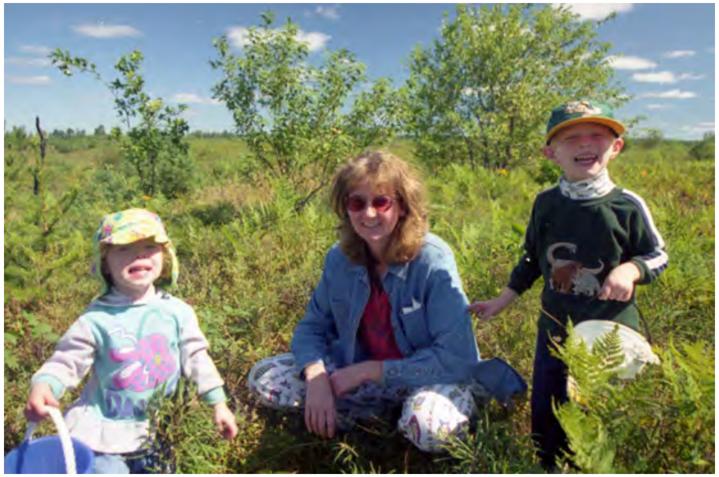

b. Blueberries, Fire Ln. Rd. Loop, Spread Eagle Barrens (7.12)

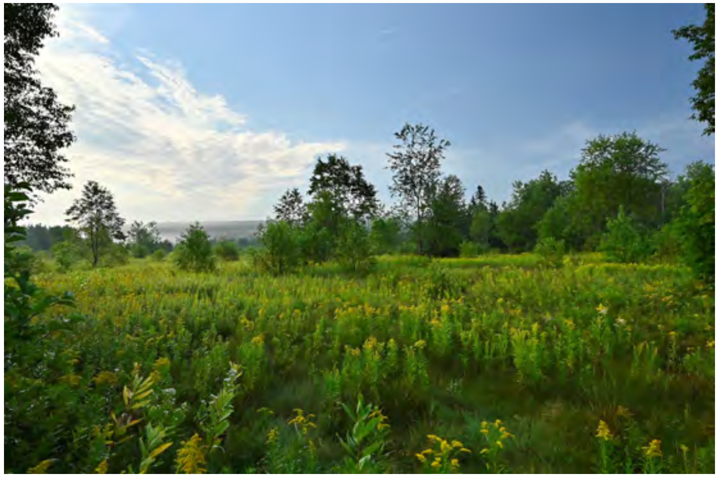

c. Goldenrod, Old Field Loop, Brule River Cliffs (5.11)

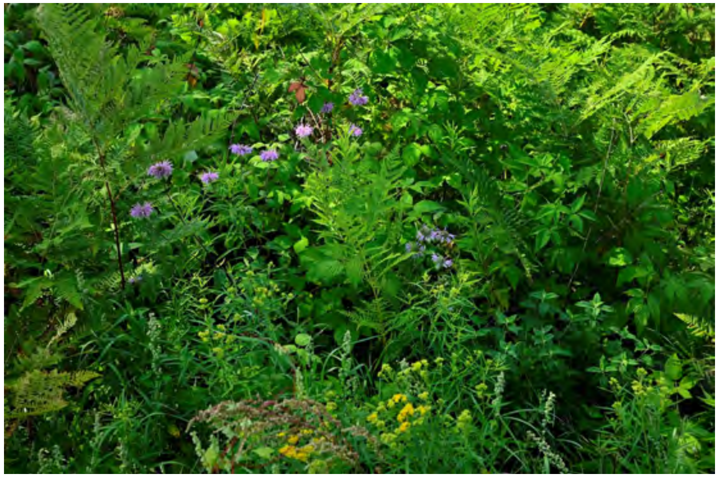

d. Wild Bergamot, Lake Anna West Loop, Spread Eagle Barrens (7.31)

3.44. Groundcover Flora– Prairie/Oak Savanna Chicago

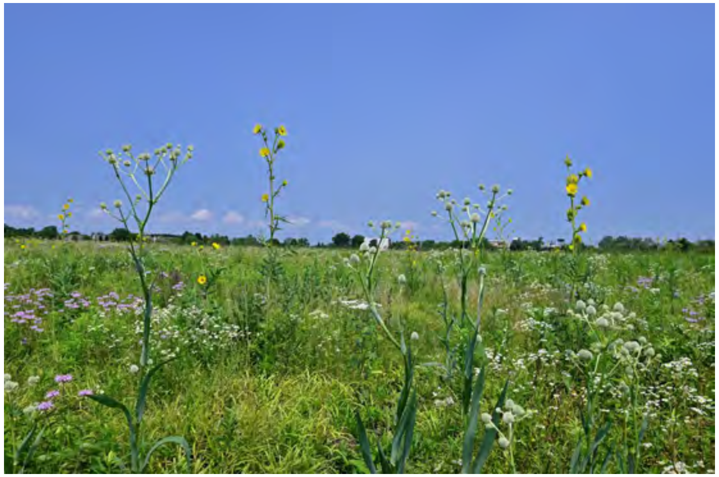

a. Rattlesnake Master and Compass Plant, Prairie Loop, Somme Prairie (16.11)

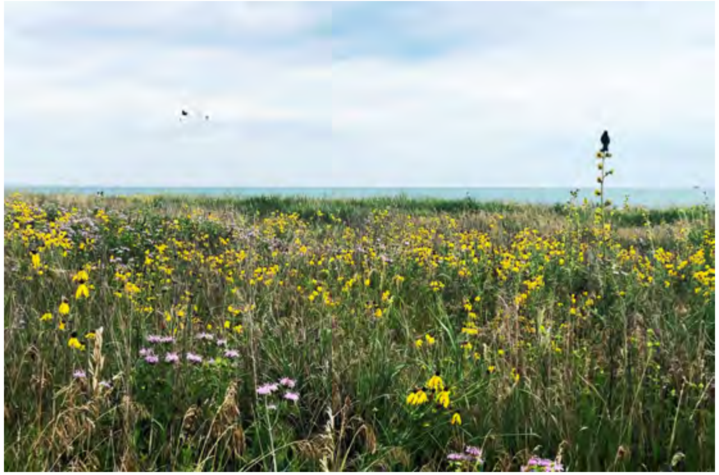

b. Yellow Coneflower and Wild Bergamot, Point-Dunes-Lake-Prairie Loop, Montrose Point (11.13)

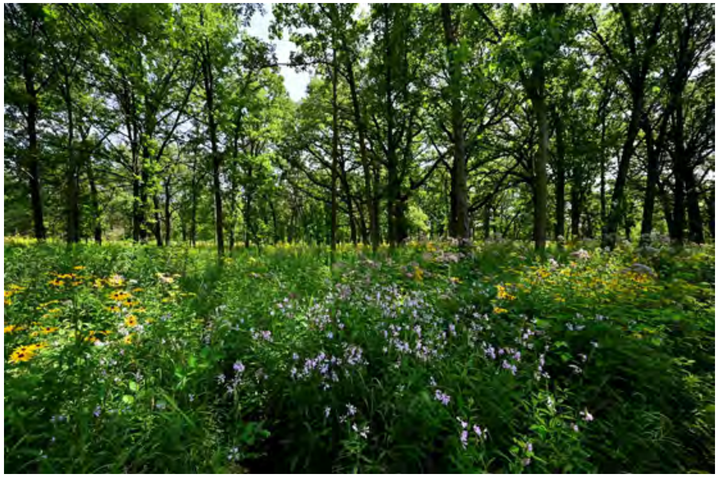

c. Obedient Plant and Brown-Eyed Susan, Vestal Grove Savanna Loop, Somme Prairie Grove (16.21)

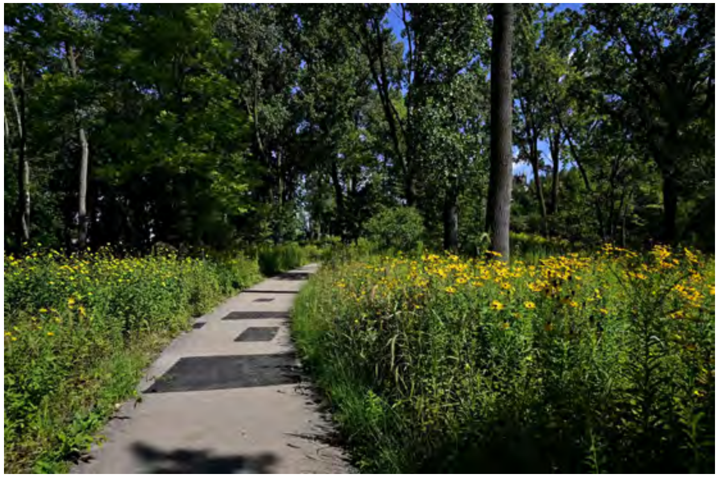

d. Brown-Eyed Susan, Woodland Loop, West Ridge Nature Park (9.21)

3.45. Aquatic Flora

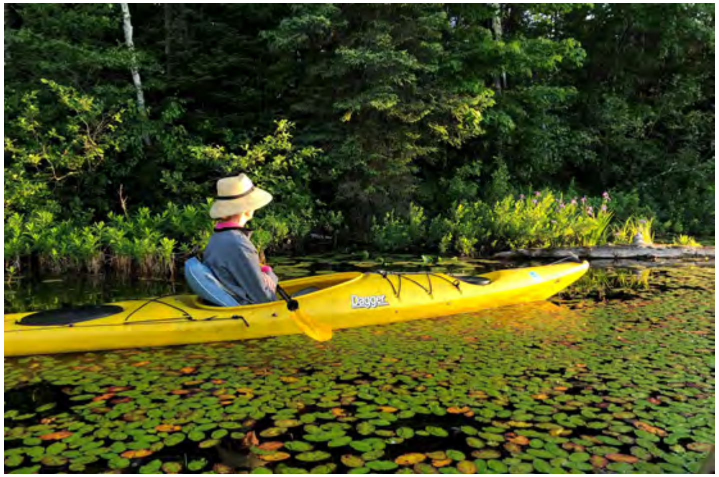

a. Watershield and Blue Flag Iris, Islands and Bays Paddle, Sea Lion Lake (2.14)

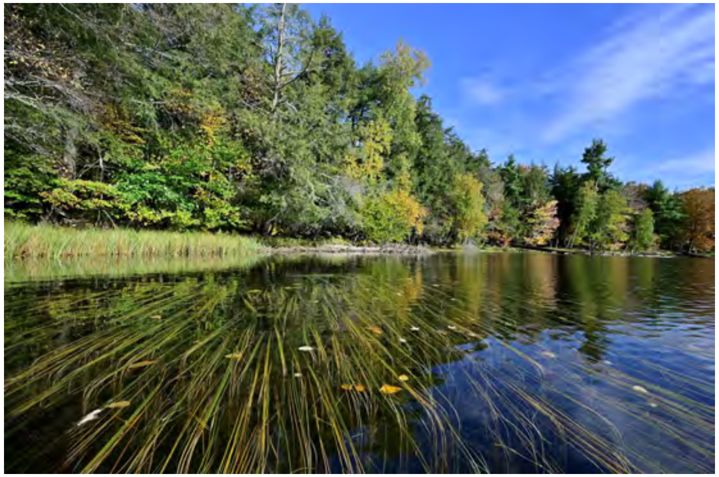

b. Bur-reed and lake sedge, Lost Lake Paddle, Lost Lake Recreation Area (4.26)

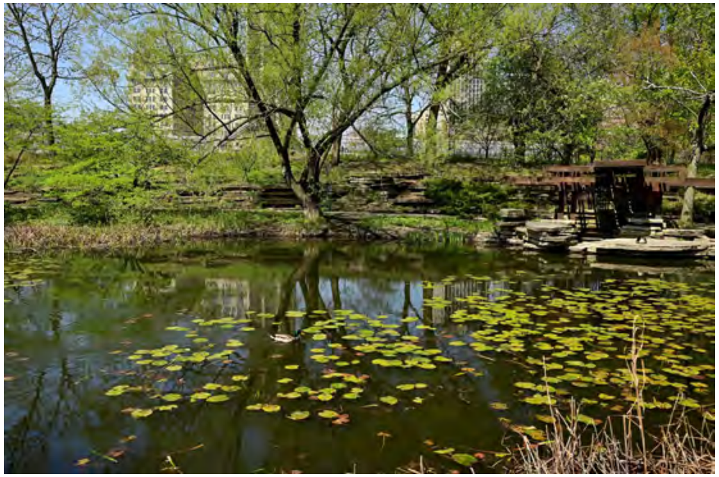

c. Bull-head pond-lily, Lily Pool Loop, Alfred Caldwell Lily Pool (12.11)

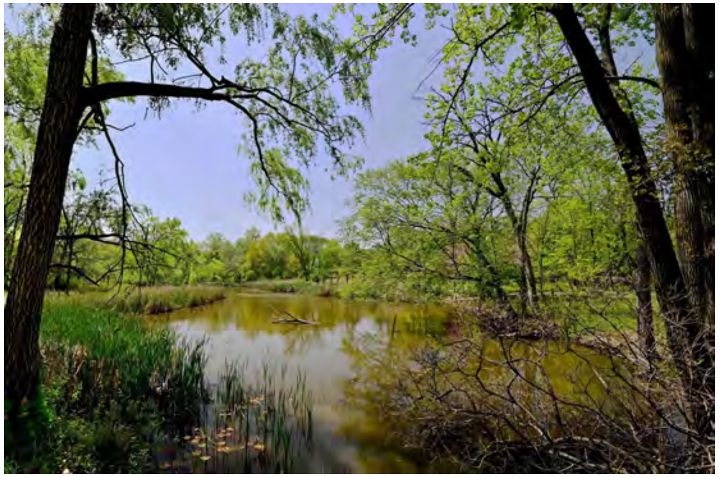

d. Cattail, Wetland Loop, North Park Village Nature Center (9.11)

3.46. Rock

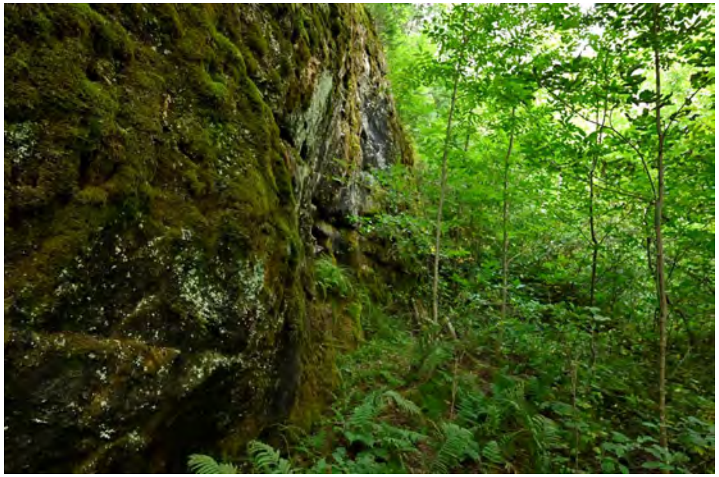

a. Cliffs-River Loop, Brule River Cliffs (5.12)

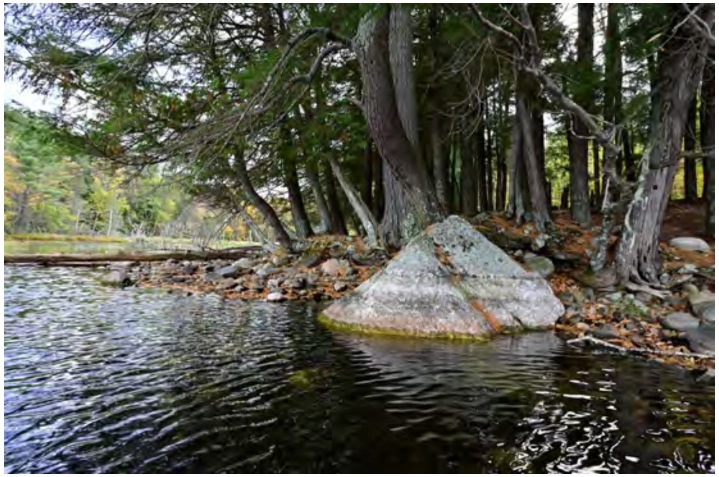

b. Lost Lake Paddle, Lost Lake Recreation Area (4.26)

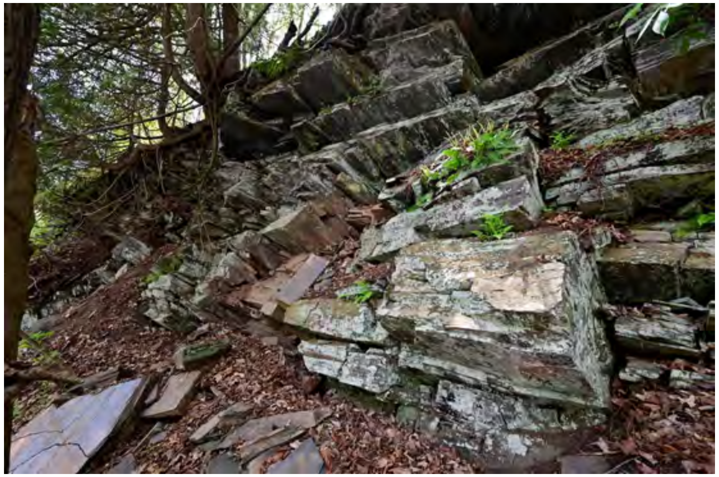

c. South Bank Trail, Breakwater Falls Pine River (1.62)

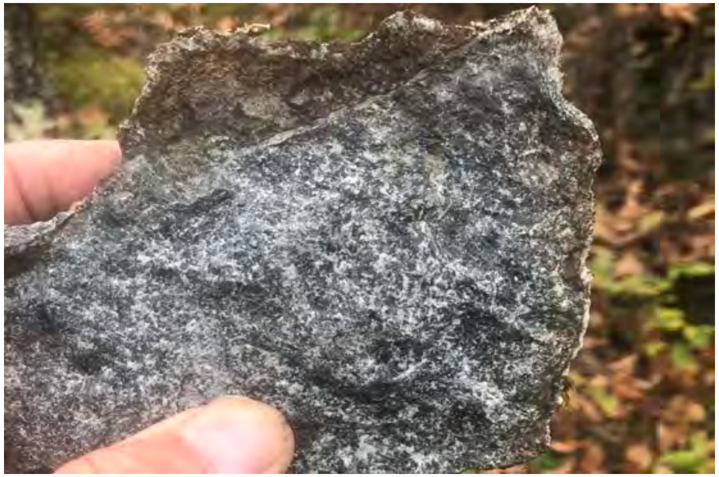

d. Outcrops-River Loop, Pine River Outcrops (1.31)

3.47. Moss and Lichens

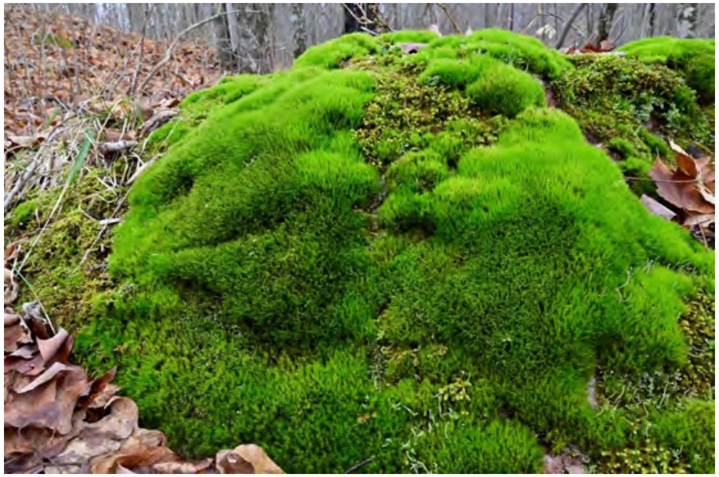

a. Broom Moss, Outcrops-River Loop, Pine River Outcrops (1.31)

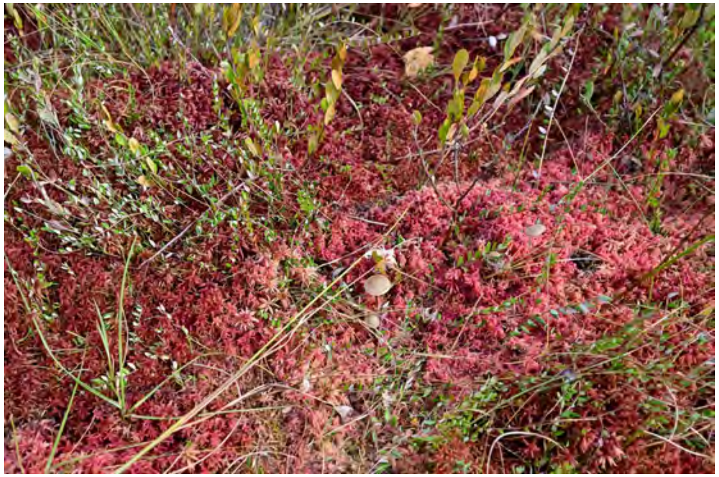

b. Sphagnum Moss, Two Sisters Lake Bog Walk, Hidden Lakes Dispersed Sites (6.23)

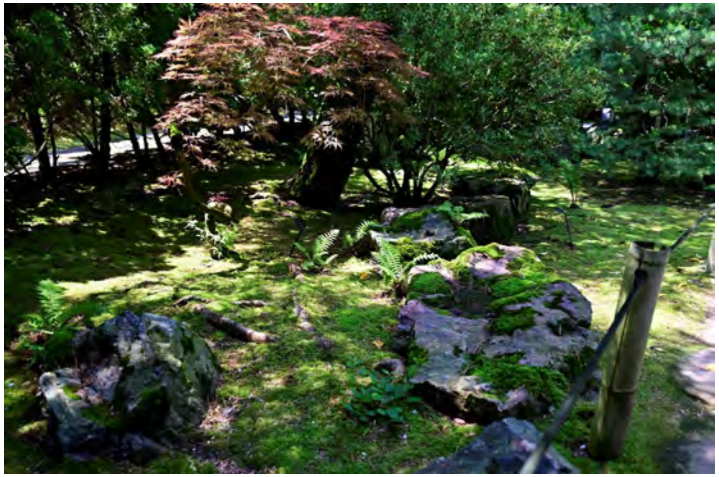

c. Fern Moss, Japanese Garden, Chicago Botanic Garden (15.24)

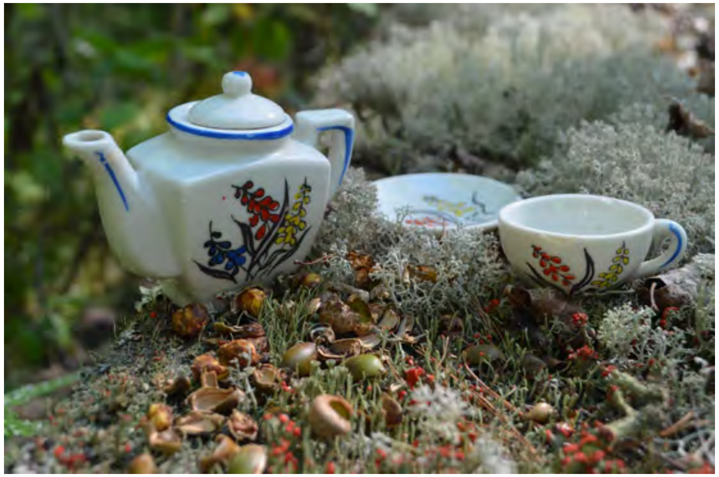

d. “Teddy Bears’ Picnic,” still life with various lichens, acorn shells, and mini tea set on old picnic table, undisclosed location, Northwoods study area.

3.48. Fungi

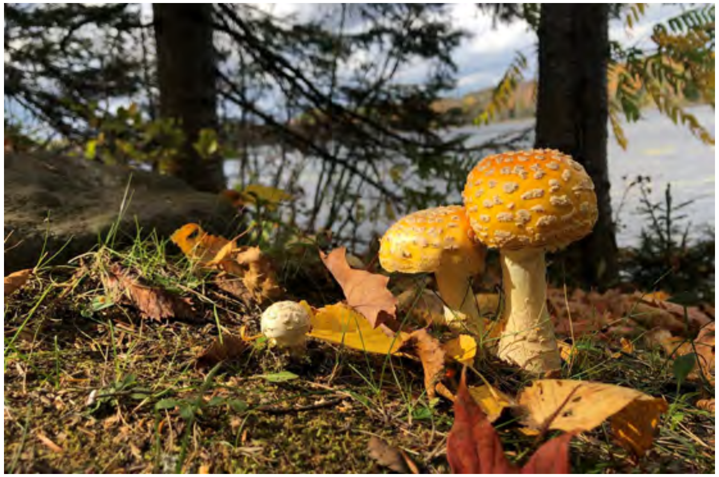

a. Amanita muscaria, Loop Trail North Portion , Perch Lake (3.21)

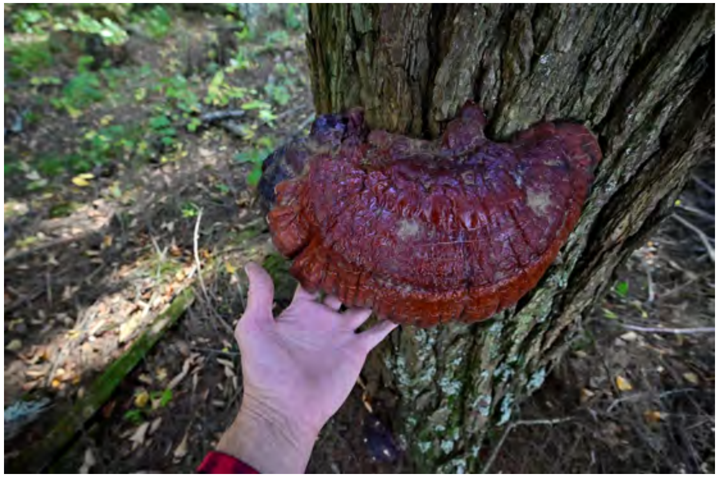

b. Red Reishi Ganoderma, Savage Lake Shoreline Walk, Savage-Robago Wild Lakes Complex (1.81)

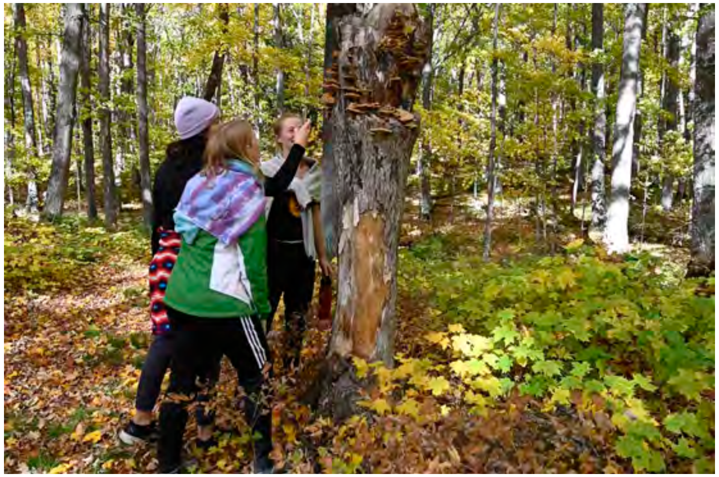

c. Ganoderma, Lauterman Lake Trail Loop, Lauterman National Recreation Trail (4.12)

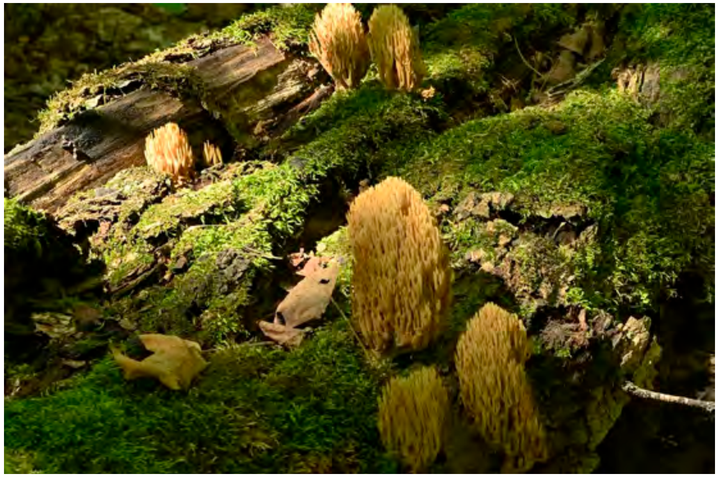

d. Coral fungus, Upland Loop, Fox Maple Woods (3.13)

4. Built and Borrowed Features

4.1. *Seating-* This sequence provides examples of various types of seating found within the two study areas for individuals and groups along a spectrum, from unmodified logs borrowed directly from nature to highly manufactured seating of plastic and metal (4.11–4.15). While natural materials are preferred in undeveloped settings, they should also be functional and comfortable, at least for short periods of sitting. In more developed settings more durable options and manufactured materials are acceptable but should be contextually compatible with respect to design, color, scale, and other characteristics.

4.2. *Gateways-* Gateways serve as important markers, signaling visitors to slow down and leave the busy world behind as they begin their forest therapy experience. Gateways can be symbolic markers such as distinctive trees or rocks near a trailhead (4.21) or physical markers such as entry signs (4.22), gates and selective barriers (4.23), or thresholds such as doorways, bridges, and low branches (4.24) that force the visitor to slow down, climb, or duck to enter. As with seating above, principles of contextual compatibility to the setting apply.

4.3. *Shelter-* Shelter can be an important built feature for forest therapy for a number of reasons, to escape the weather, for groups to gather before or after a walk, to spend the night for an extended visit on or near a trail, or for other reasons including symbolic or nature play. Examples of shelter in this sequence include borrowed and built natural elements from shade trees to tipis and wigwams (4.13) that provide limited or symbolic and play functions, to open air (4.32) and enclosed (4.33) structures of rustic, historic, and contemporary design. While examples in the two study areas were somewhat limited, they illustrate a range of possibilities and how they fit contextually to different trail settings.

4.4. *Bridges and Boardwalks-* Bridges and boardwalks serve important functional purposes to extend trails across water, wet, or sensitive terrain, but as built features can also enhance the experience forest therapy walks by their design and alignment (see also Alignment above). This sequence illustrates a spectrum of bridges and boardwalks from the use of unmodified natural materials such as branches and logs (4.41) to manufactured wood planks and bridges (4.42) to highly manufactured concrete, metal, and plastic structures (4.43).

4.5. *Miscellaneous Features-* This final sequence illustrates a variety of other built features including firepits and fireplaces (4.51), sculptures (4.52), natural play areas (4.53), observation structures (4.54,4.55), and drinking water wells and fountains (4.56). The examples show how such features may be introduced across a spectrum of settings from primitive and rustic to modern.

4.11. Seating– Logs and Raised Logs

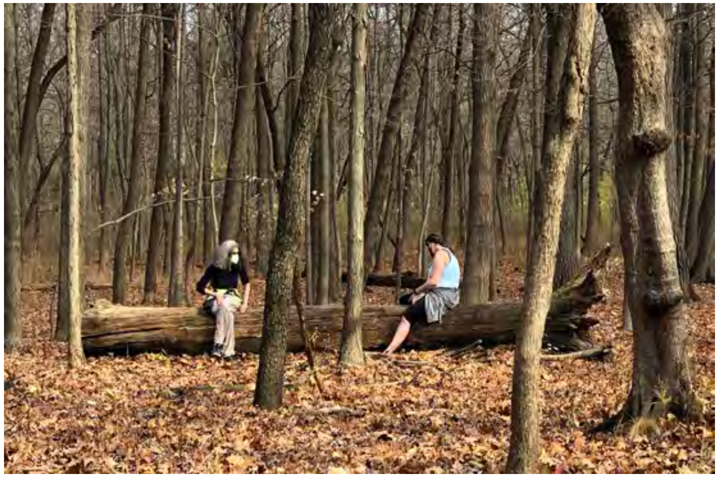

a. Full Loop, Harms Woods Nature Preserve (14.14)

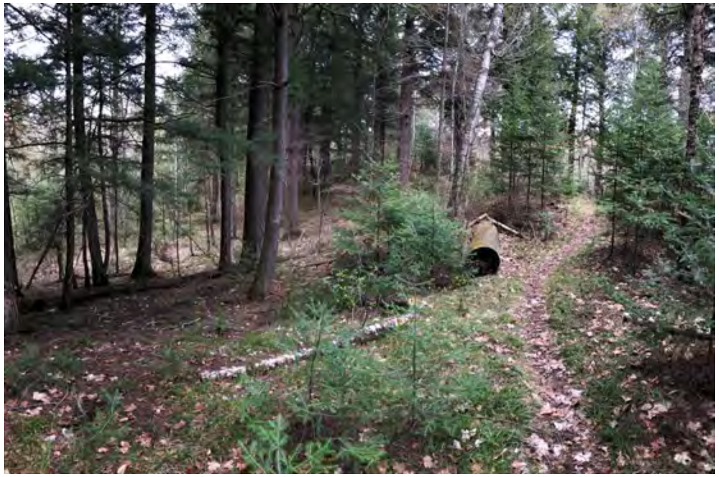

b. Lakeshore–Ridge Trail Loop, Lost Lake Recreation Area (4.23)

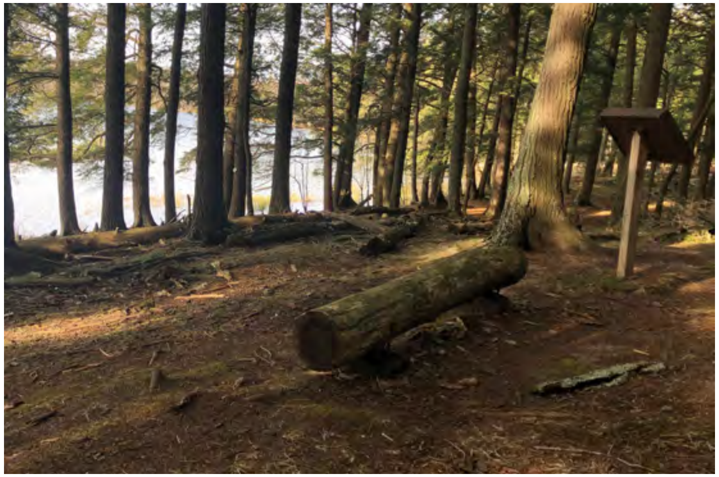

c. Assessor's Interpretive Trail Loop, Lost Lake Recreation Area (4.22)

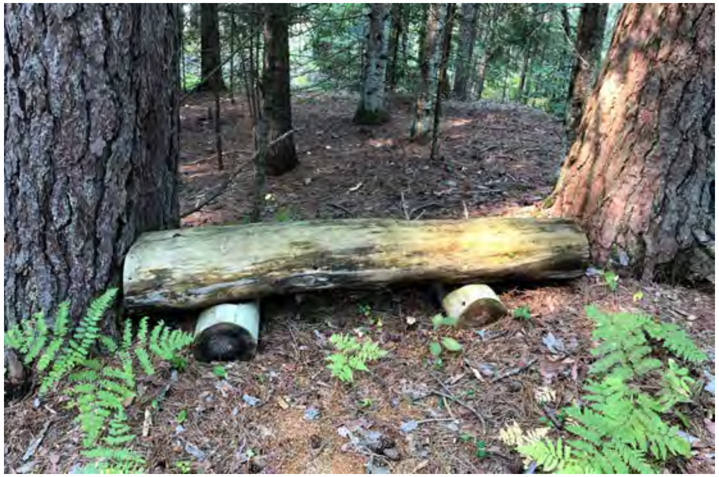

d. Healing Nature Trail (6.31)

4.12. Seating– Split Logs and Slabs

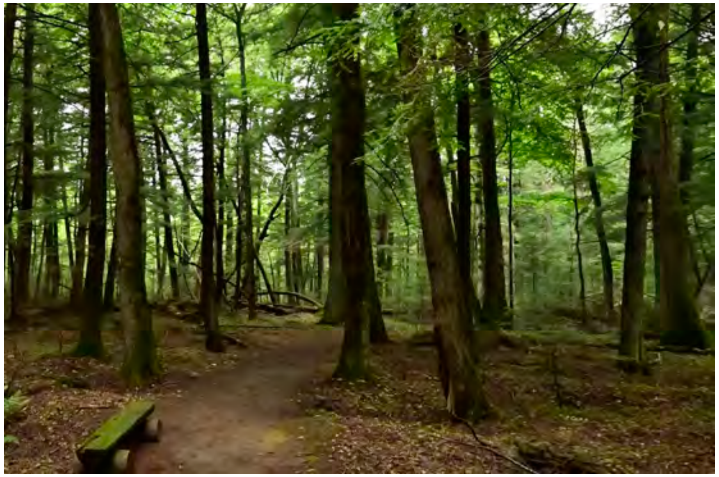

a. Franklin Nature Trail, Hidden Lakes Trail (6.11)

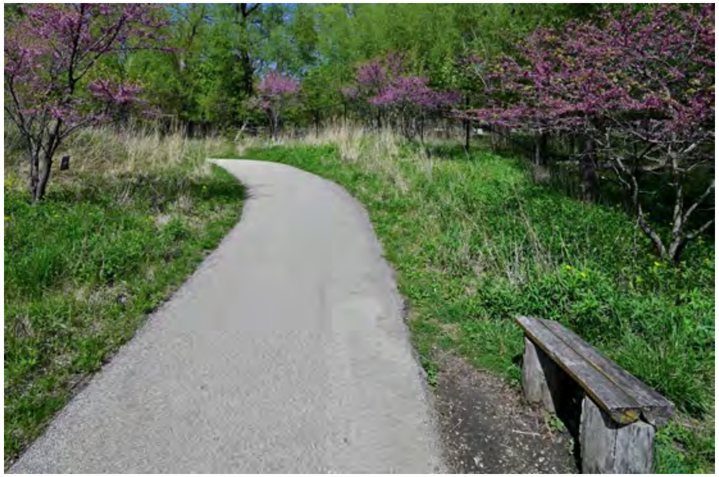

b. Lake Loop, West Ridge Nature Park (9.22)

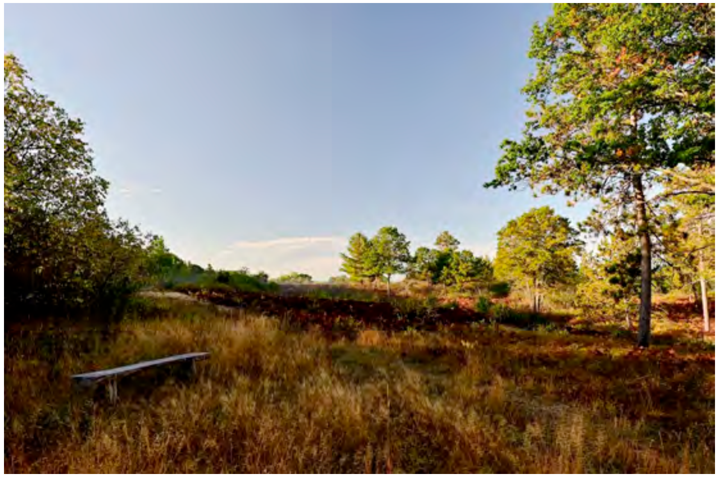

c. Short Loop, Barrens Lake (7.21)

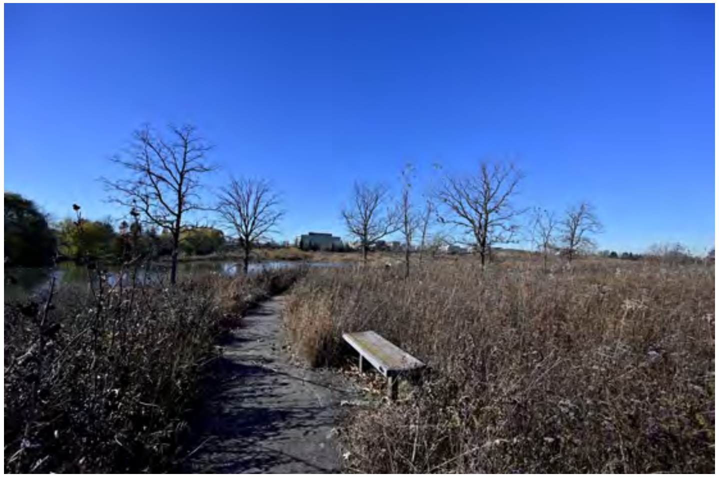

d. Dixon Prairie, Chicago Botanic Garden (15.25)

4.13. Seating– Backed Benches

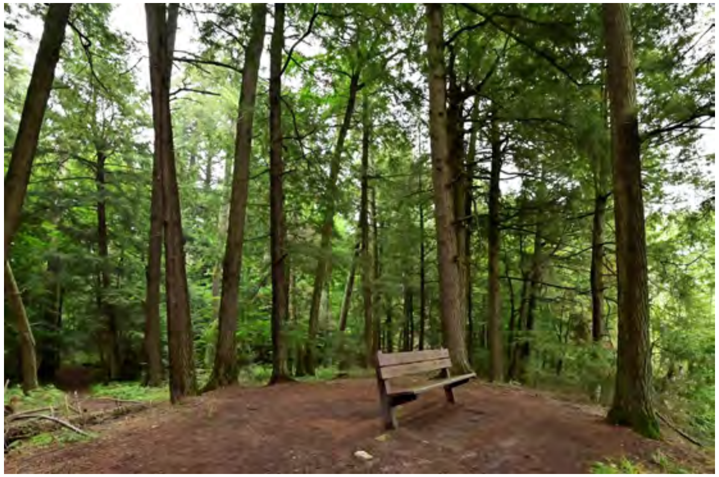

a. Franklin Nature Trail, Hidden Lakes Trail (6.11)

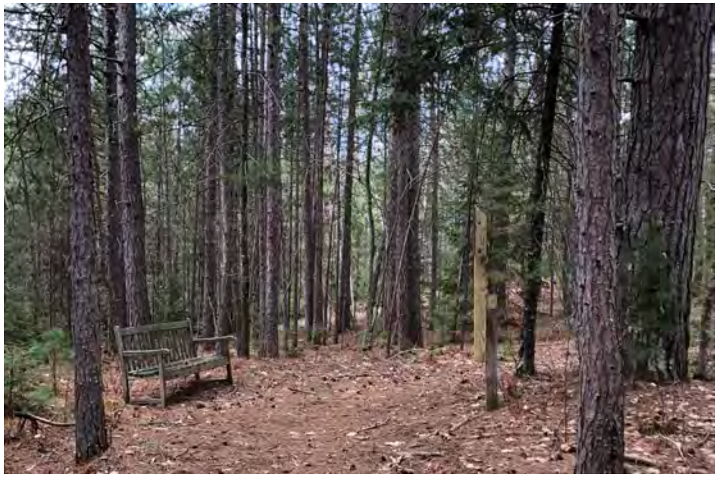

b. Return of Trees Trail, Wild Rivers Interpretive Center (1.11)

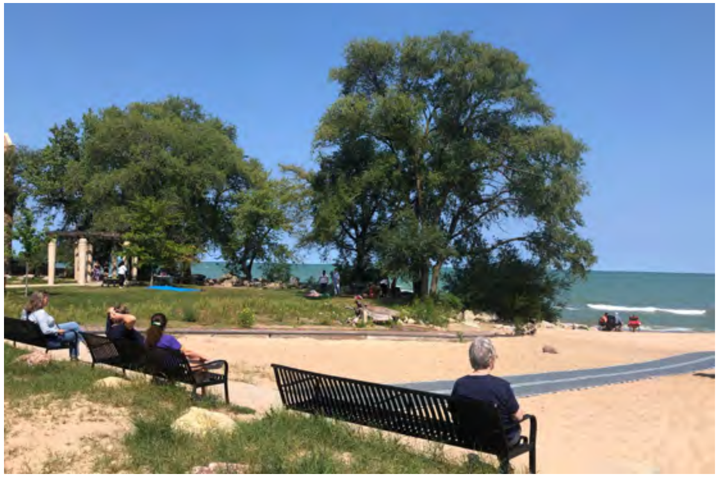

c. Loyola Natural Area-Park Loop, Loyola-Leone Parks (10.21)

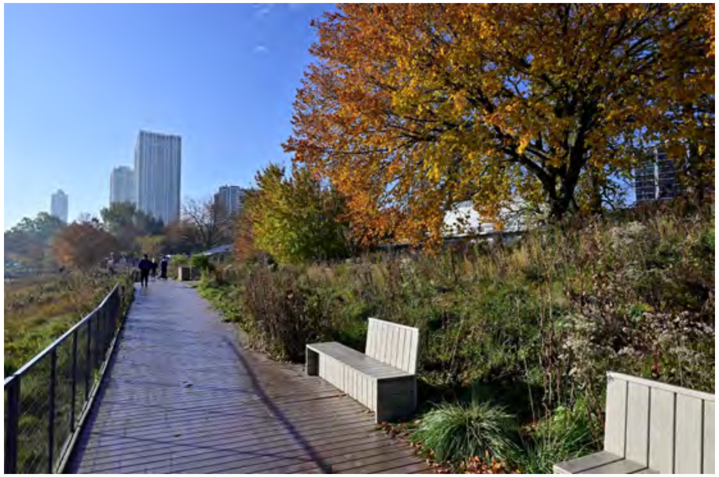

d. Nature Boardwalk, Lincoln Park Zoo (12.41)

4.14. Seating– Small Group/Conversational

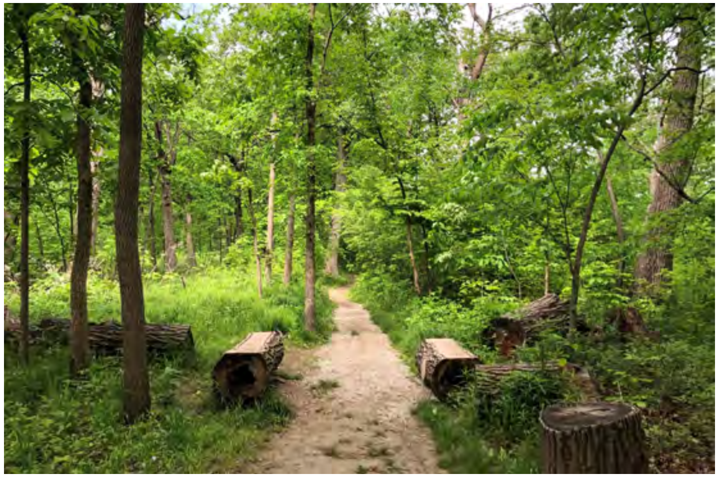

a. Woodland–River West Loop, Harms Woods Nature Preserve (14.11)

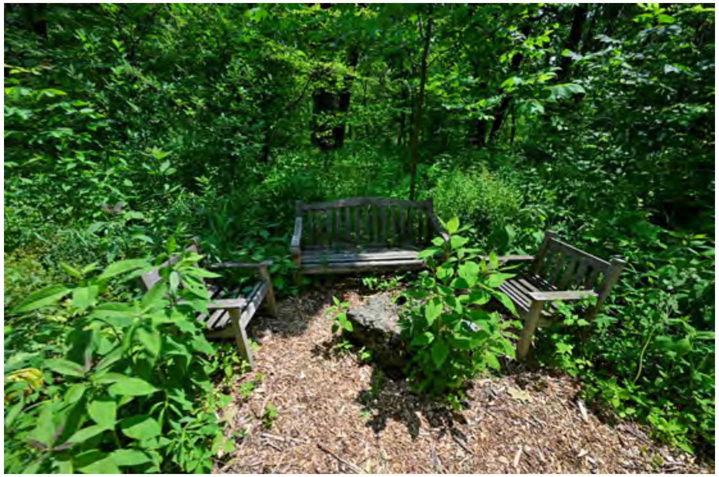

b. McDonald Woods Big Loop, Chicago Botanic Garden (15.23)

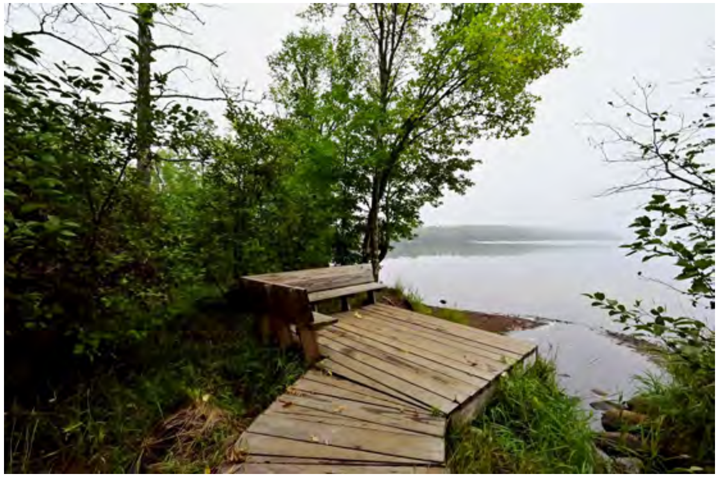

c. Franklin Nature Trail, Hidden Lakes Trail (6.11)

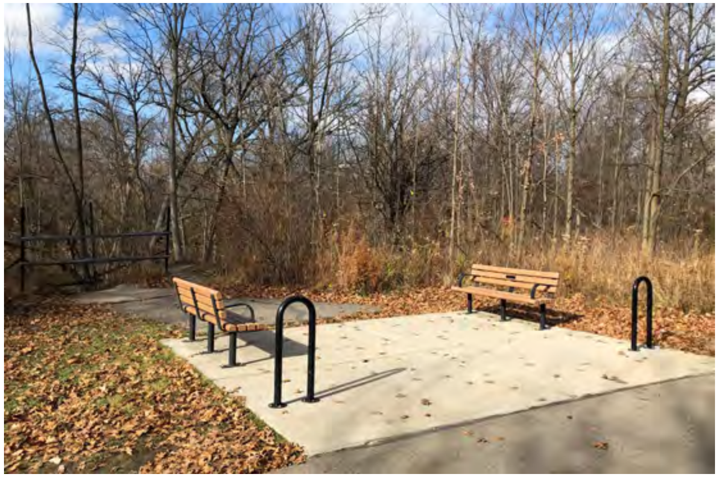

d. Savanna–Flatwoods–River Loop, Sidney Yates Flatwoods (13.12)

4.15. Seating– Group and Council Rings

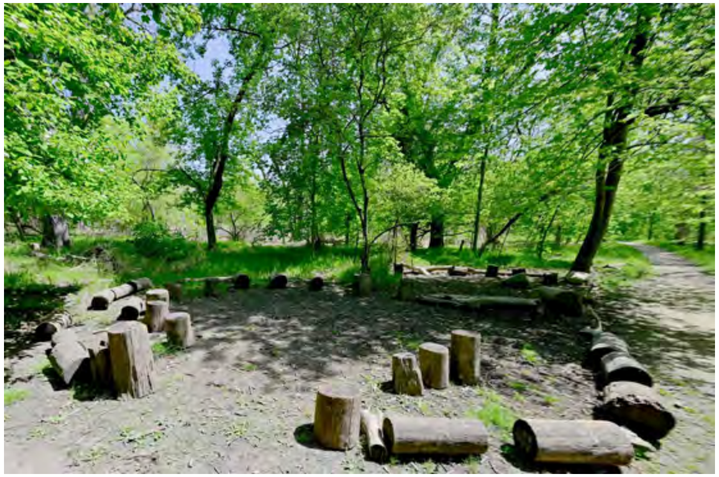

a. Woodland Loop, North Park Village Nature Center (9.12)

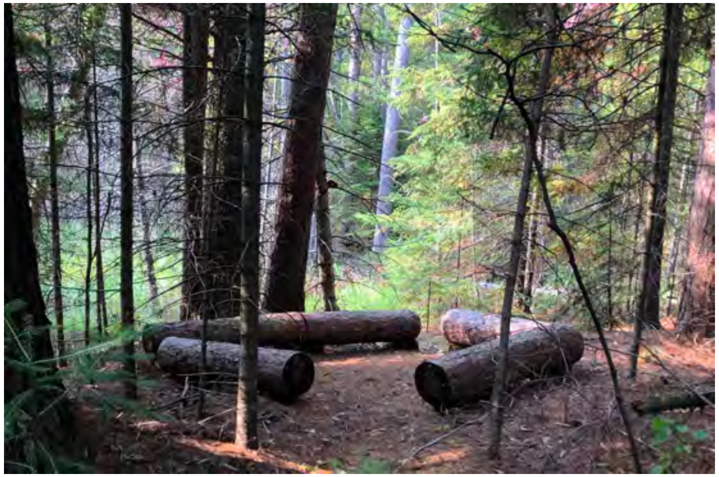

b. Healing Nature Trail (6.31)

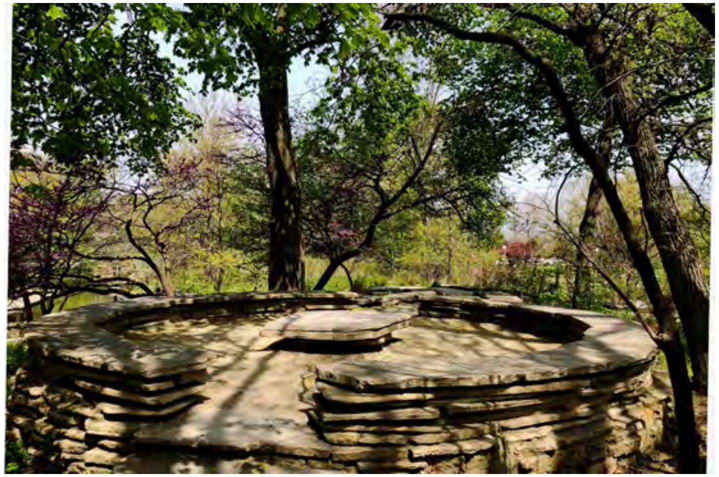

c. Lily Pool Loop, Alfred Calwell Lily Pool (12.11)

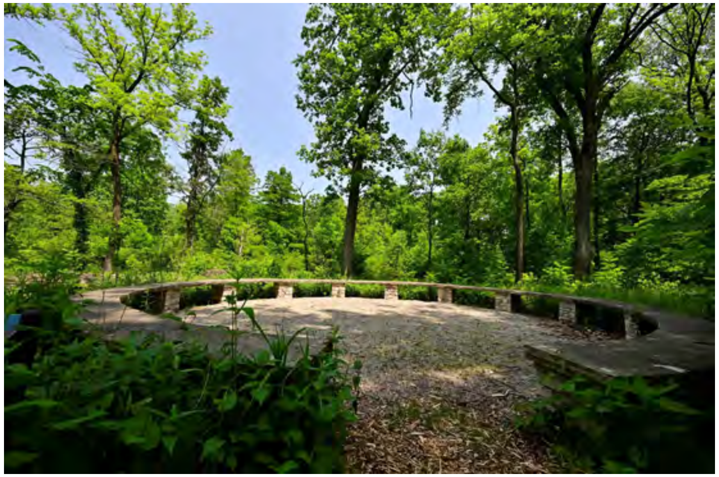

d. McDonald Woods South Loop, Chicago Botanic Garden (15.21)

4.21. Gateways– Natural & Culturally Modified Natural Features

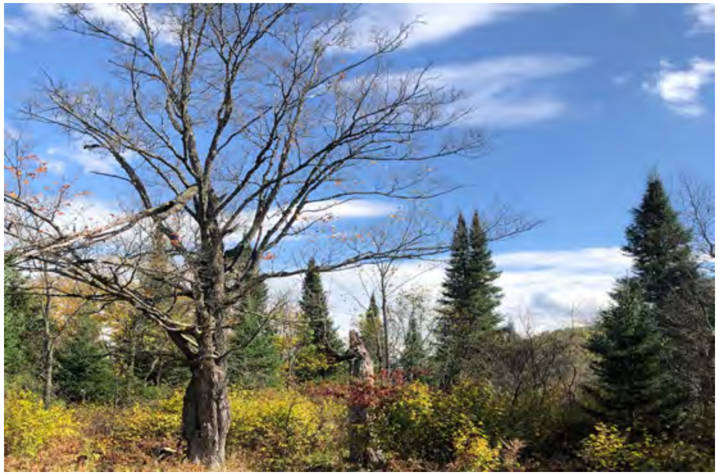

a. Cliffs-River Loop, Brule River Cliffs (5.12)

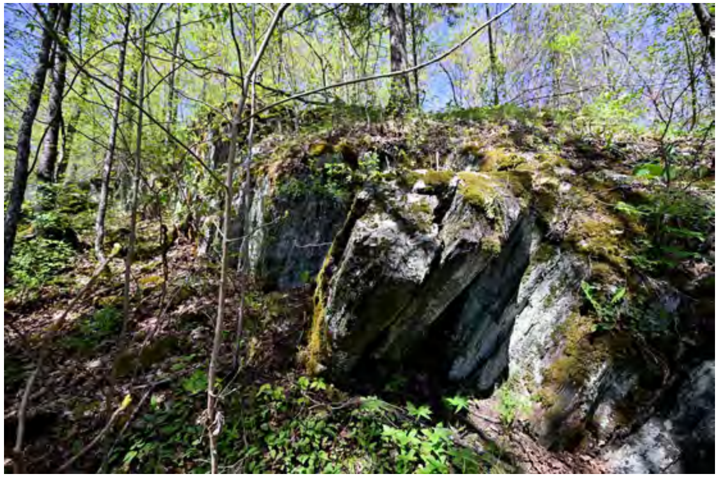

b. Outcrops-Western Trail, Pine River Outcrops (1.32)

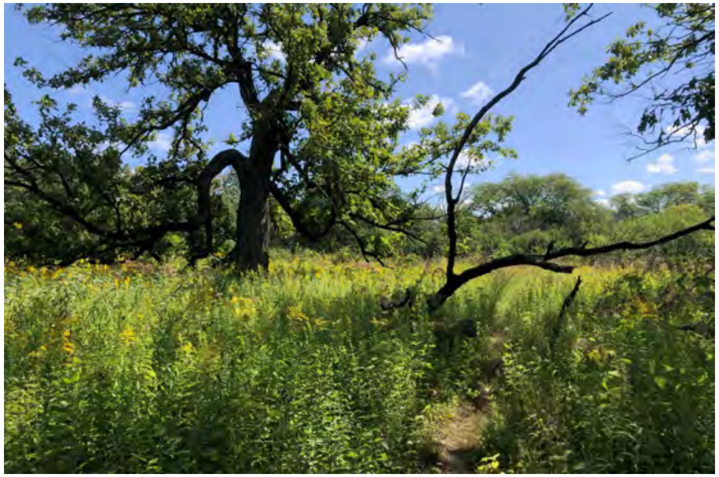

c. Vestal Grove-Savanna Loop, Somme Prairie Grove (16.21)

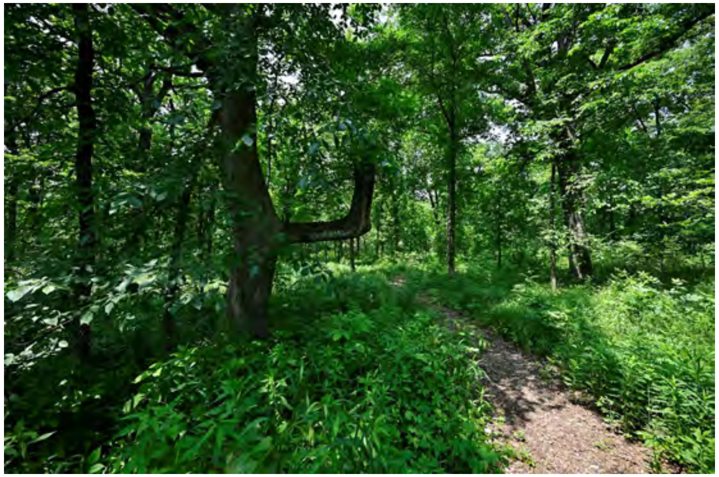

d. McDonald Woods North Loop, Chicago Botanic Garden (15.22)

4.22. Gateways– Entry Signs

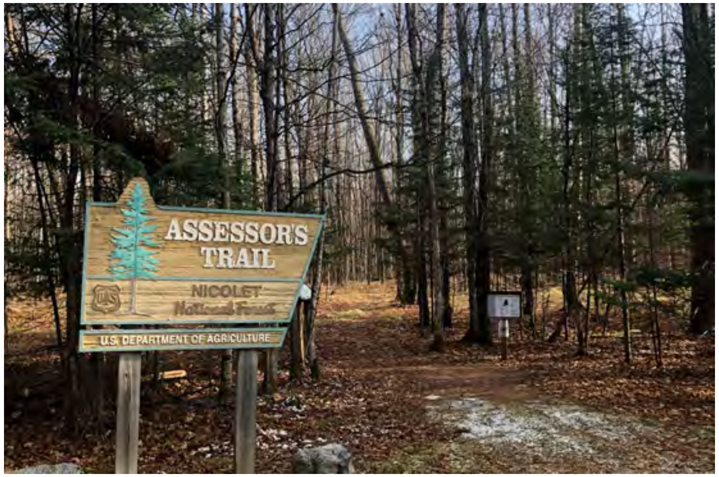

a. Assessor's Trail, Lost Lake Recreation Area (4.22)

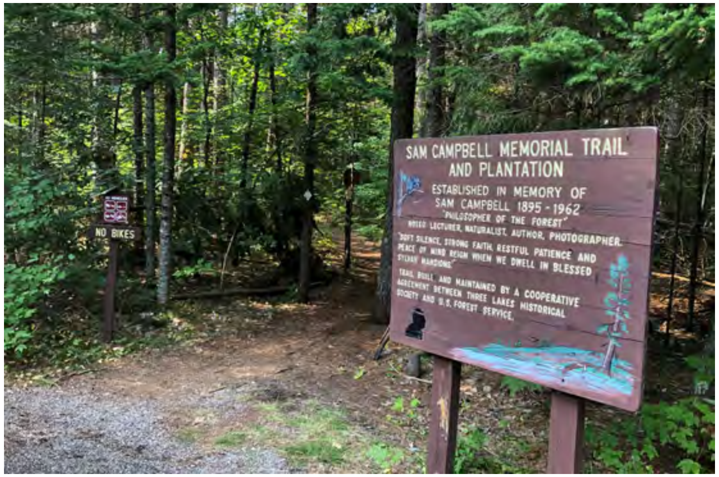

b. Sam Campbell Memorial Trail (6.32)

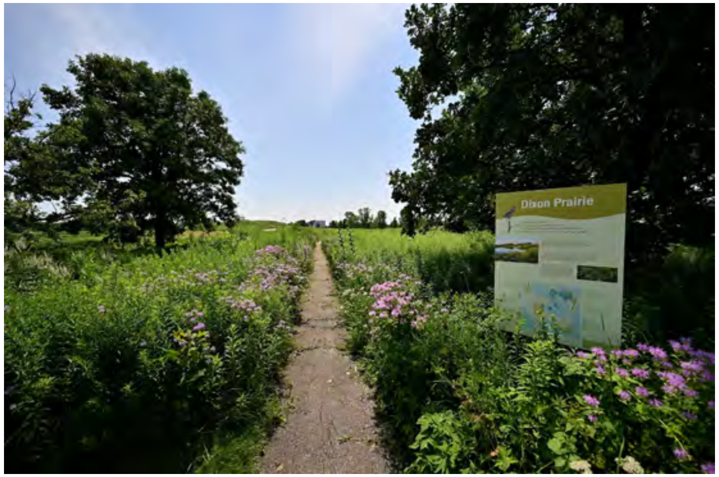

c. Dixon Prairie, Chicago Botanic Garden (15.25)

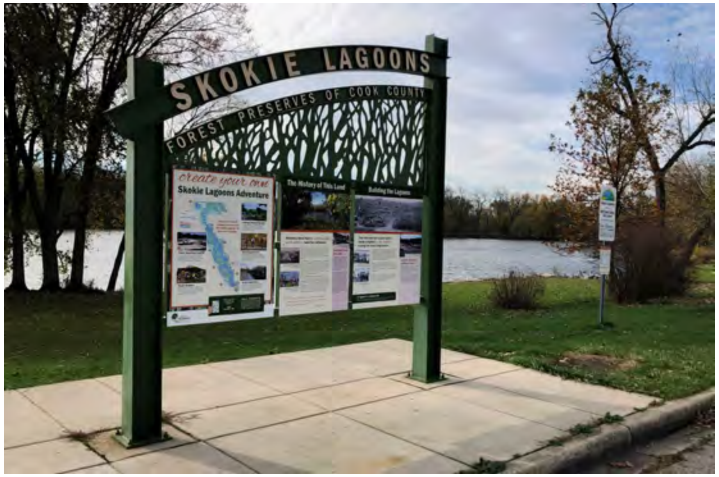

d. Skokie Lagoons Forest Preserve (15.1)

4.23. Gateways– Gates and Selective Barriers

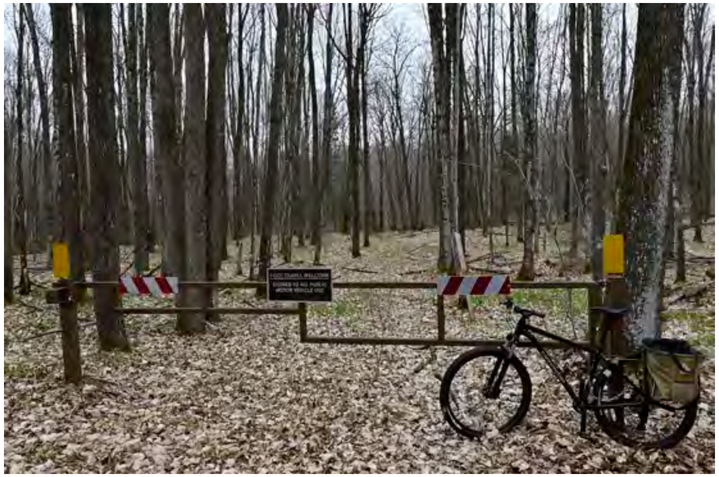

a. West Trail, Rainbow Hunter Walking Trails (3.54)

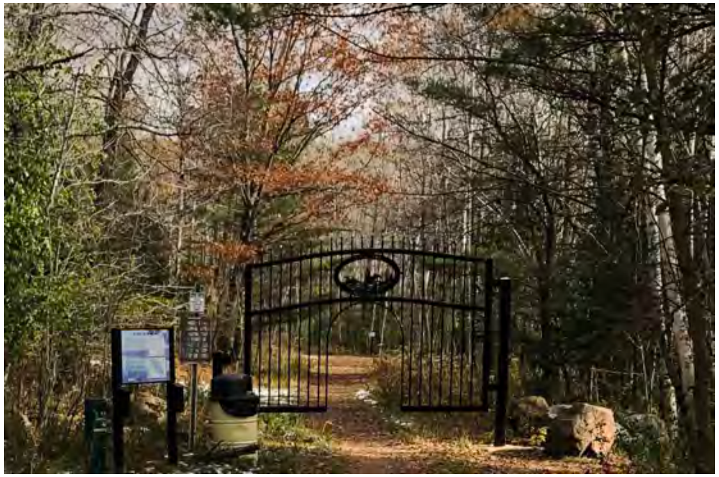

b. Fumee Lake Trails (8.2)

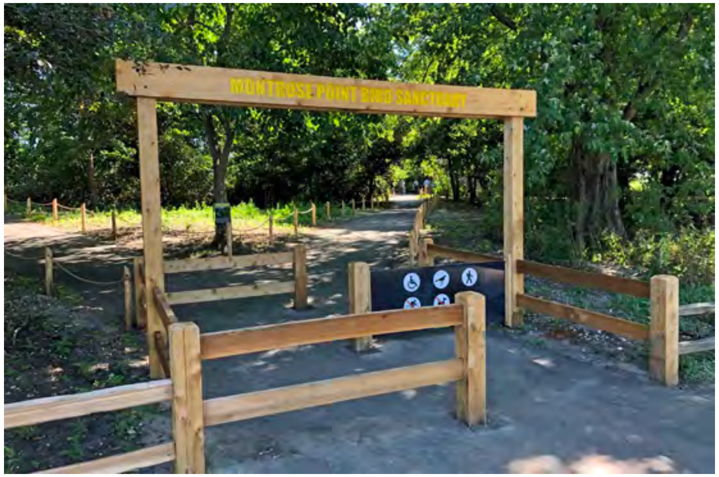

c. Bird Sanctuary Main Loop, Montrose Point (11.11)

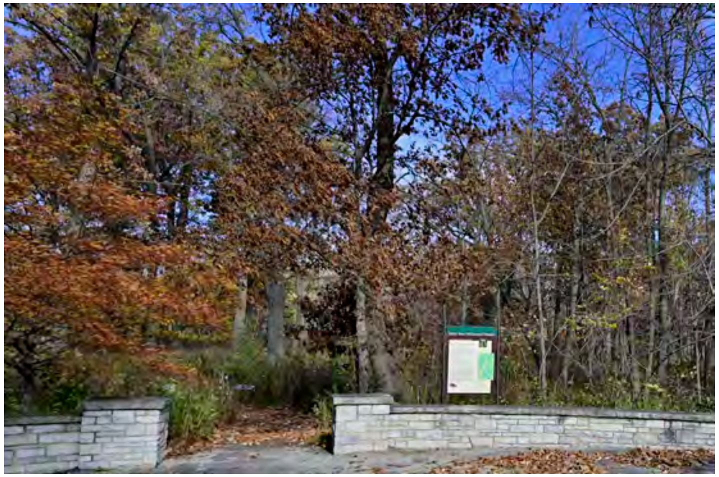

d. McDonald Woods South Loop, Chicago Botanic Garden (15.21)

4.24. Gateways– Thresholds

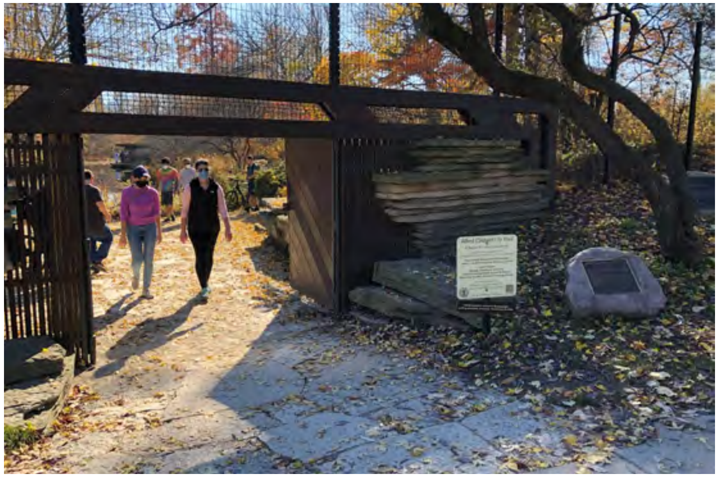

a. Lily Pool Loop, Alfred Caldwell Lily Pool (12.11)

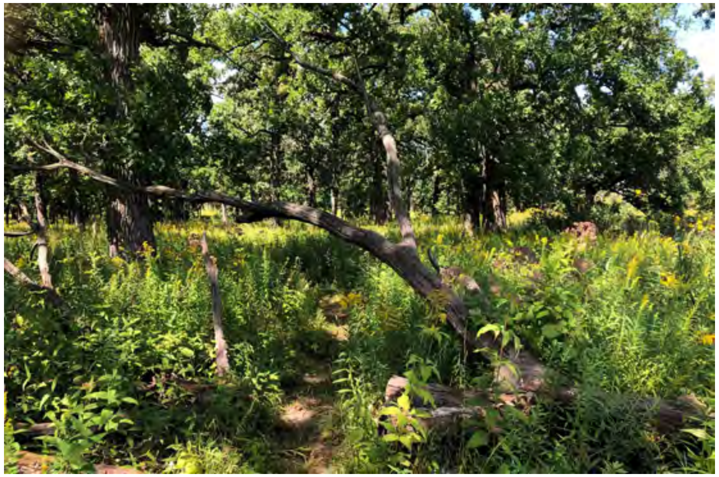

b. Vestal Grove Savanna Loop, Somme Prairie Grove (16.21)

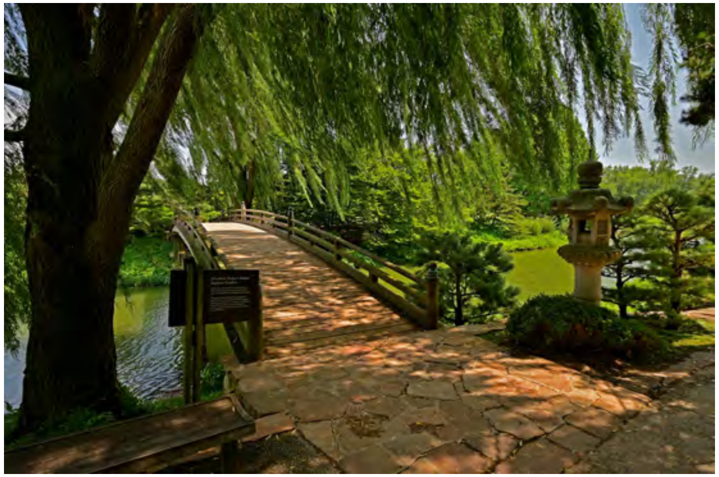

c. Japanese Garden, Chicago Botanic Garden (15.24)

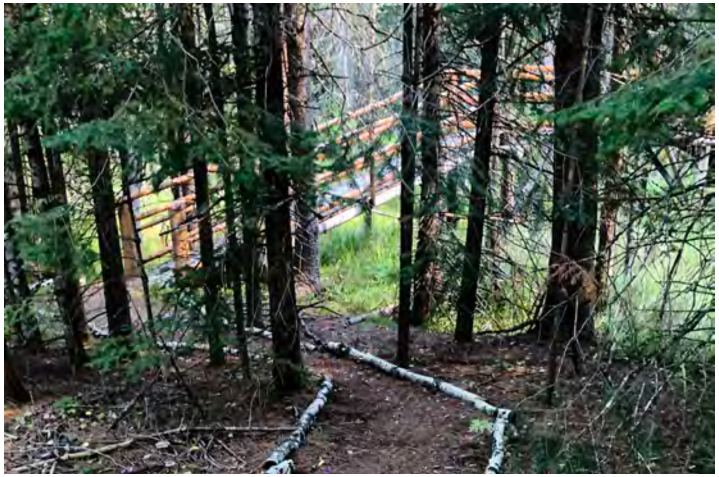

d. Healing Nature Trail (6.31)

4.31. Shelter– Nature Borrowed and Built

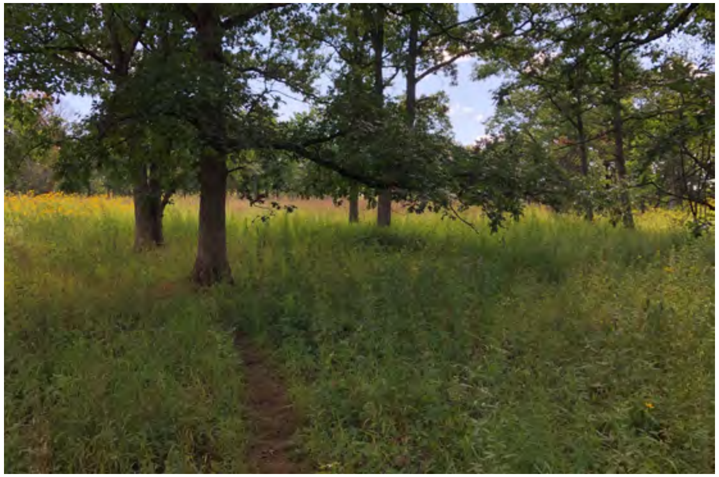

a. Prairie–Grove Loop, Somme Prairie Grove (16.22)

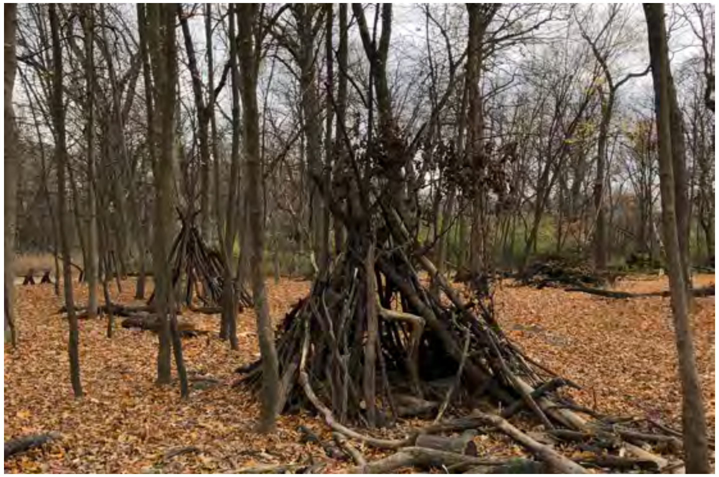

c. Woodland Loop, North Park Village Nature Center (9.12)

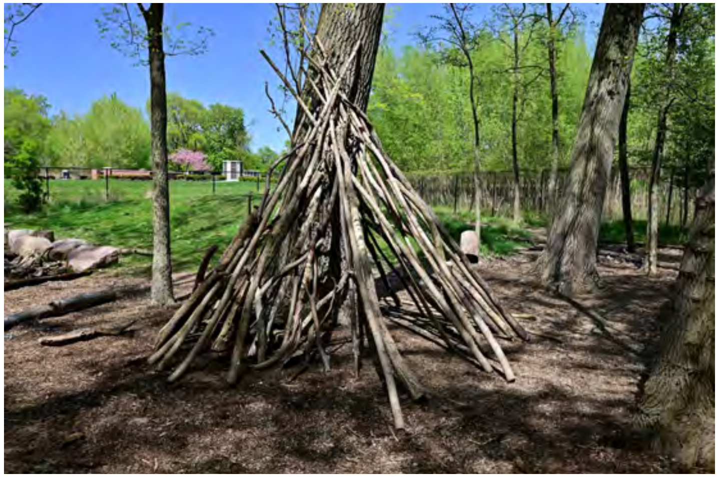

c. Woodland Loop, West Ridge Nature Park (9.21)

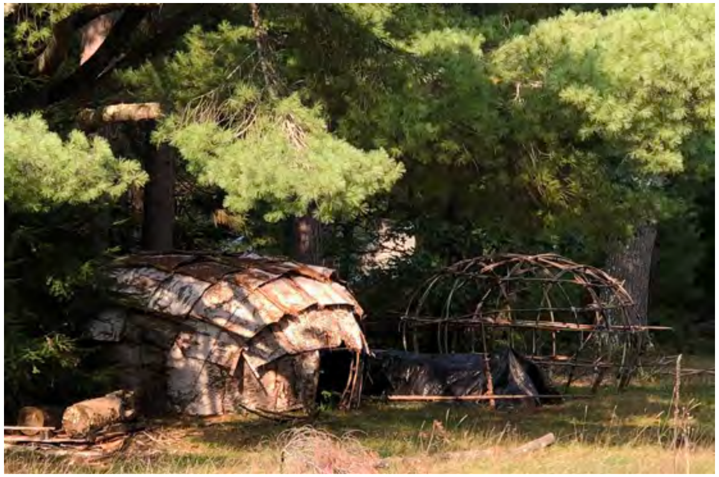

d. Healing Nature Trail (6.31)

4.32. Shelter– Open Air

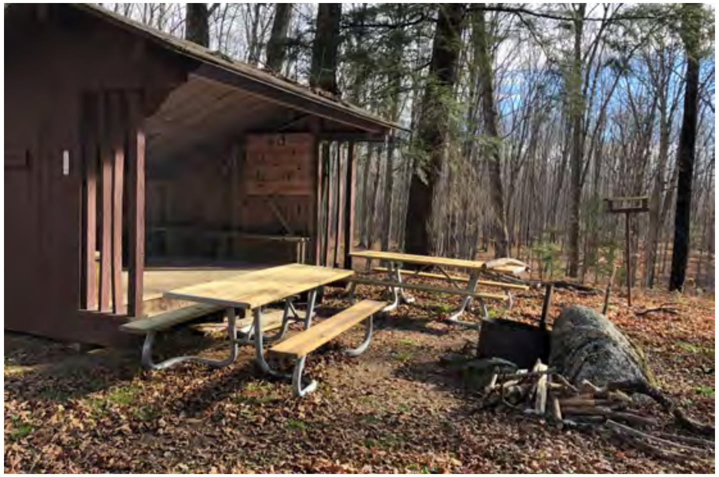

a. Lauterman Lake Trail Loop, Lauterman National Recreation Trail (4.12)

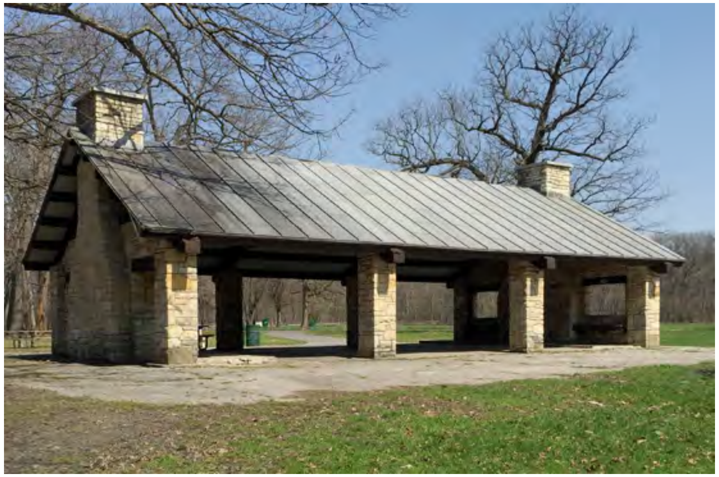

b. Bike Loop, Harms Woods Nature Preserve (14.22)

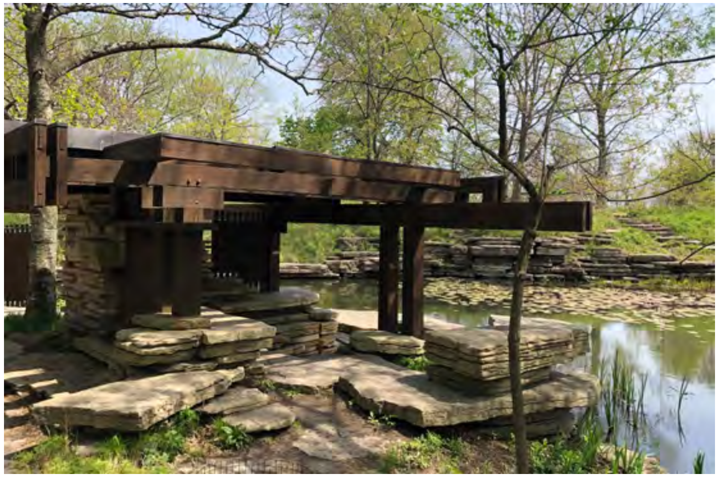

c. Lily Pool Loop, Alfred Caldwell Lily Pool (12.11)

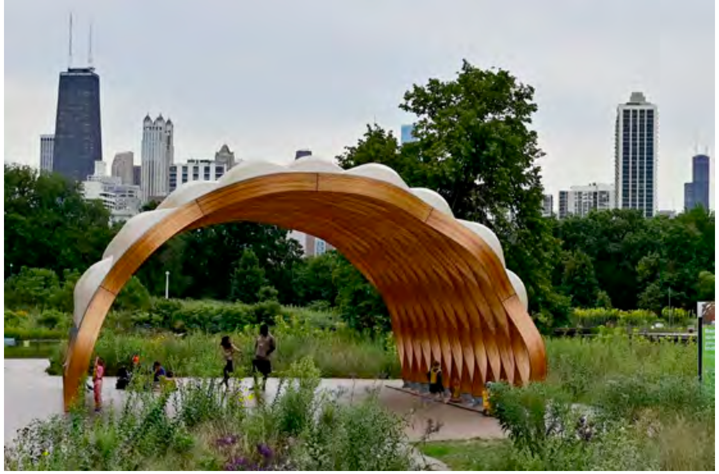

d. Nature Boardwalk, Lincoln Park Zoo (12.41)

4.33. Shelter– Enclosed

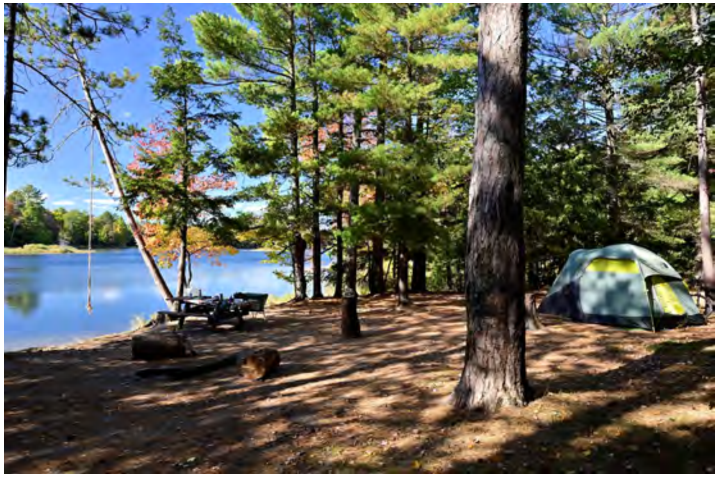

a. Three Johns Lake, Hidden Lakes Dispersed Sites (6.21)

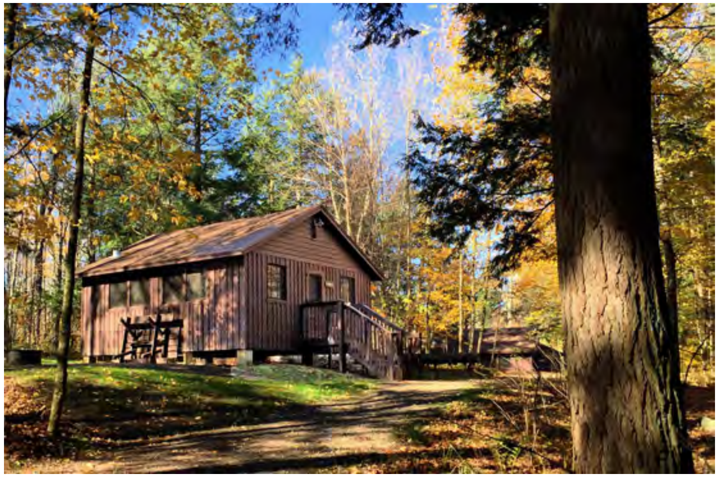

b. CCC Cabins to Ridge Trail Loop, Lost Lake Recreation Area (4.24)

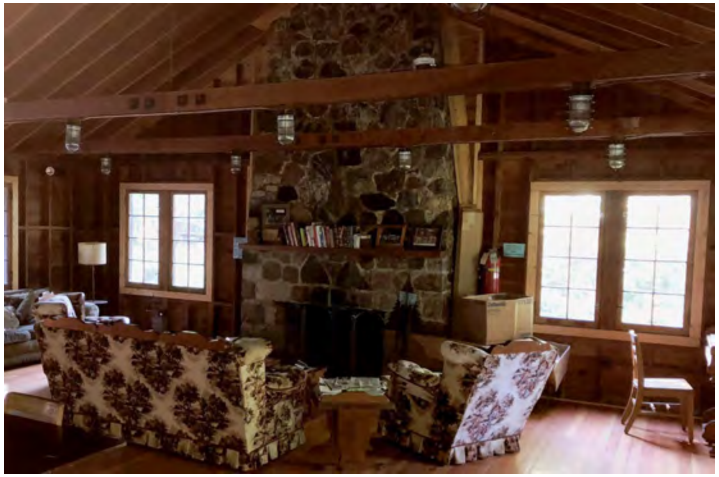

c. CCC Cabins to Ridge Trail Loop, Lost Lake Recreation Area (4.24)

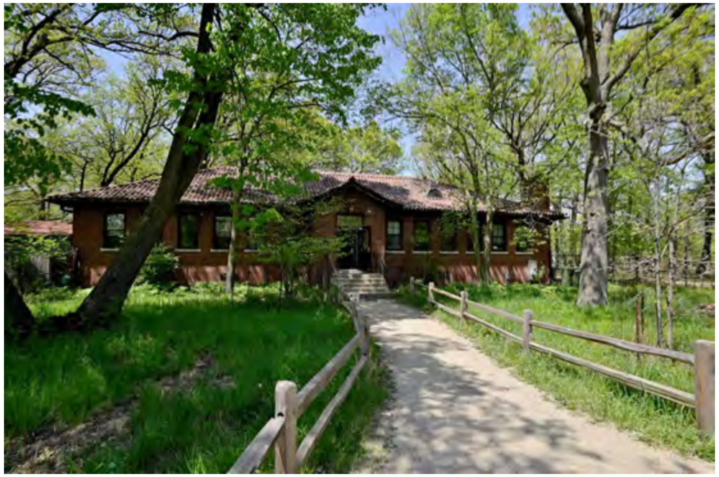

d. North Park Village Nature Center (9.1)

4.41. Bridges and Boardwalks– Unmodified Natural Materials

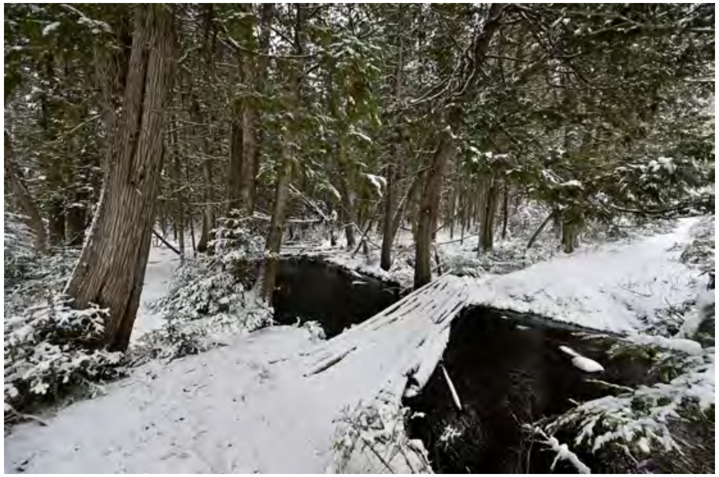

a. Whisker Lake Trail, Whisker Lake Wilderness (3.41)

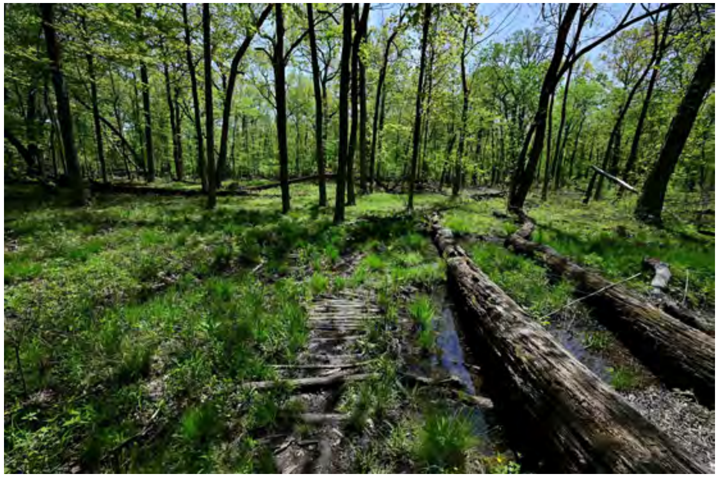

b. West Outer Loop, Somme Woods (16.32)

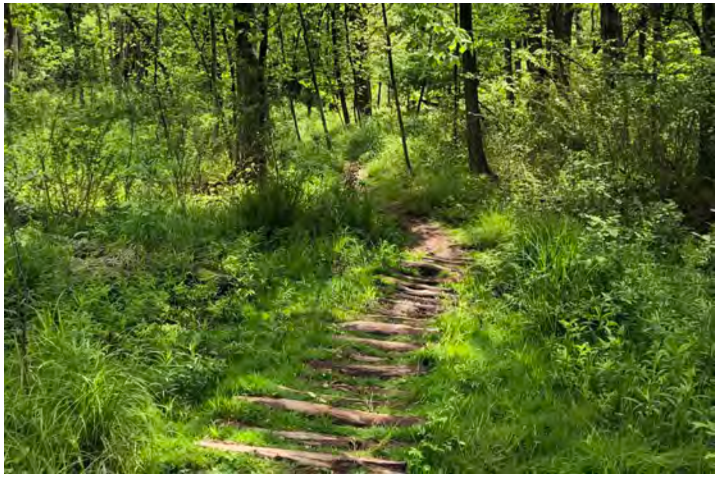

c. Woodland–Meadow Loop, Harms Woods Nature Preserve (14.13)

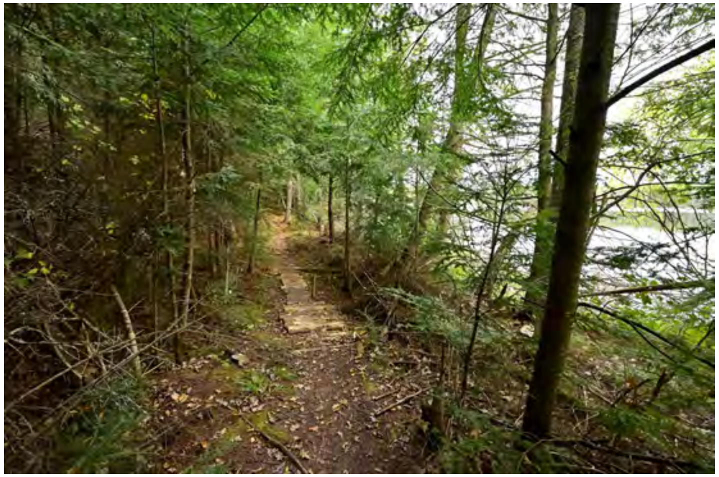

d. White Deer Trail, Hidden Lakes Trail (6.16)

4.42. Bridges and Boardwalks– Manufactured Natural Materials

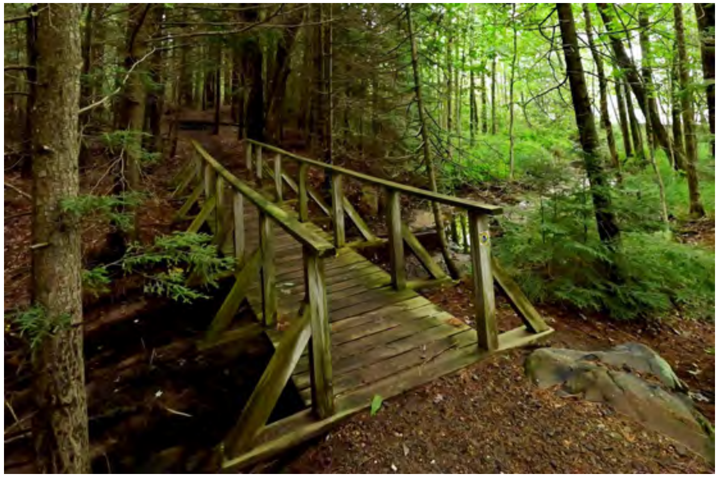
a. Franklin Nature Trail, Hidden Lakes Trail (6.11)

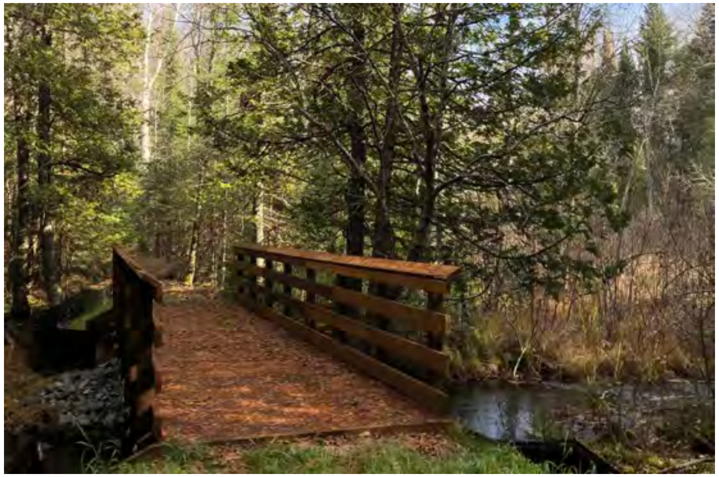
b. Lauterman Lake Trail Loop, Lauterman National Recreation Trail (4.12)

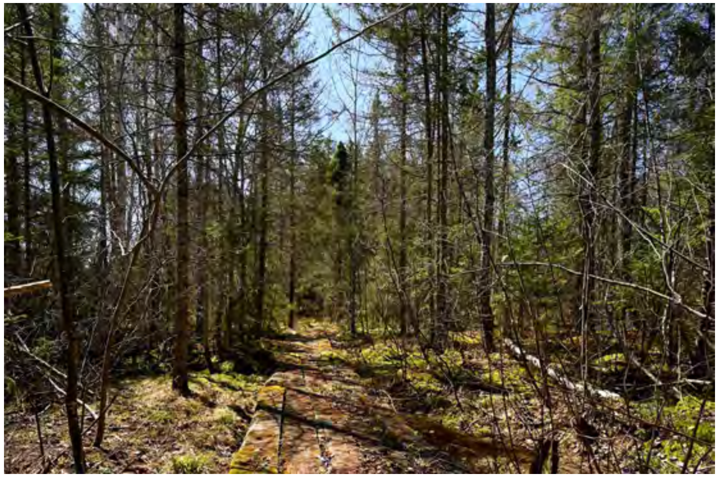
c. Old boardwalk, Lakeshore–Ridge Trail Loop, Lost Lake Recreation Area (4.23)

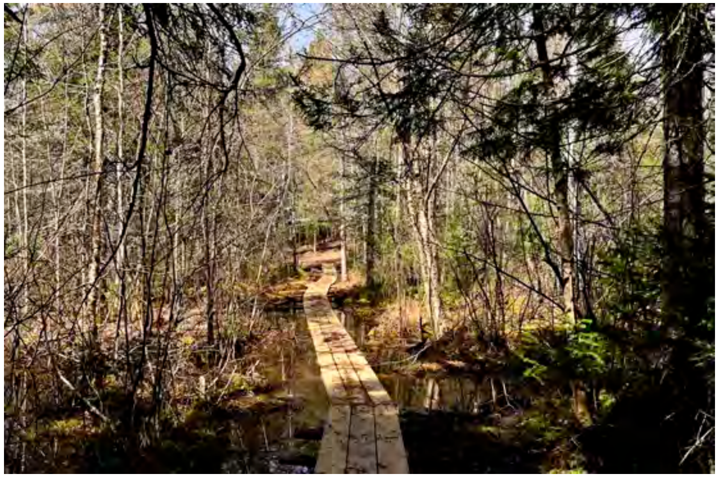
d. New boardwalk, Lakeshore–Ridge Trail Loop, Lost Lake Recreation Area (4.23)

4.43. Bridges and Boardwalks– Concrete, Metal and Plastic

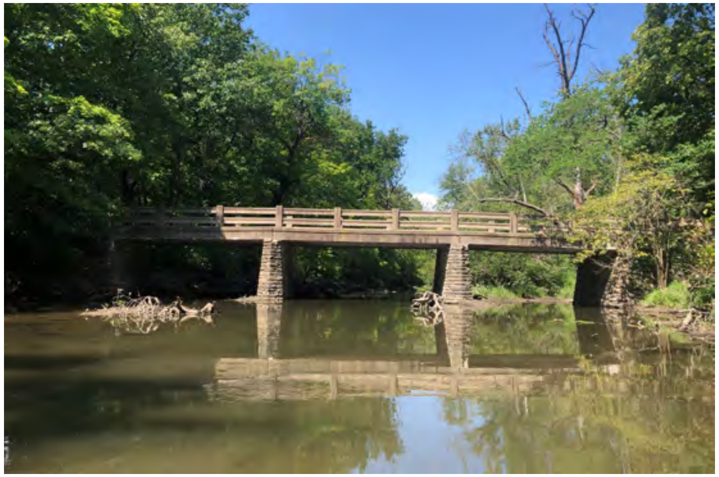

a. Chicago River Paddle, Harms Woods Nature Preserves (14.21)

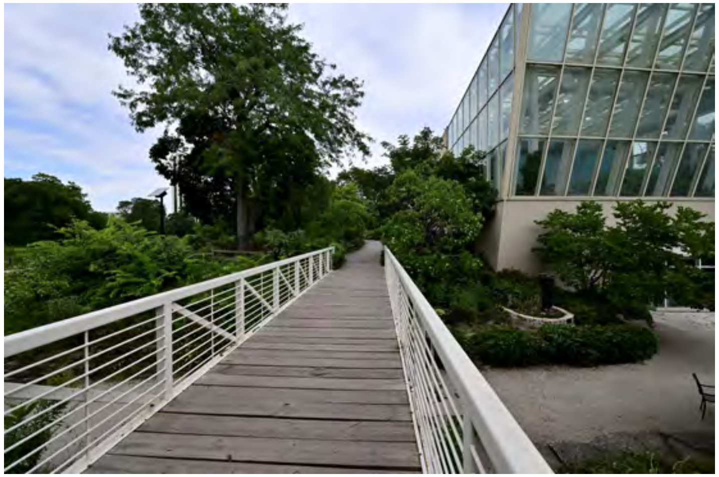

b. Deb Lahey Nature Trails, Nature Museum (12.21)

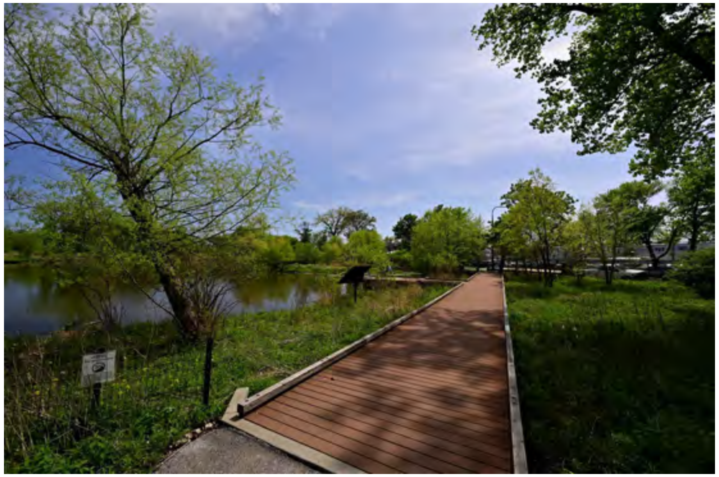

c. Lake Loop, West Ridge Nature Park (9.22)

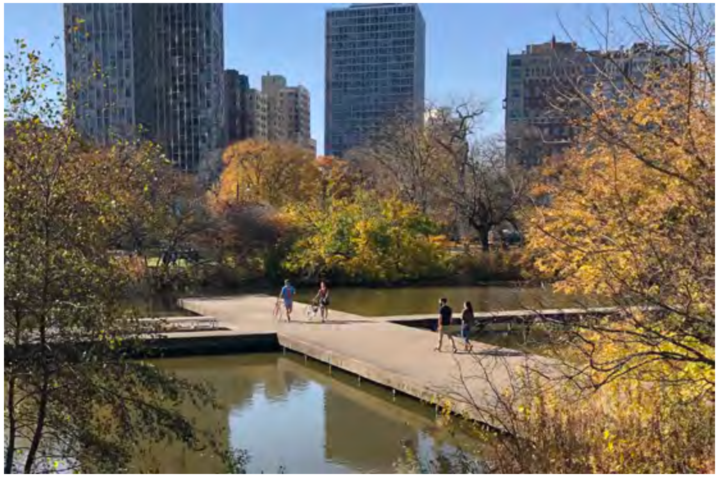

d. Casting Pier, Natural Area Loop, North Pond (12.31)

4.51. Firepits and Fireplaces

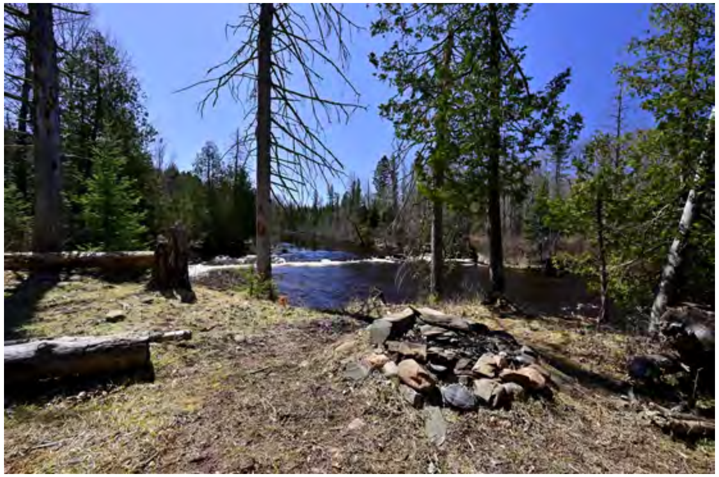

a. North Loop, Rainbow Hunter Walking Trails (3.51)

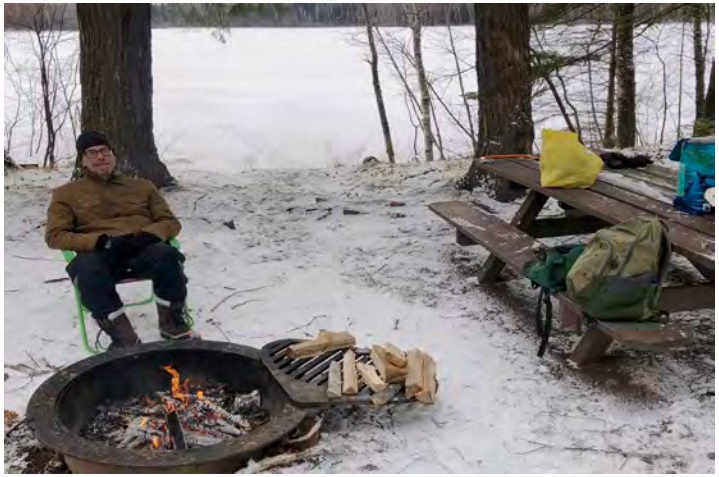

b. Full Loop Trail, Perch Lake (3.22)

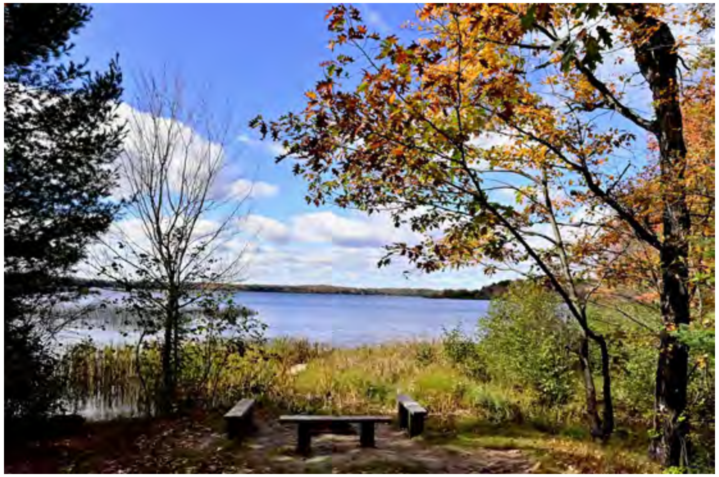

c. McKinley Lake–Luna Lake segment, Hidden Lakes Trail (6.13)

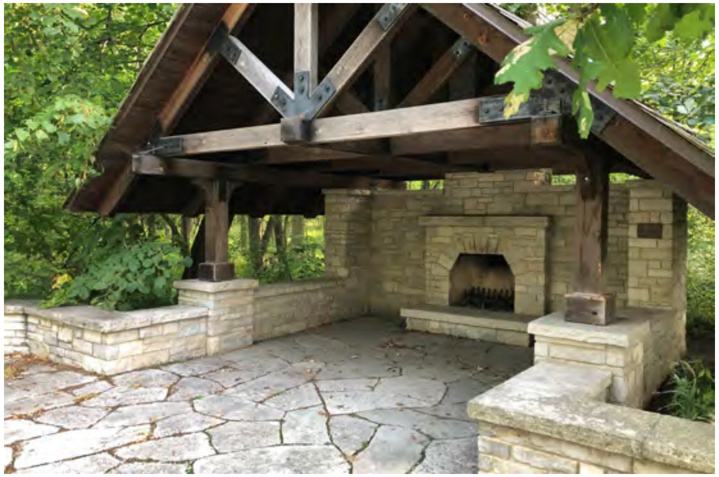

d. McDonald Woods Big Loop, Chicago Botanic Garden 15.23

4.52. Sculptures

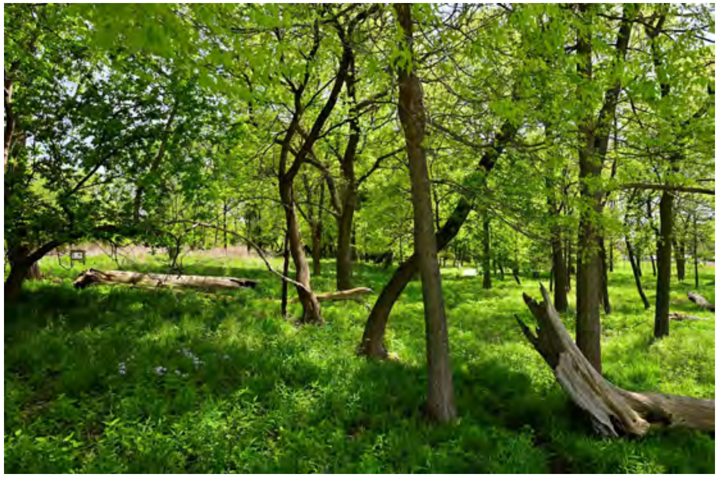

a. Woodland Loop, West Ridge Nature Park (9.21)

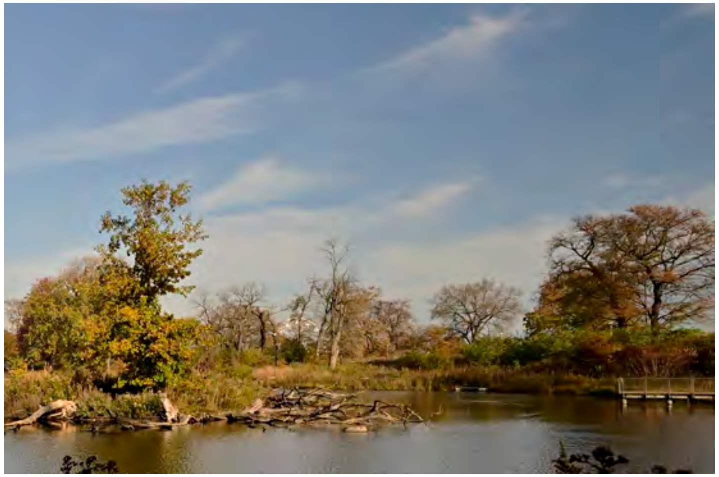

b. Nature Boardwalk, Lincoln Park Zoo (12.41)

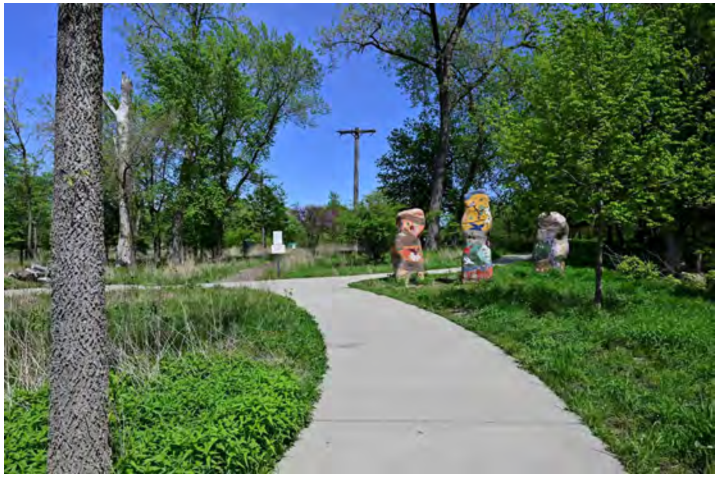

c. Woodland Loop, West Ridge Nature Park (9.21)

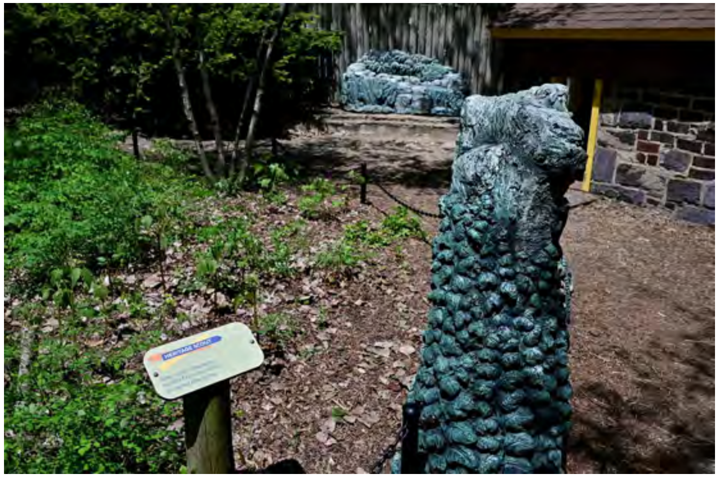

d. Park Loop, Indian Boundary Park (9.32)

4.53. Nature Play Areas

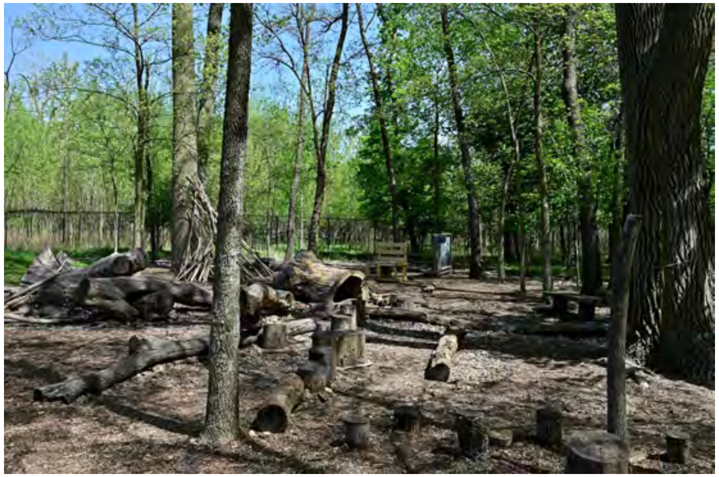

a. Woodland Loop, West Ridge Nature Park (9.21)

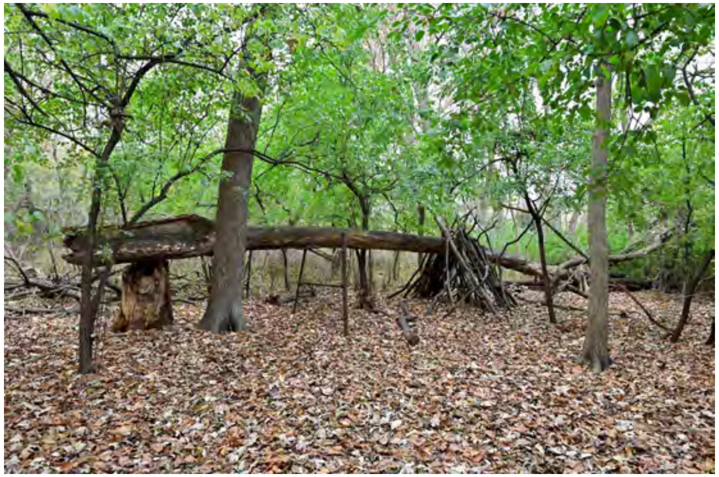

b. Woodland Loop, North Park Village Nature Center (9.12)

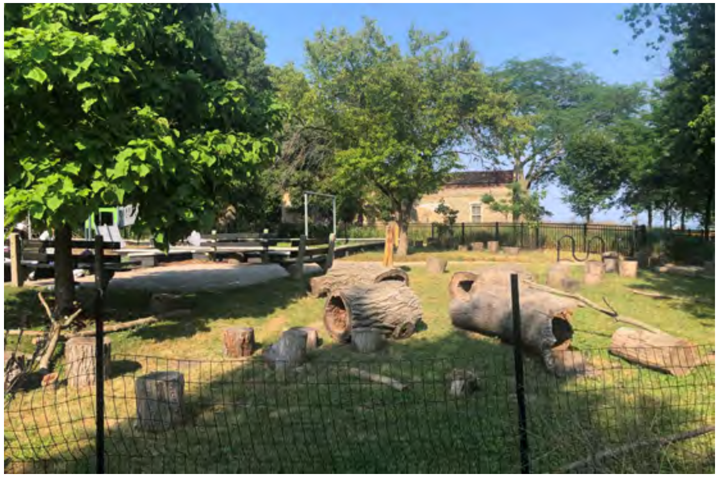

c. Leone Natural Area-Park Loop, Loyola-Leone Parks (10.23)

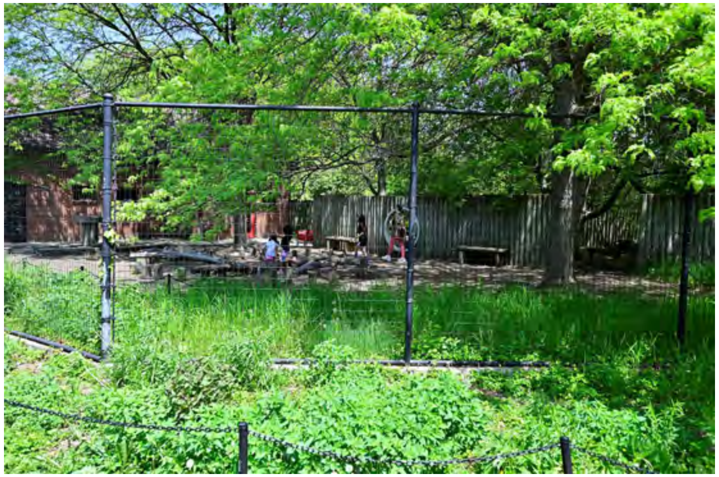

d. Park Loop, Indian Boundary Park (9.32)

4.54. Observation Structures

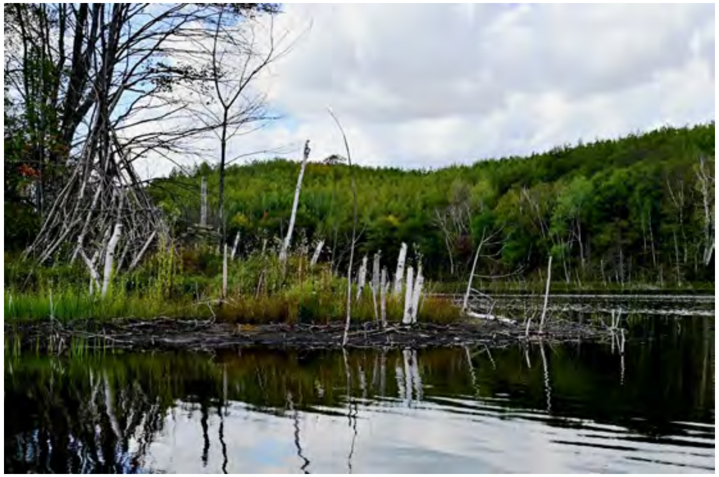

a. Sand Lake Paddle, Sand Lake (7.41)

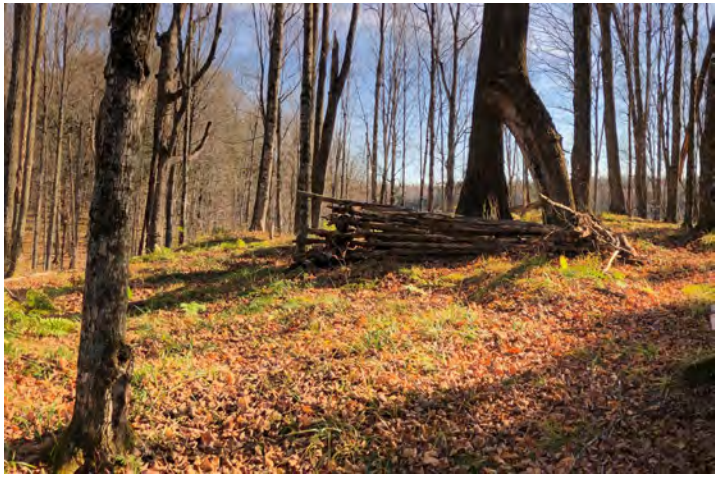

b. Beginner's Trail Loop, Lauterman National Recreation Trail (4.11)

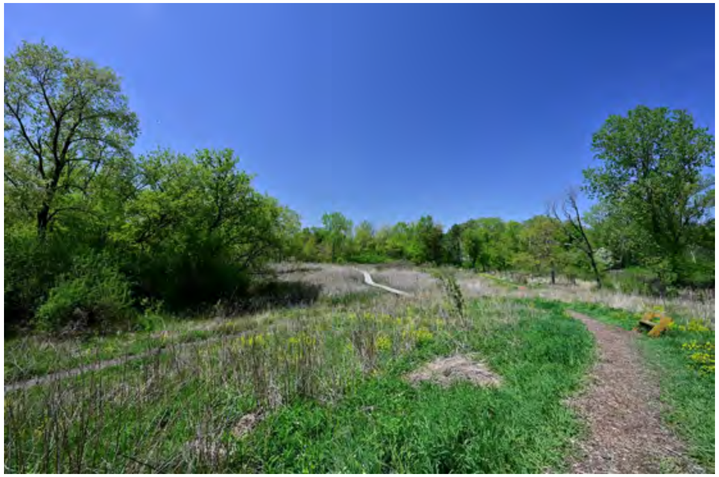

c. Wetland Loop, North Park Village Nature Center (9.11)

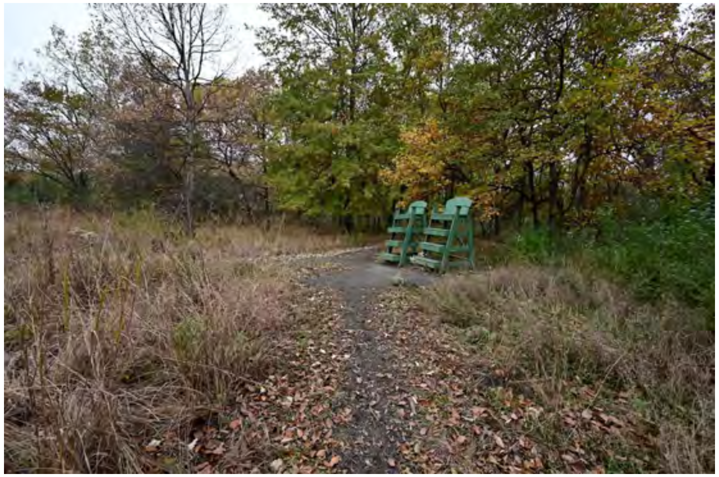

d. Walking Stick Woods, North Park Village Nature Center (9.15)

4.55. Observation Structures

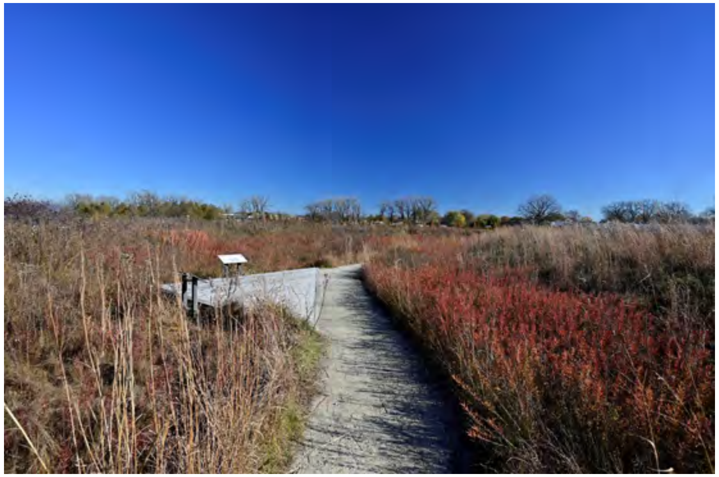

a. Dixon Prairie, Chicago Botanic Garden (15.25)

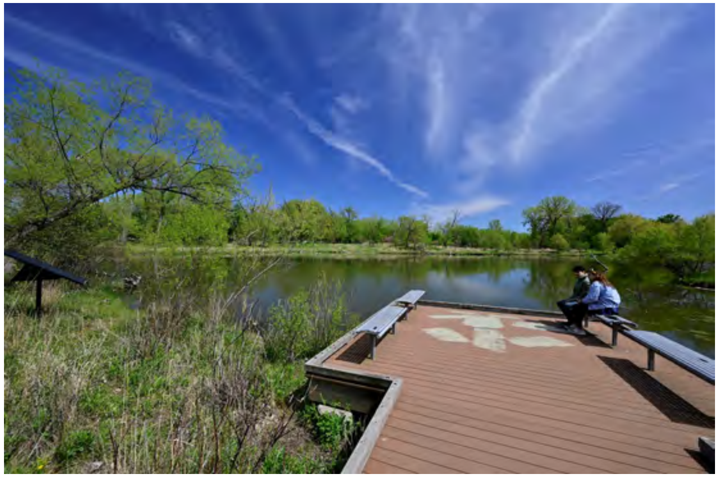

b. Lake Loop, West Ridge Nature Park (9.22)

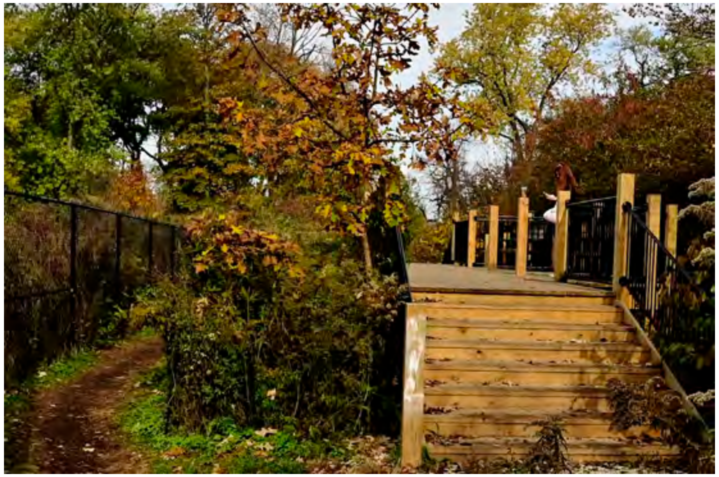

c. Loop Trail, Bill Jarvis Migratory Bird Sanctuary (11.31)

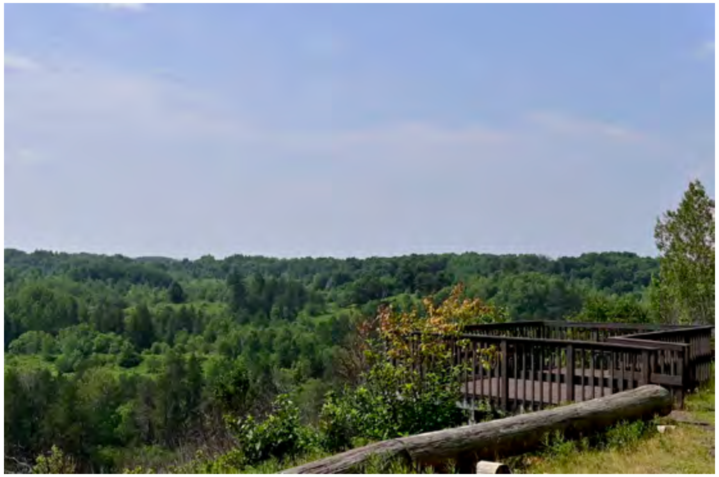

d. Lepage Creek Overlook, Lake Anna Trails (7.35)

4.56. Drinking Water Wells and Fountains

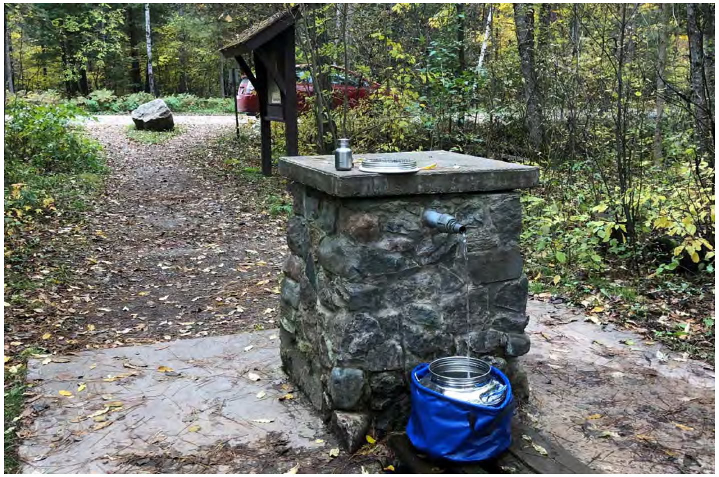

a. Artesian Well near Lakeshore-Ridge Trail Loop, Lost Lake Recreation Area

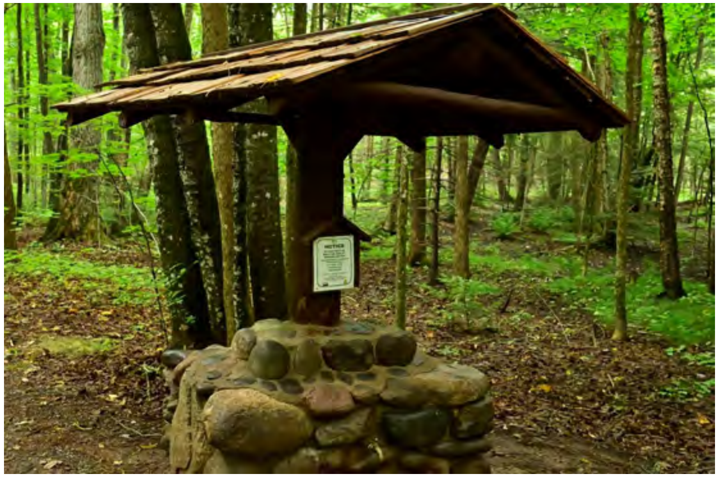

b. CCC Era Water Well with Spigot near Franklin Lake Nature Trail, Hidden Lakes Trail (6.11)

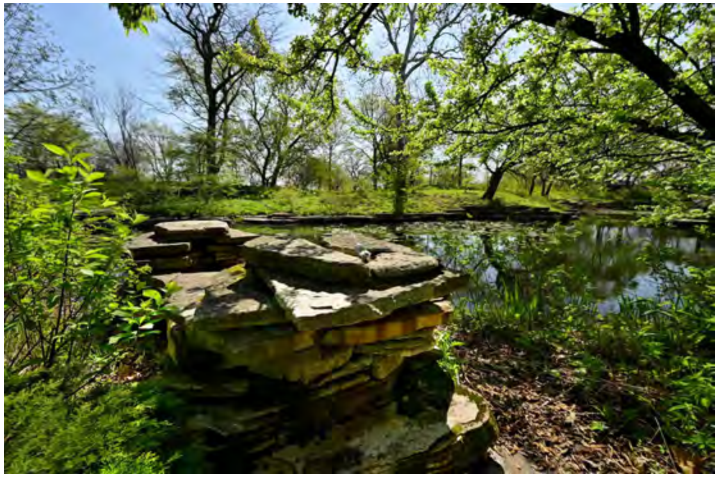

c. Lily Pool Loop, Alfred Caldwell Lily Pool (12.11)

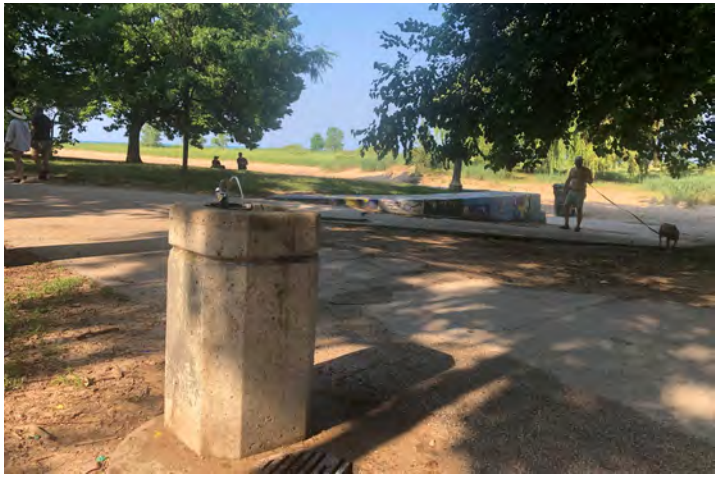

d. Loyola Natural Area-Park Loop, Loyola-Leone Parks (10.21)

5. Explorable Nature

5.1. ***Uses and Restrictions-*** The ability to explore and interact with nature beyond the trail's edge can be an important part of the forest therapy experience. Walking along fallen trees (5.11a), foraging for spring ramps and summer berries (5.11b–c), and making a winter fire along the trail for warmth and hot tea (5.11d) are a few of the many interactive opportunities permitted along most of the trails in the Northwoods study area. Restrictions on such uses were evident for most Chicago trails as posted online (5.12a) or onsite (5.12b), while restrictions for Northwoods trails were usually posted on trailhead kiosks ((5.12c–d).

5.2. ***Museumification-*** Museumification includes physical (5.21) or symbolic (5.22) barriers including fences, ropes, curbs, and other design elements that prevent or discourage visitors from stepping beyond the trail's edge. While such devices are often necessary to protect sensitive areas from trampling or other harmful activities, when excessive they can have the effect of distancing visitors from extra-visual interactions with natural features, limiting experiences to sight observation as one would in a museum. In the Chicago study area, tall chain link fencing along one trail (5.21c) was particularly inhibiting; overgrown with weedy vines, it obscured views of the natural area within it.

5.3. ***On-Trail Engagement-*** In high-use or fragile prairie and meadow areas, narrow trail segments and fencing that allows vegetation to permeate into the trail bed can provide some degree of on-trail nature engagement for touching and smelling plants, observing insects, and other activities (5.31a–b). In woodland areas, locating trails in close proximity to a few large or distinctive trees along the route can do the same, increasing the vividness of the arboreal experience. While paddle trails are naturally very interactive, foot trails along water bodies can invite shoreland erosion in high use areas unless carefully managed. In such cases, providing a few openings along the edges of ponds and lakes, sufficiently designed to minimize impact (5.32a–c) is a good solution, and in other cases platforms and observation decks extending into the water can improve the experience while still keeping visitors from direct contact with the water (5.32d).

5.11. Explorable Nature– Uses

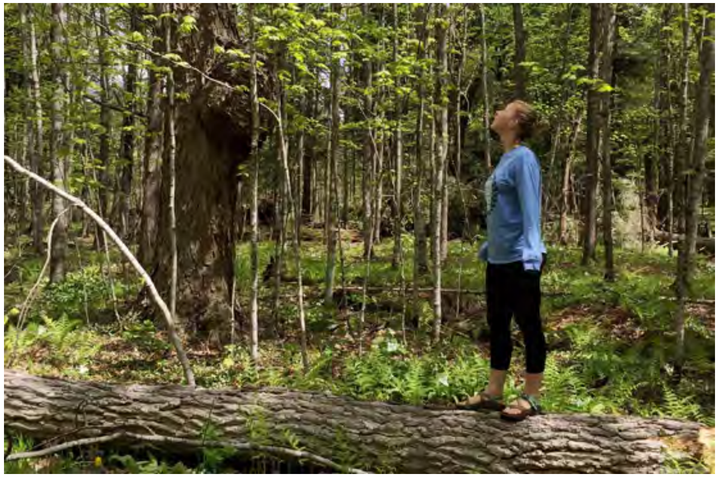

a. Main Trail, Fox Maple Woods (3.11)

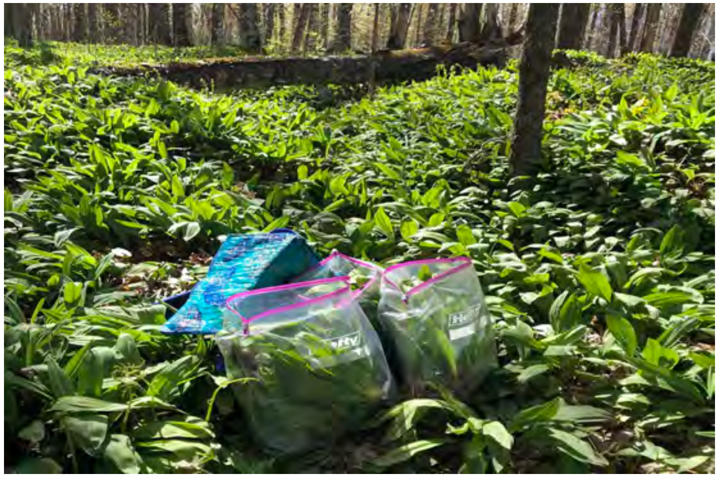

b. Lakeshore Trail Loop, Lost Lake Recreation Area (4.21)

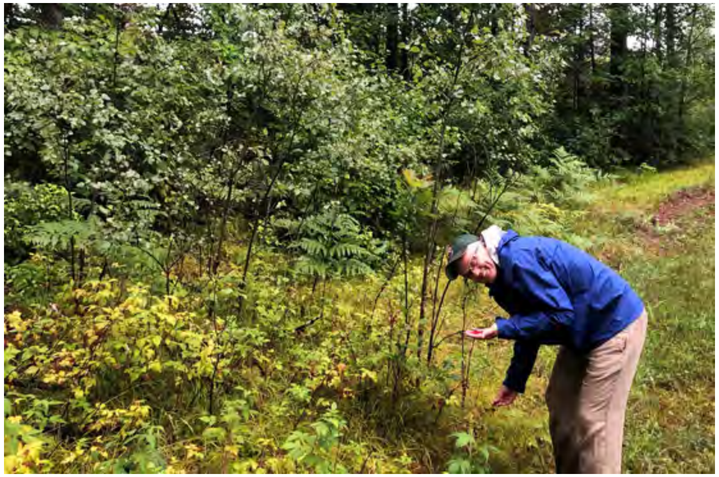

c. North Loop, Lake Emily Recreation Trail (2.21)

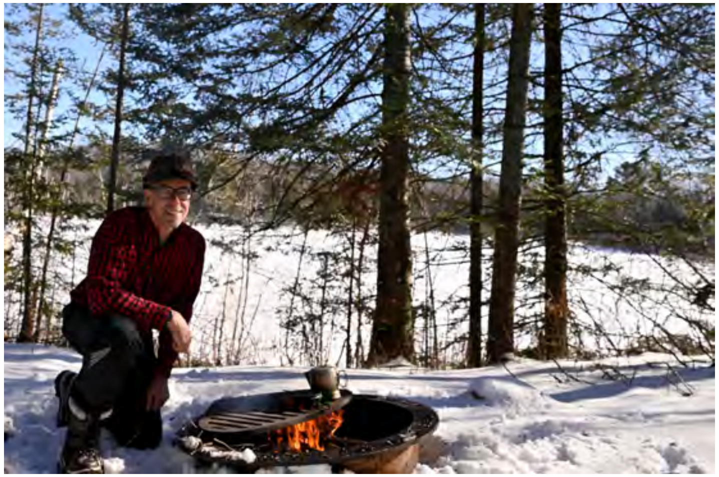

d. Full Loop Trail (winter only), Perch Lake (3.21)

5.12. Explorable Nature– Restrictions

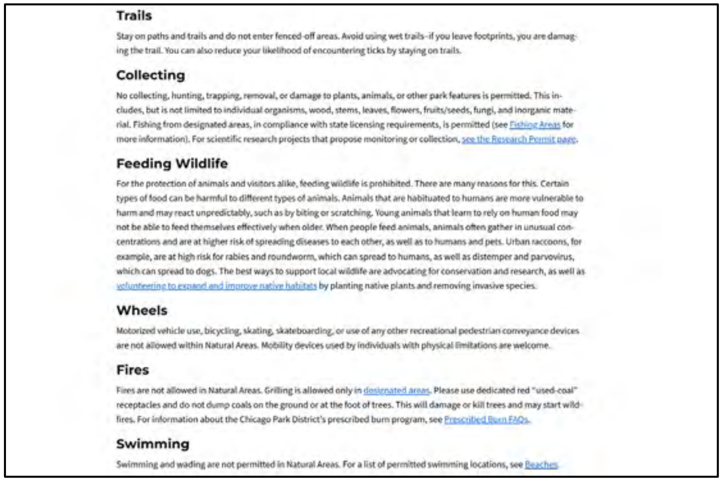

Trails

Stay on paths and trails and do not enter fenced-off areas. Avoid using wet trails–if you leave footprints, you are damaging the trail. You can also reduce your likelihood of encountering ticks by staying on trails.

Collecting

No collecting, hunting, trapping, removal, or damage to plants, animals, or other park features is permitted. This includes, but is not limited to individual organisms, wood, stems, leaves, flowers, fruits/seeds, fungi, and inorganic material. Fishing from designated areas, in compliance with state licensing requirements, is permitted (see Fishing Areas for more information). For scientific research projects that propose monitoring or collection, see the Research Permit page.

Feeding Wildlife

For the protection of animals and visitors alike, feeding wildlife is prohibited. There are many reasons for this. Certain types of food can be harmful to different types of animals. Animals that are habituated to humans are more vulnerable to harm and may react unpredictably, such as by biting or scratching. Young animals that learn to rely on human food may not be able to feed themselves effectively when older. When people feed animals, animals often gather in unusual concentrations and are at higher risk of spreading diseases to each other, as well as to humans and pets. Urban raccoons, for example, are at high risk for rabies and roundworm, which can spread to humans, as well as distemper and parvovirus, which can spread to dogs. The best ways to support local wildlife are advocating for conservation and research, as well as volunteering to expand and improve native habitats by planting native plants and removing invasive species.

Wheels

Motorized vehicle use, bicycling, skating, skateboarding, or use of any other recreational pedestrian conveyance devices are not allowed within Natural Areas. Mobility devices used by individuals with physical limitations are welcome.

Fires

Fires are not allowed in Natural Areas. Grilling is allowed only in designated areas. Please use dedicated red "used-coal" receptacles and do not dump coals on the ground or at the foot of trees. This will damage or kill trees and may start wildfires. For information about the Chicago Park District's prescribed burn program, see Prescribed Burn FAQs.

Swimming

Swimming and wading are not permitted in Natural Areas. For a list of permitted swimming locations, see Beaches.

a. Chicago Park District Natural Areas Website
https://www.chicagoparkdistrict.com/natural-areas-rules-faqs

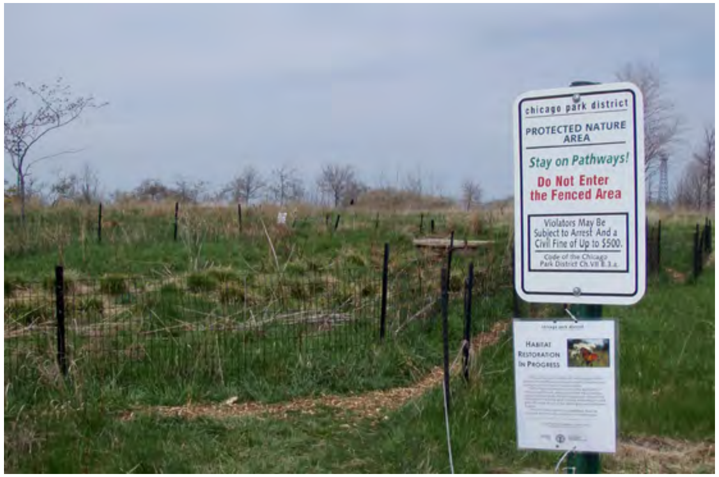

b. Bird Sanctuary Main Loop, Montrose Point (11.11)

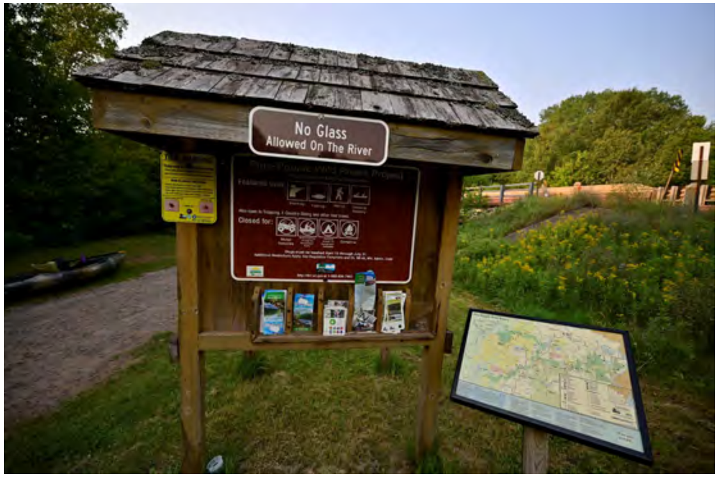

c. Oxbow Paddle, Pine River Oxbow (1.71)

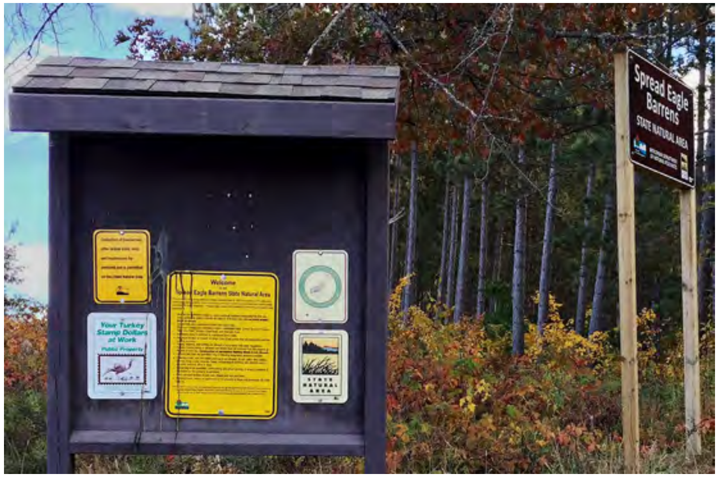

d. Fire Lane Rd. Loop, Spread Eagle Barrens (7.12)

5.21. Explorable Nature– Museumification, Physical Barriers

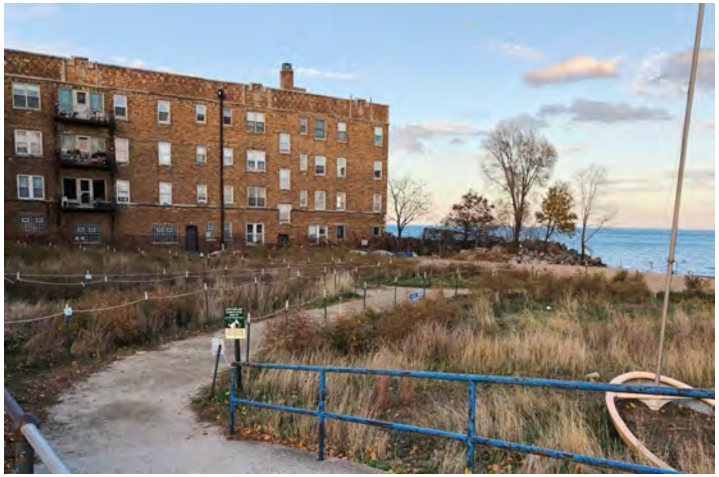

a. Leone Natural Area-Park Loop, Loyola-Leone Parks (10.23)

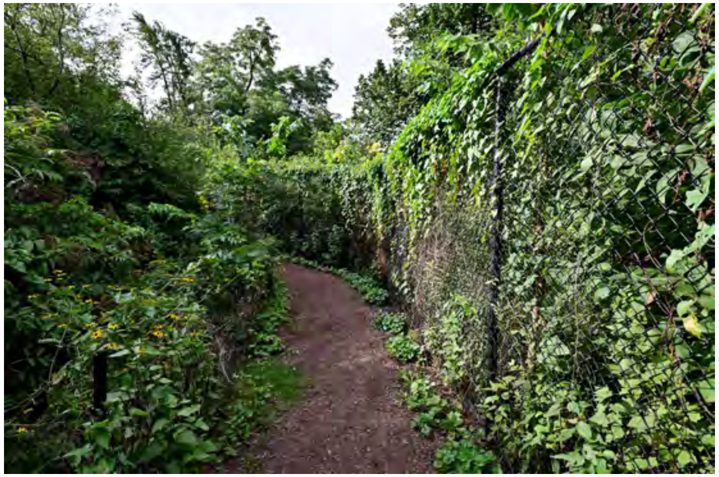

b. Bird Sanctuary Loop, Bill Jarvis Migratory Bird Sanctuary (11.31)

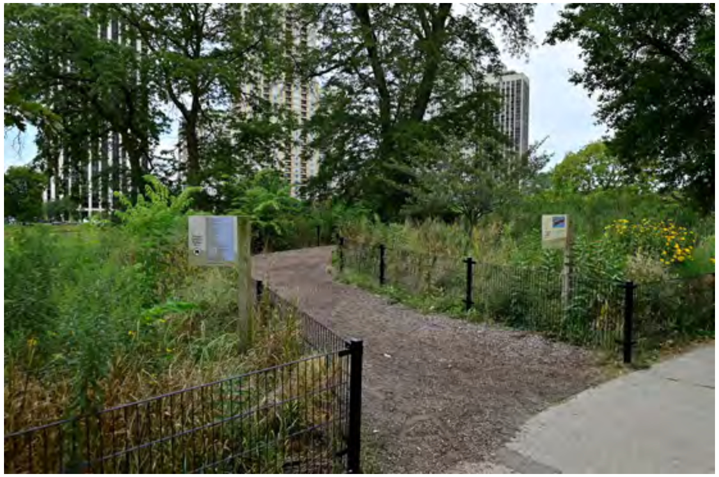

c. Natural Area Loop, North Pond (12.31)

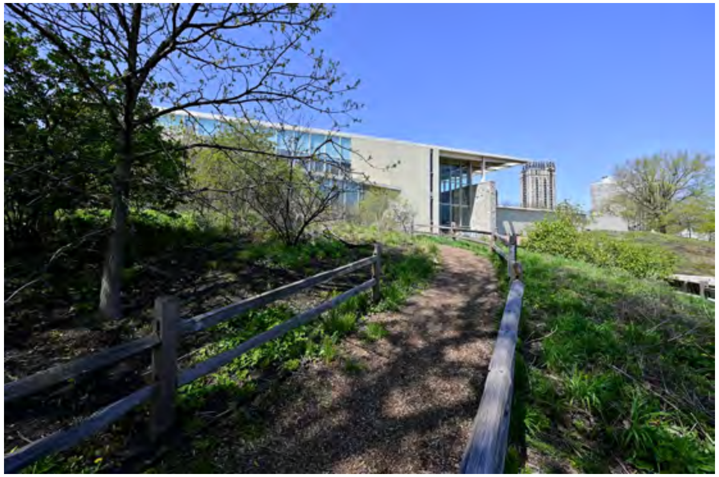

d. Deb Lahey Nature Trails, Nature Museum (12.21)

5.22. Explorable Nature– Museumification, Symbolic Barriers

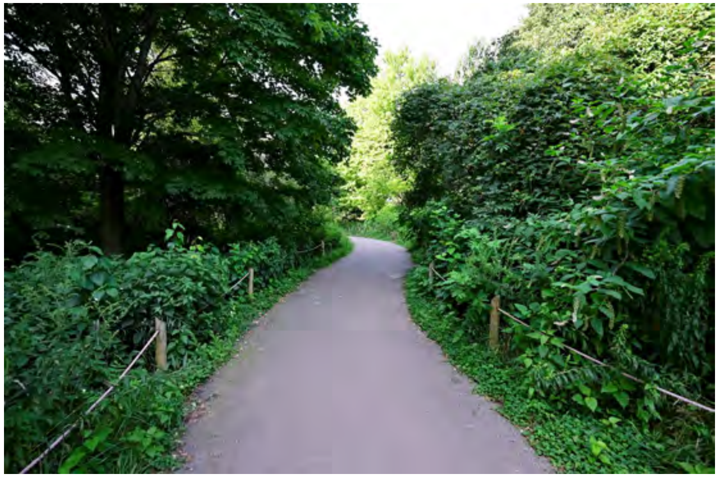

a. Bird Sanctuary Main Loop, Montrose Point (11.11)

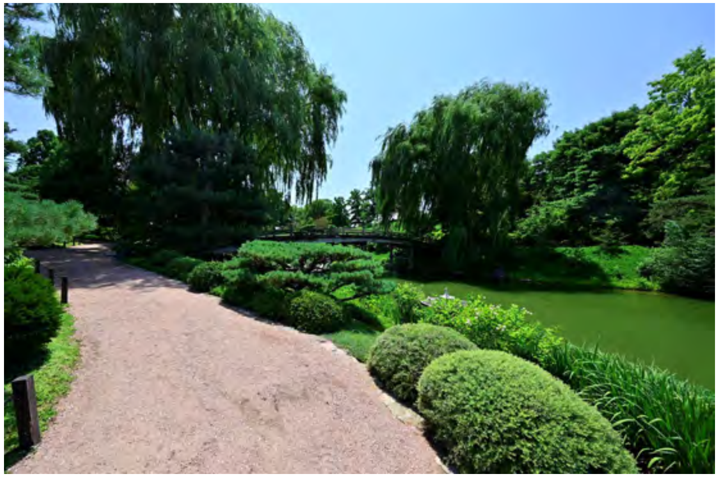

b. Japanese Garden, Chicago Botanic Garden (15.24)

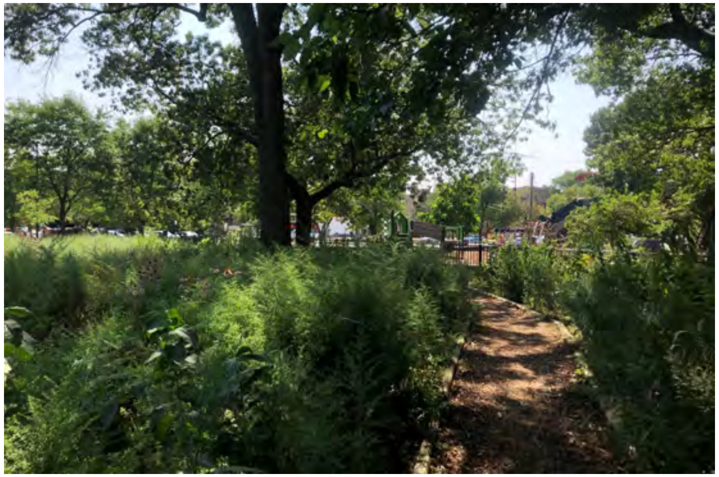

c. Leone Natural Area-Park Loop, Loyola-Leone Parks (10.23)

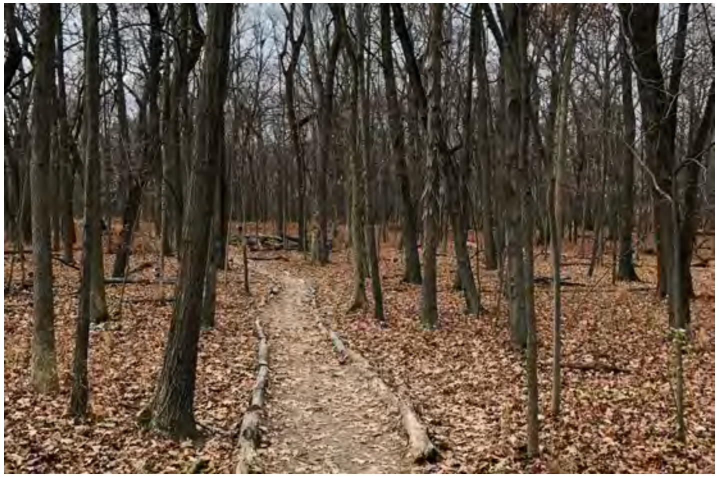

d. Woodland-River West Loop, Harms Woods Forest Preserve (14.11)

5.31. Explorable Nature– On-Trail Engagement

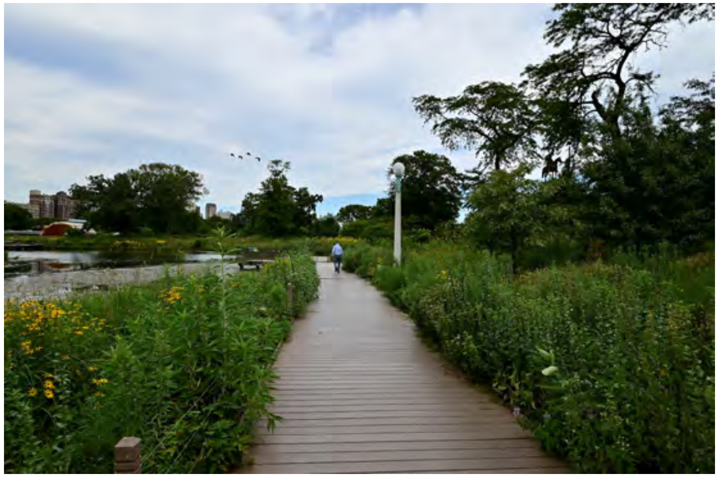

a. Nature Boardwalk, Lincoln Park Zoo (12.41)

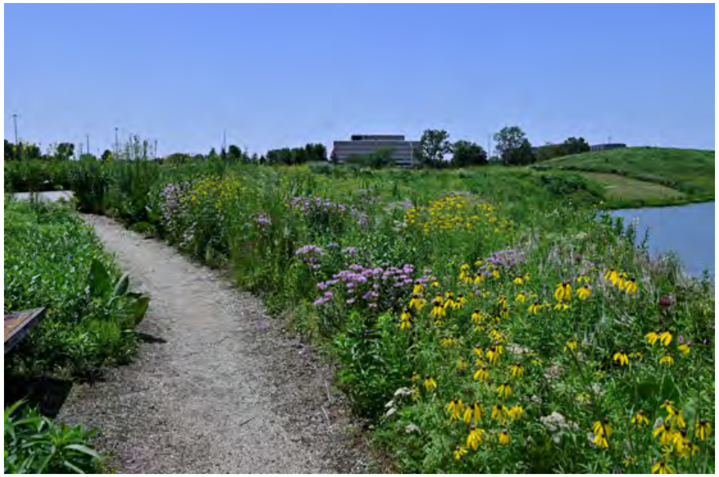

b. Dixon Prairie, Chicago Botanic Garden (15.25)

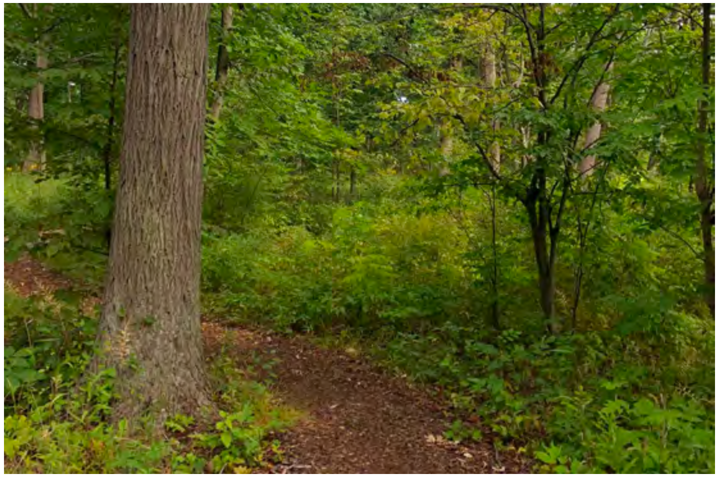

c. McDonald Woods Big Loop, Chicago Botanic Garden (15.23)

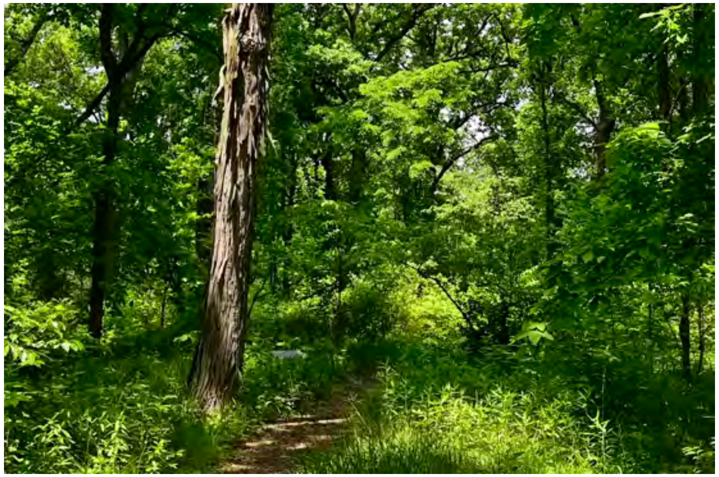

d. McDonald Woods Big Loop, Chicago Botanic Garden (15.23)

5.32. Explorable Nature– Water Access Engagement

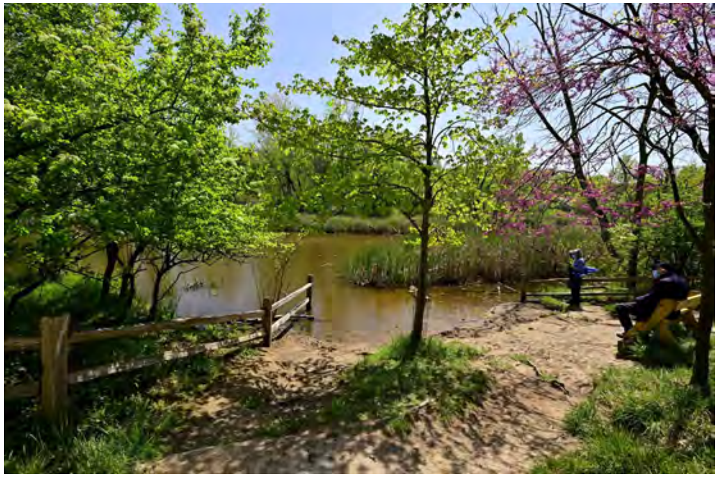

a. Wetland Loop, North Park Village Nature Center (9.11)

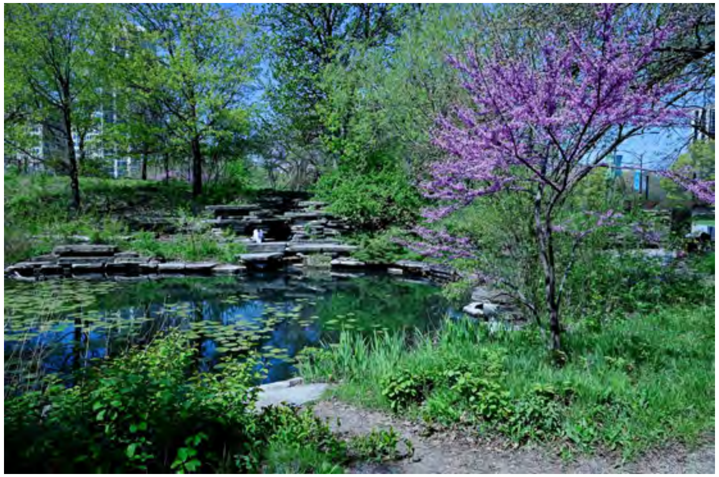

b. Lily Pool Loop, Alfred Caldwell Lily Pool (12.11)

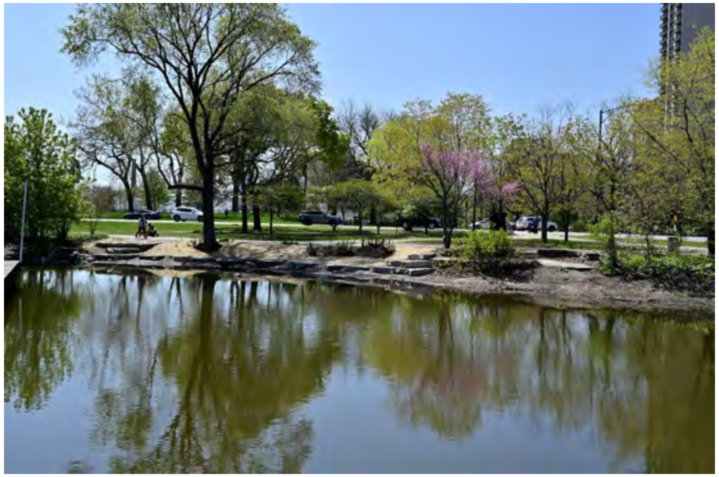

c. Natural Area Loop, North Pond (12.31)

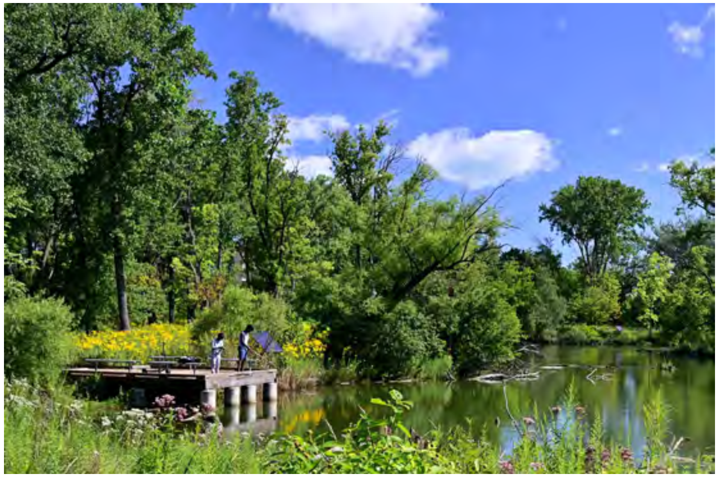

d. Lake Loop, West Ridge Nature Park (9.23)

6. Interpretation and Stewardship

6.1. ***Interpretive Signage-*** Signs in a variety of designs and placements can impart ecological, cultural/historical, or other types of interpretive information to trail visitors. These include trail stops (6.11), central interpretive signs at trail entries and key observation points (6.12), and markers, brochures, and flexible signs (6.13) that can help make messages more dynamic for seasonal or thematic purposes. Experiential signs designed specifically for enhancing forest therapy experiences were not present at the trails in my sample, but flexible signage (6.13c–d) lends itself well to this type of application. Stewardship messages (6.14) are a special kind of environmental interpretation that convey information and sometimes also impart a use or management ethic. These can be effective ways to teach stewardship, and if not preachy or overly technical can deepen appreciation for sites.

6.2. ***Demonstration Gardens-*** Demonstration gardens (6.21) can also be effective ways to teach visitors about native plants and sustainable practices, and in some cases invite participation and involvement as an entrée to land stewardship.

6.3. ***Programs and Information-*** Programs aimed at children and adults, from story (6.31a) and sensory experience walks (6.31b) to onsite tours (6.31c) and off-site displays (6.31d) are additional ways to interpret sites and deepen environmental learning.

6.4. ***Volunteer Opportunities-*** Volunteer ecological restoration opportunities abound on city and forest preserve sites in the Chicago study area, where regular workdays are scheduled to collect seeds (6.41a), cut brush (6.41b), and conduct other restorative activities. Litter pickups, trail improvement projects, and other activities developed by scouting groups (6.41c) and other organizations (6.41d) add to the spectrum of activities whereby individuals can participate in land stewardship as an entrée to or outgrowth from forest therapy experiences.

6.11. Interpretation and Stewardship–Trail Stops Interpretive Signs

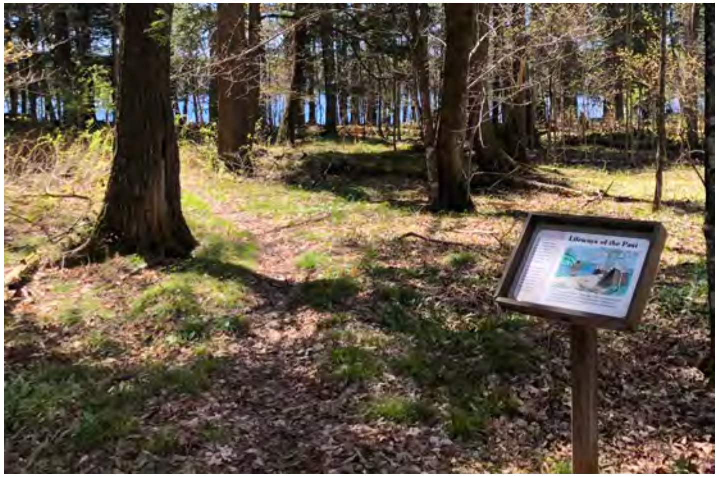

a. Assessor's Interpretive Trail, Lost Lake Recreation Area (4.22)

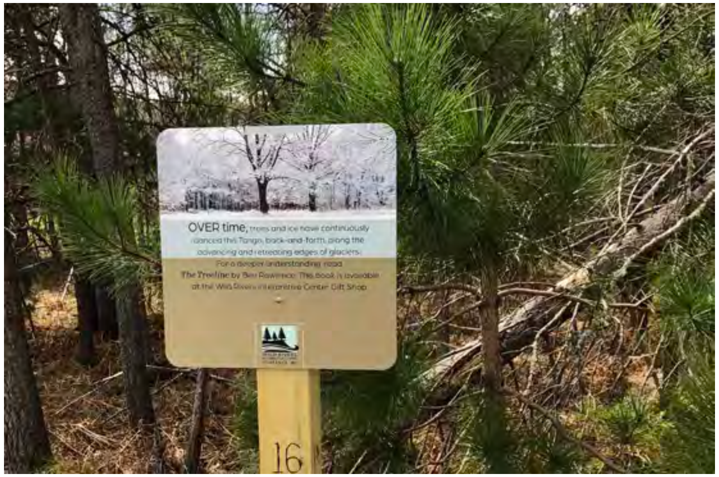

b. The Return of Trees Interpretive Trail, Wild Rivers Interpretive Center (1.11)

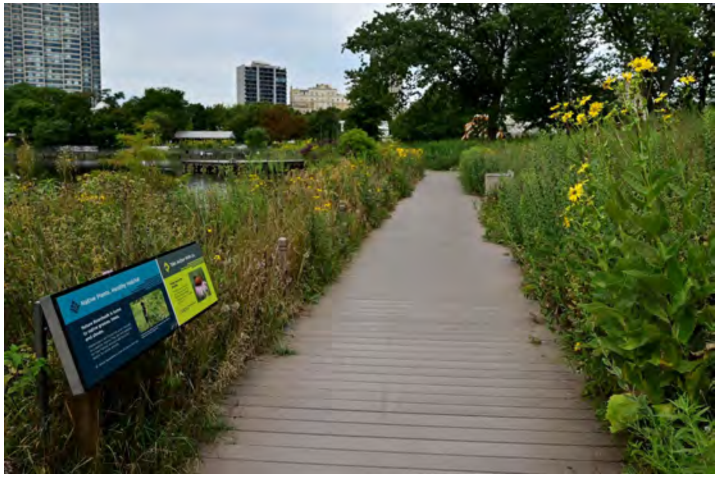

c. Nature Boardwalk, Lincoln Park Zoo (12.41)

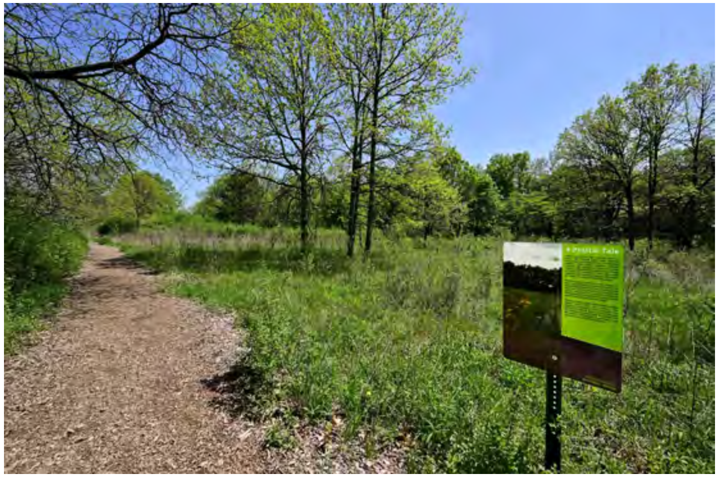

d. Wetland Loop, North Park Village Nature Center (9.11)

6.12. Interpretation and Stewardship– Central Interpretive Signs

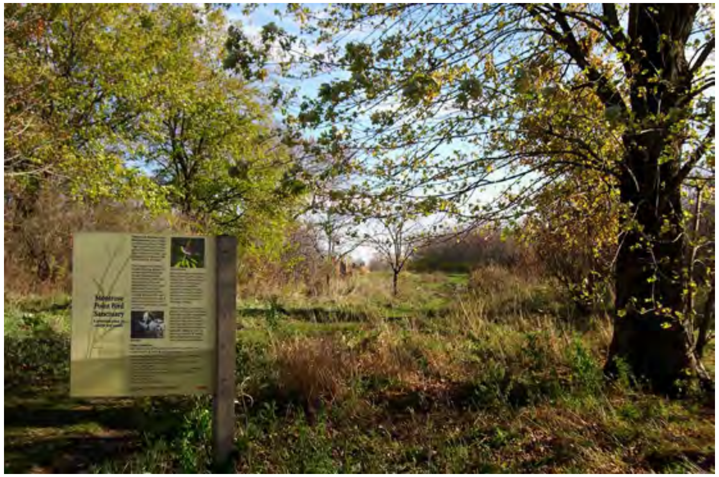

a. Bird Sanctuary Main Loop, Montrose Point (11.11)

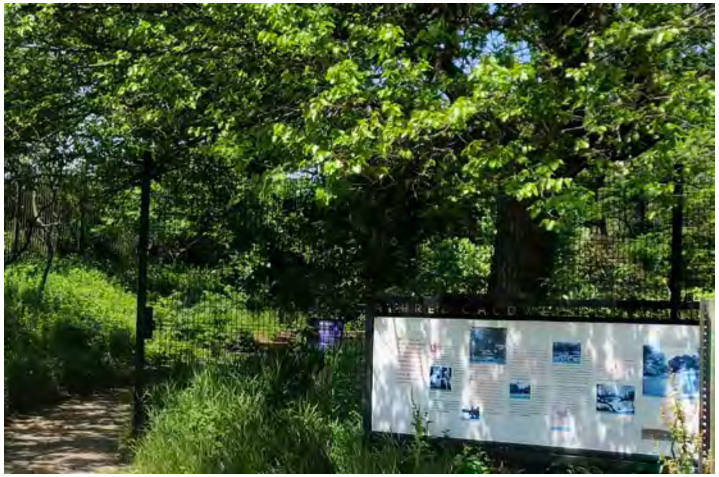

b. Lily Pool Loop, Alfred Caldwell Lily Pool (12.11)

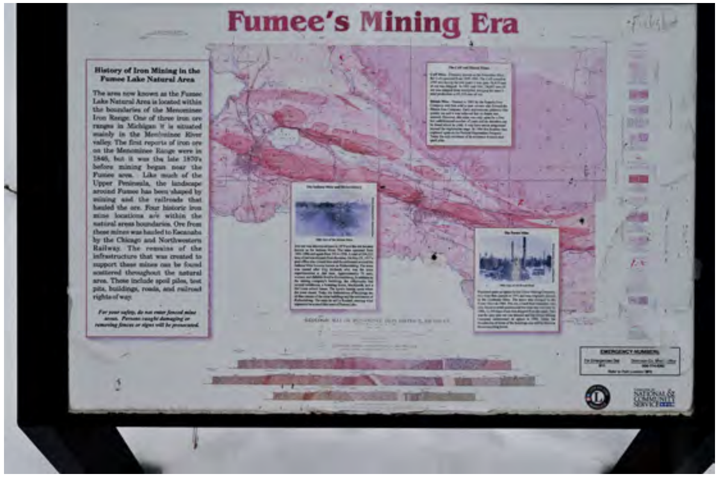

c. Big Fumee Lake Bike Loop, Fumee Lake Natural Area (8.24)

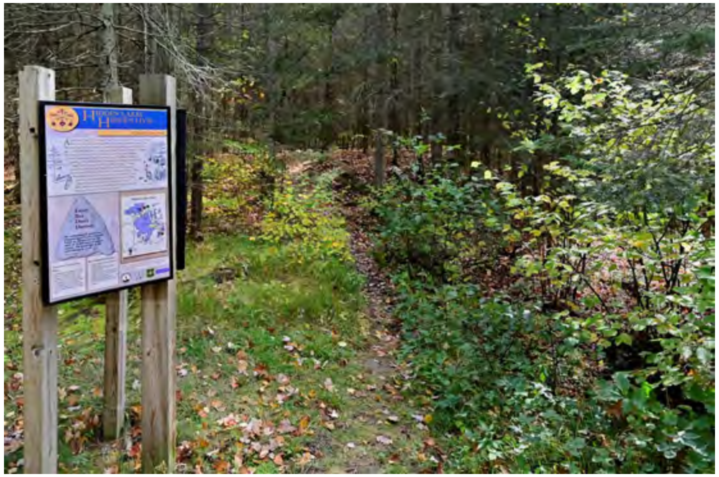

d. McKinley Lake–Luna Lake segment, Hidden Lakes Trail (6.14)

6.13. Interpretation and Stewardship– Markers, Brochures, and Flexible Signs

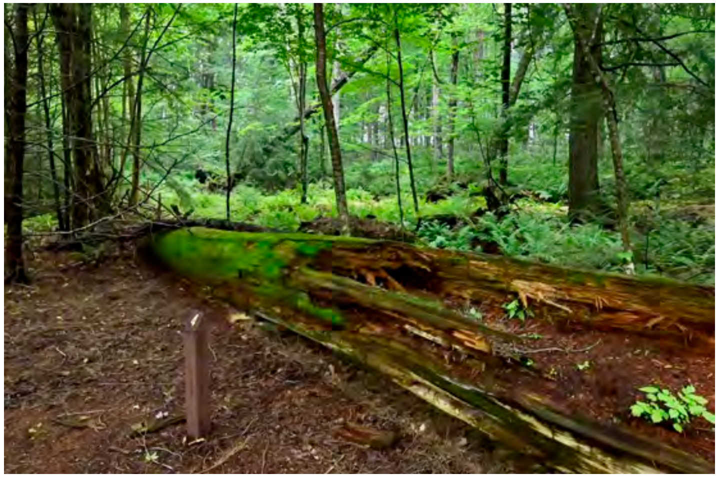

a. Franklin Nature Trail, Hidden Lakes Trail, (6.11)

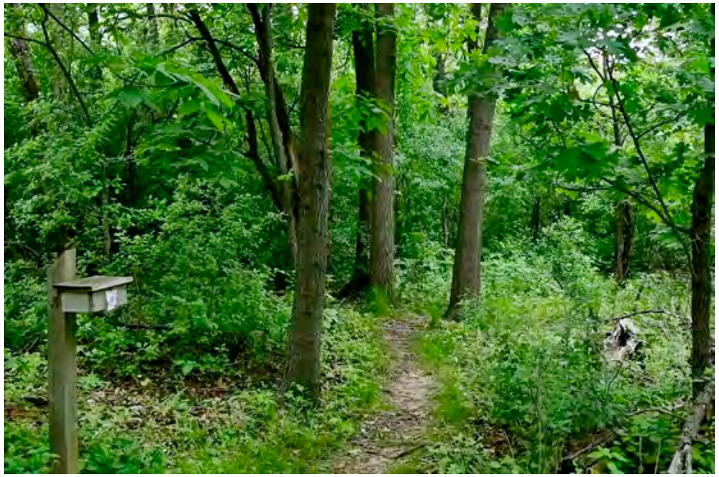

b. Entry to Somme Prairie Grove (16.2)

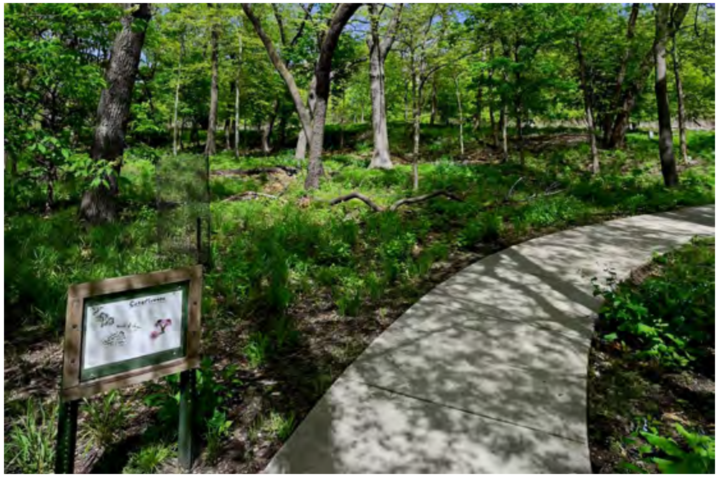

c. Woodland Loop, West Ridge Nature Park (9.21)

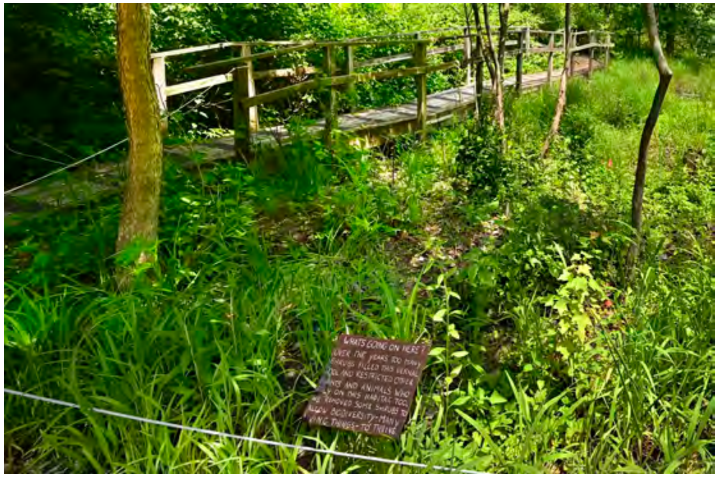

d. McDonald Woods Big Loop, Chicago Botanic Garden (15.23)

6.14. Interpretation and Stewardship– Stewardship Message Signs

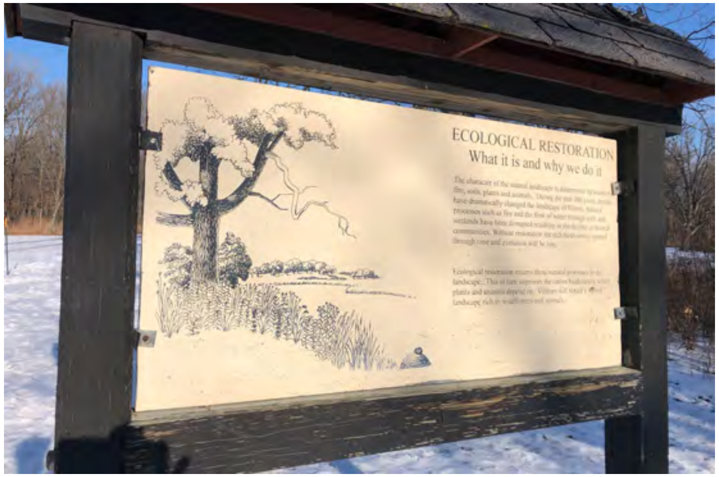

a. West Outer Loop, Somme Woods (16.23)

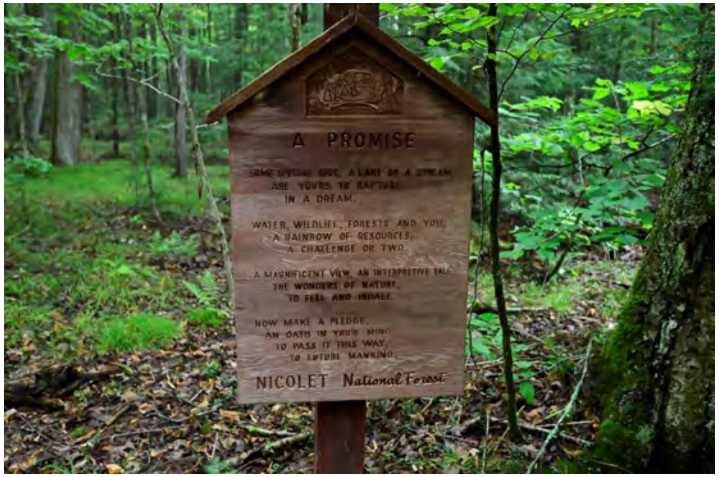

b. Franklin Nature Trail, Hidden Lakes Trail, (6.11)

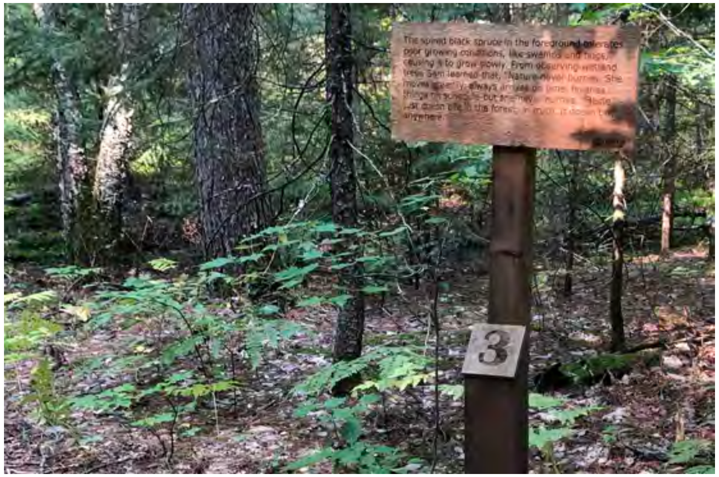

c. Sam Campbell Memorial Trail (6.32)

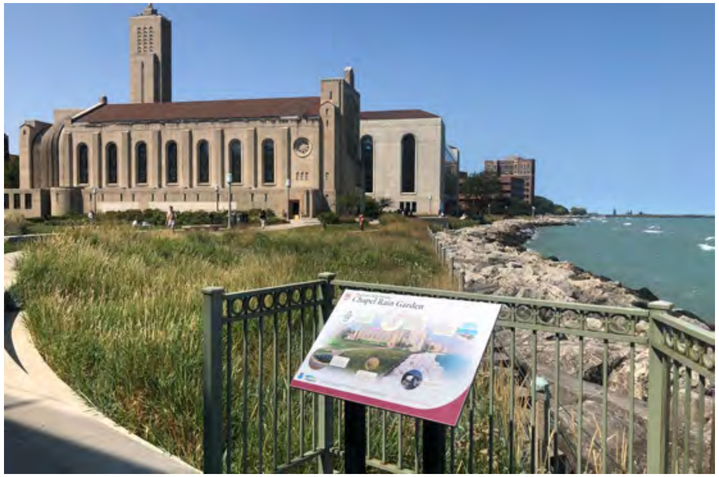

d. Loop Walk, Loyola University Lakeshore Campus (10.11)

6.21. Interpretation and Stewardship– Demonstration Gardens

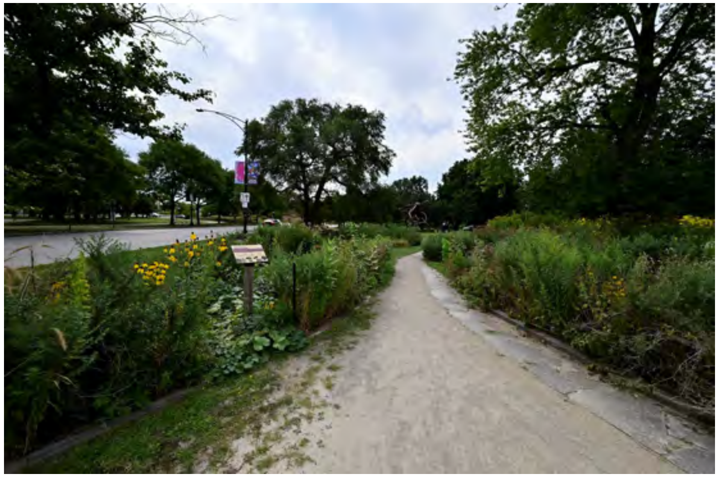

a. Deb Lahey Nature Trail, Nature Museum (12.21)

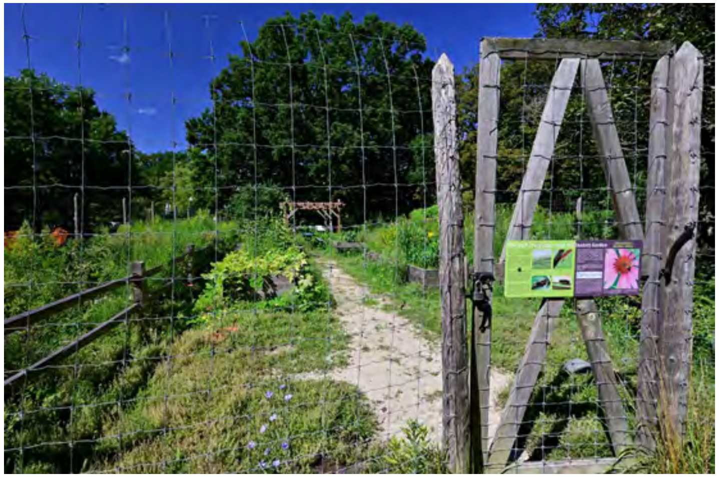

b. North Park Village Nature Center (9.1)

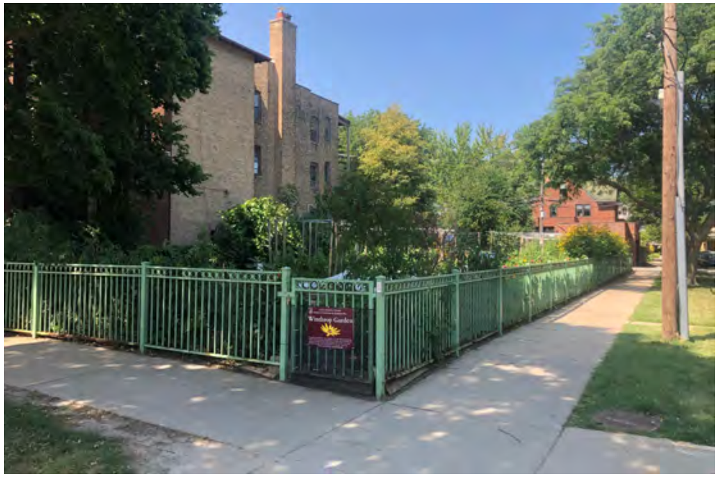

c. Loop Walk, Loyola University Lakeshore Campus (10.11)

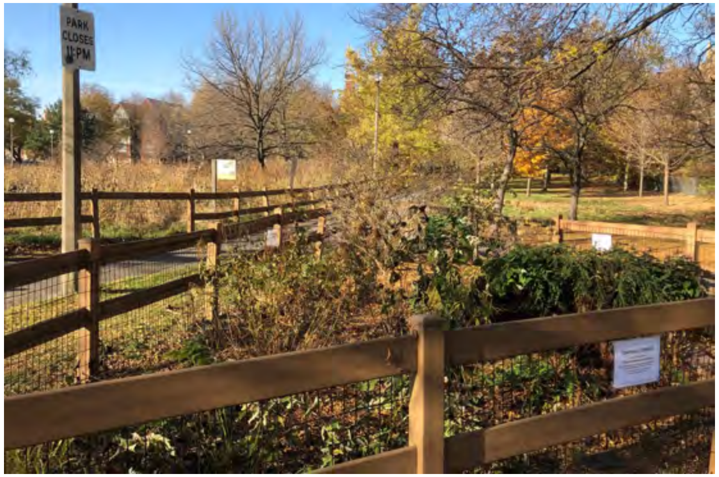

d. Park Loop, Indian Boundary Park (9.32)

6.31. Interpretation and Stewardship– Programs & Information

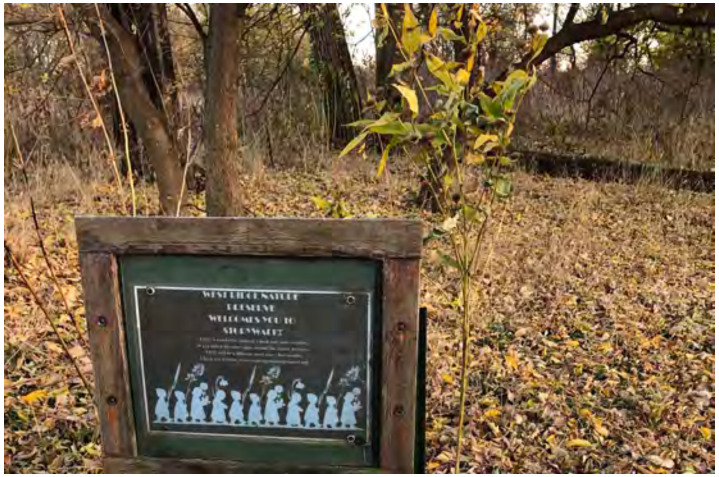

a. Woodland Loop, West Ridge Nature Park (9.12)

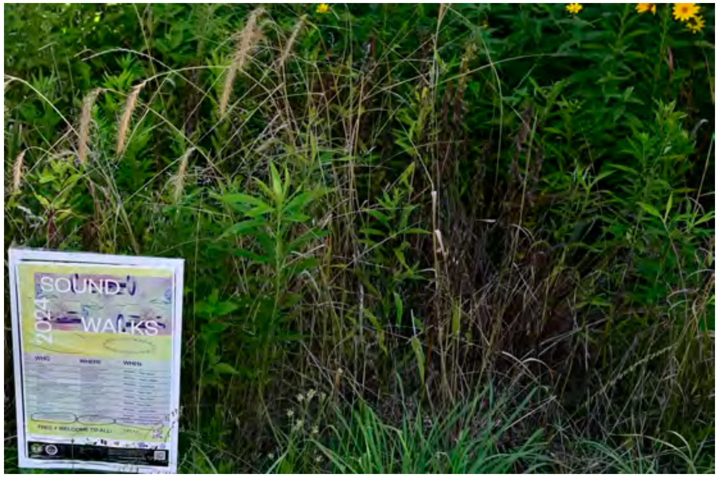

b. Woodland Loop, West Ridge Nature Park (9.12)

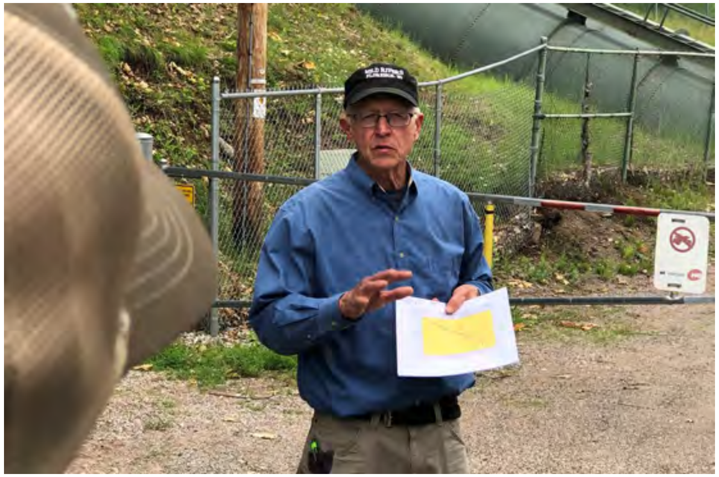

c. South Bank Trail, Pine River-Breakwater Falls (1.62)

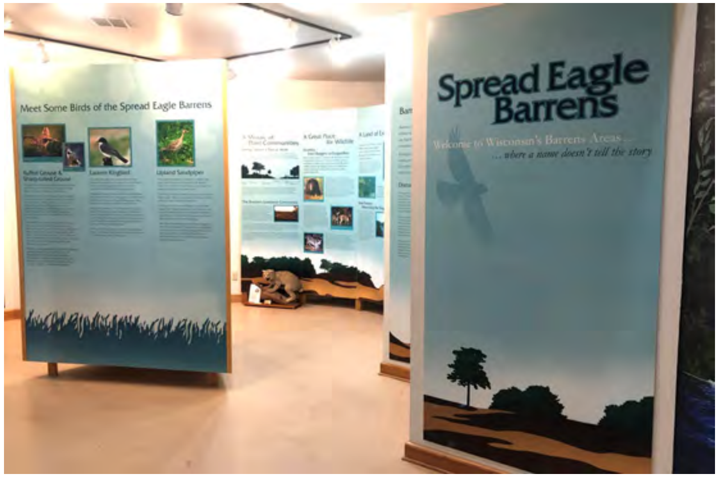

d. Spread Eagle Barrens (7) Exhibit at Wild Rivers Interpretive Center (1.1)

6.41. Interpretation and Stewardship– Volunteer Opportunities

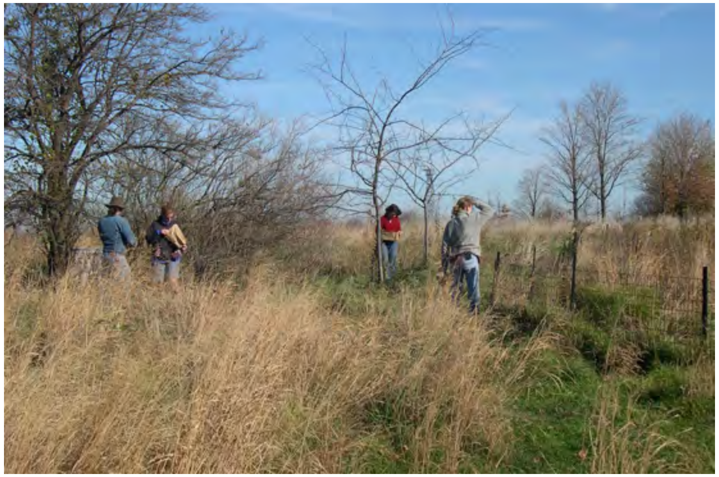

a. Bird Sanctuary Main Loop, Montrose Point (11.11)

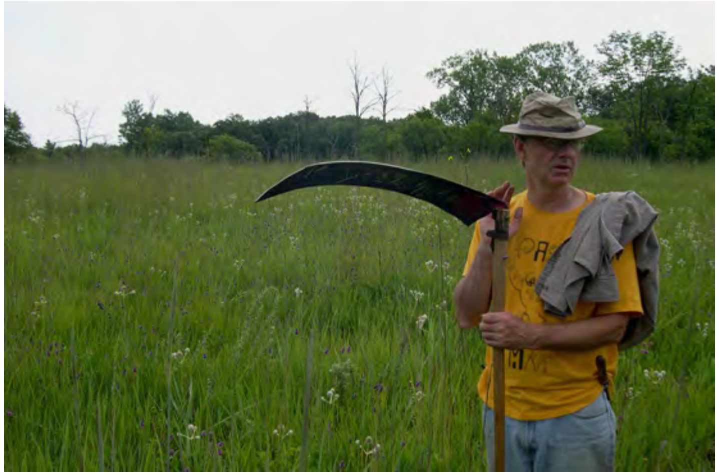

b. Prairie Inner Loop, Somme Prairie Grove (16.23)

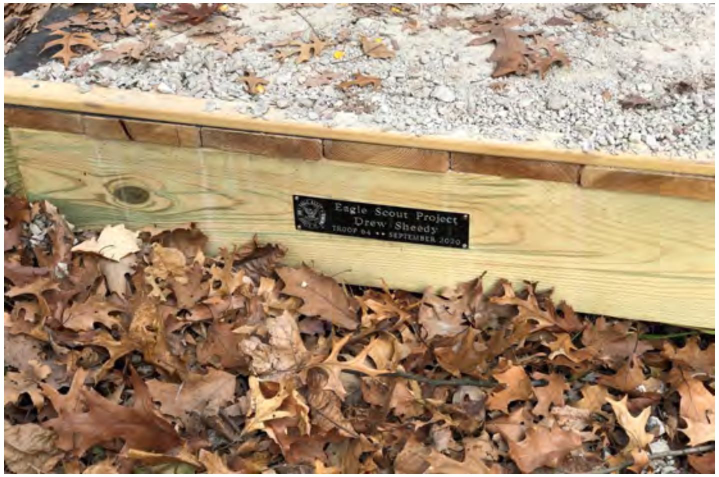

c. Full Loop, Harms Woods Nature Preserve (14.14)

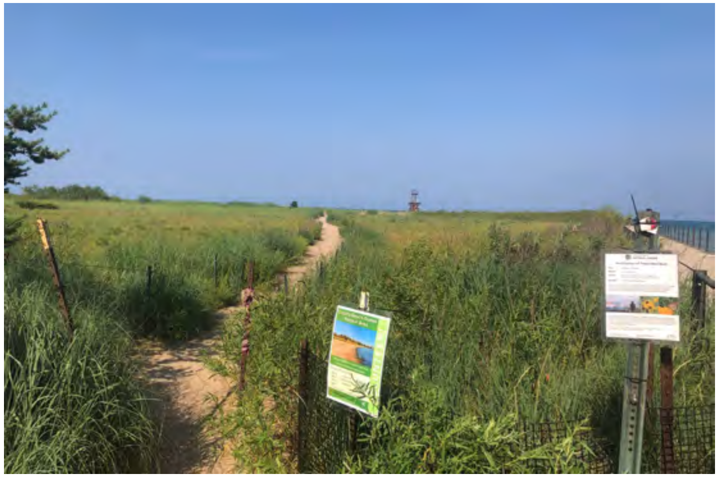

d. Point-Dunes-Lake-Prairie Loop, Montrose Point (11.13)
