# Supplementary material for: Forest Therapy Trails: Development and Application of an Assessment Protocol
Source: Int J Environ Res Public Health. 2025 Sep 16;22(9):1440. doi: 10.3390/ijerph22091440 (PMC12470198; doi:10.3390/ijerph22091440)
Supplement: Supplementary file 1 [file ijerph-22-01440-s001.zip › Supplementary Tables S1-S9.pdf]

Table S1. Site level characteristics and ratings for the Northwoods study area.[1]

Nbr.	Place, Site Name	Area (ha)	N Foot	N Paddle	N Bike	Total Trails	Beauty	Integrity	Tranquility	Accessibility	Total Score	Pct.
1	**Pine-Popple Wild Rivers**	4,673	10	4	3	17						
1.1	Wild Rivers Interpretive Center	16	1	0	0	1	2	1	1	3	7	58%
1.2	Pine River Flats	227	0	1	0	1	3	3	3	2	11	92%
1.3	Pine River Outcrops	129	2	0	0	2	3	2	2	1	8	67%
1.4	Pine River- Meyers Falls/Bull Falls	146	2	0	1	3	3	3	3	2	11	92%
1.5	Pine River- LaSalle Falls	809	1	0	0	1	3	2	2	2	9	75%
1.6	Pine River-Breakwater Falls/Pine River Flowage	202	2	1	0	3	2	2	2	2	8	67%
1.7	Pine River Oxbow	486	0	1	0	1	3	3	2	3	11	92%
1.8	Savage-Robago Wild Lakes Complex	762	2	1	2	5	3	3	3	2	11	92%
2	**Florence County Forest and Parks**	14,756	8	2	1	11						
2.1	Sea Lion Lake	30	3	1	0	4	3	2	2	3	10	83%
2.2	Lake Emily Recreation Trail	263	3	1	0	4	2	2	3	3	10	83%
2.3	Halls Creek Trails	672	2	0	1	3	2	2	3	2	9	75%
3	**Rainbow Trail**	5,666	14	3	1	18						
3.1	Fox Maple Woods	17	4	0	0	4	3	3	2	3	11	92%
3.2	Perch Lake	121	2	1	0	3	3	3	2	3	11	92%
3.3	CCC Camp Rainbow	567	2	0	0	2	2	2	3	2	9	75%
3.4	Whisker Lake Wilderness	3,006	2	2	0	4	3	3	3	1	10	83%
3.5	Rainbow Hunter Walking Trails	567	4	0	0	4	3	3	3	2	11	92%
3.6	Multi-Sites	2,428	0	0	1	1	2	2	3	2	9	75%
4	**Lauterman/Lost Lake Recreation Areas**	3,035	7	1	1	9						
4.1	Lauterman National Recreation Trail	911	2	0	1	3	3	3	3	3	12	100%
4.2	Lost Lake Recreation Area	405	5	1	0	6	3	3	3	3	12	100%
5	**Brule River Cliffs**	2,023	2	1	1	4						
5.1	Brule River Cliffs	2,023	2	1	1	4	3	2	3	2	10	83%
6	**Hidden Lakes**	4,856	9	3	0	12						
6.1	Hidden Lakes Trail	4,856	6	0	0	6	3	3	3	2	11	92%
6.2	Hidden Lakes Dispersed Sites	4,856	1	3	0	4	3	2	3	2	10	83%
6.3	Other Area Trails of Interest	100	2	0	0	2	3	3	3	2	11	92%
7	**Spread Eagle Barrens**	3,440	9	2	1	12						
7.1	Fire Lane Rd.	607	2	0	1	3	3	3	3	2	11	92%
7.2	Barrens Lake	182	2	1	0	3	3	3	2	2	10	83%
7.3	Lake Anna	202	5	0	0	5	3	3	3	2	11	92%
7.4	Sand Lake	61	0	1	0	1	3	3	3	1	10	83%
8	**Fumee Lake Natural Area**	728	6	1	1	7						
8.1	Little Fumee Lake	40	3	1	0	3	2	2	2	3	9	75%
8.2	Fumee Lake	324	3	0	1	4	3	2	3	3	11	92%

[1]Ratings 1 = low, 2 = medium, 3 = high; percent of total ratings = n/12.

Table S2. Site level characteristics and ratings for the Chicago study area.[1]

Nbr.	Place, Site Name	Area (ha)	N Foot	N Paddle	N Bike	Total Trails	Beauty	Integrity	Tranquility	Accessibility	Total Score	Pct.
9	**West Ridge/North Park Neighborhood Natural Areas**	526	11	0	1	12						
9.1	North Park Village	24	5	0	0	5	3	2	2	3	10	83%
9.2	West Ridge Nature Park/Rosehill Cemetery	142	4	0	0	4	3	2	2	3	10	83%
9.3	Indian Boundary Park	5	2	0	0	2	2	2	2	3	9	75%
9.4	Multi-Sites	526	0	0	1	1	1	1	1	2	5	42%
10	**Loyola Lakeshore Natural Areas**	34	5	1	1	7						
10.1	Loyola University Lakeshore Campus	14	1	0	0	1	3	3	2	3	11	92%
10.2	Loyola-Leone Parks	16	3	0	0	3	3	2	2	3	10	83%
10.3	Multi-Sites	20	1	1	1	3	2	2	2	3	9	75%
11	**Lincoln Park North Natural Areas**	81	6	1	1	8						
11.1	Montrose Point	11	4	0	0	4	3	2	3	3	11	92%
11.2	Marovitz Savanna	4	1	0	0	1	2	2	1	3	8	67%
11.3	Bill Jarvis Migratory Bird Sanctuary	4	1	0	0	1	2	2	2	3	9	75%
11.4	Multi-Sites	81	0	1	1	2	2	2	2	2	8	67%
12	**Lincoln Park South Natural Areas**	53	4	0	1	5						
12.1	Alfred Caldwell Lily Pool	1	1	0	0	1	3	3	3	3	12	100%
12.2	Nature Museum	0.4	1	0	0	1	3	3	1	3	10	83%
12.3	North Pond	5	1	0	0	1	3	2	2	3	10	83%
12.4	Lincoln Park Zoo	5	1	0	0	1	3	3	1	3	10	83%
12.5	Multi-Sites	53	0	0	1	1	2	2	1	3	8	67%
13	**Caldwell Preserves**	150	4	1	1	6						
13.1	Sidney Yates Flatwoods	40	2	0	0	2	3	3	2	3	11	92%
13.2	Oxbow Prairie	3	1	0	0	1	2	3	3	3	11	92%
13.3	Bunker North Flatwoods	31	1	0	0	1	2	2	2	3	9	75%
13.4	Multi-Sites	101	0	1	1	2	3	2	2	3	10	83%
14	**Harms Woods Preserves**	134	4	1	1	6						
14.1	Harms Woods Nature Preserve	68	4	0	0	4	3	2	3	3	11	92%
14.2	Multi-Sites	93	0	1	1	2	3	2	2	3	10	83%
15	**Skokie Marsh**	504	9	3	2	14						
15.1	Skokie Lagoons Forest Preserve	348	4	3	1	8	2	1	2	2	7	58%
15.2	Chicago Botanic Garden	156	5	0	1	6	3	3	2	3	11	92%
16	**Somme Preserves**	153	8	0	0	8						
16.1	Somme Prairie	28	1	0	0	1	3	3	2	3	11	92%
16.2	Somme Prairie Grove	34	4	0	0	4	3	3	2	2	10	83%
16.3	Somme Woods	91	3	0	0	3	3	3	2	3	11	92%

[1]Ratings 1 = low, 2 = medium, 3 = high; percent of total ratings = n/12.

Table S3. Trail level characteristics and ratings for the Northwoods study area.[1]

Nbr.	Place, Site, Trail Name	Type	Length (km)	Ease of Travel	Attractive Layout	Natural Features	Built Features	Explore Nature	Interp. & Steward.	Total Score	Pct .
**1**	**Pine-Popple Wild Rivers**										
1.1	***Wild Rivers Interpretive Center***										***58%***
1.11	Return of Trees Interpretive Trail	Foot	1.1	3	2	2	3	2	3	15	83%
1.2	***Pine River Flats***										***92%***
1.21	Pine River Flats Paddle	Paddle	5.3	2	3	3	1	3	1	13	72%
1.3	***Pine River Outcrops***										***67%***
1.31	Outcrops-River Loop	Foot	1.3	1	3	3	1	3	1	12	67%
1.32	Outcrops-Western Trail	Foot	1.6	2	3	3	1	3	1	13	72%
1.4	***Pine River- Meyers Falls/Bull Falls***										***92%***
1.41	Meyers Falls Trail	Foot	0.3	2	3	3	1	3	1	13	72%
1.42	Bull Falls Trail	Foot	0.8	2	3	3	1	3	1	13	72%
1.43	Goodman Grade-Falls Bike	Bike	12.9	2	2	3	1	3	1	12	67%
1.5	***Pine River- LaSalle Falls***										***75%***
1.51	LaSalle Falls Trail	Foot	3.2	2	3	3	2	2	1	13	72%
1.6	***Pine River-Breakwater Falls/Pine River Flowage***										***67%***
1.61	Breakwater Falls North Bank	Foot	0.8	2	3	3	2	3	1	14	78%
1.62	Breakwater Falls South Bank	Foot	1.5	2	3	3	2	3	1	14	78%
1.63	Pine River Flowage Paddle	Paddle	1.6	2	2	3	2	3	1	13	72%
1.7	***Pine River Oxbow***										***92%***
1.71	Oxbow Paddle	Paddle	5.6	3	3	3	2	3	1	15	83%
1.8	***Savage-Robago Wild Lakes Complex***										***92%***
1.81	Savage Lake Shoreline Walk	Foot	1.0	3	2	3	1	3	1	13	72%
1.82	Savage Lake Paddle	Paddle	0.8	2	2	3	1	3	1	12	67%
1.83	Wild Lakes Land-Water Loop	Foot	2.4	2	3	3	1	3	1	13	72%
1.84	Wild Lakes Bike Loop	Bike	20.9	2	1	2	1	2	1	9	50%
1.85	Savage Lake Road	Bike	12.1	3	2	3	1	2	1	12	67%
**2**	**Florence County Forest and Parks**										
2.1	***Sea Lion Lake***										***83%***
2.11	Point Trail	Foot	0.3	2	3	3	1	3	1	13	72%
2.12	Loop Trail	Foot	1.3	3	2	1	1	2	1	10	56%
2.13	Ridge Trail	Foot	2.6	2	2	2	1	3	1	11	61%
2.14	Sea Lion Lake Islands and Bays Paddle	Paddle	0.5	3	3	3	1	3	1	14	78%
2.2	***Lake Emily Recreation Trail***										***83%***
2.21	North Loop	Foot	3.1	2	2	2	1	2	1	10	56%
2.22	South Loop	Foot	1.8	3	2	1	1	2	1	10	56%
2.23	South Loop Little Lake Emily	Foot	1.2	1	2	3	1	2	1	10	56%
2.24	Little Lake Emily Paddle	Paddle	0.8	2	3	3	1	2	1	12	67%
2.3	***Halls Creek Trails***										***75%***
2.31	Red Loop	Foot	1.6	3	2	2	1	2	1	11	61%
2.32	Green Loop	Foot	2.4	2	2	1	1	2	1	9	50%
2.33	Blue Loop	Bike	4.7	3	2	2	1	2	1	11	61%

**3**					**Rainbow Trail**						
3.1					***Fox Maple Woods***						***92%***
3.11	Main Trail	Foot	0.8	3	2	3	1	2	1	12	67%
3.12	Marsh Loop	Foot	0.8	2	2	3	1	3	1	12	67%
3.13	Upland Loop	Foot	1.0	2	2	3	1	3	1	12	67%
3.14	Big Loop	Foot	1.3	2	3	3	1	3	1	13	72%
3.2					***Perch Lake***						***92%***
3.21	Loop Trail North Portion (to site 2)	Foot	1.9	2	2	3	2	3	1	13	72%
3.22	Full Loop Trail (winter only)	Foot	3.7	2	2	3	2	3	1	13	72%
3.23	Perch Lake Paddle	Paddle	0.8	2	3	3	2	3	1	14	78%
3.3					***CCC Camp Rainbow***						***75%***
3.31	Camp Perimeter Loop	Foot	0.8	2	1	2	1	3	1	10	56%
3.32	Rainbow Fire Tower	Foot	1.6	3	2	2	1	2	1	11	61%
3.4					***Whisker Lake Wilderness***						***83%***
3.41	Whisker Lake Trail Portion	Foot	4.4	2	2	3	1	3	1	12	67%
3.42	Riley Lake Trail Portion	Foot	4.2	1	2	3	1	3	1	11	61%
3.43	Edith Lake Paddle	Paddle	0.8	3	2	3	1	3	1	13	72%
3.44	Edith Lake-- Montagne Creek Paddle	Paddle	1.6	3	3	3	1	3	1	14	78%
3.5					***Rainbow Hunter Walking Trails***						***92%***
3.51	North Loop	Foot	3.4	2	3	3	1	3	1	13	72%
3.52	Middle Loop	Foot	2.2	2	3	3	1	3	1	13	72%
3.53	South Loop	Foot	3.2	2	3	3	1	3	1	13	72%
3.54	West Trail/Rainbow Creek	Foot	2.4	2	3	3	1	3	1	13	72%
3.6					***Multi-Sites***						***75%***
3.61	Rainbow Trail CCC Sites Bike Tour	Bike	4.8	2	2	2	1	2	1	10	56%
**4**					**Lauterman/Lost Lake Recreation Areas**						
4.1					***Lauterman National Recreation Trail***						***100%***
4.11	Beginner's Trail Loop	Foot	1.5	3	3	3	1	3	1	14	78%
4.12	Lauterman Lake Trail Loop	Foot	3.5	2	3	3	2	3	1	14	78%
4.13	Chipmunk-Little Porky Loop	Bike	11.9	2	3	3	1	3	1	13	72%
4.2					***Lost Lake Recreation Area***						***100%***
4.21	Lakeshore Trail Loop	Foot	2.6	2	3	3	3	3	3	17	94%
4.22	Assessor's Interpretive Trail Loop	Foot	1.5	3	2	3	2	2	2	14	78%
4.23	Lakeshore-Ridge Trail Loop	Foot	4.2	2	3	3	3	3	3	17	94%
4.24	CCC Cabins to Ridge Trail Loop	Foot	1.5	3	3	3	3	3	3	18	100%
4.25	West Lakeshore Trail	Foot	1.6	3	3	3	3	3	3	18	100%
4.26	Lost Lake Paddle	Paddle	1.1	3	2	3	2	2	1	13	72%
**5**					**Brule River Cliffs**						
5.1					***Brule River Cliffs***						***83%***
5.11	Old Field Loop	Foot	2.0	3	2	3	1	3	1	13	72%
5.12	Cliffs-River Loop	Foot	2.8	1	3	3	1	3	1	12	67%
5.13	Brule River Cliffs Paddle	Paddle	8.1	1	3	3	1	3	1	12	67%
5.14	Brule River Cliffs Bike Loop	Bike	17.7	1	2	2	1	2	1	9	50%

**6**					**Hidden Lakes**						
6.1					***Hidden Lakes Trail***						***92%***
6.11	Franklin Nature Trail	Foot	1.6	3	3	3	3	3	2	17	94%
6.12	Franklin Nature Trail - Two Dutchmen Lake Loop	Foot	4.8	2	3	3	2	3	2	15	83%
6.13	McKinley Lake - Three Johns Lake	Foot	3.2	2	3	3	2	3	1	14	78%
6.14	McKinley Lake - Luna Lake	Foot	3.2	2	3	3	2	3	1	14	78%
6.15	Luna Lake Trail	Foot	3.2	2	3	3	2	3	1	14	78%
6.16	White Deer Trail	Foot	2.3	2	3	3	2	3	1	14	78%
6.2					***Hidden Lakes Dispersed Sites***						***83%***
6.21	Three Johns Lake Paddle	Paddle	1.2	3	3	3	2	3	1	15	83%
6.22	McKinley Lake Paddle	Paddle	1.2	3	3	3	2	3	1	15	83%
6.23	Two Sisters Lake Bog Walk	Foot	0.3	3	2	3	1	3	1	13	72%
6.24	Quartz Lake Paddle	Paddle	1.1	3	2	3	2	3	1	14	78%
6.3					***Other Area Trails of Interest***						***92%***
6.31	Healing Nature Trail	Foot	0.6	3	3	3	3	3	3	18	100%
6.32	Sam Campbell Memorial Trail	Foot	3.4	2	2	3	2	3	2	14	78%
**7**					**Spread Eagle Barrens**						
7.1					***Fire Lane Road***						***92%***
7.11	Fire Lane Rd. Forest Edge-Barrens Short Loop	Foot	2.2	2	2	3	1	2	1	11	61%
7.12	Fire Lane Rd. Loop	Foot	5.6	1	2	3	1	2	1	10	56%
7.13	Fire Lane Rd. to Menominee R. Bike Loop	Bike	5.6	2	2	3	1	2	1	11	61%
7.2					***Barrens Lake***						***83%***
7.21	Short Loop	Foot	2.2	2	2	3	1	2	1	11	61%
7.22	Full Loop	Foot	3.7	2	2	3	1	2	1	11	61%
7.23	Barrens Lake Paddle	Paddle	0.8	3	2	3	1	2	1	12	67%
7.3					***Lake Anna***						***92%***
7.31	West Loop	Foot	3.0	2	3	3	1	2	1	12	67%
7.32	East Loop	Foot	2.7	2	2	3	1	2	1	11	61%
7.33	Full Loop	Foot	3.6	2	3	3	1	2	1	12	67%
7.34	Bog Loop	Foot	0.6	3	2	3	1	3	1	13	72%
7.35	Lepage Creek Overlook	Foot	0.0	3	3	3	2	1	1	13	72%
7.4					***Sand Lake***						***83%***
7.41	Sand Lake Paddle	Paddle	0.8	3	3	3	1	2	1	13	72%
**8**					**Fumee Lake Natural Area**						
8.1					***Little Fumee Lake - South Ridge***						***75%***
8.11	Little Fumee Lake Loop	Foot	2.5	2	1	3	2	2	2	12	67%
8.12	Little Fumee Shoreline Paddle	Paddle	0.8	3	1	3	2	2	2	13	72%
8.13	South Ridge Loop	Foot	5.2	1	2	2	1	2	2	10	56%
8.14	South Ridge-South Loop Pt.	Foot	3.7	2	1	2	1	2	2	10	56%
8.2					***Fumee Lake***						***92%***
8.21	Fumee Lake-North Ridge Trail	Foot	5.3	1	2	3	2	2	2	12	67%
8.22	Fumee Lake-Mountain Trail	Foot	5.3	1	2	3	2	2	2	12	67%
8.23	Mountain-North Ridge Short	Foot	1.6	2	2	2	1	2	2	11	61%
8.24	Big Fumee Lake Bike Loop	Bike	8.1	3	2	3	2	2	2	14	78%

[1]Ratings 1 = low, 2 = medium, 3 = high; percent of total ratings = n/18. Percentages in bold italics are for site level ratings of beauty, integrity, tranquility, and accessibility (see tables S1 and S2 for details).

Table S4. Trail level characteristics and ratings for the Chicago study area.[1]

Nbr.	Trail Name	Type	Length (km)	Ease of Travel	Attractive Layout	Natural Features	Built Features	Explore Nature	Interp. & Steward.	Total Score	Pct . (n/18)
**9**	**West Ridge/North Park Neighborhood Natural Areas**										
9.1	***North Park Village***										***83%***
9.11	Nature Center Wetland Loop	Foot	0.5	3	2	3	3	2	3	16	89%
9.12	Nature Center Woodland Loop	Foot	0.8	3	2	3	3	2	3	16	89%
9.13	Nature Center Wetland-Woodland-Savanna Loop	Foot	1.2	3	3	3	3	2	3	17	94%
9.14	Rock Garden Loop	Foot	0.2	3	2	3	3	2	1	14	78%
9.15	Walking Stick Woods Trail/Nature Place Space	Foot	0.8	3	2	2	3	3	2	15	83%
9.2	***West Ridge Nature Park/Rosehill Cemetery***										***83%***
9.21	Nature Park Woodland Loop	Foot	0.7	3	2	2	3	2	3	15	83%
9.22	Nature Park Lake Loop	Foot	1.1	3	2	3	3	2	3	16	89%
9.23	Woodland-Lake Loop	Foot	1.8	3	3	3	3	2	3	17	94%
9.24	Rosehill Cemetery Tree Loop	Foot	1.6	3	2	3	2	1	1	12	67%
9.3	***Indian Boundary Park***										***75%***
9.31	Natural Area Loop	Foot	0.3	3	2	2	3	1	1	12	67%
9.32	Park Loop	Foot	0.7	3	2	2	3	2	2	14	78%
9.4	***Multi-Sites***										***42%***
9.41	Neighborhood Natural Areas Bike Loop	Bike	6.8	2	2	2	3	1	1	11	61%
**10**	**Loyola Lakeshore Natural Areas**										
10.1	***Loyola University Lakeshore Campus***										***92%***
10.11	Lakeshore Campus Loop Walk	Foot	0.5	3	2	3	3	1	2	14	78%
10.2	***Loyola-Leone Parks***										***83%***
10.21	Loyola Natural Area-Park Loop	Foot	0.8	3	2	3	3	2	2	15	83%
10.22	Loyola Natural Area-Pier Loop	Foot	0.8	3	2	3	1	1	2	12	67%
10.23	Leone Natural Area-Park Loop	Foot	0.4	3	2	3	2	1	2	13	72%
10.3	***Multi-Sites***										***75%***
10.31	Hartigan-Leone Beach Walk	Foot	2.2	2	2	3	2	3	2	14	78%
10.32	Loyola Waterfront Paddle	Paddle	1.4	2	2	3	1	2	1	11	61%
10.33	Loyola Waterfront Bike Route	Bike	3.2	3	3	3	2	2	1	14	78%
**11**	**Lincoln Park North Natural Areas**										
11.1	***Montrose Point***										***92%***
11.11	Bird Sanctuary Main Loop	Foot	0.6	3	2	3	1	2	3	14	78%
11.12	Bird Sanctuary and Dunes Loop	Foot	1.2	3	3	3	1	2	3	15	83%
11.13	Point-Dunes-Lake-Prairie Loop	Foot	1.4	3	3	3	1	2	3	15	83%
11.14	Breakwater-Pier	Foot	2.4	2	1	2	1	1	1	8	44%
11.2	***Marovitz Savanna***										***67%***
11.21	Savanna Loop	Foot	0.9	3	2	2	1	1	2	11	61%
11.3	***Bill Jarvis Migratory Bird Sanctuary***										***75%***
11.31	Bird Sanctuary Loop	Foot	0.6	3	2	3	2	1	3	14	78%
11.4	***Multi-Sites***										***67%***
11.41	Montrose Waterfront Paddle	Paddle	1.6	2	2	2	1	2	1	10	56%
11.42	Montrose Area Bike Loop	Bike	7.6	3	3	3	3	2	1	15	83%

**12**	**Lincoln Park South Natural Areas**										
12.1	***Alfred Caldwell Lily Pool***										***100%***
12.11	Lily Pool Loop	Foot	0.4	3	3	3	3	2	2	16	89%
12.2	***Nature Museum***										***83%***
12.21	Deb Lahey Nature Trails	Foot	0.5	3	2	3	3	2	3	16	89%
12.3	***North Pond***										***83%***
12.31	Natural Area Loop	Foot	1.1	3	2	3	3	2	3	16	89%
12.4	***Lincoln Park Zoo***										***83%***
12.41	Nature Boardwalk	Foot	0.8	3	2	3	3	2	2	15	83%
12.5	***Multi-Sites***										***67%***
12.51	Fullerton Area Bike Loop	Bike	4.0	3	3	3	3	1	1	14	78%
**13**	**Caldwell Preserves**										
13.1	***Sidney Yates Flatwoods***										***92%***
13.11	Savanna-Flatwoods-Loop	Foot	0.8	3	2	2	1	2	2	12	67%
13.12	Savanna-Flatwoods-River Loop	Foot	1.2	3	3	3	1	2	2	14	78%
13.2	***Oxbow Prairie***										***92%***
13.21	Prairie Loop	Foot	0.5	2	2	2	2	2	2	12	67%
13.3	***Bunker North Flatwoods***										***75%***
13.31	Flatwoods meander	Foot	1.1	3	2	2	2	1	2	12	67%
13.4	***Multi-Sites***										***83%***
13.41	Caldwell Preserves Chicago River Paddle	Paddle	3.2	2	2	3	2	3	1	13	72%
13.42	Caldwell Preserves Bike Tour	Bike	7.1	3	3	3	2	2	1	14	78%
**14**	**Harms Woods Preserves**										
14.1	***Harms Woods Nature Preserve***										***92%***
14.11	Woodland-River West Loop	Foot	1.6	3	2	3	2	2	2	14	78%
14.12	Woodland-River East Loop	Foot	1.6	3	2	3	2	2	2	14	78%
14.13	Woodland-Meadow Loop	Foot	1.2	3	3	3	2	2	2	15	83%
14.14	Woodland-River-Meadow Full Loop	Foot	3.9	2	3	3	2	2	2	14	78%
14.2	***Multi-Sites***										***83%***
14.21	Harms Woods Chicago River Paddle	Paddle	3.2	2	2	3	1	3	1	12	67%
14.22	Harms Woods-Flatwoods Bike Loop	Bike	4.4	3	2	3	2	2	1	13	72%
**15**	**Skokie Marsh**										
15.1	***Skokie Lagoons Forest Preserve***										***58%***
15.11	Inner Trail- Main Trail	Foot	1.6	3	2	3	2	2	1	13	72%
15.12	Inner Trail- North Loop	Foot	2.6	1	1	2	2	1	1	8	44%
15.13	Inner Trail- South Loop	Foot	4.8	1	1	2	2	1	1	8	44%
15.14	Inner Trail- Full Loop	Foot	6.6	1	1	2	2	1	1	8	44%
15.15	Skokie Lagoons Paddle - Lagoons 4-5 Loop	Paddle	4.8	3	3	3	2	3	1	15	83%
15.16	Skokie Lagoons Paddle - Lagoon 3 Loop	Paddle	1.6	3	3	3	2	3	1	15	83%
15.17	Skokie Lagoons Paddle - Lagoons 1-3 Loop	Paddle	7.2	3	3	3	2	3	1	15	83%
15.18	Skokie Lagoons North Branch Trail Bike Loop	Bike	7.1	3	2	3	2	1	1	12	67%

15.2	***Chicago Botanic Garden***										***92%***
15.21	McDonald Woods South Loop	Foot	0.4	3	2	2	3	2	3	15	83%
15.22	McDonald Woods North Loop	Foot	0.6	3	2	2	1	2	3	13	72%
15.23	McDonald Woods Big Loop	Foot	1.1	3	2	2	3	2	3	15	83%
15.24	Japanese Garden	Foot	0.6	3	3	3	3	2	3	17	94%
15.25	Dixon Prairie	Foot	1.5	3	2	3	3	2	3	16	89%
15.26	Chicago Botanic Garden Bike Trail	Bike	3.2	3	2	2	2	1	3	13	72%
**16**	**Somme Preserves**										
16.1	***Somme Prairie***										***92%***
16.11	Prairie Loop	Foot	1.2	3	2	2	1	2	2	12	67%
16.2	***Somme Prairie Grove***										***83%***
16.21	Vestal Grove Savanna Loop	Foot	1.1	2	2	3	1	2	3	13	72%
16.22	Prairie-Grove Loop	Foot	1.9	2	3	3	1	2	3	14	78%
16.23	Prairie Inner Loop	Foot	2.0	2	2	2	1	2	3	12	67%
16.24	Prairie Outer Loop	Foot	2.4	2	2	2	1	2	3	12	67%
16.3	***Somme Woods***										***92%***
16.31	West Inner Loop	Foot	0.5	3	2	3	2	2	2	14	78%
16.32	West Outer Loop	Foot	1.0	3	2	3	2	2	2	14	78%
16.33	East Loop	Foot	2.1	2	2	3	2	2	2	13	72%

[1]Ratings 1 = low, 2 = medium, 3 = high; percent of total ratings = n/18. Percentages in bold italics are for site level ratings of beauty, integrity, tranquility, and accessibility (see tables S1 and S2 for details).

Table S5. Comparison of site level ratings for Northwoods versus Chicago study areas.[1]

**Beauty**	**Northwoods (*n*=29)**	**%**	**Chicago (*n*=27)**	**%**	**All Sites (*n*=56)**	**%**
High	22	75.9%	17	63.0%	39	69.6%
Medium	7	24.1%	9	33.3%	16	28.6%
Low	0	0.0%	1	3.7%	1	1.8%
Mean	2.76		2.59		2.68	
SD	0.435		0.572		0.508	
Mean Rank	30.36		26.50			
K-W H	1.23					
**Integrity**						
High	16	55.2%	10	37.0%	26	46.4%
Medium	12	41.4%	15	55.6%	27	48.2%
Low	1	3.4%	2	7.4%	3	5.4%
Mean	2.52		2.30		2.41	
SD	0.574		0.609		0.596	
Mean Rank	31.10		25.70			
K-W H	1.95					
**Tranquility**						
High	19	65.5%	4	14.8%	23	41.1%
Medium	9	31.0%	18	66.7%	27	48.2%
Low	1	3.4%	5	18.5%	6	10.7%
Mean	2.62		1.96		2.30	
SD	0.561		0.587		0.658	
Mean Rank	35.81		20.65			
K-W H	14.78***					
**Accessibility**						
High	10	34.5%	23	85.2%	33	58.9%
Medium	16	55.2%	4	14.8%	20	35.7%
Low	3	10.3%	0	0.0%	3	5.4%
Mean	2.24		2.85		2.54	
SD	0.636		0.362		0.602	
Mean Rank	21.45		36.07			
K-W H	14.99***					

[1]Kruskal-Wallis H significance levels: *$p < .05$, **$p < .005$, ***$p < .001$

Table S6. Comparison of trail level ratings for Northwoods versus Chicago study areas for all trails.[1]

**Study Area**	**Northwoods (*n*=91)**	**%**	**Chicago (*n*=66)**	**%**	**All Trails (*n*=157)**	**%**
			**Ease of Travel**			
High	31	34.1%	49	74.2%	80	51.0%
Medium	50	54.9%	14	21.2%	64	40.8%
Low	10	11.0%	3	4.5%	13	8.3%
Mean	2.23		2.70		2.43	
SD	0.634		0.544		0.643	
Mean Rank	65.80		97.20			
K-W H	22.84***					
			**Attractiveness of Layout**			
High	42	46.2%	17	25.8%	59	37.6%
Medium	44	48.4%	45	68.2%	89	56.7%
Low	5	5.5%	4	6.1%	9	5.7%
Mean	2.41		2.20		2.32	
SD	0.596		0.533		0.578	
Mean Rank	85.46		70.09			
K-W H	5.72*					
			**Natural Features**			
High	75	82.4%	45	68.2%	120	76.4%
Medium	13	14.3%	21	31.8%	34	21.7%
Low	3	3.3%	0	0.0%	3	1.9%
Mean	2.79		2.68		2.75	
SD	0.483		0.469		0.479	
Mean Rank	83.35		73.00			
K-W H	3.65					
			**Built and Borrowed Features**			
High	7	7.7%	23	34.8%	30	19.1%
Medium	26	28.6%	26	39.4%	52	33.1%
Low	58	63.7%	17	25.8%	75	47.8%
Mean	1.44		2.09		1.71	
SD	0.636		0.779		0.768	
Mean Rank	64.18		99.43			
K-W H	27.13***					
			**Explorable Nature**			
High	54	59.3%	7	10.6%	61	38.9%
Medium	36	39.6%	43	65.2%	79	50.3%
Low	1	1.1%	16	24.2%	17	10.8%
Mean	2.58		1.86		2.28	
SD	0.518		0.579		0.649	
Mean Rank	98.01		52.79			
K-W H	46.57***					
			**Interpretation and Stewardship**			
High	6	6.6%	22	33.3%	28	17.8%
Medium	12	13.2%	22	33.3%	34	21.7%
Low	73	80.2%	22	33.3%	95	60.5%
Mean	1.26		2.00		1.57	
SD	0.574		0.823		0.778	
Mean Rank	62.80		101.33			
K-W H	36.03***					

[1]Kruskal-Wallis H significance levels: $*p < .05$, $**p < .005$, $***p < .001$.

Table S7. Comparison of trail level ratings for Northwoods versus Chicago study areas for foot trails.[1]

Study Area	Northwoods (n=65)	%	Chicago (n=51)	%	All Trails (n=116)	%
**Ease of Travel**						
High	17	26.2%	39	76.5%	56	48.3%
Medium	40	61.5%	9	17.6%	49	42.2%
Low	8	12.3%	3	5.9%	11	9.5%
Mean	2.14		2.71		2.39	
SD	0.609		0.576		0.656	
Mean Rank	46.04		74.38			
K-W H	25.02***					
**Attractiveness of Layout**						
High	31	47.7%	10	19.6%	41	35.3%
Medium	31	47.7%	37	72.5%	68	58.6%
Low	3	4.6%	4	7.8%	7	6.0%
Mean	2.43		2.12		2.29	
SD	0.585		0.516		0.575	
Mean Rank	65.76		49.25			
K-W H	9.14**					
**Natural Features**						
High	53	81.5%	33	64.7%	86	74.1%
Medium	9	13.8%	18	35.3%	27	23.3%
Low	3	4.6%	0	0.0%	3	2.6%
Mean	2.77		2.65		2.75	
SD	0.523		0.483		0.507	
Mean Rank	62.38		53.56			
K-W H	3.39					
**Built and Borrowed Features**						
High	7	10.8%	20	39.2%	27	23.3%
Medium	17	26.2%	17	33.3%	34	29.3%
Low	41	63.1%	14	27.5%	55	47.4%
Mean	1.48		2.12		1.76	
SD	0.687		0.816		0.809	
Mean Rank	47.72		72.25			
K-W H	17.77***					
**Explorable Nature**						
High	40	61.5%	2	3.9%	42	36.2%
Medium	24	36.9%	37	72.5%	61	52.6%
Low	1	1.5%	12	23.5%	13	11.2%
Mean	2.60		1.80		2.25	
SD	0.524		0.491		0.644	
Mean Rank	75.12		37.31			
K-W H	44.83***					
**Interpretation and Stewardship**						
High	6	9.2%	21	41.2%	27	23.3%
Medium	10	15.4%	22	43.1%	32	27.6%
Low	49	75.4%	8	15.7%	57	49.1%
Mean	1.34		2.25		1.74	
SD	0.644		0.717		0.814	
Mean Rank	42.68		78.67			
K-W H	38.60***					

[1]Kruskal-Wallis H significance levels: *$p < .05$, **$p < .005$, ***$p < .001$

Table S8. Comparison of trail level ratings for Northwoods versus Chicago study areas for paddle trails.[1]

Study Area	Northwoods (*n*=17)	%	Chicago (*n*=7)	%	All Trails (*n*=24)	%
**Ease of Travel**						
High	11	64.7%	3	42.9%	14	58.3%
Medium	5	29.4%	4	57.1%	9	37.5%
Low	1	5.9%	0	0.0%	1	4.2%
Mean	2.59		2.43		2.54	
SD	0.618		0.535		0.588	
Mean Rank	13.15		10.93			
K-W H	0.650					
**Attractiveness of Layout**						
High	10	58.8%	3	42.9%	13	54.2%
Medium	6	35.3%	4	57.1%	10	41.7%
Low	1	5.9%	0	0.0%	1	4.2%
Mean	2.53		2.43		2.50	
SD	0.624		0.535		0.590	
Mean Rank	12.94		11.43			
K-W H	0.30					
**Natural Features**						
High	17	100.0%	6	85.7%	23	95.8%
Medium	0	0.0%	1	14.3%	1	4.2%
Low	0	0.0%	0	0.0%	0	0.0%
Mean	3.00		2.86		2.96	
SD	0.000		0.378		0.204	
Mean Rank	13.00		11.29			
K-W H	2.43					
**Built and Borrowed Features**						
High	0	0.0%	0	0.0%	0	0.0%
Medium	8	47.1%	4	57.1%	12	50.0%
Low	9	52.9%	3	42.9%	12	50.0%
Mean	1.47		1.57		1.50	
SD	0.514		0.535		0.511	
Mean Rank	12.15		13.36			
K-W H	0.19					
**Explorable Nature**						
High	12	70.6%	5	71.4%	17	70.8%
Medium	5	29.4%	2	28.6%	7	29.2%
Low	0	0.0%	0	0.0%	0	0.0%
Mean	2.71		2.71		2.71	
SD	0.470		0.488		0.464	
Mean Rank	12.47		12.57			
K-W H	0.01					
**Interpretation and Stewardship**						
High	0	0.0%	0	0.0%	0	0.0%
Medium	1	5.9%	0	0.0%	1	4.2%
Low	16	94.1%	7	100.0%	23	95.8%
Mean	1.06		1.00		1.04	
SD	0.243		0.000		0.204	
Mean Rank	12.71		12.00			
K-W H	0.41					

[1]Kruskal-Wallis H significance levels: *$p < .05$, **$p < .005$, ***$p < .001$

Table S9. Comparison of trail level ratings for Northwoods versus Chicago study areas for bike trails.[1]

**Study Area**	**Northwoods (*n*=9)**	**%**	**Chicago (*n*=8)**	**%**	**All Trails (*n*=17)**	**%**
**Ease of Travel**						
High	3	33.3%	7	87.5%	10	58.8%
Medium	5	55.6%	1	12.5%	6	35.3%
Low	1	11.1%	0	0.0%	1	5.9%
Mean	2.22		2.87		2.53	
SD	0.667		0.354		0.624	
Mean Rank	6.78		11.5			
K-W H	4.91*					
**Attractiveness of Layout**						
High	1	11.1%	4	50.0%	5	29.4%
Medium	7	77.8%	4	50.0%	11	64.7%
Low	1	11.1%	0	0.0%	1	5.9%
Mean	2.00		2.50		2.24	
SD	0.500		0.535		0.562	
Mean Rank	7.22		11			
K-W H	3.36					
**Natural Features**						
High	5	55.6%	6	75.0%	11	64.7%
Medium	4	44.4%	2	25.0%	6	35.3%
Low	0	0.0%	0	0.0%	0	0.0%
Mean	2.56		2.75		2.65	
SD	0.527		0.463		0.493	
Mean Rank	8.22		9.88			
K-W H	0.66					
**Built and Borrowed Features**						
High	0	0.0%	3	37.5%	3	17.6%
Medium	1	11.1%	5	62.5%	6	35.3%
Low	8	88.9%	0	0.0%	8	47.1%
Mean	1.11		2.38		1.71	
SD	0.333		0.518		0.772	
Mean Rank	5.28		13.19			
K-W H	12.24***					
**Explorable Nature**						
High	0	0.0%	1	12.5%	1	5.9%
Medium	1	11.1%	0	0.0%	1	5.9%
Low	8	88.9%	7	87.5%	15	88.2%
Mean	2.22		1.50		1.88	
SD	0.441		0.535		0.600	
Mean Rank	11.44		6.25			
K-W H	6.25*					
**Interpretation and Stewardship**						
High	0	0.0%	1	12.5%	1	5.9%
Medium	1	11.1%	0	0.0%	1	5.9%
Low	8	88.9%	7	87.5%	15	88.2%
Mean	1.11		1.25		1.18	
SD	0.333		0.707		0.529	
Mean Rank	8.89		9.13			
K-W H	0.03					

[1]Kruskal-Wallis H significance levels: *p ¸.05, **p < .005, ***p < .001
